# The Framingham Heart Study 100K SNP genome-wide association study resource: overview of 17 phenotype working group reports

**DOI:** 10.1186/1471-2350-8-S1-S1

**Published:** 2007-09-19

**Authors:** L Adrienne Cupples, Heather T Arruda, Emelia J Benjamin, Ralph B D'Agostino, Serkalem Demissie, Anita L DeStefano, Josée Dupuis, Kathleen M Falls, Caroline S Fox, Daniel J Gottlieb, Diddahally R Govindaraju, Chao-Yu Guo, Nancy L Heard-Costa, Shih-Jen Hwang, Sekar Kathiresan, Douglas P Kiel, Jason M Laramie, Martin G Larson, Daniel Levy, Chun-Yu Liu, Kathryn L Lunetta, Matthew D Mailman, Alisa K Manning, James B Meigs, Joanne M Murabito, Christopher Newton-Cheh, George T O'Connor, Christopher J O'Donnell, Mona Pandey, Sudha Seshadri, Ramachandran S Vasan, Zhen Y Wang, Jemma B Wilk, Philip A Wolf, Qiong Yang, Larry D Atwood

**Affiliations:** 1 National Heart Lung and Blood Institute's Framingham Heart Study, Framingham, MA, USA; 2 School of Public Health, Boston University, Boston, MA, USA; 3 School of Medicine, Boston University, Boston, MA, USA; 4 Whitaker Cardiovascular Institute, Boston University, Boston, MA, USA; 5 Department of Mathematics and Statistics, Boston University, Boston, MA, USA; 6 Department of Molecular and Cellular Biology, Harvard University, Cambridge, MA; 7 National Heart, Lung and Blood Institute, Bethesda, MD, USA; 8 VA Boston Healthcare System, Boston, MA, USA; 9 Broad Institute of Massachusetts Institute of Technology and Harvard University, Cambridge, MA, USA; 10 Cardiovascular Disease Prevention Center, Massachusetts General Hospital and Harvard Medical School, Boston, MA, USA; 11 Cardiology Division, Massachusetts General Hospital and Harvard Medical School, Boston, MA, USA; 12 Hebrew SeniorLife: Institute for Aging Research and Harvard Medical School, Boston, MA, USA; 13 Bioinformatics Program, Boston University, Boston, MA, USA; 14 National Center for Biotechnology Information, Bethesda, MD, USA; 15 General Medicine Division, Massachusetts General Hospital and Harvard Medical School, Boston, MA, USA; 16 Department of Medicine, Massachusetts General Hospital and Harvard Medical School, Boston, MA, USA

## Abstract

**Background:**

The Framingham Heart Study (FHS), founded in 1948 to examine the epidemiology of cardiovascular disease, is among the most comprehensively characterized multi-generational studies in the world. Many collected phenotypes have substantial genetic contributors; yet most genetic determinants remain to be identified. Using single nucleotide polymorphisms (SNPs) from a 100K genome-wide scan, we examine the associations of common polymorphisms with phenotypic variation in this community-based cohort and provide a full-disclosure, web-based resource of results for future replication studies.

**Methods:**

Adult participants (n = 1345) of the largest 310 pedigrees in the FHS, many biologically related, were genotyped with the 100K Affymetrix GeneChip. These genotypes were used to assess their contribution to 987 phenotypes collected in FHS over 56 years of follow up, including: cardiovascular risk factors and biomarkers; subclinical and clinical cardiovascular disease; cancer and longevity traits; and traits in pulmonary, sleep, neurology, renal, and bone domains. We conducted genome-wide variance components linkage and population-based and family-based association tests.

**Results:**

The participants were white of European descent and from the FHS Original and Offspring Cohorts (examination 1 Offspring mean age 32 ± 9 years, 54% women). This overview summarizes the methods, selected findings and limitations of the results presented in the accompanying series of 17 manuscripts. The presented association results are based on 70,897 autosomal SNPs meeting the following criteria: minor allele frequency ≥ 10%, genotype call rate ≥ 80%, Hardy-Weinberg equilibrium p-value ≥ 0.001, and satisfying Mendelian consistency. Linkage analyses are based on 11,200 SNPs and short-tandem repeats. Results of phenotype-genotype linkages and associations for all autosomal SNPs are posted on the NCBI dbGaP website at http://www.ncbi.nlm.nih.gov/projects/gap/cgi-bin/study.cgi?id=phs000007.

**Conclusion:**

We have created a full-disclosure resource of results, posted on the dbGaP website, from a genome-wide association study in the FHS. Because we used three analytical approaches to examine the association and linkage of 987 phenotypes with thousands of SNPs, our results must be considered hypothesis-generating and need to be replicated. Results from the FHS 100K project with NCBI web posting provides a resource for investigators to identify high priority findings for replication.

## Background

Cardiovascular diseases are major illnesses among Americans, affecting about a third of the population (79 million with prevalent disease) and resulting in more than 870,000 cardiovascular disease deaths annually [1]. Cardiovascular disease and its risk factors have substantial genetic contributors [2-11]. Numerous reports from the Framingham Heart Study (FHS) have documented that coronary heart disease [12,13], blood pressure [14-16], lipids [17-20], diabetes [21-23] and weight [24,25] have substantial heritability and linkage/association to specific genomic regions. To evaluate the genetic contributors to these phenotypes, the Framingham Heart Study conducted a genome-wide scan of 1345 study participants in two generations, using genotyping from the 100K Affymetrix GeneChip Human Mapping Set.

In this manuscript, we summarize the strategies that we pursued to conduct the 100K genome-wide study, providing an overview for a series of 17 companion manuscripts (Table 1 of the Overview) describing associations with specific collections of traits [26-42]. The primary purpose of this project was to generate hypotheses regarding genetic factors that may contribute to the wide spectrum of phenotypic variables collected in the FHS through a genome-wide approach. More specifically, we primarily hypothesized that common genetic variants contributing to phenotypic variation can be detected through a genome-wide association study (GWAS) and that genetic loci contributing to phenotypic variation can be detected through linkage. Each manuscript also examines whether the 100K analyses replicated previously reported associations with consistent evidence from the literature for some specific traits. The main purpose of this series of publications is to describe the association results made available for investigators and to direct readers to their free availability in the database of Genotype and Phenotype (dbGaP) public repository http://www.ncbi.nlm.nih.gov/projects/gap/cgi-bin/study.cgi?id=phs000007 at the National Center for Biotechnology Information (NCBI), where these comprehensive results are posted and may be browsed in the context of multiple genomic tracks including Entrez Gene, RefSeq, dbSNP, genetic markers, and OMIM. The deposition of these data in a public repository is consistent with the long tradition of publishing preliminary results from the FHS to benefit the wider scientific community.

**Table 1:** Papers published in the Framingham Heart Study 100K Series

First Author	Title
Ramachandran S. Vasan	Genome-wide association of echocardiographic dimensions, brachial artery endothelial function and treadmill exercise responses in the Framingham Heart Study
Daniel Levy	Framingham Heart Study 100K Project: genome-wide associations for blood pressure and arterial stiffness
Christopher J. O'Donnell	Genome-wide association study for subclinical atherosclerosis in major arterial territories in the NHLBI's Framingham Heart Study
Martin G. Larson	Framingham Heart Study 100K project: genome-wide associations for cardiovascular disease outcomes
Joanne M. Murabito	Genome-wide association study of breast and prostate cancer in the NHLBI's Framingham Heart Study
Christopher Newton-Cheh	Genome-wide association study of electrocardiographic and heart rate variability traits: the Framingham Heart Study
Jemma B. Wilk	Framingham Heart Study genome-wide association: results for pulmonary function measures
Daniel J. Gottlieb	Genome-wide association of sleep and circadian phenotypes
Shih-Jen Hwang	A genome-wide association for kidney function and endocrine-related traits in the NHLBI's Framingham Heart Study
Emelia J. Benjamin	Genome-wide association with select biomarker traits in the Framingham Heart Study
Qiong Yang	Genome-wide association and linkage analyses of hemostatic factors and hematological phenotypes in the Framingham Heart Study
Kathryn L. Lunetta	Genetic correlates of longevity and selected age-related phenotypes: a genome-wide association study in the Framingham Study
Douglas P. Kiel	Genome-wide association with bone mass and geometry in the Framingham Heart Study
Sudha Seshadri	Genetic correlates of brain aging on MRI and cognitive test measures: a genome-wide association and linkage analysis in the Framingham study
James B. Meigs	Genome-wide association with diabetes-related traits in the Framingham Heart Study
Sekar Kathiresan	A genome-wide association study for blood lipid phenotypes in the Framingham Heart Study
Caroline S. Fox	Genome-wide association to body mass index and waist circumference: the Framingham Heart Study 100K project

To organize the evaluation of the rich resource of data collected over nearly 60 years of follow up, we established a set of "Phenotype Working Groups" that included clinicians, epidemiologists, geneticists, and biostatisticians. These groups specified the traits to be studied, along with covariate adjustment and subgroups for analyses. In all, 987 phenotypes were examined for association, 835 for linkage. Some phenotypes are the same trait with different covariate adjustments, at different examinations or evaluated in different subgroups. For example, many traits were evaluated with both age and sex adjustment as well as with additional multivariable adjustments, yielding more than one phenotype for analysis. Each manuscript in this series provides a platform for the web posted results. Not every trait is described in the manuscripts; rather, the purpose of each manuscript is to introduce the trait areas and to present a brief summary of the results. In the present manuscript, we describe the general approach to analysis of the traits, provide an overview of some results, and discuss the limitations of the studies.

## Methods

### Study sample

The Framingham Heart Study (FHS) began in 1948 with recruitment of 5209 men and women (2336 men and 2873 women) between the ages of 28 and 62 years in the town of Framingham, Massachusetts, about 20 miles west of Boston [43-46]. These individuals were recruited through a two-thirds systematic sample of the households of Framingham, Massachusetts. Although not initially intended as a family study, many households consisted of spouse pairs (1644 pairs). The primary purpose of the Study was to follow individuals over time for development of cardiovascular disease events to evaluate the interplay among multiple risk factors that lead to disease and their individual and joint effects. The participants in the Original Cohort have been examined every two years since.

In 1971, an Offspring Cohort of 5124 men and women, who were adult children of Original Cohort members or were spouses of these offspring, was recruited and has been examined every four to eight years since [47,48]. The subjects in this report are drawn from the largest 310 pedigrees in these two generations. The participants were recruited without regard to phenotypes. Thus, the Offspring Cohort of 5124 (2483 men and 2641 women) was recruited by inviting all offspring of the spouse pairs (2616 and 34 stepchildren), the offspring spouses (1576) and, additionally, those offspring (898) of singleton Original Cohort members with elevated lipid levels. Further information regarding recruitment can be seen in Cupples *et al*. [49] and Dawber [43].

In the late 1980s and through the 1990s, DNA was collected from living study participants. As many of the Original Cohort members were deceased by that time, these DNAs were mostly collected in Offspring Study participants. During the mid- to late-1990s, 1702 DNA samples were genotyped by the Mammalian Genotyping Service in the largest 330 two-generation pedigrees consisting of 2885 Framingham Study participants. These pedigrees were used for linkage analyses of blood pressure [15], lipids [17,50], body mass index [25] and a wide variety of other traits [51-56]. The numbers of relative pairs among the 1345 subjects both genotyped and phenotyped in this study are 435 parent-offspring pairs, 988 sib pairs, 300 avuncular pairs and 634 first-cousin pairs. Among the 1087 Offspring Cohort participants, who were the only participants evaluated in some analyses, there were 936 sib pairs, 63 avuncular pairs and 612 first-cousin pairs.

Original Cohort study subjects return to the Study every two years for a detailed medical history, physical examination and laboratory tests. The Original Cohort subjects are currently in their 29^th ^examination. Participants in the Offspring Cohort return every 4 to 8 years for similar examinations and the 8^th ^examination is currently underway.

In the early 2000s, a family DNA plate set with 1,399 participants from these 330 pedigrees http://www.nhlbi.nih.gov/about/framingham/policies/index.htm was established. Only subjects with lymphoblast cell lines were included on the plate set, although a substantial number of the DNA samples on the plate set were derived from whole blood or buffy coat. The family plate set was used for genotyping of the Affymetrix 100K GeneChip. After cleaning the genotyping data, the study sample comprised 1345 FHS participants, 278 from the Original and 1087 Offspring Cohorts.

### Phenotype definition & methods

Given the breadth of phenotypes, we established 8 larger Phenotype Working Groups, overseeing 17 discrete phenotypic domains, as follows: 1. Blood pressure, arterial stiffness, echocardiography, endothelial function and exercise testing; 2. Metabolic traits including anthropometry, glycemic traits and lipids; 3. Pulmonary function and sleep; 4. Systemic biomarkers, including inflammatory and thrombotic factors; 5. Subclinical atherosclerosis; 6. Renal and endocrine function; 7. Longevity and aging, including brain and bone aging phenotypes; 8. Cardiovascular disease outcomes, cancer, electrocardiography and heart rate variability. In addition, we established a statistical and analytical methodology group. These groups were convened by the FHS Genetic Steering Committee to define phenotypes to be evaluated, including the covariates used in analyses, to review results of linkage and association analyses, to foster communication among various Framingham investigators who were working on different traits, and to suggest possible follow-up strategies.

For the 100K genome-wide project, each Working Group defined the phenotypes to be studied. Since most traits have well established factors that contribute to their variation, each group created a set of residuals from multivariable regression models accounting for the primary known covariates, in order to control for confounding from these variables and to increase the ability to detect genetic signals. For quantitative traits, the adjusted standardized residuals were generated using linear regression models. For qualitative traits, we used a variety of approaches including Cox proportional hazards with Martingale residuals for time-to-event (survival) traits and logistic regression with deviance residuals for dichotomous traits. These methods are described below. In some cases, several different covariate adjustments were used for a single trait. Each manuscript describes the specific adjustments that were applied. We used residuals from regression models that included all subjects with traits in each Cohort, rather than limiting analyses to those who were genotyped, to produce residuals based on all subjects with phenotypic values, regardless of availability of genotypic data. This approach avoids potential biases in covariate adjustment based only upon the subset of individuals with both genotype and phenotype data and produces robust estimates of covariate effects.

### Genotyping methods

Genomic DNA derived from whole blood or buffy coat was phenol-chloroform extracted and DNA from immortalized lymphoblast cell lines was salt-precipitate extracted. Genotyping of the 100K SNPs in FHS families was performed through an ancillary study to Drs. Michael Christman and Alan Herbert at Boston University School of Medicine in the Department of Genetics and Genomics using the GeneChip Human Mapping 100K set from Affymetrix, following the manufacturer's protocol as previously described [57]. Genotypes were determined using the Dynamic Modeling (DM) algorithm [58]. For linkage analyses, we also included microsatellites that had been genotyped by the NHLBI Mammalian Genotyping Service, Center for Medical Genetics, Marshfield Medical Research Foundation http://research.marshfieldclinic.org/genetics. A set of 401 microsatellite markers)[59], covering the genome at an average density of one marker every 10 cM and with an average heterozygosity of 0.77, were genotyped in 1702 subjects in the mid to late 1990s (Screening Set v. 8))[60]. An additional 190 participants on the Family Plate Set were genotyped later with microsatellites using Screening Set v.13 and some additional microsatellites were also genotyped in the FHS Genetics Laboratory. With the addition of these microsatellites and changes in the marker sets from Set 8, there were 613 microsatellite markers available for analysis.

### Statistical analysis methods

#### Data cleaning

A total of 1380 individuals were successfully genotyped. First, familial relationships were checked using the sib_kin utility in the Aspex software package [61]. Because this study focused on participants of families, nine individuals were excluded as they no longer had biologic relatives in the sample. Twenty-six individuals were excluded due to inconsistencies; the majority of these individuals were found to have an excessive number of Mendelian errors as identified by the software PedCheck, Version 1.1 [62]. Others were excluded for having a relationship inconsistency, for a sex discrepancy or for a low genotyping call rate. Mendelian inconsistencies were resolved by removing the genotypes of all individuals within nuclear families in which the error occurred. These steps left 1345 individuals with genotypes available for analyses.

For Hardy-Weinberg equilibrium (HWE) testing, we randomly selected one individual per family to form a sample of unrelated individuals. Then, for each of the 100K SNPs, the observed genotype frequencies were compared to those expected under HWE using an exact chi-square test statistic [63] implemented in the Genetics package [64] in R, Version 1.2.0 [65]. To guard against a result that might depend upon an unusual selection of individuals, we repeated this process of a random selection of subjects ten times and computed the geometric mean of the ten p-values for the ten random samples of individuals as the final p-value for HWE tests for each SNP. Tests for HWE that indicate a SNP is far from HWE suggest that the SNP may have issues with genotyping error.

We found 38,062 SNPs with MAF <10% (of which 3084 autosomal SNPs were monomorphic), an additional 2346 SNPs with genotyping call rates <80% and still another 1595 with HWE p-value < 0.001. We used a genotyping call rate cutpoint of 80% in part because of the use of the less accurate DM algorithm. SNPs with low MAF, low genotyping call rates and inadequate HWE produced unstable results, so they were excluded from our association results reported in this set of manuscripts, leaving 70,987 SNPs. Results for all autosomal SNPs are reported on the dbGaP website, regardless of MAF, genotyping call rates or HWE p-values; however, filters for these factors are provided on the dbGaP website.

#### Linkage analyses

Both microsatellites previously genotyped by the Mammalian Genotyping Service and SNPs from the 100K were used to calculate identity by descent probabilities. We constructed genetic maps using all microsatellite NCBI genetic markers with Marshfield genetic location available and whose physical order and genetic order were consistent. Using this NCBI Marshfield map as our skeleton, we applied linear interpolation from physical to genetic distance to obtain approximate genetic locations (in centiMorgans) for all SNPs in the 100K set with known physical location.

Because current linkage analysis software cannot handle the marker density available from a 100K scan, we selected a subset of 10,592 SNPs to supplement 613 genome scan microsatellite markers available on 1886 members of the largest 330 Framingham families. We selected SNPs to minimize linkage disequilibrium (LD) because current linkage software assumes that markers are in linkage equilibrium, and violation of this assumption has been shown to create spurious linkage evidence in certain contexts [66,67]. Thus, for calculation of identity by descent (IBD) probabilities for linkage analyses we used SNPs with a call rate of at least 85%, HWE p-value > 0.05 and more informative markers with MAF > 5%. We iteratively identified SNP pairs with LD measure D' > 0.5, as estimated from HapMap data, and eliminated the SNP that was least informative for linkage (lowest MAF). We started with SNP pairs most closely located (physical distance) and continued until no pairs of SNPs had a D' measure exceeding 0.5. The final set of 10,592 SNPs combined with the 613 microsatellites were checked for excess recombination using MERLIN, Version 0.10.2 [68], and 4 SNPs and 1 microsatellite were omitted from linkage analyses based on a high number of possible errors, leaving a total of 11,200 markers to perform linkage analysis (10,588 SNPs + 612 short tandem repeats).

Variance component linkage analyses were performed on residuals of up to 1341 individuals in 310 full pedigrees. Four of the 1345 subjects were the only person in a pedigree and were excluded from linkage analyses since they contributed no information. Multipoint probabilities of IBD between relative pairs were computed at each genetic marker location with the program MERLIN, Version 0.10.2. Due to size limitations for exact identity by descent (IBD) multipoint computation in MERLIN software [68], the 310 full pedigrees were broken into 356 smaller pedigrees. The hypothesis of "no linkage at a specific genomic location" was tested by comparing models incorporating an effect of a putative quantitative trait locus (QTL) in complete linkage to the genetic marker, in the form of multipoint IBD sharing probabilities at the locus, to models incorporating only polygenic effects without a QTL effect. At each genetic location, a LOD score was computed as the logarithm to base 10 of the likelihood ratio of the locus-specific model to the polygenic model using the program SOLAR, Version 3.0.4 [69]. Allele frequencies were estimated by simple allele counts. In Framingham family data, which were collected from randomly sampled pedigrees, we have found the allele frequency estimates by simple allele counting closely match those calculated by maximum likelihood methods accounting for familial correlations.

#### Association testing

We applied population-based and family-based methods to test for association between the 100K SNPs and residual phenotypes using an additive model unless otherwise specified. We used family-based association test methods, implemented in the program FBAT, Version 1.5.5 [70,71], to test for differences in probability of transmission of a genotype from parents to offspring based on phenotype, as a test of linkage and association. FBAT has limited power because it requires association within families, and many families are non-informative. However, because FBAT examines association only within families, the type-I error rate is not affected by population stratification bias)[72,73]. We did not report results if the number of informative families was fewer than 10.

For the population-based approach, we used generalized estimating equation (GEE) [74] regression models to test for association between the 100K SNPs and each residual phenotype while taking into account the correlation among related individuals. We implemented the GEE approach by breaking families into sibships and used an exchangeable working correlation matrix to account for correlation within each sibship. Parental correlations with their children were not considered in these analyses. The analyses were performed using the gee program package, Version 4.13-10 [75] in R [65]. The GEE association test is a population-based approach that uses all individuals with both genotype and phenotype, regardless of genotype configuration within a family. Therefore, it is expected to be a powerful test of association if population stratification bias is not believed to be an issue, as in the FHS [76].

## Results

### Participant characteristics

Of the 1345 subjects who satisfied appropriate familial relationships and who were considered in the presentation of results in these manuscripts, 258 were Original Cohort participants (90 men and 168 women) and 1087 were Offspring Cohort participants (527 men and 560 women). Table 2 of the Overview presents descriptive information on these participants at enrollment (examination one). The Offspring and Cohort participants included on the family plates had lower mean age than other examination 1 participants, as these subjects needed to survive to the mid 1990s to provide DNA. We note that we used residuals based upon all subjects, as opposed to only those who were genotyped. Thus, the phenotypes reflect deviations of these subjects based on regressions for the full sample of subjects and are thus representative of the full sample.

**Table 2:** Description of Framingham Heart Study Subjects in 100K Genome-Wide Scan. Baseline Data at Exam 1 for Original (1948–1951) and Offspring (1971–1975) Cohorts

	Original Cohort Men	Original Cohort Women	Offspring Cohort Men	Offspring Cohort Women
	N = 90	N = 168	N = 527	N = 560
Age, years	35 ± 4	35 ± 4	31 ± 10	32 ± 10
(Limits)	(30–46)	(29–48)	(11–62)	(5–59)
Body Mass Index, kg/m^2^	25.7 ± 3.2	23.9 ± 3.6	26.0 ± 3.9	23.6 ± 4.3
Obese (BMI 30 ≥kg/m^2^), %	7.8	6.0	13.3	8.2
Systolic Blood Pressure, mm Hg	129 ± 13	120 ± 12	124 ± 14	115 ± 14
Diastolic Blood Pressure, mm Hg	81 ± 9	76 ± 8	81 ± 10	75 ± 10
Antihypertensive Medication, %	0	0	1.7	1.4
Hypertension, %	22.2	8.9	15.0	6.8
Current Smoking, %	61.1	42.3	40.0	41.3
Blood Glucose, mg/dL	78 ± 13	78 ± 11	102 ± 10	97 ± 9
Prevalent Diabetes, %	0	0	0.6	0.4
Total Cholesterol, mg/dL	220 ± 44	194 ± 37	192 ± 37	185 ± 36
HDL Cholesterol, mg/dL	N/A	N/A	44.9 ± 11.0	56.4 ± 13.9
Lipid Lowering Medication, %	0	0	0.2	0.5
Hemoglobin, g/dL	14.3 ± 1.2	12.3 ± 1.1	15.5 ± 1.0	13.5 ± 1.0
Forced Vital Capacity, dL	40 ± 7	28 ± 6	45 ± 8	32 ± 5
Prevalent CVD, %	0	0	5.5	2.6

Abbreviations: BMI = body mass index; HDL = high density lipoprotein; CVD = cardiovascular disease.

### Format of the FHS 100K manuscripts

We present 17 manuscripts, each displaying selected results for an epidemiologically related group of traits. Table 1 of the Overview presents the title and first author of each manuscript. The web resource displaying genetic association and linkage results is available at the NCBI dbGaP website, http://www.ncbi.nlm.nih.gov/projects/gap/cgi-bin/study.cgi?id=phs000007. Each manuscript describes the traits that were studied and presents some results. These manuscripts are not intended to be comprehensive and generally do not include results for all phenotypes and covariate adjustment schemes that were studied and presented on the website. Full listings of all traits evaluated are provided in Additional file 1 (phenotypes for population-based GEE analyses), Additional file 2 (phenotypes for family-based FBAT analyses) and Additional file 3 (phenotypes for linkage analyses), including url links to the corresponding analytical results on the NCBI dbGaP website. To facilitate the reading of these manuscripts, we have used a common format for all manuscripts. Table 1 of each manuscript presents a general description of the phenotypes that were evaluated. Table 2 of each manuscript displays the top results (lowest p-values) from GEE analyses, the top results (lowest p-values) from FBAT analyses and linkage results where the LOD score was 2 or more. Whereas top association results are based solely on p-value rank, the Working Groups also applied various additional strategies to identify SNPs that the group would prioritize to pursue further. For Table 3 of each manuscript, the groups devised schema to summarize results for related traits, grouping phenotypic traits within biologically plausible domains, or traits examined longitudinally. Each manuscript provides a description of the strategy employed and the results for its Table 3. Finally, Table 4 in each manuscript lists some SNPs that are the same as or correlated with genetic variants in genes that have been reported in the literature to be associated with the manuscript's phenotypes and indicates whether our results replicate those reports. Physical locations of the SNPs are provided according to NCBI Build35, whereas the dbGaP website uses a more recent version. Thus, the physical locations reported in the manuscripts may differ from those on the website. Each manuscript provides criteria for choosing which results were reported.

**Table 3:** Proportion of p-values falling below nominal levels for selected traits

SNPs with MAF ≥ 10%, HWE p ≥ 0.001 and Genotyping Call Rate ≥ 80%									
		**Nominal alpha level**							

	# SNPs	0.05	0.01	0.001	1 × 10^-4^	1 × 10^-5^	1 × 10^-6^	1 × 10^-7^	1 × 10^-8^

Metabolic Traits*									
Mean^† ^FBAT	70987	0.050	0.010	8.5 × 10^-4^	7.3 × 10^-5^	5.1 × 10^-6^	6.1 × 10^-7^	1.4 × 10^-7^	0
Min^† ^FBAT		0.046	0.008	4.4 × 10^-4^	0	0	0	0	0
Max^† ^FBAT		0.056	0.012	1.5 × 10^-3^	2.7 × 10^-4^	5.6 × 10^-5^	4.2 × 10^-5^	2.8 × 10^-5^	0
Mean GEE	70987	0.058	0.013	1.6 × 10^-3^	2.3 × 10^-4^	3.7 × 10^-5^	7.9 × 10^-6^	1.9 × 10^-6^	1.0 × 10^-7^
Min GEE		0.051	0.010	9.2 × 10^-4^	5.6 × 10^-5^	0	0	0	0
Max GEE		0.081	0.022	3.3 × 10^-3^	6.6 × 10^-4^	1.4 × 10^-4^	8.5 × 10^-5^	5.6 × 10^-5^	1.4 × 10^-5^

CVD Events*									
Mean FBAT	70987	0.050	0.009	6.6 × 10^-4^	4.4 × 10^-5^	4.0 × 10^-6^	0	0	0
Min FBAT		0.047	0.006	1.7 × 10^-4^	0	0	0	0	0
Max FBAT		0.053	0.010	9.4 × 10^-4^	8.5 × 10^-5^	1.4 × 10^-5^	0	0	0
Mean GEE	70987	0.054	0.012	1.4 × 10^-3^	1.8 × 10^-4^	2.4 × 10^-5^	6.7 × 10^-6^	2.7 × 10^-6^	6.7 × 10^-7^
Min GEE		0.046	0.009	7.9 × 10^-4^	4.2 × 10^-5^	0	0	0	0
Max GEE		0.062	0.015	2.5 × 10^-3^	5.9 × 10^-4^	1.3 × 10^-4^	5.6 × 10^-5^	2.8 × 10^-5^	1.4 × 10^-5^

All SNPs posted on website http://www.ncbi.nlm.nih.gov/projects/gap/cgi-bin/study.cgi?id=phs000007									

Metabolic Traits*									
Mean FBAT	100584	0.050	0.009	7.7 × 10^-4^	6.3 × 10^-5^	4.3 × 10^-6^	5.7 × 10^-7^	1.2 × 10^-7^	0
Min FBAT		0.046	0.008	4.4 × 10^-4^	0	0	0	0	0
Max FBAT		0.054	0.011	1.3 × 10^-3^	2.2 × 10^-4^	5.0 × 10^-5^	3.0 × 10^-5^	2.0 × 10^-5^	0
Mean GEE	101944	0.061	0.015	2.4 × 10^-3^	5.5 × 10^-4^	1.9 × 10^-4^	8.4 × 10^-5^	4.3 × 10^-5^	2.5 × 10^-5^
Min GEE		0.052	0.012	1.5 × 10^-3^	1.9 × 10^-4^	3.0 × 10^-5^	0	0	0
Max GEE		0.082	0.025	1.1 × 10^-2^	6.8 × 10^-3^	4.3 × 10^-3^	2.6 × 10^-3^	1.7 × 10^-3^	9.4 × 10^-4^

CVD Events*									
Mean FBAT	101060	0.048	0.008	5.9 × 10^-4^	3.5 × 10^-5^	3.3 × 10^-6^	0	0	0
Min FBAT		0.043	0.005	1.2 × 10^-4^	0	0	0	0	0
Max FBAT		0.052	0.010	9.1 × 10^-4^	9.9 × 10^-5^	1.0 × 10^-5^	0	0	0
Mean GEE	103194	0.059	0.016	3.4 × 10^-3^	1.1 × 10^-3^	4.7 × 10^-4^	2.0 × 10^-4^	9.5 × 10^-5^	4.4 × 10^-5^
Min GEE		0.050	0.011	1.1 × 10^-3^	1.3 × 10^-4^	2.9 × 10^-5^	0	0	0
Max GEE		0.078	0.033	1.4 × 10^-2^	7.1 × 10^-3^	3.6 × 10^-3^	1.6 × 10^-3^	7.8 × 10^-4^	3.3 × 10^-4^

* There were 415 Metabolic Traits and 14 CVD Events from which these descriptive statistics were calculated using the # SNPs indicated. # SNPs = average number of SNPs across all traits in the Trait Group; ^†^Mean = average proportion of SNPs below nominal level across phenotypes in the trait group; Min = minimum proportion below nominal level across phenotypes in the trait group; Max = maximum proportion below nominal level across phenotypes in the trait group.

**Table 4:** Power of the population-based association approach (GEE test) for a SNP with MAF = 0.1

			Nominal Type I Error							
SNP QTL Heritability	Effect Size (SD)*	Model	0.05	0.01	0.001	10^-4^	10^-5^	10^-6^	10^-7^	10^-8^

**100% phenotype available (n = 1345)**										
1%	0.24	GEE	0.977	0.919	0.78	0.60	0.43	0.27	0.17	0.10
2%	0.33	GEE	1	1.00	0.99	0.97	0.91	0.84	0.72	0.59
3%	0.41	GEE	1	1	1	0.998	0.992	0.985	0.957	0.918
4%	0.47	GEE	1	1	1	1	1	1	0.996	0.991
5%	0.53	GEE	1	1	1	1	1	1	1	1

**~80% subjects have phenotype(~20% missing at random): Sample size = 1076**										
1%	0.24	GEE	0.934	0.836	0.631	0.427	0.261	0.149	0.074	0.041
2%	0.33	GEE	1.00	0.99	0.97	0.93	0.82	0.66	0.51	0.37
3%	0.41	GEE	1	1	0.998	0.993	0.976	0.938	0.85	0.753
4%	0.47	GEE	0.998	0.998	0.998	0.998	0.991	0.984	0.971	0.949
5%	0.53	GEE	1	1	1	1	1	1	1	0.99

**~60% subjects have phenotype(~40% missing at random): Sample size = 783**										
1%	0.24	GEE	0.879	0.737	0.48	0.281	0.137	0.055	0.023	0.008
2%	0.33	GEE	0.992	0.96	0.88	0.744	0.591	0.433	0.293	0.186
3%	0.41	GEE	1	1	0.982	0.945	0.874	0.754	0.602	0.467
4%	0.47	GEE	1	1	1	0.998	0.981	0.95	0.884	0.797
5%	0.53	GEE	1	1	0.999	0.997	0.996	0.982	0.964	0.931

*SD: Standard Deviation. The effect size is expressed in unit of standard deviation of the phenotype.

### SNP allele frequencies and distribution

**Figure 1:**
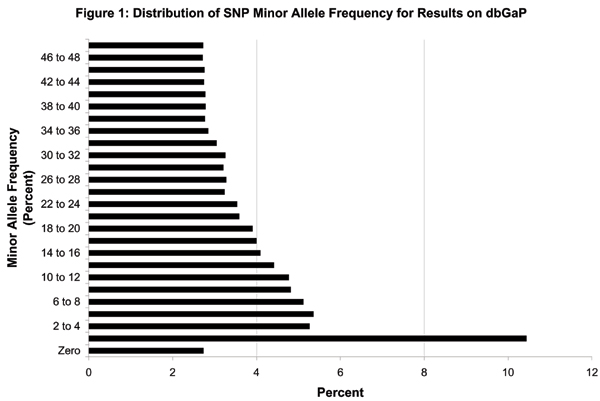
**Distribution of Minor Allele Frequency for SNPs in Framingham Sample displayed on dbGaP Website**. The percentage of SNPs (X axis) with MAF of zero and in ranges (0,2], (2,4], ..., (48,50] percent (Y axis) in the Framingham sample of 1345 subjects is detailed. For example, approximately 2.5–3% of SNPs in the Affymetrix 100K GeneChip had MAF of zero in the Framingham sample and about 4% had MAF greater than 16 percent and less than or equal to 18 percent. This distribution represents SNPs described on the dbGaP website. The manuscripts only include SNPs with MAF of 10% or more.

**Figure 2:**
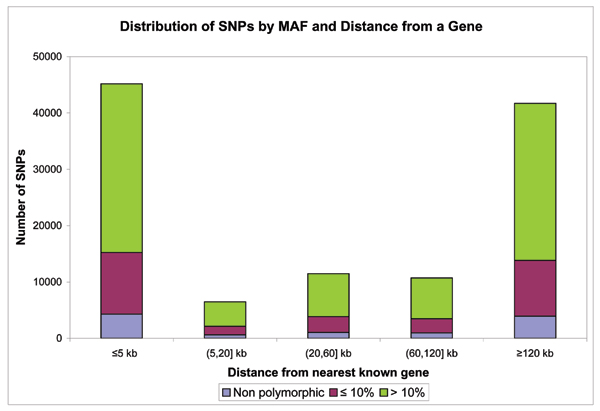
**Distribution of Affymetrix 100K GeneChip SNPs by distance from known genes**. The X axis is the distance from known genes and the Y axis is the number of SNPs according to each distance. Blue represents monomorphic SNPs, maroon is for SNPs with MAF < 10% and green is for SNPs with MAF ≥ 10%.

Allele frequencies for the 100K Affymetrix GeneChip in the Framingham sample are displayed in Figure 1. About 38% have MAF < 10% and are not considered in the series of manuscripts, although they are included on the dbGaP website. Among SNPs with MAF ≥ 10%, there were large numbers between 10–25% and were somewhat evenly spread over the range from 25–50% MAF. Many SNPs on the Affymetrix Chip are not near genes (Figure 2). About 30,000 with MAF ≥ 10% are within 5 kb of a gene; another 10,000 with MAF < 10% are within 5 kb. The remaining SNPs are further away from known genes.

### P-value distribution

The results displayed on the NCBI dbGaP http://www.ncbi.nlm.nih.gov/projects/gap/cgi-bin/study.cgi?id=phs000007 include all autosomal SNPs, regardless of genotyping call rate, HWE p-value or MAF; adjustable filters for these factors are provided. In our manuscripts, we present results for SNPs that satisfy genotyping call rates ≥ 80%, HWE p-value ≥ 0.001, and MAF ≥ 10%. The proportion of SNPs satisfying a genotyping call rate of ≥80% is 97.4%; 91% of SNPs satisfied a call rate of ≥90%.

We expect only a small percentage of all tested SNPs to be truly associated with any phenotype. Therefore, to obtain an approximation of the null distribution of p-values, we examined the distribution of p-values for 415 phenotypes from the Metabolic Working Group and 14 CVD event phenotypes. If one assumes that only a few true associations exist for each phenotype, these p-value distributions approximate the null distribution, because only a few SNPs out of the large total number tested would be expected to exceed any critical value due to true associations. Table 3 of the Overview displays the proportion of p-values among all SNPs below specific nominal alpha levels, summarized (mean, minimum and maximum) across all phenotypes in the trait group for GEE and FBAT results.

Many of the phenotypes in the Metabolic Working Group were approximately normally distributed (about 90% had absolute value of skewness <1 and about 80% had absolute value of kurtosis <2) and thus may reflect the situation for which the assumptions of the analytical methods were generally satisfied. We display two sets of results in Table 3 of the Overview for these phenotypes, those used in the publication of the manuscripts with the number of SNPs equal to 70,987 and the larger set of results displayed on the website with the number of SNPs equal to ~100–103 K. The difference in the number of SNPs evaluated for GEE and FBAT results arises from those SNPs that are uninformative for FBAT analyses (those with sufficiently rare minor allele so that fewer than 10 nuclear families were informative for transmission). The p-value distributions suggest that FBAT p-values generally follow the expected null distribution, assuming that nearly all results are false positives, and may actually be somewhat conservative. In contrast, the GEE p-values exhibit an excess of small p-values, especially for smaller nominal alpha levels. For example, for SNPs reported in the manuscripts, the average proportion of SNPs for a phenotype with p-value below specified alpha levels ranged from 1.3 to 19 times greater than the nominal level (1.3 times larger for nominal alpha of 0.01, 19 for nominal alpha of 10^-7 ^and 10 for 10^-8^). The excess is higher for the full set of SNPs reported on the website. Here we found that the average proportion ranged from 1.2 times larger for nominal level of 0.05 to 19 times greater for nominal level of 10^-5 ^and 2500 times greater for nominal level 10^-8^.

The CVD phenotypes represent an extreme case, as the phenotypes were residuals from survival models, were generally bimodal, and do not satisfy general assumptions for normality. We see the same general pattern that we observed for the Metabolic Working Group phenotypes with somewhat conservative FBAT tests and excess numbers of small p-values for GEE tests. As might be expected, the CVD phenotypes revealed larger excesses of small GEE p-values, with the average proportion of SNPs falling below specified alpha levels ranging from 1.08 (5.4% for nominal 5% with SNPs reported in the manuscripts) to 2.4 times greater for nominal level of 10^-5 ^and 67 times greater for nominal 10^-8 ^than expected. Thus, GEE p-values need to be interpreted with care, as SNPs in the lowest p-value range have the potential to be especially enriched with false positives.

We also examined the dependence of the p-value distribution for GEE results on the genotyping call rate for the Metabolic Working Group phenotypes. Our sample was not ascertained on trait status; so genotyping failures were likely to be randomly distributed. Therefore, one might expect that the effect of genotyping error on type I error would be more modest than for case-control studies)[77]. As expected, we continued to find an excess of small p-values, despite increasingly stringent call rate thresholds. More importantly, we found that this excess occurred regardless of call rate. For example, for nominal alpha of 0.001 and genotyping call rate > 95%, we found that the ratio of the number of observed to expected significant results ranged from 1.6 for MAF in the range of (0.2, 0.5) to 7.0 for MAF in the range (0, 0.05). Similarly, for call rate less than 80% we found similar ratios of 1.6 to 8.1, respectively. For nominal alpha of 10^-6 ^we found this ratio varied from 9.5 to 614 for call rate > 95% and 6.8 to 667 for call rate < 80%. Thus, we used a liberal genotyping call rate of > 80% for presentation of results in our manuscripts to err on the side of including a result rather than not, even though we expect nearly all results to be false positives.

**Figure 3:**
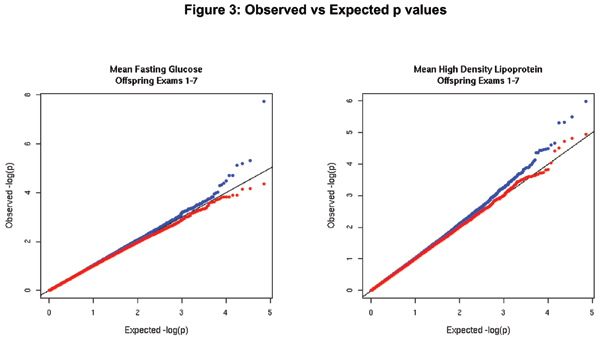
**Observed versus Expected p-values (-log base 10 scale) for Mean Fasting Glucose in Offspring Exams 1 to 7 (Left) and for Mean Fasting High Density Lipoprotein in Offspring Exams 1 to 7 (Right)**. Blue dots are for GEE and red dots for FBAT.

Figure 3 displays observed GEE (blue) and FBAT (red) p-values versus expected p-values (straight line) on a negative logarithm scale for mean fasting plasma glucose and mean high density lipoprotein cholesterol, calculated from Offspring measurements over exams 1 to 7. We see that FBAT p-values tend to be less significant than expected (conservative) whereas GEE p-values tend to be more significant than expected (liberal), especially for smaller expected p-values. While we would expect most p-values to fall on the line if there were no genetic associations, p-values that reflect true associations will be more extreme (smaller) than expected. In looking at the figure for mean fasting glucose, SNPs represented by the blue dots (GEE) far above the expected line on the right hand side of the figure may represent true associations with mean fasting plasma glucose. The plot for mean fasting HDL cholesterol also suggests that there may be some true positives, as even a few FBAT p-values are more extreme than expected.

As in any GWAS, we expect that most results with small p-values are false positives. The p-value distributions support this notion and further suggest that the GEE results may have more false positives than one would expect. Table 2 in each manuscript ranks results by p-value, but each paper also pursues its own strategy to identify which results may be more worthy of follow up in Table 3 of each manuscript, usually by considering evidence from several sources such as correlated traits.

### Power estimations for population-based association approach

To assess the power of the population-based association approach with GEE, we simulated a trait following a normal distribution with 30% polygenic heritability in this sample of 1345 subjects. We generated a SNP with MAF 0.10 and assumed that the SNP was the QTL with an additive effect and QTL heritability varying from 1% to 5%. We also varied the proportion of phenotyped individuals from 60% to 100%, as some traits were not available in all subjects genotyped. The phenotype and genotype data were simulated using SOLAR simqtl, Version 3.0.4. We tested the association between the SNP and the trait using GEE. One thousand replicates were performed for each scenario. The results are displayed in Table 4 of the Overview. For a conservative alpha level such as 10^-8^, we have more than 80% power to detect a SNP explaining 4% or more total phenotypic variation when 60% or more individuals are phenotyped. With higher MAF, the power remains similar for the same QTL heritability (data not shown). Thus, we have sufficient power to detect SNPs explaining ≥4% or more of the phenotypic variance using the population-based GEE association test approach, controlling for multiple testing for a single trait. The effect size for a specific QTL heritability, defined as the increase/decrease of the phenotype value with one copy increment of the allele tested, depends on the MAF of the SNP. For example, for a SNP explaining 4% of the phenotypic variation, the effect size is 0.47 times the phenotypic standard deviation (SD) for a MAF of 0.1, and 0.28 SD for a MAF of 0.5.

### Synthetic strategies

Beyond simple examination of individual p-values for single tests, the authors of each manuscript developed their own synthetic strategies to prioritize SNPs that may be worthy of follow up (results shown in Table 3 of each manuscript). Many strategies considered results among similar traits. For example, the Subclinical Working Group created four subgroups of traits: (1) ankle brachial index phenotypes, (2) common carotid IMT phenotypes, (3) internal carotid IMT phenotypes, and (4) multidetector computed tomography (MDCT) coronary calcification phenotypes [29]. Within each trait group, SNPs were ranked according to the proportion of traits with p < 0.01 for both FBAT and GEE in the group. Table 3 in the Subclinical manuscript displays the top 5 ranked SNPs with highest proportions of significant traits and lowest mean GEE p-values for each trait group [29]. The Lipids Subgroup of the Metabolic Working Group decided to focus on four phenotypes measured in Offspring subjects: apolipoprotein A-I levels measured at exam 4, small low-density lipoprotein as measured by nuclear magnetic resonance, mean high-density lipoprotein cholesterol levels over 7 exams, and mean log triglyceride levels over 7 exams [39]. Presenting only those results where at least 3 of the 4 traits had GEE p-value < 0.01, the SNPs were ranked according to geometric mean of these four traits [39]. The manuscript presenting results for neurological traits focused on specific phenotypes within subgroups with p < 0.001 in FBAT or GEE results and also other traits within the subgroup with p < 0.01 [37].

### Some results of interest

The main results for each Working Group are presented in the individual manuscripts of this series. Here we highlight some results that address some of our expectations.

#### Overlap of linkage and association results

Whereas strategies for genetic studies have been undergoing substantial changes in recent years, partly due to changes in the laboratory, we hypothesized that genomic regions that harbor significant linkage results would also contain significant association results. One example of the concordance of linkage and association results was noted for monocyte chemoattractant protein-1 (MCP1), an inflammatory marker, where a region of linkage (LOD = 4.96 at chromosome 1, 159 Mb) for MCP1 concentrations also contains two SNPs on chromosome 1 within the 1.5 support interval for the linkage peak and within 60 kb of the genes *OR10J1 *and *FCER1A *or *OR10J3 *(rs4128725 and rs2494250 with p-values in the 10^-8 ^range by FBAT, ≤10^-12 ^by GEE) [33]. We found a similar result for C-reactive protein with a LOD score of 3.28 on chromosome 1 and two significant SNPs (rs2794520 (p = 2.83*10^-8^) and rs2808629 (p = 3.19*10^-8^)) within the 1.5 support interval of this peak [33]. Whereas Lp(a) had the highest LOD score (LOD = 23.0) at 159.4 Mb, a SNP (rs1591375) located at 160.7 Mb near this peak had somewhat modest p-values by comparison (4.37*10^-06 ^by GEE and 0.0045 by FBAT) [39]. However, there were many instances for which there was no evidence of linkage in the setting of significant association. Additionally, each manuscript describes linkage results that are in accord with previously published linkage results.

#### SNPs overlapping across phenotypes

We did not expect the same SNPs to appear in many manuscripts, as cardiovascular disease is complex and involves a large and varied number of pathways for its development. In contrast, some manuscripts report on correlated traits. Thus, we examined overlap among the top 500 SNPs associated with the phenotypes across 3 Metabolic Working Groups: glycemic/diabetes phenotypes, lipid phenotypes and obesity phenotypes. Of 11 SNPs found in more than one group, none were found among the top 500 SNPs in all three groups. However, 7 SNPs were found in the glycemia and obesity groups, 2 in glycemia and lipid groups and 2 in lipid and obesity groups [38-40].

#### Replication of prior associations

In Table 4 of each manuscript, we investigated whether our results replicated previous reports in the literature. The 100K chip does not contain many SNPs in well-known lipid genes, such as *APOE*. On the other hand, we found that SNP rs7007797 in the *LPL *gene was associated with both HDL and triglycerides [39]; we replicated recent findings of association of a SNP in the TCF7L2 gene with diabetes [38]. Strong statistical support was found for the association of factor VII concentrations with SNP rs561241 on chromosome 13 (4*10^-16^) [34], which resides near the factor VII gene and is in complete linkage disequilibrium (r^2 ^= 1) with the Arg/Gln FVII SNP previously shown to account for 9% of the total phenotypic variance [78]. Similarly, we found associations of circulating levels of C-reactive protein with a SNP in the gene encoding C-reactive protein [33]. Two SNPs in *SORL 1*, a gene recently related to the risk of Alzheimer's disease [79], were found to be associated with performance on tests of abstract reasoning (rs1131497; FBAT p = 3.2 × 10^-6^; rs726601; FBAT p = 8.2 × 10^-4^) [37]. We found that SNPs (rs2543600 and rs27225364) near the *WRN *gene that causes premature aging are associated with age at death and morbidity-free survival at age 65 years [35]. The LD between these SNPs and those previously reported in the WRN gene is unknown as the previously reported SNPs are not in the HapMap. We found that SNP rs2478518 in the *AGT *gene was associated with both systolic and diastolic blood pressure [28]. The association of common variation at the *NOS1AP *locus with electrocardiographic QT interval duration was replicated with p-values ranging from 0.0001–0.0009 for 4 partially correlated SNPs [27]. In contrast, a number of results reported in the literature were not replicated in our results. For example, we did not find significant SNPs in *ACE *associated with blood pressure [28]. *PPARG *P12A (rs1801282) was not associated with diabetes or related traits including body mass index [38]. Some of these 'negative' results may be due to low power or low LD between a SNP reported in the literature and SNPs on the 100K chip.

We have also identified a few results that are biologically compelling, although replication of the SNP association is warranted. For example, we found that a SNP (rs1158167) near the *CST3 *gene was highly associated with serum cystatin-C levels (GEE p-value = 8.5*10^-09^) [42]. This SNP explained 2.5% of the variation in serum cystatin-C levels in our data and has been previously reported to be associated with cystatin-C. These results are presented in more detail in the Renal Endocrine Working Group manuscript [42].

#### Replication of results from other genome-wide studies

While we have been preparing this series of manuscripts, several genome-wide studies have been published [80-86]. Some results in our analyses support results reported in these recent studies. For example, we find significant associations for coronary heart disease, cardiovascular disease and coronary artery calcium [29,30] in the same chromosomal region on 9p recently reported to be associated with myocardial infarction by Helgadottir *et al*. [82] and McPherson *et al*. [83]. While our results need to be compared more closely with results being reported by other genome-wide studies, this example provides evidence that our results replicate strong associations from other genome-wide studies.

## Discussion

We have presented a brief description of the methods and a few selected results derived from analyses of the 100K Affymetrix GeneChip with a large number of FHS traits, ranging from CVD events and subclinical measures to traditional cardiovascular risk factors of diabetes, lipid levels, blood pressure and also including more novel biomarker measures that reflect modern hypotheses, such as the role of inflammatory pathways in the development of CVD. We have also reported on a number of neurological, renal, cancer and aging traits, including longevity (age at death) and bone mass and structure. None of these manuscripts provide a comprehensive report. Rather, the purpose of this set of manuscripts is to provide a brief summary of the results and to introduce readers to the data posted on the dbGaP website http://www.ncbi.nlm.nih.gov/projects/gap/cgi-bin/study.cgi?id=phs000007. We note that the genotypes in this sample have also been evaluated by Drs. Michael Christman and Alan Herbert. Some of their results are reported on line as described by Herbert *et al*. [87].

Several aspects of our investigation merit comment. **First**, the present investigation represents a comprehensive GWAS analysis of numerous phenotypes in a large community-based cohort. To our knowledge, it is the largest GWAS performed in an observational cohort in terms of the number of phenotypes analyzed and web posted. **Second**, we exploited the phenotypic diversity and richness of the Framingham Offspring Study database to analyze a set of phenotypes that were for the most part collected by detailed, direct measurements of study participants. Further, many of the phenotypes are quantitative traits. Phenotypes have been broadly categorized into seventeen different domains for manuscripts in this supplement. It is noteworthy that key risk factor phenotypes, such as blood pressure and lipid levels, were collected at multiple examinations, and thus we were able to conduct analyses using time-averaged traits, maximizing the scientific yield from the longitudinal prospective design of our cohort study. Further, several recently collected phenotypes, in particular biomarkers and imaging measures, were collected using highly reproducible, state-of-the-art modalities. Correlated phenotypes facilitated the assessment of pleiotropy by seeking associations of SNPs with such phenotypes. These investigations occurred primarily among the variables in each individual manuscript. Finally, for most phenotypes, there was evidence for a significant heritable component from FHS or other studies. We acknowledge that some phenotypic domains may represent analytical constructs, rather than truly distinct groups from a biological standpoint.

**Third**, we have web-posted the results of all analyses on autosomes on dbGaP, including results without statistical evidence of association, so that investigators world-wide can access the data freely and mine them *in silico *for hypothesis generation, inclusion in meta-analysis, and direct comparisons with their own results. In addition to the freely posted aggregate results, participant-specific genotypic and phenotypic data are available for distribution for further analyses to approved scientific investigators world-wide via the NCBI/NHLBI and consistent with Framingham Study data distribution policies (see http://www.nhlbi.nih.gov/about/framingham/policies/index.htm). For the purpose of publication, reference to these analyses may be made by referring to either the appropriate manuscript or the specific URL for web-posted data. **Fourth**, the simultaneous and full-disclosure release (on the web) of all association and linkage results of phenotypes encompassing at least 17 different domains in a cohesive and comprehensive manner signifies the tremendous teamwork of numerous FHS investigators, statisticians, programmers, and others. Most importantly, this effort would not be possible without the full cooperation and commitment of the FHS participants, who continue to attend Study examinations in an effort to further the scientific knowledge of factors that lead to heart disease and other traits.

**Fifth**, as in any genome-wide association study with a large number of SNPs, most results that are considered statistically significant by a conventional p < 0.05 may be falsely positive; so it is difficult to decide what results are important. Not only do we have a large number of statistical tests for each phenotype, but we also have numerous phenotypes. Thus, considering multiple testing in the interpretation of results is of paramount importance. There are several approaches to address the issue of multiple testing, such as Bonferroni correction, permutation testing and false discovery rates. To conduct permutation testing for all of the traits that we considered is prohibitively time-consuming, particularly in preserving heritability of the traits with family data. Further, with correlated traits it is difficult to decide what traits should be included in a permutation testing strategy. One approach to controlling the false-positive rate in genome-wide association studies is to set a stringent threshold for declaring statistical significance. According to the report of the International HapMap Consortium, complete testing of common variants (MAF > 0.05) in each 500 kb is equivalent to performing 150 independent tests in white populations of European descent [88]. Using this guide and given that there are about 3000 Mb in the human genome, we would estimate that there are approximately 900,000–1,000,000 independent tests if testing all common variants in the genome. A conservative Bonferroni correction using this number of tests (0.05/1,000,000) yields an approximate threshold of genome-wide significance to be 5*10^-8^. Thus, for a single trait, one could use this threshold. Several results do fall below this threshold (Table 5 of the Overview). In considering these results, we note that our sample size of 1345 biologically related subjects is relatively small for detecting genetic variants of modest effect. We also note that we have a large number of correlated traits, including the same traits with different covariate adjustments. Further, we have already observed that our GEE results have an excess number of small p-values. Thus, we are hesitant to regard any result reported in our manuscripts as significant at a genome-wide level. We believe these findings are best regarded as hypothesis-generating. The determination of what constitutes genome-wide significance is challenged both by theoretical considerations as well as practical ones. Without pursuing more computationally intensive analyses, it is thus difficult to provide specific advice regarding what SNPs are most important. **It may be safer to assume that most of the small p-values are likely to be false positives and that replication of our results in other independent samples is of critical importance**. We proceed with presentation of full-disclosure results to encourage readers to pursue such studies.

**Table 5:** Associations achieving nominal genome wide significance, p < 5*10^-8 ^across the 17 phenotype working groups

Phenotype working group/manuscript	Trait	SNP rs ID*	Chr	Physical location (bp)	GEE P-value	FBAT P-value	IN/NEAR gene
Select biomarkers [33]	Monocyte chemoattractant protein-1	rs2494250	1	156,091,324	1.0*10^-14^	3.5*10^-8^	*FCER1A, OR10J3*
	Monocyte chemoattractant protein-1	rs4128725	1	156,219,032	3.7*10^-12^	3.3*10^-8^	*OR10J1*
	C-reactive protein average exams 2,6,7	rs2794520	1	156,491,889	2.8*10^-8^	4.3*10^-5^	*CRP*
	C-reactive protein average exams 2,6,7	rs2808629	1	156,489,869	3.2*10^-8^	4.8*10^-5^	*CRP*
Kidney/Endocrine [42]	Cystatin C	rs1158167	20	23,526,189	8.5*10^-09^	0.006	*CST9L|CST9|CST3*
Diabetes [38]	28-year mean fasting plasma glucose	rs2722425	8	40,603,396	2.0*10^-8^	0.005	*ZMAT4*
Sleep and circadian [26]	Epworth sleepiness scale	rs1823068	5	58,711,806	2.5*10^-8^	0.069	*PDE4D*
Neurology [37]	Total Cerebral Brain Volume (ATCBV)	rs1970546	20	59287333	4.0*10^-8^	0.005	*CDH4*
Hemostatic factors [34]	Factor VII	rs561241	13	112,808,035	4.5*10^-16^	3.4*10^-4^	*F7*

The following phenotype working groups did not have any traits achieving nominal genome-wide significance: echocardiography, flow-mediated dilation and exercise tolerance testing; blood pressure and tonometry; subclinical cardiovascular disease; cardiovascular outcomes; cancer; electrocardiography and heart rate variability; pulmonary function testing; aging; bone; lipids; obesity

**Sixth**, we note that use of the 80% genotyping call rate is unusually liberal by today's standards in GWAS. We used this threshold in these manuscripts to be inclusive, rather than exclusive, in a first look such as this. We recognize that this threshold may permit consideration of some results that could be spurious due problems with genotyping. However, a limitation of our genotypes is that the genotype calls were made with the DM algorithm, which is less precise than those that have recently been introduced. At this time, we are unable to apply more accurate, reliable genotyping calls [89], as we do not have access to the source data. Further, we found that the choice of the 80% threshold versus a more conservative one had little effect upon p-value distributions. Finally, all results, regardless of genotyping call rate, are posted on the dbGaP website and thus, investigators can evaluate for themselves what they believe to be the more valid results from this study.

**Seventh**, in our analyses we found that the GEE results appear to have an excess of significant results. We suspect that one reason is low MAF. Also, given the small sample of at most 1345 subjects, we would expect only 13–14 individuals to have the minor homozygote. Thus, we limited the results that we present in the manuscripts to those SNPs with MAF = 10%. Further analyses have indicated that use of a linear mixed effects model such as incorporating a SNP as a covariate in a regression model with proper correlation structure for the error terms that fully represent the familial correlations remedies this problem and has a valid type I error rate in simulated data.

**Eighth**, coverage of LD is incomplete with the 100K scan. Nicolae *et al*. report that the Affymetrix 100K GeneChip includes fewer SNPs in coding and more SNPs in intergenic regions than represented on the HapMap [90]. Further, our sample size is modest. These two facts combined likely limit the power for detection of associations with several traits in these data. For instance, while we noted modest to high heritability of numerous phenotypes, underscoring the contribution of additive genetic effects to interindividual variation in these traits, we did not find significant low p-values for several heritable traits in relation to the SNPs evaluated. Factors contributing to this observation included both the limited coverage of the Affymetrix 100K GeneChip as well as the possibility that some of the less significant p-values (example between 0.05 and 10^-5^) may represent true positive findings. The limited power to detect SNPs of small effect sizes offered by the analysis of our relatively modest sample size of ~1300 participants contributes to this phenomenon as well; we only have high power to detect a SNP explaining 4% or more of the phenotypic variance in the population-based GEE association test; the power of FBAT and variance component linkage analysis is even lower.

Additionally, for several of the analyzed phenotypes we did not observe any overlap between the top SNP-phenotype associations noted in GEE and FBAT analyses. The inherent differences in the two analytical methods especially in the context of the modest sample sizes, particularly for FBAT with small numbers of informative trios, may contribute to this phenomenon. FBAT is limited by the number of informative transmissions and although we suspect that there is little population stratification in our sample [76], GEE is limited by potential bias due to stratification. Furthermore, for several phenotypes the SNPs associated with the top LOD scores in linkage analyses were not among the top 50 SNPs in association analyses (GEE or FBAT).

**Ninth**, we were limited in our ability to replicate genetic variants previously reported to be associated with phenotypes in our database because specific coverage of such genetic variation in these candidates was limited in the Affymetrix 100K GeneChip. We view such analyses as more illustrative of the potential utility of our GWAS, rather than as definitive evidence for or against an association described with a putative candidate gene in the published literature.

Our data do suggest several interesting biological candidates among the SNPs most strongly associated with different traits in the various analytical approaches. The strongest and most clear-cut of the associations were for those phenotypes that represent the direct protein product of a gene. Examples include the association of CRP concentrations with SNPs in the *CRP *gene (Benjamin *et al*. in this series [33]) and factor VII levels with SNP rs561241 on chromosome 13 (Yang *et al*. in this series [34]). Thus, while it is difficult to point to any result as definitive, those results for which we find some evidence of replication of associations found in the literature are regarded as worthy of further research.

**Finally**, the Framingham Study participants were white of European descent and predominantly middle-aged to elderly. Hence, the genetic associations may not be generalizable to other ethnicities/races or to younger individuals.

## Conclusion

In summary, the results from the FHS 100K association and linkage studies described herein and posted on the NCBI website provide a GWAS resource for investigators. We have presented a description of the methods and general strategies used for analysis of the 100K Affymetrix GeneChip in relation to a broad range of traits measured in the FHS. Brief descriptions of results of these analyses are provided a series of 17 manuscripts, with results for all autosomal SNPs genotyped successfully displayed at http://www.ncbi.nlm.nih.gov/projects/gap/cgi-bin/study.cgi?id=phs000007. Interested investigators can also access the data through a standing protocol, described at http://www.nhlbi.nih.gov/about/framingham/policies/index.htm. Key to interpretation of these results is replication and evaluation of these results in other cohorts and ultimately, functional studies. We encourage investigators to examine the results and to pursue the genetic signals therein in their own cohorts. In the near future we will provide results and data from approximately 550,000 SNPs on more than 9000 participants from three generations in the FHS SNP Health Association Resource (SHARe) project. Data will be available to qualified investigators through an application process to dbGaP. It is our hope that the results from these two genome-wide association studies will lead to a much deeper understanding of the role of common genetic variation in the development of cardiovascular disease and its risk factors.

## Abbreviations

FBAT = family-based association test; GEE = generalized estimating equations; GWAS = Genome wide association study; IBD = identity by descent; LD = linkage disequilibrium; LOD = logarithm of the odds; SNP = single nucleotide polymorphism.

## Competing interests

Dr. Meigs currently has research grants from GlaxoSmithKline and Wyeth, and serves on safety or advisory boards for GlaxoSmithKline, Merck, and Lilly.

## Authors' contributions

All authors have made substantial contributions to conception and design, or acquisition of data, or analysis and interpretation of data; LAC, EJB, SD, ALD, JD, KF, CSF, DJG, SK, DPK, MGL, DL, KLL, MDM, JBM, CNC, GTO, CJO, SS, RSV, JBW, QY and LDA have been involved in drafting the manuscript or revising it critically for important intellectual content; and All authors have given final approval of the version to be published.

In addition, MDM, ZYW: imported, performed QC, processed, organized, and displayed all results within the NCBI dbGaP.

## Supplementary Material

Additional file 1 Phenotypes for population-based GEE analyses. Click here for file

Additional file 2 Phenotypes for family-based FBAT analyses. Click here for file

Additional file 3 Phenotypes for linkage analyses. Click here for file
