# Supplementary material for: The Framingham Heart Study 100K SNP genome-wide association study resource: overview of 17 phenotype working group reports
Source: BMC Med Genet. 2007 Sep 19;8(Suppl 1):S1. doi: 10.1186/1471-2350-8-S1-S1 (PMC1995613; doi:10.1186/1471-2350-8-S1-S1)
Supplement: Additional file 1 — Phenotypes for population-based GEE analyses. [file 1471-2350-8-S1-S1-S1.pdf]

Group	Category	Trait Label	Name	GEE Link
Aging	BoneAging	BUA	Bone Ultrasound attenuation measured by QUS, multivariable adjusted	http://www.ncbi.nlm.nih.gov/projects/gap/cgi-bin/analysis.cgi?id=pha001754
Aging	BoneAging	deltaOSSR	Bone age by osseographic scoring system, multivariable adjusted	http://www.ncbi.nlm.nih.gov/projects/gap/cgi-bin/analysis.cgi?id=pha001788
Aging	BoneAging	deltaOSSrf	Bone age by osseographic scoring system (Women), multivariable adjusted	http://www.ncbi.nlm.nih.gov/projects/gap/cgi-bin/analysis.cgi?id=pha001790
Aging	BoneAging	deltaOSSrm	Bone age by osseographic scoring system (Men, multivariable adjusted)	http://www.ncbi.nlm.nih.gov/projects/gap/cgi-bin/analysis.cgi?id=pha001792
Aging	BoneAging	FNBMD	Femoral Neck BMD measured by DXA, multivariable adjusted	http://www.ncbi.nlm.nih.gov/projects/gap/cgi-bin/analysis.cgi?id=pha001756
Aging	BoneAging	FNBMDf	Femoral Neck BMD measured by DXA (Women), multivariable adjusted	http://www.ncbi.nlm.nih.gov/projects/gap/cgi-bin/analysis.cgi?id=pha001794
Aging	BoneAging	FNBMDm	Femoral Neck BMD measured by DXA (Men), multivariable adjusted	http://www.ncbi.nlm.nih.gov/projects/gap/cgi-bin/analysis.cgi?id=pha001796
Aging	BoneAging	ITAvgBR1rf	Hip geometry (IT BucklingRatio, Women), multivariable adjusted	http://www.ncbi.nlm.nih.gov/projects/gap/cgi-bin/analysis.cgi?id=pha001798
Aging	BoneAging	ITAvgBR1rm	Hip geometry (IT BucklingRatio, Men), multivariable adjusted	http://www.ncbi.nlm.nih.gov/projects/gap/cgi-bin/analysis.cgi?id=pha001800
Aging	BoneAging	ITAvgBRrf	Hip geometry (IT BucklingRatio, Women), age- adjusted	http://www.ncbi.nlm.nih.gov/projects/gap/cgi-bin/analysis.cgi?id=pha001802
Aging	BoneAging	ITAvgBRrm	Hip geometry (IT BucklingRatio, Men), age- adjusted	http://www.ncbi.nlm.nih.gov/projects/gap/cgi-bin/analysis.cgi?id=pha001804
Aging	BoneAging	LSBMD	Lumbar Spine BMD measured by DXA, multivariable adjusted	http://www.ncbi.nlm.nih.gov/projects/gap/cgi-bin/analysis.cgi?id=pha001758
Aging	BoneAging	LSBMDf	Spine Neck BMD measured by DXA (Women), multivariable adjusted	http://www.ncbi.nlm.nih.gov/projects/gap/cgi-bin/analysis.cgi?id=pha001818
Aging	BoneAging	LSBMDm	Spine Neck BMD measured by DXA (Men), multivariable adjusted	http://www.ncbi.nlm.nih.gov/projects/gap/cgi-bin/analysis.cgi?id=pha001820
Aging	BoneAging	NeckCSMI	Hip geometry by DXA (Neck Moment of Inertia), age- and sex-adjusted	http://www.ncbi.nlm.nih.gov/projects/gap/cgi-bin/analysis.cgi?id=pha001760
Aging	BoneAging	NeckCSMI1	Hip geometry by DXA (Neck Moment of Inertia), multivariable adjusted	http://www.ncbi.nlm.nih.gov/projects/gap/cgi-bin/analysis.cgi?id=pha001762
Aging	BoneAging	NeckLeng	Hip geometry by DXA (Neck Length), age- and sex-adjusted	http://www.ncbi.nlm.nih.gov/projects/gap/cgi-bin/analysis.cgi?id=pha001764
Aging	BoneAging	NeckLeng1	Hip geometry by DXA (Neck Length), multivariable adjusted	http://www.ncbi.nlm.nih.gov/projects/gap/cgi-bin/analysis.cgi?id=pha001766
Aging	BoneAging	NeckLengf	Hip geometry (Neck Length, Women), multivariable adjusted	http://www.ncbi.nlm.nih.gov/projects/gap/cgi-bin/analysis.cgi?id=pha001822
Aging	BoneAging	NeckLengm	Hip geometry (Neck Length, Men), multivariable adjusted	http://www.ncbi.nlm.nih.gov/projects/gap/cgi-bin/analysis.cgi?id=pha001824
Aging	BoneAging	NeckW	Hip geometry by DXA (Neck Width), age- and sex-adjusted	http://www.ncbi.nlm.nih.gov/projects/gap/cgi-bin/analysis.cgi?id=pha001768
Aging	BoneAging	NeckW1	Hip geometry by DXA (Neck Width), multivariable adjusted	http://www.ncbi.nlm.nih.gov/projects/gap/cgi-bin/analysis.cgi?id=pha001770
Aging	BoneAging	NeckW1rf	Hip geometry (Neck Width, Women), multivariable adjusted	http://www.ncbi.nlm.nih.gov/projects/gap/cgi-bin/analysis.cgi?id=pha001826
Aging	BoneAging	NeckW1rm	Hip geometry (Neck Width, Men), multivariable adjusted	http://www.ncbi.nlm.nih.gov/projects/gap/cgi-bin/analysis.cgi?id=pha001828
Aging	BoneAging	NeckWrf	Hip geometry (Neck Width, Women), age-adjusted	http://www.ncbi.nlm.nih.gov/projects/gap/cgi-bin/analysis.cgi?id=pha001830
Aging	BoneAging	NeckWrm	Hip geometry (Neck Width, Men), age-adjusted	http://www.ncbi.nlm.nih.gov/projects/gap/cgi-bin/analysis.cgi?id=pha001832
Aging	BoneAging	NeckZ	Hip geometry by DXA (Neck Section Modulus), age- and sex-adjusted	http://www.ncbi.nlm.nih.gov/projects/gap/cgi-bin/analysis.cgi?id=pha001772
Aging	BoneAging	NeckZ1	Hip geometry by DXA (Neck Section Modulus), multivariable adjusted	http://www.ncbi.nlm.nih.gov/projects/gap/cgi-bin/analysis.cgi?id=pha001774
Aging	BoneAging	NeckZ1rf	Hip geometry (Neck Section Modulus, Women), multivariable adjusted	http://www.ncbi.nlm.nih.gov/projects/gap/cgi-bin/analysis.cgi?id=pha001834
Aging	BoneAging	NeckZ1rm	Hip geometry (Neck Section Modulus, Men), multivariable adjusted	http://www.ncbi.nlm.nih.gov/projects/gap/cgi-bin/analysis.cgi?id=pha001836
Aging	BoneAging	NeckZrf	Hip geometry (Neck Section Modulus, Women), age-adjusted	http://www.ncbi.nlm.nih.gov/projects/gap/cgi-bin/analysis.cgi?id=pha001838
Aging	BoneAging	NeckZrm	Hip geometry (Neck Section Modulus, Men), age-adjusted	http://www.ncbi.nlm.nih.gov/projects/gap/cgi-bin/analysis.cgi?id=pha001840
Aging	BoneAging	NNAvgBR1rf	Hip geometry (NN BucklingRatio, Women), multivariable adjusted	http://www.ncbi.nlm.nih.gov/projects/gap/cgi-bin/analysis.cgi?id=pha001842
Aging	BoneAging	NNAvgBR1rm	Hip geometry (NN BucklingRatio, Men), multivariable adjusted	http://www.ncbi.nlm.nih.gov/projects/gap/cgi-bin/analysis.cgi?id=pha001844
Aging	BoneAging	NNAvgBRrf	Hip geometry (NN BucklingRatio, Women), age-adjusted	http://www.ncbi.nlm.nih.gov/projects/gap/cgi-bin/analysis.cgi?id=pha001846
Aging	BoneAging	NNAvgBRrm	Hip geometry (NN BucklingRatio, Men), age-adjusted	http://www.ncbi.nlm.nih.gov/projects/gap/cgi-bin/analysis.cgi?id=pha001848
Aging	BoneAging	NNCSMI1rf	Hip geometry (Neck CSMI, Women), multivariable adjusted	http://www.ncbi.nlm.nih.gov/projects/gap/cgi-bin/analysis.cgi?id=pha001850
Aging	BoneAging	NNCSMI1rm	Hip geometry (Neck CSMI, Men), multivariable adjusted	http://www.ncbi.nlm.nih.gov/projects/gap/cgi-bin/analysis.cgi?id=pha001852
Aging	BoneAging	NNCSMIrf	Hip geometry (Neck CSMI, Women), age-adjusted	http://www.ncbi.nlm.nih.gov/projects/gap/cgi-bin/analysis.cgi?id=pha001854
Aging	BoneAging	NNCSMIrm	Hip geometry (Neck CSMI, Men), age-adjusted	http://www.ncbi.nlm.nih.gov/projects/gap/cgi-bin/analysis.cgi?id=pha001856
Aging	BoneAging	NSA	Hip geometry by DXA (Neck-Shaft Angle), age- and sex-adjusted	http://www.ncbi.nlm.nih.gov/projects/gap/cgi-bin/analysis.cgi?id=pha001776
Aging	BoneAging	NSA1	Hip geometry by DXA (Neck-Shaft Angle), multivariable adjusted	http://www.ncbi.nlm.nih.gov/projects/gap/cgi-bin/analysis.cgi?id=pha001778
Aging	BoneAging	NSAf	Hip geometry (Neck-Shaft Angle, Women), multivariable adjusted	http://www.ncbi.nlm.nih.gov/projects/gap/cgi-bin/analysis.cgi?id=pha001858
Aging	BoneAging	NSAm	Hip geometry (Neck-Shaft Angle, Men), multivariable adjusted	http://www.ncbi.nlm.nih.gov/projects/gap/cgi-bin/analysis.cgi?id=pha001860
Aging	BoneAging	ShaftAvgBRr1f	Hip geometry (Shaft BucklingRatio, Women), multivariable adjusted	http://www.ncbi.nlm.nih.gov/projects/gap/cgi-bin/analysis.cgi?id=pha001862
Aging	BoneAging	ShaftAvgBRr1m	Hip geometry (Shaft BucklingRatio, Men), multivariable adjusted	http://www.ncbi.nlm.nih.gov/projects/gap/cgi-bin/analysis.cgi?id=pha001864
Aging	BoneAging	ShaftAvgBRrf	Hip geometry (Shaft BucklingRatio, Women), age-adjusted	http://www.ncbi.nlm.nih.gov/projects/gap/cgi-bin/analysis.cgi?id=pha001866
Aging	BoneAging	ShaftAvgBRrm	Hip geometry (Shaft BucklingRatio, Men), age-adjusted	http://www.ncbi.nlm.nih.gov/projects/gap/cgi-bin/analysis.cgi?id=pha001868
Aging	BoneAging	ShaftCSMI1R	Hip geometry (Shaft CSMI), multivariable adjusted	http://www.ncbi.nlm.nih.gov/projects/gap/cgi-bin/analysis.cgi?id=pha001870
Aging	BoneAging	ShaftCSMIrf	Hip geometry (Shaft CSMI, Women), multivariable adjusted	http://www.ncbi.nlm.nih.gov/projects/gap/cgi-bin/analysis.cgi?id=pha001872
Aging	BoneAging	ShaftCSMIrm	Hip geometry (Shaft CSMI, Men), multivariable adjusted	http://www.ncbi.nlm.nih.gov/projects/gap/cgi-bin/analysis.cgi?id=pha001874
Aging	BoneAging	ShaftW	Hip geometry by DXA (Shaft Width), age- and sex-adjusted	http://www.ncbi.nlm.nih.gov/projects/gap/cgi-bin/analysis.cgi?id=pha001780
Aging	BoneAging	ShaftW1	Hip geometry by DXA (Shaft Width), multivariable adjusted	http://www.ncbi.nlm.nih.gov/projects/gap/cgi-bin/analysis.cgi?id=pha001782
Aging	BoneAging	ShaftW1f	Hip geometry (Shaft Width, Women), multivariable adjusted	http://www.ncbi.nlm.nih.gov/projects/gap/cgi-bin/analysis.cgi?id=pha001876
Aging	BoneAging	ShaftW1m	Hip geometry (Shaft Width, Men), multivariable adjusted	http://www.ncbi.nlm.nih.gov/projects/gap/cgi-bin/analysis.cgi?id=pha001878

Group	Category	Trait Label	Name	GEE Link
Aging	BoneAging	ShaftWf	Hip geometry (Shaft Width, Women), age-adjusted	http://www.ncbi.nlm.nih.gov/projects/gap/cgi-bin/analysis.cgi?id=pha001880
Aging	BoneAging	ShaftWm	Hip geometry (Shaft Width, Men), age-adjusted	http://www.ncbi.nlm.nih.gov/projects/gap/cgi-bin/analysis.cgi?id=pha001882
Aging	BoneAging	ShaftZ1R	Hip geometry (Shaft Section Modulus), multivariable adjusted	http://www.ncbi.nlm.nih.gov/projects/gap/cgi-bin/analysis.cgi?id=pha001884
Aging	BoneAging	ShaftZ1rf	Hip geometry (Shaft Section Modulus, Women), multivariable adjusted	http://www.ncbi.nlm.nih.gov/projects/gap/cgi-bin/analysis.cgi?id=pha001886
Aging	BoneAging	ShaftZ1rm	Hip geometry (Shaft Section Modulus, Men), multivariable adjusted	http://www.ncbi.nlm.nih.gov/projects/gap/cgi-bin/analysis.cgi?id=pha001888
Aging	BoneAging	SOS	Bone Ultrasound speed measured by QUS, multivariable adjusted	http://www.ncbi.nlm.nih.gov/projects/gap/cgi-bin/analysis.cgi?id=pha001784
Aging	BoneAging	TRBMD	Trochanter BMD measured by DXA, multivariable adjusted	http://www.ncbi.nlm.nih.gov/projects/gap/cgi-bin/analysis.cgi?id=pha001786
Aging	BoneAging	TRBMDf	Troch Neck BMD measured by DXA (Women), multivariable adjusted	http://www.ncbi.nlm.nih.gov/projects/gap/cgi-bin/analysis.cgi?id=pha001890
Aging	BoneAging	TRBMDm	Troch Neck BMD measured by DXA (Men), multivariable adjusted	http://www.ncbi.nlm.nih.gov/projects/gap/cgi-bin/analysis.cgi?id=pha001892
Aging	BrainMRI	AFBV	Frontal lobe to intracranial volume ratio, multivariable adjusted	http://www.ncbi.nlm.nih.gov/projects/gap/cgi-bin/analysis.cgi?id=pha001894
Aging	BrainMRI	AHPV	Hippocampal to intracranial volume ratio, multivariable adjusted	http://www.ncbi.nlm.nih.gov/projects/gap/cgi-bin/analysis.cgi?id=pha001896
Aging	BrainMRI	AHPVapoe1	Hippocampal to intracranial volume ratio, multivariable with APOE	http://www.ncbi.nlm.nih.gov/projects/gap/cgi-bin/analysis.cgi?id=pha001930
Aging	BrainMRI	AHPVr	Hippocampal to total cerebral volume ratio, multivariable adjusted	http://www.ncbi.nlm.nih.gov/projects/gap/cgi-bin/analysis.cgi?id=pha001898
Aging	BrainMRI	AHPVrapoe1	Hippocampal to total cerebral volume ratio, multivariable with APOE	http://www.ncbi.nlm.nih.gov/projects/gap/cgi-bin/analysis.cgi?id=pha001932
Aging	BrainMRI	ALLV	Log lateral ventricular to intracranial volume ratio, multivariable adjusted	http://www.ncbi.nlm.nih.gov/projects/gap/cgi-bin/analysis.cgi?id=pha001900
Aging	BrainMRI	ALTHBV	Log temporal horn to intracranial volume ratio, multivariable adjusted	http://www.ncbi.nlm.nih.gov/projects/gap/cgi-bin/analysis.cgi?id=pha001902
Aging	BrainMRI	ALWMHIVapoe1	Log white matter hyperintensity to cranial ratio, multivariable with APOE	http://www.ncbi.nlm.nih.gov/projects/gap/cgi-bin/analysis.cgi?id=pha001934
Aging	BrainMRI	AOBV	Occipital lobe to intracranial volume ratio, multivariable adjusted	http://www.ncbi.nlm.nih.gov/projects/gap/cgi-bin/analysis.cgi?id=pha001904
Aging	BrainMRI	APBV	Parietal lobe to intracranial volume ratio, multivariable adjusted	http://www.ncbi.nlm.nih.gov/projects/gap/cgi-bin/analysis.cgi?id=pha001906
Aging	BrainMRI	ATBV	Temporal lobe to intracranial volume ratio, multivariable adjusted	http://www.ncbi.nlm.nih.gov/projects/gap/cgi-bin/analysis.cgi?id=pha001908
Aging	BrainMRI	ATCBV	Total cerebral brain to intracranial volume ratio, multivariable adjusted	http://www.ncbi.nlm.nih.gov/projects/gap/cgi-bin/analysis.cgi?id=pha001910
Aging	BrainMRI	ATCBVapoe1	Total cerebral brain to intracranial volume ratio, multivariable with APOE	http://www.ncbi.nlm.nih.gov/projects/gap/cgi-bin/analysis.cgi?id=pha001936
Aging	BrainMRI	CFBV	Frontal lobe to intracranial volume ratio, age and sex adjusted	http://www.ncbi.nlm.nih.gov/projects/gap/cgi-bin/analysis.cgi?id=pha001912
Aging	BrainMRI	CHPV	Hippocampal to intracranial volume ratio, age and sex adjusted	http://www.ncbi.nlm.nih.gov/projects/gap/cgi-bin/analysis.cgi?id=pha001914
Aging	BrainMRI	CHPVr	Hippocampal to total cerebral volume ratio, age and sex adjusted	http://www.ncbi.nlm.nih.gov/projects/gap/cgi-bin/analysis.cgi?id=pha001916
Aging	BrainMRI	CLLV	Log lateral ventricular to intracranial volume ratio, age and sex adjusted	http://www.ncbi.nlm.nih.gov/projects/gap/cgi-bin/analysis.cgi?id=pha001918
Aging	BrainMRI	CLTHBV	Log temporal horn to intracranial volume ratio, age and sex adjusted	http://www.ncbi.nlm.nih.gov/projects/gap/cgi-bin/analysis.cgi?id=pha001920
Aging	BrainMRI	COBV	Occipital lobe to intracranial volume ratio, age and sex adjusted	http://www.ncbi.nlm.nih.gov/projects/gap/cgi-bin/analysis.cgi?id=pha001922
Aging	BrainMRI	CPBV	Parietal lobe to intracranial volume ratio, age and sex adjusted	http://www.ncbi.nlm.nih.gov/projects/gap/cgi-bin/analysis.cgi?id=pha001924
Aging	BrainMRI	CTBV	Temporal lobe to intracranial volume ratio, age and sex adjusted	http://www.ncbi.nlm.nih.gov/projects/gap/cgi-bin/analysis.cgi?id=pha001926
Aging	BrainMRI	CTCBV	Total cerebral brain to intracranial volume ratio, age and sex adjusted	http://www.ncbi.nlm.nih.gov/projects/gap/cgi-bin/analysis.cgi?id=pha001928
Aging	CognitiveFunction	F1	Visual memory composite score, multivariable adjusted	http://www.ncbi.nlm.nih.gov/projects/gap/cgi-bin/analysis.cgi?id=pha001938
Aging	CognitiveFunction	F2	Visuospatial memory & organization, multivariable adjusted	http://www.ncbi.nlm.nih.gov/projects/gap/cgi-bin/analysis.cgi?id=pha001940
Aging	CognitiveFunction	F3	Visual scanning & motor speed, multivariable adjusted	http://www.ncbi.nlm.nih.gov/projects/gap/cgi-bin/analysis.cgi?id=pha001942
Aging	CognitiveFunction	MMSE5MVX	MMSE score, offspring exam 5, multivariable- adjusted	http://www.ncbi.nlm.nih.gov/projects/gap/cgi-bin/analysis.cgi?id=pha001950
Aging	CognitiveFunction	MMSE5to7MVX	average MMSE score, offspring exams 5 & 7, multivariable-adjusted	http://www.ncbi.nlm.nih.gov/projects/gap/cgi-bin/analysis.cgi?id=pha001952
Aging	CognitiveFunction	MMSE5to7X	average MMSE score, offspring exam 5 & 7, age-adjusted	http://www.ncbi.nlm.nih.gov/projects/gap/cgi-bin/analysis.cgi?id=pha001954
Aging	CognitiveFunction	MMSE5X	MMSE score, offspring exam 5, age-adjusted	http://www.ncbi.nlm.nih.gov/projects/gap/cgi-bin/analysis.cgi?id=pha001956
Aging	CognitiveFunction	MMSE65MVX	MMSE score at age 65, offspring & cohort pooled, multivariable-adjusted	http://www.ncbi.nlm.nih.gov/projects/gap/cgi-bin/analysis.cgi?id=pha001958
Aging	CognitiveFunction	MMSE65X	MMSE score at age 65, offspring & cohort pooled, birth cohort-adjusted	http://www.ncbi.nlm.nih.gov/projects/gap/cgi-bin/analysis.cgi?id=pha001960
Aging	CognitiveFunction	MMSE7MV	MMSE score, offspring exam 7, multivariable- adjusted	http://www.ncbi.nlm.nih.gov/projects/gap/cgi-bin/analysis.cgi?id=pha002837
Aging	CognitiveFunction	MMSE7X	MMSE score, offspring exam 7, age-adjusted	http://www.ncbi.nlm.nih.gov/projects/gap/cgi-bin/analysis.cgi?id=pha001962
Aging	CognitiveFunction	Nam	Boston Naming Test score without cues, multivariable adjusted	http://www.ncbi.nlm.nih.gov/projects/gap/cgi-bin/analysis.cgi?id=pha001944
Aging	CognitiveFunction	Sim	Similarities raw score, multivariable adjusted	http://www.ncbi.nlm.nih.gov/projects/gap/cgi-bin/analysis.cgi?id=pha001946
Aging	CognitiveFunction	WRAT	Wide Range Achievement Test, multivariable adjusted	http://www.ncbi.nlm.nih.gov/projects/gap/cgi-bin/analysis.cgi?id=pha001948
Aging	Hearing	SPTA	Pure tone audiometry average over medium frequencies, age and sex adjusted	http://www.ncbi.nlm.nih.gov/projects/gap/cgi-bin/analysis.cgi?id=pha001964
Aging	Hearing	SPTAHI	Pure tone audiometry average over high frequencies, age and sex adjusted	http://www.ncbi.nlm.nih.gov/projects/gap/cgi-bin/analysis.cgi?id=pha001966
Aging	Hearing	SPTALO	Pure tone audiometry average over low frequencies, age and sex adjusted	http://www.ncbi.nlm.nih.gov/projects/gap/cgi-bin/analysis.cgi?id=pha001968
Aging	Morbidity-freesurvival	Morbidityfree65MVX	morbidity-free survival at age 65, offspring & cohort pooled, multivariable-adjusted	http://www.ncbi.nlm.nih.gov/projects/gap/cgi-bin/analysis.cgi?id=pha001970
Aging	Morbidity-freesurvival	Morbidityfree65X	morbidity-free survival at age 65, offspring & cohort pooled, birth cohort- adjusted	http://www.ncbi.nlm.nih.gov/projects/gap/cgi-bin/analysis.cgi?id=pha001972
Aging	PhysicalDisability	Handgrip727X	hand grip, offspring exam 7 & cohort exam 27	http://www.ncbi.nlm.nih.gov/projects/gap/cgi-bin/analysis.cgi?id=pha001978
Aging	PhysicalDisability	Handgrip7X	hand grip, offspring exam 7	http://www.ncbi.nlm.nih.gov/projects/gap/cgi-bin/analysis.cgi?id=pha001980
Aging	PhysicalDisability	Walkingspeed727X	walking speed, offspring exam 7 & cohort exam 27	http://www.ncbi.nlm.nih.gov/projects/gap/cgi-bin/analysis.cgi?id=pha001982
Aging	PhysicalDisability	Walkingspeed7X	walking speed, offspring exam 7	http://www.ncbi.nlm.nih.gov/projects/gap/cgi-bin/analysis.cgi?id=pha001984
Aging	ReproductiveTraits	MenoageMVX	age at natural menopause, adjusted	http://www.ncbi.nlm.nih.gov/projects/gap/cgi-bin/analysis.cgi?id=pha001986

Group	Category	Trait Label	Name	GEE Link
Aging	ReproductiveTraits	MenoageX	age at natural menopause, crude	http://www.ncbi.nlm.nih.gov/projects/gap/cgi-bin/analysis.cgi?id=pha001988
Aging	Survival	DeathageMV	age at death, offspring & cohort pooled, multivariable-adjusted	http://www.ncbi.nlm.nih.gov/projects/gap/cgi-bin/analysis.cgi?id=pha001990
Aging	Survival	DeathageX	age at death, offspring & cohort pooled, adjusted for birth cohort	http://www.ncbi.nlm.nih.gov/projects/gap/cgi-bin/analysis.cgi?id=pha001992
Aging	Survival	DeathpastALEMV	death past average life expectancy, offspring & cohort pooled, multivariable-adjusted	http://www.ncbi.nlm.nih.gov/projects/gap/cgi-bin/analysis.cgi?id=pha001994
Aging	Survival	DeathpastALEX	death past average life expectancy, offspring & cohort pooled, birth cohort adjusted	http://www.ncbi.nlm.nih.gov/projects/gap/cgi-bin/analysis.cgi?id=pha001996
BloodPressure	Bloodpressure	DBP17AVGIMPAS	diastolic BP average age-sex-adjusted residual exams 1-7	http://www.ncbi.nlm.nih.gov/projects/gap/cgi-bin/analysis.cgi?id=pha001418
BloodPressure	Bloodpressure	DBP17AVGIMPMV	diastolic BP average multivariable-adjusted residual exams 1-7	http://www.ncbi.nlm.nih.gov/projects/gap/cgi-bin/analysis.cgi?id=pha001420
BloodPressure	Bloodpressure	DBP1IMPMEANAS	diastolic BP exam 1, age- sex- adjusted	http://www.ncbi.nlm.nih.gov/projects/gap/cgi-bin/analysis.cgi?id=pha001422
BloodPressure	Bloodpressure	DBP1IMPMEANMV	diastolic BP exam 1, multivariable-adjusted	http://www.ncbi.nlm.nih.gov/projects/gap/cgi-bin/analysis.cgi?id=pha001424
BloodPressure	Bloodpressure	DBP2IMPMEANAS	diastolic BP exam 2, age- sex- adjusted	http://www.ncbi.nlm.nih.gov/projects/gap/cgi-bin/analysis.cgi?id=pha001426
BloodPressure	Bloodpressure	DBP2IMPMEANMV	diastolic BP exam 2, multivariable-adjusted	http://www.ncbi.nlm.nih.gov/projects/gap/cgi-bin/analysis.cgi?id=pha001428
BloodPressure	Bloodpressure	DBP3IMPMEANAS	diastolic BP exam 3, age- sex- adjusted	http://www.ncbi.nlm.nih.gov/projects/gap/cgi-bin/analysis.cgi?id=pha001430
BloodPressure	Bloodpressure	DBP3IMPMEANMV	diastolic BP exam 3, multivariable-adjusted	http://www.ncbi.nlm.nih.gov/projects/gap/cgi-bin/analysis.cgi?id=pha001432
BloodPressure	Bloodpressure	DBP4IMPMEANAS	diastolic BP exam 4, age- sex- adjusted	http://www.ncbi.nlm.nih.gov/projects/gap/cgi-bin/analysis.cgi?id=pha001434
BloodPressure	Bloodpressure	DBP4IMPMEANMV	diastolic BP exam 4, multivariable-adjusted	http://www.ncbi.nlm.nih.gov/projects/gap/cgi-bin/analysis.cgi?id=pha001436
BloodPressure	Bloodpressure	DBP5IMPMEANAS	diastolic BP exam 5, age- sex- adjusted	http://www.ncbi.nlm.nih.gov/projects/gap/cgi-bin/analysis.cgi?id=pha001438
BloodPressure	Bloodpressure	DBP5IMPMEANMV	diastolic BP exam 5, multivariable-adjusted	http://www.ncbi.nlm.nih.gov/projects/gap/cgi-bin/analysis.cgi?id=pha001440
BloodPressure	Bloodpressure	DBP6IMPMEANAS	diastolic BP exam 6, age- sex- adjusted	http://www.ncbi.nlm.nih.gov/projects/gap/cgi-bin/analysis.cgi?id=pha001442
BloodPressure	Bloodpressure	DBP6IMPMEANMV	diastolic BP exam 6, multivariable-adjusted	http://www.ncbi.nlm.nih.gov/projects/gap/cgi-bin/analysis.cgi?id=pha001444
BloodPressure	Bloodpressure	DBP7IMPMEANAS	diastolic BP exam 7, age- sex- adjusted	http://www.ncbi.nlm.nih.gov/projects/gap/cgi-bin/analysis.cgi?id=pha001446
BloodPressure	Bloodpressure	DBP7IMPMEANMV	diastolic BP exam 7, multivariable-adjusted	http://www.ncbi.nlm.nih.gov/projects/gap/cgi-bin/analysis.cgi?id=pha001448
BloodPressure	Bloodpressure	PP17AVGIMPAS	pulse pressure average age-sex-adjusted residual exams 1-7	http://www.ncbi.nlm.nih.gov/projects/gap/cgi-bin/analysis.cgi?id=pha001450
BloodPressure	Bloodpressure	PP17AVGIMPMV	pulse pressure average multivariable-adjusted residual exams 1-7	http://www.ncbi.nlm.nih.gov/projects/gap/cgi-bin/analysis.cgi?id=pha001452
BloodPressure	Bloodpressure	PP1IMPMEANAS	pulse pressure exam 1, age- sex- adjusted	http://www.ncbi.nlm.nih.gov/projects/gap/cgi-bin/analysis.cgi?id=pha001454
BloodPressure	Bloodpressure	PP1IMPMEANMV	pulse pressure exam 1, multivariable- adjusted	http://www.ncbi.nlm.nih.gov/projects/gap/cgi-bin/analysis.cgi?id=pha001456
BloodPressure	Bloodpressure	PP2IMPMEANAS	pulse pressure exam 2, age- sex- adjusted	http://www.ncbi.nlm.nih.gov/projects/gap/cgi-bin/analysis.cgi?id=pha001458
BloodPressure	Bloodpressure	PP2IMPMEANMV	pulse pressure exam 2, multivariable- adjusted	http://www.ncbi.nlm.nih.gov/projects/gap/cgi-bin/analysis.cgi?id=pha001460
BloodPressure	Bloodpressure	PP3IMPMEANAS	pulse pressure exam 3, age- sex- adjusted	http://www.ncbi.nlm.nih.gov/projects/gap/cgi-bin/analysis.cgi?id=pha001462
BloodPressure	Bloodpressure	PP3IMPMEANMV	pulse pressure exam 3, multivariable- adjusted	http://www.ncbi.nlm.nih.gov/projects/gap/cgi-bin/analysis.cgi?id=pha001464
BloodPressure	Bloodpressure	PP4IMPMEANAS	pulse pressure exam 4, age- sex- adjusted	http://www.ncbi.nlm.nih.gov/projects/gap/cgi-bin/analysis.cgi?id=pha001466
BloodPressure	Bloodpressure	PP4IMPMEANMV	pulse pressure exam 4, multivariable- adjusted	http://www.ncbi.nlm.nih.gov/projects/gap/cgi-bin/analysis.cgi?id=pha001468
BloodPressure	Bloodpressure	PP5IMPMEANAS	pulse pressure exam 5, age- sex- adjusted	http://www.ncbi.nlm.nih.gov/projects/gap/cgi-bin/analysis.cgi?id=pha001470
BloodPressure	Bloodpressure	PP5IMPMEANMV	pulse pressure exam 5, multivariable- adjusted	http://www.ncbi.nlm.nih.gov/projects/gap/cgi-bin/analysis.cgi?id=pha001472
BloodPressure	Bloodpressure	PP6IMPMEANAS	pulse pressure exam 6, age- sex- adjusted	http://www.ncbi.nlm.nih.gov/projects/gap/cgi-bin/analysis.cgi?id=pha001474
BloodPressure	Bloodpressure	PP6IMPMEANMV	pulse pressure exam 6, multivariable- adjusted	http://www.ncbi.nlm.nih.gov/projects/gap/cgi-bin/analysis.cgi?id=pha001476
BloodPressure	Bloodpressure	PP7IMPMEANAS	pulse pressure exam 7, age- sex- adjusted	http://www.ncbi.nlm.nih.gov/projects/gap/cgi-bin/analysis.cgi?id=pha001478
BloodPressure	Bloodpressure	PP7IMPMEANMV	pulse pressure exam 7, multivariable- adjusted	http://www.ncbi.nlm.nih.gov/projects/gap/cgi-bin/analysis.cgi?id=pha001480
BloodPressure	Bloodpressure	SBP17AVGIMPAS	systolic BP average age-sex-adjusted residual exams 1-7	http://www.ncbi.nlm.nih.gov/projects/gap/cgi-bin/analysis.cgi?id=pha001482
BloodPressure	Bloodpressure	SBP17AVGIMPMV	systolic BP average multivariable-adjusted residual exams 1-7	http://www.ncbi.nlm.nih.gov/projects/gap/cgi-bin/analysis.cgi?id=pha001484
BloodPressure	Bloodpressure	SBP1IMPMEANAS	systolic BP exam 1, age- sex- adjusted	http://www.ncbi.nlm.nih.gov/projects/gap/cgi-bin/analysis.cgi?id=pha001486
BloodPressure	Bloodpressure	SBP1IMPMEANMV	systolic BP exam 1, multivariable-adjusted	http://www.ncbi.nlm.nih.gov/projects/gap/cgi-bin/analysis.cgi?id=pha001488
BloodPressure	Bloodpressure	SBP2IMPMEANAS	systolic BP exam 2, age- sex- adjusted	http://www.ncbi.nlm.nih.gov/projects/gap/cgi-bin/analysis.cgi?id=pha001490
BloodPressure	Bloodpressure	SBP2IMPMEANMV	systolic BP exam 2, multivariable-adjusted	http://www.ncbi.nlm.nih.gov/projects/gap/cgi-bin/analysis.cgi?id=pha001492
BloodPressure	Bloodpressure	SBP3IMPMEANAS	systolic BP exam 3, age- sex- adjusted	http://www.ncbi.nlm.nih.gov/projects/gap/cgi-bin/analysis.cgi?id=pha001494
BloodPressure	Bloodpressure	SBP3IMPMEANMV	systolic BP exam 3, multivariable-adjusted	http://www.ncbi.nlm.nih.gov/projects/gap/cgi-bin/analysis.cgi?id=pha001496
BloodPressure	Bloodpressure	SBP4IMPMEANAS	systolic BP exam 4, age- sex- adjusted	http://www.ncbi.nlm.nih.gov/projects/gap/cgi-bin/analysis.cgi?id=pha001498
BloodPressure	Bloodpressure	SBP4IMPMEANMV	systolic BP exam 4, multivariable-adjusted	http://www.ncbi.nlm.nih.gov/projects/gap/cgi-bin/analysis.cgi?id=pha001500
BloodPressure	Bloodpressure	SBP5IMPMEANAS	systolic BP exam 5, age- sex- adjusted	http://www.ncbi.nlm.nih.gov/projects/gap/cgi-bin/analysis.cgi?id=pha001502
BloodPressure	Bloodpressure	SBP5IMPMEANMV	systolic BP exam 5, multivariable-adjusted	http://www.ncbi.nlm.nih.gov/projects/gap/cgi-bin/analysis.cgi?id=pha001504
BloodPressure	Bloodpressure	SBP6IMPMEANAS	systolic BP exam 6, age- sex- adjusted	http://www.ncbi.nlm.nih.gov/projects/gap/cgi-bin/analysis.cgi?id=pha001506
BloodPressure	Bloodpressure	SBP6IMPMEANMV	systolic BP exam 6, multivariable-adjusted	http://www.ncbi.nlm.nih.gov/projects/gap/cgi-bin/analysis.cgi?id=pha001508
BloodPressure	Bloodpressure	SBP7IMPMEANAS	systolic BP exam 7, age- sex- adjusted	http://www.ncbi.nlm.nih.gov/projects/gap/cgi-bin/analysis.cgi?id=pha001510
BloodPressure	Bloodpressure	SBP7IMPMEANMV	systolic BP exam 7, multivariable-adjusted	http://www.ncbi.nlm.nih.gov/projects/gap/cgi-bin/analysis.cgi?id=pha001512
BloodPressure	echocardiography	AOR26AVGAS	M-mode Echo aortic root diastolic diameter, avg age-sex resid, exams 2,4,5,6	http://www.ncbi.nlm.nih.gov/projects/gap/cgi-bin/analysis.cgi?id=pha001514
BloodPressure	echocardiography	AOR26AVGMV	M-mode Echo aortic root diastolic diameter, avg multivar resid, exams 2,4,5,6	http://www.ncbi.nlm.nih.gov/projects/gap/cgi-bin/analysis.cgi?id=pha001516

Group	Category	Trait Label	Name	GEE Link
BloodPressure	echocardiography	AOR2AS	M-mode Echo aortic root diastolic diameter, exam 2, age-sex-adjusted	http://www.ncbi.nlm.nih.gov/projects/gap/cgi-bin/analysis.cgi?id=pha001518
BloodPressure	echocardiography	AOR2MV	M-mode Echo aortic root diastolic diameter, exam 2, multivariable-adjusted	http://www.ncbi.nlm.nih.gov/projects/gap/cgi-bin/analysis.cgi?id=pha001520
BloodPressure	echocardiography	AOR4AS	M-mode Echo aortic root diastolic diameter, exam 4, age-sex-adjusted	http://www.ncbi.nlm.nih.gov/projects/gap/cgi-bin/analysis.cgi?id=pha001522
BloodPressure	echocardiography	AOR4MV	M-mode Echo aortic root diastolic diameter, exam 4, multivariable-adjusted	http://www.ncbi.nlm.nih.gov/projects/gap/cgi-bin/analysis.cgi?id=pha001524
BloodPressure	echocardiography	AOR5AS	M-mode Echo aortic root diastolic diameter, exam 5, age-sex-adjusted	http://www.ncbi.nlm.nih.gov/projects/gap/cgi-bin/analysis.cgi?id=pha001526
BloodPressure	echocardiography	AOR5MV	M-mode Echo aortic root diastolic diameter, exam 5, multivariable-adjusted	http://www.ncbi.nlm.nih.gov/projects/gap/cgi-bin/analysis.cgi?id=pha001528
BloodPressure	echocardiography	AOR6AS	M-mode Echo aortic root diastolic diameter, exam 6, age-sex-adjusted	http://www.ncbi.nlm.nih.gov/projects/gap/cgi-bin/analysis.cgi?id=pha001530
BloodPressure	echocardiography	AOR6MV	M-mode Echo aortic root diastolic diameter, exam 6, multivariable-adjusted	http://www.ncbi.nlm.nih.gov/projects/gap/cgi-bin/analysis.cgi?id=pha001532
BloodPressure	echocardiography	LAD26AVGAS	M-mode Echo Left atrial systolic diameter, avg age-sex resid, exams 2,4,5,6	http://www.ncbi.nlm.nih.gov/projects/gap/cgi-bin/analysis.cgi?id=pha001534
BloodPressure	echocardiography	LAD26AVGMV	M-mode Echo Left atrial systolic diameter, avg multivar resid, exams 2,4,5,6	http://www.ncbi.nlm.nih.gov/projects/gap/cgi-bin/analysis.cgi?id=pha001536
BloodPressure	echocardiography	LAD2AS	M-mode Echo Left atrial systolic diameter, exam 2, age-sex-adjusted	http://www.ncbi.nlm.nih.gov/projects/gap/cgi-bin/analysis.cgi?id=pha001538
BloodPressure	echocardiography	LAD2MV	M-mode Echo Left atrial systolic diameter, exam 2, multivariable-adjusted	http://www.ncbi.nlm.nih.gov/projects/gap/cgi-bin/analysis.cgi?id=pha001540
BloodPressure	echocardiography	LAD4AS	M-mode Echo Left atrial systolic diameter, exam 4, age-sex-adjusted	http://www.ncbi.nlm.nih.gov/projects/gap/cgi-bin/analysis.cgi?id=pha001542
BloodPressure	echocardiography	LAD4MV	M-mode Echo Left atrial systolic diameter, exam 4, multivariable-adjusted	http://www.ncbi.nlm.nih.gov/projects/gap/cgi-bin/analysis.cgi?id=pha001544
BloodPressure	echocardiography	LAD5AS	M-mode Echo Left atrial systolic diameter, exam 5, age-sex-adjusted	http://www.ncbi.nlm.nih.gov/projects/gap/cgi-bin/analysis.cgi?id=pha001546
BloodPressure	echocardiography	LAD5MV	M-mode Echo Left atrial systolic diameter, exam 5, multivariable-adjusted	http://www.ncbi.nlm.nih.gov/projects/gap/cgi-bin/analysis.cgi?id=pha001548
BloodPressure	echocardiography	LAD6AS	M-mode Echo Left atrial systolic diameter, exam 6, age-sex-adjusted	http://www.ncbi.nlm.nih.gov/projects/gap/cgi-bin/analysis.cgi?id=pha001550
BloodPressure	echocardiography	LAD6MV	M-mode Echo Left atrial systolic diameter, exam 6, multivariable-adjusted	http://www.ncbi.nlm.nih.gov/projects/gap/cgi-bin/analysis.cgi?id=pha001552
BloodPressure	echocardiography	LVDD26AVGAS	M-mode Echo LV diastolic diameter, avg age-sex resid, exams 2,4,5,6	http://www.ncbi.nlm.nih.gov/projects/gap/cgi-bin/analysis.cgi?id=pha001554
BloodPressure	echocardiography	LVDD26AVGMV	M-mode Echo LV diastolic diameter, avg multivar resid, exams 2,4,5,6	http://www.ncbi.nlm.nih.gov/projects/gap/cgi-bin/analysis.cgi?id=pha001556
BloodPressure	echocardiography	LVDD2AS	M-mode Echo LV diastolic diameter, exam 2, age-sex-adjusted	http://www.ncbi.nlm.nih.gov/projects/gap/cgi-bin/analysis.cgi?id=pha001558
BloodPressure	echocardiography	LVDD2MV	M-mode Echo LV diastolic diameter, exam 2, multivariable-adjusted	http://www.ncbi.nlm.nih.gov/projects/gap/cgi-bin/analysis.cgi?id=pha001560
BloodPressure	echocardiography	LVDD4AS	M-mode Echo LV diastolic diameter, exam 4, age-sex-adjusted	http://www.ncbi.nlm.nih.gov/projects/gap/cgi-bin/analysis.cgi?id=pha001562
BloodPressure	echocardiography	LVDD4MV	M-mode Echo LV diastolic diameter, exam 4, multivariable-adjusted	http://www.ncbi.nlm.nih.gov/projects/gap/cgi-bin/analysis.cgi?id=pha001564
BloodPressure	echocardiography	LVDD5AS	M-mode Echo LV diastolic diameter, exam 5, age-sex-adjusted	http://www.ncbi.nlm.nih.gov/projects/gap/cgi-bin/analysis.cgi?id=pha001566
BloodPressure	echocardiography	LVDD5MV	M-mode Echo LV diastolic diameter, exam 5, multivariable-adjusted	http://www.ncbi.nlm.nih.gov/projects/gap/cgi-bin/analysis.cgi?id=pha001568
BloodPressure	echocardiography	LVDD6AS	M-mode Echo LV diastolic diameter, exam 6, age-sex-adjusted	http://www.ncbi.nlm.nih.gov/projects/gap/cgi-bin/analysis.cgi?id=pha001570
BloodPressure	echocardiography	LVDD6MV	M-mode Echo LV diastolic diameter, exam 6, multivariable-adjusted	http://www.ncbi.nlm.nih.gov/projects/gap/cgi-bin/analysis.cgi?id=pha001572
BloodPressure	echocardiography	LVDS26AVGAS	M-mode Echo LV systolic diameter, avg age-sex resid, exams 2,4,5,6	http://www.ncbi.nlm.nih.gov/projects/gap/cgi-bin/analysis.cgi?id=pha001574
BloodPressure	echocardiography	LVDS26AVGMV	M-mode Echo LV systolic diameter, avg multivar resid, exams 2,4,5,6	http://www.ncbi.nlm.nih.gov/projects/gap/cgi-bin/analysis.cgi?id=pha001576
BloodPressure	echocardiography	LVDS2AS	M-mode Echo LV systolic diameter, exam 2, age-sex-adjusted	http://www.ncbi.nlm.nih.gov/projects/gap/cgi-bin/analysis.cgi?id=pha001578
BloodPressure	echocardiography	LVDS2MV	M-mode Echo LV systolic diameter, exam 2, multivariable-adjusted	http://www.ncbi.nlm.nih.gov/projects/gap/cgi-bin/analysis.cgi?id=pha001580
BloodPressure	echocardiography	LVDS4AS	M-mode Echo LV systolic diameter, exam 4, age-sex-adjusted	http://www.ncbi.nlm.nih.gov/projects/gap/cgi-bin/analysis.cgi?id=pha001582
BloodPressure	echocardiography	LVDS4MV	M-mode Echo LV systolic diameter, exam 4, multivariable-adjusted	http://www.ncbi.nlm.nih.gov/projects/gap/cgi-bin/analysis.cgi?id=pha001584
BloodPressure	echocardiography	LVDS5AS	M-mode Echo LV systolic diameter, exam 5, age-sex-adjusted	http://www.ncbi.nlm.nih.gov/projects/gap/cgi-bin/analysis.cgi?id=pha001586
BloodPressure	echocardiography	LVDS5MV	M-mode Echo LV systolic diameter, exam 5, multivariable-adjusted	http://www.ncbi.nlm.nih.gov/projects/gap/cgi-bin/analysis.cgi?id=pha001588
BloodPressure	echocardiography	LVDS6AS	M-mode Echo LV systolic diameter, exam 6, age-sex-adjusted	http://www.ncbi.nlm.nih.gov/projects/gap/cgi-bin/analysis.cgi?id=pha001590
BloodPressure	echocardiography	LVDS6MV	M-mode Echo LV systolic diameter, exam 6, multivariable-adjusted	http://www.ncbi.nlm.nih.gov/projects/gap/cgi-bin/analysis.cgi?id=pha001592
BloodPressure	echocardiography	LVFS26AVGAS	M-mode Echo LV fractional shortening, avg age-sex resid, exams 2,4,5,6	http://www.ncbi.nlm.nih.gov/projects/gap/cgi-bin/analysis.cgi?id=pha001594
BloodPressure	echocardiography	LVFS26AVGMV	M-mode Echo LV fractional shortening, avg multivar resid, exams 2,4,5,6	http://www.ncbi.nlm.nih.gov/projects/gap/cgi-bin/analysis.cgi?id=pha001596
BloodPressure	echocardiography	LVFS2AS	M-mode Echo LV fractional shortening, exam 2, age-sex-adjusted	http://www.ncbi.nlm.nih.gov/projects/gap/cgi-bin/analysis.cgi?id=pha001598
BloodPressure	echocardiography	LVFS2MV	M-mode Echo LV fractional shortening, exam 2, multivariable-adjusted	http://www.ncbi.nlm.nih.gov/projects/gap/cgi-bin/analysis.cgi?id=pha001600
BloodPressure	echocardiography	LVFS4AS	M-mode Echo LV fractional shortening, exam 4, age-sex-adjusted	http://www.ncbi.nlm.nih.gov/projects/gap/cgi-bin/analysis.cgi?id=pha001602
BloodPressure	echocardiography	LVFS4MV	M-mode Echo LV fractional shortening, exam 4, multivariable-adjusted	http://www.ncbi.nlm.nih.gov/projects/gap/cgi-bin/analysis.cgi?id=pha001604
BloodPressure	echocardiography	LVFS5AS	M-mode Echo LV fractional shortening, exam 5, age-sex-adjusted	http://www.ncbi.nlm.nih.gov/projects/gap/cgi-bin/analysis.cgi?id=pha001606
BloodPressure	echocardiography	LVFS5MV	M-mode Echo LV fractional shortening, exam 5, multivariable-adjusted	http://www.ncbi.nlm.nih.gov/projects/gap/cgi-bin/analysis.cgi?id=pha001608
BloodPressure	echocardiography	LVFS6AS	M-mode Echo LV fractional shortening, exam 6, age-sex-adjusted	http://www.ncbi.nlm.nih.gov/projects/gap/cgi-bin/analysis.cgi?id=pha001610
BloodPressure	echocardiography	LVFS6MV	M-mode Echo LV fractional shortening, exam 6, multivariable-adjusted	http://www.ncbi.nlm.nih.gov/projects/gap/cgi-bin/analysis.cgi?id=pha001612
BloodPressure	echocardiography	LVM26AVGAS	M-mode Echo LV mass, avg age-sex resid, exams 2,4,5,6	http://www.ncbi.nlm.nih.gov/projects/gap/cgi-bin/analysis.cgi?id=pha001614
BloodPressure	echocardiography	LVM26AVGMV	M-mode Echo LV mass, avg multivar resid, exams 2,4,5,6	http://www.ncbi.nlm.nih.gov/projects/gap/cgi-bin/analysis.cgi?id=pha001616
BloodPressure	echocardiography	LVM2AS	M-mode Echo LV mass, exam 2, age-sex-adjusted	http://www.ncbi.nlm.nih.gov/projects/gap/cgi-bin/analysis.cgi?id=pha001618
BloodPressure	echocardiography	LVM2MV	M-mode Echo LV mass, exam 2, multivariable-adjusted	http://www.ncbi.nlm.nih.gov/projects/gap/cgi-bin/analysis.cgi?id=pha001620
BloodPressure	echocardiography	LVM4AS	M-mode Echo LV mass, exam 4, age-sex-adjusted	http://www.ncbi.nlm.nih.gov/projects/gap/cgi-bin/analysis.cgi?id=pha001622
BloodPressure	echocardiography	LVM4MV	M-mode Echo LV mass, exam 4, multivariable-adjusted	http://www.ncbi.nlm.nih.gov/projects/gap/cgi-bin/analysis.cgi?id=pha001624
BloodPressure	echocardiography	LVM5AS	M-mode Echo LV mass, exam 5, age-sex-adjusted	http://www.ncbi.nlm.nih.gov/projects/gap/cgi-bin/analysis.cgi?id=pha001626

Group	Category	Trait Label	Name	GEE Link
BloodPressure	echocardiography	LVM5MV	M-mode Echo LV mass, exam 5, multivariable-adjusted	http://www.ncbi.nlm.nih.gov/projects/gap/cgi-bin/analysis.cgi?id=pha001628
BloodPressure	echocardiography	LVM6AS	M-mode Echo LV mass, exam 6, age-sex-adjusted	http://www.ncbi.nlm.nih.gov/projects/gap/cgi-bin/analysis.cgi?id=pha001630
BloodPressure	echocardiography	LVM6MV	M-mode Echo LV mass, exam 6, multivariable-adjusted	http://www.ncbi.nlm.nih.gov/projects/gap/cgi-bin/analysis.cgi?id=pha001632
BloodPressure	echocardiography	LVWT26AVGAS	M-mode Echo LV diastolic wall thickness, avg age-sex resid, exams 2,4,5,6	http://www.ncbi.nlm.nih.gov/projects/gap/cgi-bin/analysis.cgi?id=pha001634
BloodPressure	echocardiography	LVWT26AVGMV	M-mode Echo LV diastolic wall thickness, avg multivar resid, exams 2,4,5,6	http://www.ncbi.nlm.nih.gov/projects/gap/cgi-bin/analysis.cgi?id=pha001636
BloodPressure	echocardiography	LVWT2AS	M-mode Echo LV diastolic wall thickness, exam 2, age-sex-adjusted	http://www.ncbi.nlm.nih.gov/projects/gap/cgi-bin/analysis.cgi?id=pha001638
BloodPressure	echocardiography	LVWT2MV	M-mode Echo LV diastolic wall thickness, exam 2, multivariable-adjusted	http://www.ncbi.nlm.nih.gov/projects/gap/cgi-bin/analysis.cgi?id=pha001640
BloodPressure	echocardiography	LVWT4AS	M-mode Echo LV diastolic wall thickness, exam 4, age-sex-adjusted	http://www.ncbi.nlm.nih.gov/projects/gap/cgi-bin/analysis.cgi?id=pha001642
BloodPressure	echocardiography	LVWT4MV	M-mode Echo LV diastolic wall thickness, exam 4, multivariable-adjusted	http://www.ncbi.nlm.nih.gov/projects/gap/cgi-bin/analysis.cgi?id=pha001644
BloodPressure	echocardiography	LVWT5AS	M-mode Echo LV diastolic wall thickness, exam 5, age-sex-adjusted	http://www.ncbi.nlm.nih.gov/projects/gap/cgi-bin/analysis.cgi?id=pha001646
BloodPressure	echocardiography	LVWT5MV	M-mode Echo LV diastolic wall thickness, exam 5, multivariable-adjusted	http://www.ncbi.nlm.nih.gov/projects/gap/cgi-bin/analysis.cgi?id=pha001648
BloodPressure	echocardiography	LVWT6AS	M-mode Echo LV diastolic wall thickness, exam 6, age-sex-adjusted	http://www.ncbi.nlm.nih.gov/projects/gap/cgi-bin/analysis.cgi?id=pha001650
BloodPressure	echocardiography	LVWT6MV	M-mode Echo LV diastolic wall thickness, exam 6, multivariable-adjusted	http://www.ncbi.nlm.nih.gov/projects/gap/cgi-bin/analysis.cgi?id=pha001652
BloodPressure	Endothelialfunction	BASEFLOW7AS	Brachial artery Baseline flow velocity, exam 7, age-sex-adjusted	http://www.ncbi.nlm.nih.gov/projects/gap/cgi-bin/analysis.cgi?id=pha001654
BloodPressure	Endothelialfunction	BASEFLOW7MV	Brachial artery Baseline flow velocity, exam 7, multivariable-adjusted	http://www.ncbi.nlm.nih.gov/projects/gap/cgi-bin/analysis.cgi?id=pha001656
BloodPressure	Endothelialfunction	BASELINEDIAMETERAS7	baseline brachial artery diameter, exam 7, age and sex-adjusted	http://www.ncbi.nlm.nih.gov/projects/gap/cgi-bin/analysis.cgi?id=pha001658
BloodPressure	Endothelialfunction	BASELINEDIAMETERMV7	baseline brachial artery diameter, exam 7, multivariable-adjusted	http://www.ncbi.nlm.nih.gov/projects/gap/cgi-bin/analysis.cgi?id=pha001660
BloodPressure	Endothelialfunction	FMD7PCTAS	Brachial artery Flow mediated dilation, exam 7, age-adjusted	http://www.ncbi.nlm.nih.gov/projects/gap/cgi-bin/analysis.cgi?id=pha001662
BloodPressure	Endothelialfunction	FMD7PCTMV	Brachial artery Flow mediated dilation, exam 7, multivariable-adjusted	http://www.ncbi.nlm.nih.gov/projects/gap/cgi-bin/analysis.cgi?id=pha001664
BloodPressure	Endothelialfunction	HYPERFLOW7AS	Brachial artery hyperemic flow velocity, exam 7, age-sex-adjusted	http://www.ncbi.nlm.nih.gov/projects/gap/cgi-bin/analysis.cgi?id=pha001666
BloodPressure	Endothelialfunction	HYPERFLOW7MV	Brachial artery hyperemic flow velocity, exam 7, multivariable-adjusted	http://www.ncbi.nlm.nih.gov/projects/gap/cgi-bin/analysis.cgi?id=pha001668
BloodPressure	ExerciseTest	ETT2DBPREC3AS	Exercise recovery 3-min diastolic BP, exam 2, age-sex-adjusted	http://www.ncbi.nlm.nih.gov/projects/gap/cgi-bin/analysis.cgi?id=pha001670
BloodPressure	ExerciseTest	ETT2DBPREC3MV	Exercise recovery 3-min diastolic BP, exam 2, multivariable-adjusted	http://www.ncbi.nlm.nih.gov/projects/gap/cgi-bin/analysis.cgi?id=pha001672
BloodPressure	ExerciseTest	ETT2DBPSTG2AS	Exercise Stage 2 diastolic BP, exam 2, age-sex-adjusted	http://www.ncbi.nlm.nih.gov/projects/gap/cgi-bin/analysis.cgi?id=pha001674
BloodPressure	ExerciseTest	ETT2DBPSTG2MV	Exercise Stage 2 diastolic BP, exam 2, multivariable-adjusted	http://www.ncbi.nlm.nih.gov/projects/gap/cgi-bin/analysis.cgi?id=pha001676
BloodPressure	ExerciseTest	ETT2HRREC3AS	Exercise recovery 3-min heart rate, exam 2, age-sex-adjusted	http://www.ncbi.nlm.nih.gov/projects/gap/cgi-bin/analysis.cgi?id=pha001678
BloodPressure	ExerciseTest	ETT2HRREC3MV	Exercise recovery 3-min heart rate, exam 2, multivariable-adjusted	http://www.ncbi.nlm.nih.gov/projects/gap/cgi-bin/analysis.cgi?id=pha001680
BloodPressure	ExerciseTest	ETT2HRSTG2AS	Exercise Stage 2 heart rate, exam 2, age-sex-adjusted	http://www.ncbi.nlm.nih.gov/projects/gap/cgi-bin/analysis.cgi?id=pha001682
BloodPressure	ExerciseTest	ETT2HRSTG2MV	Exercise Stage 2 heart rate, exam 2, multivariable-adjusted	http://www.ncbi.nlm.nih.gov/projects/gap/cgi-bin/analysis.cgi?id=pha001684
BloodPressure	ExerciseTest	ETT2SBPREC3AS	Exercise recovery 3-min systolic BP, exam 2, age-sex-adjusted	http://www.ncbi.nlm.nih.gov/projects/gap/cgi-bin/analysis.cgi?id=pha001686
BloodPressure	ExerciseTest	ETT2SBPREC3MV	Exercise recovery 3-min systolic BP, exam 2, multivariable-adjusted	http://www.ncbi.nlm.nih.gov/projects/gap/cgi-bin/analysis.cgi?id=pha001688
BloodPressure	ExerciseTest	ETT2SBPSTG2AS	Exercise Stage 2 systolic BP, exam 2, age-sex-adjusted	http://www.ncbi.nlm.nih.gov/projects/gap/cgi-bin/analysis.cgi?id=pha001690
BloodPressure	ExerciseTest	ETT2SBPSTG2MV	Exercise Stage 2 systolic BP, exam 2, multivariable-adjusted	http://www.ncbi.nlm.nih.gov/projects/gap/cgi-bin/analysis.cgi?id=pha001692
BloodPressure	tonometry	AI7AS	augmentation index, exam 7, age-sex-adjusted	http://www.ncbi.nlm.nih.gov/projects/gap/cgi-bin/analysis.cgi?id=pha001694
BloodPressure	tonometry	AI7MV	augmentation index, exam 7, multivariable-adjusted	http://www.ncbi.nlm.nih.gov/projects/gap/cgi-bin/analysis.cgi?id=pha001696
BloodPressure	tonometry	CBPWV7AS	carotid-brachial pulse wave velocity, exam 7, age-sex-adjusted	http://www.ncbi.nlm.nih.gov/projects/gap/cgi-bin/analysis.cgi?id=pha001698
BloodPressure	tonometry	CBPWV7MV	carotid-brachial pulse wave velocity, exam 7, multivariable-adjusted	http://www.ncbi.nlm.nih.gov/projects/gap/cgi-bin/analysis.cgi?id=pha001700
BloodPressure	tonometry	CFPWV7AS	carotid-femoral pulse wave velocity, exam 7, age-sex-adjusted	http://www.ncbi.nlm.nih.gov/projects/gap/cgi-bin/analysis.cgi?id=pha001702
BloodPressure	tonometry	CFPWV7MV	carotid-femoral pulse wave velocity, exam 7, multivariable-adjusted	http://www.ncbi.nlm.nih.gov/projects/gap/cgi-bin/analysis.cgi?id=pha001704
BloodPressure	tonometry	CPP7AS	central pulse pressure, exam 7, age-sex-adjusted	http://www.ncbi.nlm.nih.gov/projects/gap/cgi-bin/analysis.cgi?id=pha001706
BloodPressure	tonometry	CPP7MV	central pulse pressure, exam 7, multivariable-adjusted	http://www.ncbi.nlm.nih.gov/projects/gap/cgi-bin/analysis.cgi?id=pha001708
BloodPressure	tonometry	CRPW7VAS	carotid-radial pulse wave velocity, exam 7, age-sex-adjusted	http://www.ncbi.nlm.nih.gov/projects/gap/cgi-bin/analysis.cgi?id=pha001710
BloodPressure	tonometry	CRPWV7MV	carotid-radial pulse wave velocity, exam 7, multivariable-adjusted	http://www.ncbi.nlm.nih.gov/projects/gap/cgi-bin/analysis.cgi?id=pha001712
BloodPressure	tonometry	DBP7BRAOSCAS	Diastolic BP Brachial oscillometric, exam 7, age-sex-adjusted	http://www.ncbi.nlm.nih.gov/projects/gap/cgi-bin/analysis.cgi?id=pha001714
BloodPressure	tonometry	DBP7BRAOSCMV	Diastolic BP Brachial oscillometric, exam 7, multivariable-adjusted	http://www.ncbi.nlm.nih.gov/projects/gap/cgi-bin/analysis.cgi?id=pha001716
BloodPressure	tonometry	FWDWAVE7AS	forward wave amplitude, exam 7, age-sex-adjusted	http://www.ncbi.nlm.nih.gov/projects/gap/cgi-bin/analysis.cgi?id=pha001718
BloodPressure	tonometry	FWDWAVE7MV	forward wave amplitude, exam 7, multivariable-adjusted	http://www.ncbi.nlm.nih.gov/projects/gap/cgi-bin/analysis.cgi?id=pha001720
BloodPressure	tonometry	INVCFPWV7AS	LV ejection time, exam 7, age-sex-adjusted	http://www.ncbi.nlm.nih.gov/projects/gap/cgi-bin/analysis.cgi?id=pha001722
BloodPressure	tonometry	INVCFPWV7MV	LV ejection time, exam 7, multivariable-adjusted	http://www.ncbi.nlm.nih.gov/projects/gap/cgi-bin/analysis.cgi?id=pha001724
BloodPressure	tonometry	MAP7AS	mean arterial pressure, exam 7, age-sex-adjusted	http://www.ncbi.nlm.nih.gov/projects/gap/cgi-bin/analysis.cgi?id=pha001726
BloodPressure	tonometry	MAP7MV	mean arterial pressure, exam 7, multivariable-adjusted	http://www.ncbi.nlm.nih.gov/projects/gap/cgi-bin/analysis.cgi?id=pha001728
BloodPressure	tonometry	PERAMPAP7AS	Peripheral amplification apparent, exam 7, age-sex-adjusted	http://www.ncbi.nlm.nih.gov/projects/gap/cgi-bin/analysis.cgi?id=pha001730
BloodPressure	tonometry	PERAMPAP7MV	Peripheral amplification apparent, exam 7, multivariable-adjusted	http://www.ncbi.nlm.nih.gov/projects/gap/cgi-bin/analysis.cgi?id=pha001732
BloodPressure	tonometry	PERAMPTRU7AS	Peripheral amplification true, exam 7, age-sex-adjusted	http://www.ncbi.nlm.nih.gov/projects/gap/cgi-bin/analysis.cgi?id=pha001734
BloodPressure	tonometry	PERAMPTRU7MV	Peripheral amplification true, exam 7, multivariable-adjusted	http://www.ncbi.nlm.nih.gov/projects/gap/cgi-bin/analysis.cgi?id=pha001736

Group	Category	Trait Label	Name	GEE Link
BloodPressure	tonometry	PP7BRAOSCAS	Pulse pressure Brachial oscillometric, exam 7, age-sex-adjusted	http://www.ncbi.nlm.nih.gov/projects/gap/cgi-bin/analysis.cgi?id=pha001738
BloodPressure	tonometry	PP7BRAOSCMV	Pulse pressure Brachial oscillometric, exam 7, multivariable-adjusted	http://www.ncbi.nlm.nih.gov/projects/gap/cgi-bin/analysis.cgi?id=pha001740
BloodPressure	tonometry	REFWAVE7AS	reflected wave amplitude, exam 7, age-sex-adjusted	http://www.ncbi.nlm.nih.gov/projects/gap/cgi-bin/analysis.cgi?id=pha001742
BloodPressure	tonometry	REFWAVE7MV	reflected wave amplitude, exam 7, multivariable-adjusted	http://www.ncbi.nlm.nih.gov/projects/gap/cgi-bin/analysis.cgi?id=pha001744
BloodPressure	tonometry	RWTT7AS	reflected wave transit time, exam 7, age-sex-adjusted	http://www.ncbi.nlm.nih.gov/projects/gap/cgi-bin/analysis.cgi?id=pha001746
BloodPressure	tonometry	RWTT7MV	reflected wave transit time, exam 7, multivariable-adjusted	http://www.ncbi.nlm.nih.gov/projects/gap/cgi-bin/analysis.cgi?id=pha001748
BloodPressure	tonometry	SBP7BRAOSCAS	Systolic BP Brachial oscillometric, exam 7, age-sex-adjusted	http://www.ncbi.nlm.nih.gov/projects/gap/cgi-bin/analysis.cgi?id=pha001750
BloodPressure	tonometry	SBP7BRAOSCMV	Systolic BP Brachial oscillometric, exam 7, multivariable-adjusted	http://www.ncbi.nlm.nih.gov/projects/gap/cgi-bin/analysis.cgi?id=pha001752
CVDMiscTraits	Cancer	ALLCANCER1	all cancer, adjusted for age & sex	http://www.ncbi.nlm.nih.gov/projects/gap/cgi-bin/analysis.cgi?id=pha001332
CVDMiscTraits	Cancer	ALLCANCER2	all cancer, fully adjusted	http://www.ncbi.nlm.nih.gov/projects/gap/cgi-bin/analysis.cgi?id=pha001334
CVDMiscTraits	Cancer	BREASTCANCER1	breast cancer (women only), age-adjusted	http://www.ncbi.nlm.nih.gov/projects/gap/cgi-bin/analysis.cgi?id=pha001336
CVDMiscTraits	Cancer	BREASTCANCER2	breast cancer (women only), adjusted for age, parity, BMI at entry	http://www.ncbi.nlm.nih.gov/projects/gap/cgi-bin/analysis.cgi?id=pha001338
CVDMiscTraits	Cancer	PROSTATECANCER1	prostate cancer, adjusted for age at entry	http://www.ncbi.nlm.nih.gov/projects/gap/cgi-bin/analysis.cgi?id=pha001340
CVDMiscTraits	CVDbySOE	AllatheroCVD1D	Age- & sex- adjusted incident CHD, ABI, TIA, or IC	http://www.ncbi.nlm.nih.gov/projects/gap/cgi-bin/analysis.cgi?id=pha001342
CVDMiscTraits	CVDbySOE	AllatheroCVD2D	multivariable-adjusted incident CHD, ABI, TIA, or IC	http://www.ncbi.nlm.nih.gov/projects/gap/cgi-bin/analysis.cgi?id=pha001344
CVDMiscTraits	CVDbySOE	AllCHD1D	Age- & sex-adjusted incident MI, AP, CI, or CHD death	http://www.ncbi.nlm.nih.gov/projects/gap/cgi-bin/analysis.cgi?id=pha001346
CVDMiscTraits	CVDbySOE	AllCHD2D	multivariable-adjusted incident MI, AP, CI, or CHD death	http://www.ncbi.nlm.nih.gov/projects/gap/cgi-bin/analysis.cgi?id=pha001348
CVDMiscTraits	CVDbySOE	ALLCHF1D	Age- & sex-adjusted incident heart failure (free of MI)	http://www.ncbi.nlm.nih.gov/projects/gap/cgi-bin/analysis.cgi?id=pha001350
CVDMiscTraits	CVDbySOE	ALLCHF2D	multivariable-adjusted incident heart failure (free of MI)	http://www.ncbi.nlm.nih.gov/projects/gap/cgi-bin/analysis.cgi?id=pha001352
CVDMiscTraits	CVDbySOE	HardatheroCVD1D	Age- & sex-adjusted incident MI, CI, CHD death, or ABI	http://www.ncbi.nlm.nih.gov/projects/gap/cgi-bin/analysis.cgi?id=pha001358
CVDMiscTraits	CVDbySOE	HardatheroCVD2D	multivariable-adjusted incident MI, CI, CHD death, or ABI	http://www.ncbi.nlm.nih.gov/projects/gap/cgi-bin/analysis.cgi?id=pha001360
CVDMiscTraits	CVDbySOE	HardCHD1D	Age- & sex-adjusted incident MI, CI, or CHD death	http://www.ncbi.nlm.nih.gov/projects/gap/cgi-bin/analysis.cgi?id=pha001362
CVDMiscTraits	CVDbySOE	HardCHD2D	multivariable-adjusted incident MI, CI, or CHD death	http://www.ncbi.nlm.nih.gov/projects/gap/cgi-bin/analysis.cgi?id=pha001364
CVDMiscTraits	CVDbySOE	NoMICHF1D	Age- & sex-adjusted incident heart faliure, censored at interval MI	http://www.ncbi.nlm.nih.gov/projects/gap/cgi-bin/analysis.cgi?id=pha001366
CVDMiscTraits	CVDbySOE	NoMICHF2D	multivariable-adjusted incident heart failure, censored at interval MI	http://www.ncbi.nlm.nih.gov/projects/gap/cgi-bin/analysis.cgi?id=pha001368
CVDMiscTraits	ECGtraits	PRAdjRRPOOL	Age & RR adjusted PR interval cohort ex 11 offspring ex 1	http://www.ncbi.nlm.nih.gov/projects/gap/cgi-bin/analysis.cgi?id=pha001370
CVDMiscTraits	ECGtraits	QTMEN	Age & RR adjusted QT interval men cohort ex 11 offspring ex 1	http://www.ncbi.nlm.nih.gov/projects/gap/cgi-bin/analysis.cgi?id=pha001372
CVDMiscTraits	ECGtraits	QTPOOL	Age & RR adjusted QT interval men & women cohort ex 11 offspring ex 1	http://www.ncbi.nlm.nih.gov/projects/gap/cgi-bin/analysis.cgi?id=pha001374
CVDMiscTraits	ECGtraits	QTWOMEN	Age & RR adjusted QT interval women cohort ex 11 offspring ex 1	http://www.ncbi.nlm.nih.gov/projects/gap/cgi-bin/analysis.cgi?id=pha001376
CVDMiscTraits	ECGtraits	RRMEN	Age adjusted RR interval men cohort ex 11 offspring ex 1	http://www.ncbi.nlm.nih.gov/projects/gap/cgi-bin/analysis.cgi?id=pha001378
CVDMiscTraits	ECGtraits	RRPOOL	Age adjusted RR interval men & women cohort ex 11 offspring ex 1	http://www.ncbi.nlm.nih.gov/projects/gap/cgi-bin/analysis.cgi?id=pha001380
CVDMiscTraits	ECGtraits	RRWOMEN	Age adjusted RR interval women cohort ex 11 offspring ex 1	http://www.ncbi.nlm.nih.gov/projects/gap/cgi-bin/analysis.cgi?id=pha001382
CVDMiscTraits	HRVtraits	HFHRV	Age & HR adjusted high freq power HRV exams 18 (cohort), 3 (offspring)	http://www.ncbi.nlm.nih.gov/projects/gap/cgi-bin/analysis.cgi?id=pha001384
CVDMiscTraits	HRVtraits	LFHFHRV	Age & HR adjusted low:high freq power HRV exams 18 (cohort), 3 (offspring)	http://www.ncbi.nlm.nih.gov/projects/gap/cgi-bin/analysis.cgi?id=pha001386
CVDMiscTraits	HRVtraits	LFHRV	Age & HR adjusted low frequency power HRV exams 18 (cohort), 3 (offspring)	http://www.ncbi.nlm.nih.gov/projects/gap/cgi-bin/analysis.cgi?id=pha001388
CVDMiscTraits	HRVtraits	PNN50HRV	Age & HR adjusted PNN50 HRV exams 18 (cohort), 3 (offspring)	http://www.ncbi.nlm.nih.gov/projects/gap/cgi-bin/analysis.cgi?id=pha001390
CVDMiscTraits	HRVtraits	SDNNHRV	Age & HR adjsuted SDNN HRV exams 18 (cohort), 3 (offspring)	http://www.ncbi.nlm.nih.gov/projects/gap/cgi-bin/analysis.cgi?id=pha001392
CVDMiscTraits	HRVtraits	TOTPWRHRV	Age & HR adjsuted total power HRV exams 18 (cohort), 3 (offspring)	http://www.ncbi.nlm.nih.gov/projects/gap/cgi-bin/analysis.cgi?id=pha001394
CVDMiscTraits	HRVtraits	VLFHRV	Age & HR adjusted very low freq power HRV exams 18 (cohort), 3 (offspring)	http://www.ncbi.nlm.nih.gov/projects/gap/cgi-bin/analysis.cgi?id=pha001396
CVDMiscTraits	Otherclinicaltraits	ALCOHOL1	Age- & sex-adjusted drinks/day ex 1	http://www.ncbi.nlm.nih.gov/projects/gap/cgi-bin/analysis.cgi?id=pha001398
CVDMiscTraits	Otherclinicaltraits	ALCOHOL2	Age- & sex-adjusted drinks/day across all exams	http://www.ncbi.nlm.nih.gov/projects/gap/cgi-bin/analysis.cgi?id=pha001400
CVDMiscTraits	Otherclinicaltraits	ALCOHOL3	Age- & sex-adjusted max drinks/day at any exam	http://www.ncbi.nlm.nih.gov/projects/gap/cgi-bin/analysis.cgi?id=pha001402
CVDMiscTraits	Otherclinicaltraits	GALLBLADDER	Age- & sex-adjusted gallbladder disease	http://www.ncbi.nlm.nih.gov/projects/gap/cgi-bin/analysis.cgi?id=pha001404
CVDMiscTraits	Otherclinicaltraits	GOUT	Age-, sex- & BMI-adjusted gout	http://www.ncbi.nlm.nih.gov/projects/gap/cgi-bin/analysis.cgi?id=pha001406
CVDMiscTraits	Othervasctraits	ALLAF1	Age- & sex-adjusted incident atrial fibrillation	http://www.ncbi.nlm.nih.gov/projects/gap/cgi-bin/analysis.cgi?id=pha001408
CVDMiscTraits	Othervasctraits	ALLAF2	multivariable-adjusted incident atrial fibrillation	http://www.ncbi.nlm.nih.gov/projects/gap/cgi-bin/analysis.cgi?id=pha001410
CVDMiscTraits	Othervasctraits	VARICOSEVEINS	age- & sex-adjusted varicose veins, men and women	http://www.ncbi.nlm.nih.gov/projects/gap/cgi-bin/analysis.cgi?id=pha001412
CVDMiscTraits	Othervasctraits	VARICOSEVEINSF	age- & #childbirths-adjusted varicose veins in women	http://www.ncbi.nlm.nih.gov/projects/gap/cgi-bin/analysis.cgi?id=pha001414
CVDMiscTraits	Othervasctraits	VARICOSEVEINSM	age-adjusted varicose veins in men	http://www.ncbi.nlm.nih.gov/projects/gap/cgi-bin/analysis.cgi?id=pha001416
ITBiomarkers	Hematological	Hctavg12as	Hematocrit, averaged residuals of exam 1 & 2, age & sex	http://www.ncbi.nlm.nih.gov/projects/gap/cgi-bin/analysis.cgi?id=pha000980
ITBiomarkers	Hematological	Hctavg12mv	Hematocrit, averaged residuals of exam 1 & 2, multivariable	http://www.ncbi.nlm.nih.gov/projects/gap/cgi-bin/analysis.cgi?id=pha000982
ITBiomarkers	Hematological	Hctex1as	Hematocrit exam 1 age & sex	http://www.ncbi.nlm.nih.gov/projects/gap/cgi-bin/analysis.cgi?id=pha000984
ITBiomarkers	Hematological	Hctex1mv	Hematocrit exam 1 multivariable	http://www.ncbi.nlm.nih.gov/projects/gap/cgi-bin/analysis.cgi?id=pha000986
ITBiomarkers	Hematological	Hctex2as	Hematocrit exam 2 age & sex	http://www.ncbi.nlm.nih.gov/projects/gap/cgi-bin/analysis.cgi?id=pha000988
ITBiomarkers	Hematological	Hctex2mv	Hematocrit exam 2 multivariable	http://www.ncbi.nlm.nih.gov/projects/gap/cgi-bin/analysis.cgi?id=pha000990

Group	Category	Trait Label	Name	GEE Link
ITBiomarkers	Hematological	Hgbavg12as	Hemoglobin, averaged residuals of exam 1 & 2, age & sex	http://www.ncbi.nlm.nih.gov/projects/gap/cgi-bin/analysis.cgi?id=pha000992
ITBiomarkers	Hematological	Hgbavg12mv	Hemoglobin averaged residuals of exam 1 & 2, multivariable	http://www.ncbi.nlm.nih.gov/projects/gap/cgi-bin/analysis.cgi?id=pha000994
ITBiomarkers	Hematological	Hgbex1as	Hemoglobin exam 1 age & sex	http://www.ncbi.nlm.nih.gov/projects/gap/cgi-bin/analysis.cgi?id=pha000996
ITBiomarkers	Hematological	Hgbex1mv	Hemoglobin exam 1 multivariable	http://www.ncbi.nlm.nih.gov/projects/gap/cgi-bin/analysis.cgi?id=pha000998
ITBiomarkers	Hematological	Hgbex2as	Hemoglobin exam 2 age & sex	http://www.ncbi.nlm.nih.gov/projects/gap/cgi-bin/analysis.cgi?id=pha001000
ITBiomarkers	Hematological	Hgbex2mv	Hemoglobin exam 2 multivariable	http://www.ncbi.nlm.nih.gov/projects/gap/cgi-bin/analysis.cgi?id=pha001002
ITBiomarkers	Hematological	MCHCavg12as	Mean corpuscular hemoglobin concentration avg. residuals exam 1&2 age & sex	http://www.ncbi.nlm.nih.gov/projects/gap/cgi-bin/analysis.cgi?id=pha001004
ITBiomarkers	Hematological	MCHCavg12mv	Mean corpuscular hemoglobin concentration avg. residuals of exam 1&2 MV	http://www.ncbi.nlm.nih.gov/projects/gap/cgi-bin/analysis.cgi?id=pha001006
ITBiomarkers	Hematological	MCHCex1as	Mean corpuscular hemoglobin concentration exam 1 age & sex	http://www.ncbi.nlm.nih.gov/projects/gap/cgi-bin/analysis.cgi?id=pha001008
ITBiomarkers	Hematological	MCHCex1mv	Mean corpuscular hemoglobin concentration exam 1 multivariable	http://www.ncbi.nlm.nih.gov/projects/gap/cgi-bin/analysis.cgi?id=pha001010
ITBiomarkers	Hematological	MCHCex2as	Mean corpuscular hemoglobin concentration exam 2 age & sex	http://www.ncbi.nlm.nih.gov/projects/gap/cgi-bin/analysis.cgi?id=pha001012
ITBiomarkers	Hematological	MCHCex2mv	Mean corpuscular hemoglobin concentration exam 2 multivariable	http://www.ncbi.nlm.nih.gov/projects/gap/cgi-bin/analysis.cgi?id=pha001014
ITBiomarkers	Hematological	MCVavg12as	Mean corpuscular volume averaged residuals of exam 1 &2, age & sex	http://www.ncbi.nlm.nih.gov/projects/gap/cgi-bin/analysis.cgi?id=pha001016
ITBiomarkers	Hematological	MCVavg12mv	Mean corpuscular volume averaged residuals of exam 1 and 2, multivariable	http://www.ncbi.nlm.nih.gov/projects/gap/cgi-bin/analysis.cgi?id=pha001018
ITBiomarkers	Hematological	MCVex1as	Mean corpuscular volume exam 1 age & sex	http://www.ncbi.nlm.nih.gov/projects/gap/cgi-bin/analysis.cgi?id=pha001020
ITBiomarkers	Hematological	MCVex1mv	Mean corpuscular volume multivariable	http://www.ncbi.nlm.nih.gov/projects/gap/cgi-bin/analysis.cgi?id=pha001022
ITBiomarkers	Hematological	MCVex2as	Mean corpuscular volume exam 2 age & sex	http://www.ncbi.nlm.nih.gov/projects/gap/cgi-bin/analysis.cgi?id=pha001024
ITBiomarkers	Hematological	MCVex2mv	Mean corpuscular volume multivariable exam 2	http://www.ncbi.nlm.nih.gov/projects/gap/cgi-bin/analysis.cgi?id=pha001026
ITBiomarkers	Hematological	RBCCavg12as	Red blood cell count averaged residuals of exam 1 & 2, age & sex	http://www.ncbi.nlm.nih.gov/projects/gap/cgi-bin/analysis.cgi?id=pha001028
ITBiomarkers	Hematological	RBCCavg12mv	Red blood cell count averaged residuals exam 1 & 2, multivariable	http://www.ncbi.nlm.nih.gov/projects/gap/cgi-bin/analysis.cgi?id=pha001030
ITBiomarkers	Hematological	RBCCex1as	Red blood cell count exam 1 age & sex	http://www.ncbi.nlm.nih.gov/projects/gap/cgi-bin/analysis.cgi?id=pha001032
ITBiomarkers	Hematological	RBCCex1mv	Red blood cell count exam 1 multivariable	http://www.ncbi.nlm.nih.gov/projects/gap/cgi-bin/analysis.cgi?id=pha001034
ITBiomarkers	Hematological	RBCCex2as	Red blood cell count exam 2 age & sex	http://www.ncbi.nlm.nih.gov/projects/gap/cgi-bin/analysis.cgi?id=pha001036
ITBiomarkers	Hematological	RBCCex2asWIN	Red blood cell count exam 2 age & sex, Winsorized	http://www.ncbi.nlm.nih.gov/projects/gap/cgi-bin/analysis.cgi?id=pha001038
ITBiomarkers	Hematological	RBCCex2mv	Red blood cell count exam 2 multivariable	http://www.ncbi.nlm.nih.gov/projects/gap/cgi-bin/analysis.cgi?id=pha001040
ITBiomarkers	Hematological	RBCCex2mvWIN	Red blood cell count exam 2 multivariable, Winsorized	http://www.ncbi.nlm.nih.gov/projects/gap/cgi-bin/analysis.cgi?id=pha001042
ITBiomarkers	Hematological	WBCavg12as	White blood cell count averaged residuals of exam 1 & 2, age & sex	http://www.ncbi.nlm.nih.gov/projects/gap/cgi-bin/analysis.cgi?id=pha001044
ITBiomarkers	Hematological	WBCavg12mv	White blood cell count, averaged residuals of exam 1 & 2, multivariable	http://www.ncbi.nlm.nih.gov/projects/gap/cgi-bin/analysis.cgi?id=pha001046
ITBiomarkers	Hematological	WBCex1as	White blood cell count exam 1 age & sex, log transformed	http://www.ncbi.nlm.nih.gov/projects/gap/cgi-bin/analysis.cgi?id=pha001048
ITBiomarkers	Hematological	WBCex1mv	White blood cell count exam 1 multivariable, log transformed	http://www.ncbi.nlm.nih.gov/projects/gap/cgi-bin/analysis.cgi?id=pha001050
ITBiomarkers	Hematological	WBCex2as	White blood cell count exam 2 age & sex	http://www.ncbi.nlm.nih.gov/projects/gap/cgi-bin/analysis.cgi?id=pha001052
ITBiomarkers	Hematological	WBCex2mv	White blood cell count exam 2 multivariable	http://www.ncbi.nlm.nih.gov/projects/gap/cgi-bin/analysis.cgi?id=pha001054
ITBiomarkers	Hemostatic	dDimerex5as	D dimer at exam 6 age & sex, log transformed	http://www.ncbi.nlm.nih.gov/projects/gap/cgi-bin/analysis.cgi?id=pha001056
ITBiomarkers	Hemostatic	dDimerex5mv	D dimer at exam 6 multivariable, log transformed	http://www.ncbi.nlm.nih.gov/projects/gap/cgi-bin/analysis.cgi?id=pha001058
ITBiomarkers	Hemostatic	FibrinogenAvg567as	Fibrinogen averaged residuals of exam 5, 6 & 7 age & sex, log transformed	http://www.ncbi.nlm.nih.gov/projects/gap/cgi-bin/analysis.cgi?id=pha001060
ITBiomarkers	Hemostatic	FibrinogenAvg567mv	Fibrinogen averaged residuals exam 5, 6 & 7 multivariable, log transformed	http://www.ncbi.nlm.nih.gov/projects/gap/cgi-bin/analysis.cgi?id=pha001062
ITBiomarkers	Hemostatic	Fibrinogenex5as	Fibrinogen at exam 5 age & sex, log transformed	http://www.ncbi.nlm.nih.gov/projects/gap/cgi-bin/analysis.cgi?id=pha001064
ITBiomarkers	Hemostatic	Fibrinogenex5mv	Fibrinogen at exam 5 multivariable, log transformed	http://www.ncbi.nlm.nih.gov/projects/gap/cgi-bin/analysis.cgi?id=pha001066
ITBiomarkers	Hemostatic	Fibrinogenex6as	Fibrinogen at exam 6 age & sex, log transformed	http://www.ncbi.nlm.nih.gov/projects/gap/cgi-bin/analysis.cgi?id=pha001068
ITBiomarkers	Hemostatic	Fibrinogenex6mv	Fibrinogen at exam 6 multivariable, log transformed	http://www.ncbi.nlm.nih.gov/projects/gap/cgi-bin/analysis.cgi?id=pha001070
ITBiomarkers	Hemostatic	Fibrinogenex7as	Fibrinogen at exam 7 age & sex, log transformed	http://www.ncbi.nlm.nih.gov/projects/gap/cgi-bin/analysis.cgi?id=pha001072
ITBiomarkers	Hemostatic	Fibrinogenex7mv	Fibrinogen at exam 7 multivariable, log transformed	http://www.ncbi.nlm.nih.gov/projects/gap/cgi-bin/analysis.cgi?id=pha001074
ITBiomarkers	Hemostatic	FVIIex5as	Factor VII at exam 5 age & sex	http://www.ncbi.nlm.nih.gov/projects/gap/cgi-bin/analysis.cgi?id=pha001076
ITBiomarkers	Hemostatic	FVIIex5mv	Factor VII at exam 5 multivariable	http://www.ncbi.nlm.nih.gov/projects/gap/cgi-bin/analysis.cgi?id=pha001078
ITBiomarkers	Hemostatic	PAI1ex56Avgas	Log plasminogen Activator 1 avg. residuals exam 5& 6 age & sex	http://www.ncbi.nlm.nih.gov/projects/gap/cgi-bin/analysis.cgi?id=pha001080
ITBiomarkers	Hemostatic	PAI1ex56Avgmv	Log plasminogen Activator 1 avg. residuals exam 5 & 6 multivariable	http://www.ncbi.nlm.nih.gov/projects/gap/cgi-bin/analysis.cgi?id=pha001082
ITBiomarkers	Hemostatic	PAI1ex5as	Plasminogen Activator 1 at exam 5 age & sex, log transformed	http://www.ncbi.nlm.nih.gov/projects/gap/cgi-bin/analysis.cgi?id=pha001084
ITBiomarkers	Hemostatic	PAI1ex5mv	Plasminogen Activator 1 at exam 5 multivariable, log transformed	http://www.ncbi.nlm.nih.gov/projects/gap/cgi-bin/analysis.cgi?id=pha001086
ITBiomarkers	Hemostatic	PAI1ex6as	Plasminogen Activator 1 at exam 6 age & sex, log transformed	http://www.ncbi.nlm.nih.gov/projects/gap/cgi-bin/analysis.cgi?id=pha001088
ITBiomarkers	Hemostatic	PAI1ex6mv	Plasminogen Activator 1 at exam 6 multivariable, log transformed	http://www.ncbi.nlm.nih.gov/projects/gap/cgi-bin/analysis.cgi?id=pha001090
ITBiomarkers	Hemostatic	PlateletAgADPex5as	Platelet Aggregation to ADP exam 5, age & sex, log transformed	http://www.ncbi.nlm.nih.gov/projects/gap/cgi-bin/analysis.cgi?id=pha001092
ITBiomarkers	Hemostatic	PlateletAgADPex5mv	Log ADP platelet aggregation, exam 5, multivariable	http://www.ncbi.nlm.nih.gov/projects/gap/cgi-bin/analysis.cgi?id=pha001094
ITBiomarkers	Hemostatic	PlateletAgCollex5as	Platelet Aggregation to collagen, exam 5, age & sex, log transformed	http://www.ncbi.nlm.nih.gov/projects/gap/cgi-bin/analysis.cgi?id=pha001096
ITBiomarkers	Hemostatic	PlateletAgCollex5mv	Platelet Aggregation to collagen, exam 5, multivariable, log transformed	http://www.ncbi.nlm.nih.gov/projects/gap/cgi-bin/analysis.cgi?id=pha001098
ITBiomarkers	Hemostatic	PlateletAgEpiex5as	Platelet Aggregation to Epinephrine at exam 5 age & sex, log transformed	http://www.ncbi.nlm.nih.gov/projects/gap/cgi-bin/analysis.cgi?id=pha001100

Group	Category	Trait Label	Name	GEE Link
ITBiomarkers	Hemostatic	PlateletAgEpiex5mv	Platelet Aggregation to Epinephrine exam 5 multivariable, log transformed	http://www.ncbi.nlm.nih.gov/projects/gap/cgi-bin/analysis.cgi?id=pha001102
ITBiomarkers	Hemostatic	tPAex5as	tPA Antigen at exam 5 age & sex, log transformed	http://www.ncbi.nlm.nih.gov/projects/gap/cgi-bin/analysis.cgi?id=pha001104
ITBiomarkers	Hemostatic	tPAex5mv	tPA Antigen at exam 5 multivariable, log transformed	http://www.ncbi.nlm.nih.gov/projects/gap/cgi-bin/analysis.cgi?id=pha001106
ITBiomarkers	Hemostatic	Viscoscityex5as	Viscosity I at exam 5 age & sex	http://www.ncbi.nlm.nih.gov/projects/gap/cgi-bin/analysis.cgi?id=pha001108
ITBiomarkers	Hemostatic	Viscoscityex5mv	Viscosity I at exam 5 multivariable	http://www.ncbi.nlm.nih.gov/projects/gap/cgi-bin/analysis.cgi?id=pha001110
ITBiomarkers	Hemostatic	wVWex5as	von Willebrand Factor at exam 5 age & sex	http://www.ncbi.nlm.nih.gov/projects/gap/cgi-bin/analysis.cgi?id=pha001112
ITBiomarkers	Hemostatic	wVWex5mv	von Willebrand Factor at exam 5 multivariable	http://www.ncbi.nlm.nih.gov/projects/gap/cgi-bin/analysis.cgi?id=pha001114
ITBiomarkers	Inflammation	CD40Lplasmaex7as	CD40Ligand plasma exam 7, age- & sex-adjusted, log transformed	http://www.ncbi.nlm.nih.gov/projects/gap/cgi-bin/analysis.cgi?id=pha001116
ITBiomarkers	Inflammation	CD40Lplasmaex7mv	CD40Ligand plasma exam 7 multivariable-adjusted, log transformed	http://www.ncbi.nlm.nih.gov/projects/gap/cgi-bin/analysis.cgi?id=pha001118
ITBiomarkers	Inflammation	CD40Lserumex7as	CD40Ligand serum exam 7, age- & sex-adjusted, log transformed	http://www.ncbi.nlm.nih.gov/projects/gap/cgi-bin/analysis.cgi?id=pha001120
ITBiomarkers	Inflammation	CD40Lserumex7mv	CD40Ligand serum, exam 7 multivariable-adjusted, log transformed	http://www.ncbi.nlm.nih.gov/projects/gap/cgi-bin/analysis.cgi?id=pha001122
ITBiomarkers	Inflammation	CRPavg267asB	Log C-reactive protein average exam 2, 6 & 7 age- & sex-adjusted	http://www.ncbi.nlm.nih.gov/projects/gap/cgi-bin/analysis.cgi?id=pha001172
ITBiomarkers	Inflammation	CRPavg267mvB	Log C-reactive protein average exam 2, 6 & 7, multivariable-adjusted	http://www.ncbi.nlm.nih.gov/projects/gap/cgi-bin/analysis.cgi?id=pha001174
ITBiomarkers	Inflammation	CRPex2asB	Log C-reactive protein exam 2, age- & sex-adjusted	http://www.ncbi.nlm.nih.gov/projects/gap/cgi-bin/analysis.cgi?id=pha001176
ITBiomarkers	Inflammation	CRPex2mvB	Log C-reactive protein exam 2, multivariable-adjusted	http://www.ncbi.nlm.nih.gov/projects/gap/cgi-bin/analysis.cgi?id=pha001178
ITBiomarkers	Inflammation	CRPex5as	Log C-reactive protein Hemagen Assay exam 5, age- & sex-adjusted	http://www.ncbi.nlm.nih.gov/projects/gap/cgi-bin/analysis.cgi?id=pha001124
ITBiomarkers	Inflammation	CRPex5mv	Log C-reactive protein Hemagen Assay exam 5, multivariable-adjusted	http://www.ncbi.nlm.nih.gov/projects/gap/cgi-bin/analysis.cgi?id=pha001126
ITBiomarkers	Inflammation	CRPex6as	Log C-reactive protein exam 6, age- & sex-adjusted	http://www.ncbi.nlm.nih.gov/projects/gap/cgi-bin/analysis.cgi?id=pha001128
ITBiomarkers	Inflammation	CRPex6mv	Log C-reactive protein exam 6, multivariable-adjusted	http://www.ncbi.nlm.nih.gov/projects/gap/cgi-bin/analysis.cgi?id=pha001130
ITBiomarkers	Inflammation	CRPex7as	Log C-reactive protein exam 7, age- & sex-adjusted	http://www.ncbi.nlm.nih.gov/projects/gap/cgi-bin/analysis.cgi?id=pha001132
ITBiomarkers	Inflammation	CRPex7mv	Log C-reactive protein exam 7, multivariable-adjusted	http://www.ncbi.nlm.nih.gov/projects/gap/cgi-bin/analysis.cgi?id=pha001134
ITBiomarkers	Inflammation	ICAM1ex7as	Log intercellular adhesion molecule-1 exam 7, age- & sex-adjusted	http://www.ncbi.nlm.nih.gov/projects/gap/cgi-bin/analysis.cgi?id=pha001136
ITBiomarkers	Inflammation	ICAM1ex7mv	Log intercellular adhesion molecule-1 exam 7, multivariable-adjusted	http://www.ncbi.nlm.nih.gov/projects/gap/cgi-bin/analysis.cgi?id=pha001138
ITBiomarkers	Inflammation	IL6ex7as	Log interleukin 6 Exam 7,age- & sex-adjusted	http://www.ncbi.nlm.nih.gov/projects/gap/cgi-bin/analysis.cgi?id=pha001140
ITBiomarkers	Inflammation	IL6ex7mv	Log interleukin 6 Exam 7,multivariable-adjusted	http://www.ncbi.nlm.nih.gov/projects/gap/cgi-bin/analysis.cgi?id=pha001142
ITBiomarkers	Inflammation	IsoCrUrinePex7as	Log urinary isoprostanes/creatinine exam 7, age- & sex-adjusted	http://www.ncbi.nlm.nih.gov/projects/gap/cgi-bin/analysis.cgi?id=pha001144
ITBiomarkers	Inflammation	IsoCrUrinePex7mv	Log urinary isoprostanes/creatinine exam 7, multivariable-adjusted	http://www.ncbi.nlm.nih.gov/projects/gap/cgi-bin/analysis.cgi?id=pha001146
ITBiomarkers	Inflammation	MCP1ex7as	Log monocyte chemoattractant protein 1 exam 7, age- & sex-adjusted	http://www.ncbi.nlm.nih.gov/projects/gap/cgi-bin/analysis.cgi?id=pha001148
ITBiomarkers	Inflammation	MCP1ex7mv	Log monocyte chemoattractant protein 1 exam 7, multivariable-adjusted	http://www.ncbi.nlm.nih.gov/projects/gap/cgi-bin/analysis.cgi?id=pha001150
ITBiomarkers	Inflammation	MPOex7as	Log myeloperoxidase exam 7, age- & sex-adjusted	http://www.ncbi.nlm.nih.gov/projects/gap/cgi-bin/analysis.cgi?id=pha001152
ITBiomarkers	Inflammation	MPOex7mv	Log myeloperoxidase exam 7, multivariable-adjusted	http://www.ncbi.nlm.nih.gov/projects/gap/cgi-bin/analysis.cgi?id=pha001154
ITBiomarkers	Inflammation	OPGex7as	Log osteoprotegerin exam 7, age- & sex-adjusted	http://www.ncbi.nlm.nih.gov/projects/gap/cgi-bin/analysis.cgi?id=pha001156
ITBiomarkers	Inflammation	OPGex7mv	Log osteoprotegerin exam 7, multivariable-adjusted	http://www.ncbi.nlm.nih.gov/projects/gap/cgi-bin/analysis.cgi?id=pha001158
ITBiomarkers	Inflammation	Pselectinex7as	Log P-selectin exam 7, age- & sex-adjusted	http://www.ncbi.nlm.nih.gov/projects/gap/cgi-bin/analysis.cgi?id=pha001160
ITBiomarkers	Inflammation	Pselectinex7mv	Log P-selectin exam 7, multivariable-adjusted	http://www.ncbi.nlm.nih.gov/projects/gap/cgi-bin/analysis.cgi?id=pha001162
ITBiomarkers	Inflammation	TNFAex7as	Log tumor necrosis factor alpha exam 7, age- & sex-adjusted	http://www.ncbi.nlm.nih.gov/projects/gap/cgi-bin/analysis.cgi?id=pha001164
ITBiomarkers	Inflammation	TNFAex7mv	Log tumor necrosis factor alpha exam 7, multivariable-adjusted	http://www.ncbi.nlm.nih.gov/projects/gap/cgi-bin/analysis.cgi?id=pha001166
ITBiomarkers	Inflammation	TNFRIIex7as	Log tumor necrosis factor receptor II exam 7, age- & sex-adjusted	http://www.ncbi.nlm.nih.gov/projects/gap/cgi-bin/analysis.cgi?id=pha001168
ITBiomarkers	Inflammation	TNFRIIex7mv	Log tumor necrosis factor receptor II exam 7 multivariable-adjusted	http://www.ncbi.nlm.nih.gov/projects/gap/cgi-bin/analysis.cgi?id=pha001170
ITBiomarkers	LFT	AlkPhosex2as	Log alkaline phosphatase exam 2 age- & sex-adjusted	http://www.ncbi.nlm.nih.gov/projects/gap/cgi-bin/analysis.cgi?id=pha001180
ITBiomarkers	LFT	AlkPhosex2mv	Log alkaline phosphatase exam 2, multivariable-adjusted	http://www.ncbi.nlm.nih.gov/projects/gap/cgi-bin/analysis.cgi?id=pha001182
ITBiomarkers	LFT	ALTex2as	Log alanine transaminase exam 2, age- & sex-adjusted	http://www.ncbi.nlm.nih.gov/projects/gap/cgi-bin/analysis.cgi?id=pha001184
ITBiomarkers	LFT	ALTex2mv	Log alanine transaminase exam 2; multivariable-adjusted	http://www.ncbi.nlm.nih.gov/projects/gap/cgi-bin/analysis.cgi?id=pha001186
ITBiomarkers	LFT	ASTex2as	Log aspartate aminotransferase exam, age- & sex-adjusted	http://www.ncbi.nlm.nih.gov/projects/gap/cgi-bin/analysis.cgi?id=pha001188
ITBiomarkers	LFT	ASTex2mv	Log aspartate aminotransferase exam 2 multivariable-adjusted	http://www.ncbi.nlm.nih.gov/projects/gap/cgi-bin/analysis.cgi?id=pha001190
ITBiomarkers	LFT	Bilirubinex2as	Log bilirubin exam 2, age- & sex-adjusted	http://www.ncbi.nlm.nih.gov/projects/gap/cgi-bin/analysis.cgi?id=pha001192
ITBiomarkers	LFT	Bilirubinex2mv	Log bilirubin exam 2 multivariable	http://www.ncbi.nlm.nih.gov/projects/gap/cgi-bin/analysis.cgi?id=pha001194
ITBiomarkers	LFT	GGTex2as	Log gamma-glutamyl transferase exam 2, age- & sex-adjusted	http://www.ncbi.nlm.nih.gov/projects/gap/cgi-bin/analysis.cgi?id=pha001196
ITBiomarkers	LFT	GGTex2mv	Log Gamma-glutamyl transferase exam 2, Multivariable,adjusted	http://www.ncbi.nlm.nih.gov/projects/gap/cgi-bin/analysis.cgi?id=pha001198
ITBiomarkers	Neurohumoral	ANPex6as	Log atrial natriuretic peptide exam 6, age- & sex-adjusted Tobit-model	http://www.ncbi.nlm.nih.gov/projects/gap/cgi-bin/analysis.cgi?id=pha001200
ITBiomarkers	Neurohumoral	ANPex6mv	Log atrial natriuretic peptide exam 6, multivariable-adjusted Tobit model	http://www.ncbi.nlm.nih.gov/projects/gap/cgi-bin/analysis.cgi?id=pha001202
ITBiomarkers	Neurohumoral	BNPex6as	Log brain natriuretic peptide exam 6, age- & sex-adjusted Tobit model	http://www.ncbi.nlm.nih.gov/projects/gap/cgi-bin/analysis.cgi?id=pha001204
ITBiomarkers	Neurohumoral	BNPex6mv	Log brain natriuretic peptide exam 6, multivariable-adjusted Tobit model	http://www.ncbi.nlm.nih.gov/projects/gap/cgi-bin/analysis.cgi?id=pha001206
ITBiomarkers	Vitamins	VitD25OHex6or7as	25(OH)-D exam 6 or 7, age & sex adjusted	http://www.ncbi.nlm.nih.gov/projects/gap/cgi-bin/analysis.cgi?id=pha001208
ITBiomarkers	Vitamins	VitD25OHex6or7mv	25(OH)-D exam 6 or 7, multivariable-adjusted; covariates from vit D exam	http://www.ncbi.nlm.nih.gov/projects/gap/cgi-bin/analysis.cgi?id=pha001210

Group	Category	Trait Label	Name	GEE Link
ITBiomarkers	Vitamins	VitKPhylloqex6or7as	Vit K exam 6 or 7, age & sex adjusted, log transformed, no warfarin	http://www.ncbi.nlm.nih.gov/projects/gap/cgi-bin/analysis.cgi?id=pha001212
ITBiomarkers	Vitamins	VitKPhylloqex6or7mv	Vit K exam 6 or 7, multivariable-adjusted, log transformed, no warfarin	http://www.ncbi.nlm.nih.gov/projects/gap/cgi-bin/analysis.cgi?id=pha001214
ITBiomarkers	Vitamins	VitKPucOCex6or7as	%ucOC, exam 6 or 7, age, sex adjust, log transformed; no warfarin	http://www.ncbi.nlm.nih.gov/projects/gap/cgi-bin/analysis.cgi?id=pha001216
ITBiomarkers	Vitamins	VitKPucOCex6or7mv	%ucOC, exam 6 or 7, multivariable, log-transformed, no warfarin use	http://www.ncbi.nlm.nih.gov/projects/gap/cgi-bin/analysis.cgi?id=pha001218
Metabolic	Adiposity	allbmi1602	adj bmi, offsp 2 & cohort 16 exams	http://www.ncbi.nlm.nih.gov/projects/gap/cgi-bin/analysis.cgi?id=pha000102
Metabolic	Adiposity	allbmi1803	adj bmi, offsp 3 & cohort 18 exams	http://www.ncbi.nlm.nih.gov/projects/gap/cgi-bin/analysis.cgi?id=pha000104
Metabolic	Adiposity	allbmi1x01	adj bmi, offsp 1 & cohort 10 exams	http://www.ncbi.nlm.nih.gov/projects/gap/cgi-bin/analysis.cgi?id=pha000106
Metabolic	Adiposity	allbmi2004	adj bmi, offsp 4 & cohort 20 exams	http://www.ncbi.nlm.nih.gov/projects/gap/cgi-bin/analysis.cgi?id=pha000108
Metabolic	Adiposity	allbmi2205	adj bmi, offsp 5 & cohort 22 exams	http://www.ncbi.nlm.nih.gov/projects/gap/cgi-bin/analysis.cgi?id=pha000110
Metabolic	Adiposity	allbmi2406	adj bmi, offsp 6 & cohort 24 exams	http://www.ncbi.nlm.nih.gov/projects/gap/cgi-bin/analysis.cgi?id=pha000112
Metabolic	Adiposity	allbmi2607	adj bmi, offsp 7 & cohort 26 exams	http://www.ncbi.nlm.nih.gov/projects/gap/cgi-bin/analysis.cgi?id=pha000114
Metabolic	Adiposity	allchgbmi	adj bmi change, offsp 1-7 & cohort exams 10-26	http://www.ncbi.nlm.nih.gov/projects/gap/cgi-bin/analysis.cgi?id=pha000116
Metabolic	Adiposity	allchgwgt	adj weight change, offsp 1-7 & cohort exams 10-26	http://www.ncbi.nlm.nih.gov/projects/gap/cgi-bin/analysis.cgi?id=pha000118
Metabolic	Adiposity	allhgt1602	adj height, offsp 2 & cohort 16 exams	http://www.ncbi.nlm.nih.gov/projects/gap/cgi-bin/analysis.cgi?id=pha000120
Metabolic	Adiposity	allhgt1803	adj height, offsp 3 & cohort 18 exams	http://www.ncbi.nlm.nih.gov/projects/gap/cgi-bin/analysis.cgi?id=pha000122
Metabolic	Adiposity	allhgt1x01	adj height, offsp 1 & cohort 10 exams	http://www.ncbi.nlm.nih.gov/projects/gap/cgi-bin/analysis.cgi?id=pha000124
Metabolic	Adiposity	allhgt2004	adj height, offsp 4 & cohort 20 exams	http://www.ncbi.nlm.nih.gov/projects/gap/cgi-bin/analysis.cgi?id=pha000126
Metabolic	Adiposity	allhgt2205	adj height, offsp 5 & cohort 22 exams	http://www.ncbi.nlm.nih.gov/projects/gap/cgi-bin/analysis.cgi?id=pha000128
Metabolic	Adiposity	allhgt2406	adj height, offsp 6 & cohort 24 exams	http://www.ncbi.nlm.nih.gov/projects/gap/cgi-bin/analysis.cgi?id=pha000130
Metabolic	Adiposity	allhgt2607	adj height, offsp 7 & cohort 26 exams	http://www.ncbi.nlm.nih.gov/projects/gap/cgi-bin/analysis.cgi?id=pha000132
Metabolic	Adiposity	allmeanbmi	adj mean bmi, offsp 1-7 & cohort 10,16,18,20,22,24,26 exams	http://www.ncbi.nlm.nih.gov/projects/gap/cgi-bin/analysis.cgi?id=pha000134
Metabolic	Adiposity	allmeanhgt	adj mean height, offsp 1-7 & cohort 10,16,18,20,22,24,26 exams	http://www.ncbi.nlm.nih.gov/projects/gap/cgi-bin/analysis.cgi?id=pha000136
Metabolic	Adiposity	allmeanwgt	adj mean weight, offsp 1-7 & cohort 10,16,18,20,22,24,26 exams	http://www.ncbi.nlm.nih.gov/projects/gap/cgi-bin/analysis.cgi?id=pha000138
Metabolic	Adiposity	allochgwst	adj waist change, offsp 4 to 7	http://www.ncbi.nlm.nih.gov/projects/gap/cgi-bin/analysis.cgi?id=pha000140
Metabolic	Adiposity	allomeanwst	adj mean waist, offsp 4 to 7	http://www.ncbi.nlm.nih.gov/projects/gap/cgi-bin/analysis.cgi?id=pha000142
Metabolic	Adiposity	allowst0007	adj waist, offsp 7	http://www.ncbi.nlm.nih.gov/projects/gap/cgi-bin/analysis.cgi?id=pha000144
Metabolic	Adiposity	allrankbmi1602	rank adj bmi, offsp 2 & cohort 16 exams	http://www.ncbi.nlm.nih.gov/projects/gap/cgi-bin/analysis.cgi?id=pha000146
Metabolic	Adiposity	allrankbmi1803	rank adj bmi, offsp 3 & cohort 18 exams	http://www.ncbi.nlm.nih.gov/projects/gap/cgi-bin/analysis.cgi?id=pha000148
Metabolic	Adiposity	allrankbmi1x01	rank adj bmi, offsp 1 & cohort 10 exams	http://www.ncbi.nlm.nih.gov/projects/gap/cgi-bin/analysis.cgi?id=pha000150
Metabolic	Adiposity	allrankbmi2004	rank adj bmi, offsp 4 & cohort 20 exams	http://www.ncbi.nlm.nih.gov/projects/gap/cgi-bin/analysis.cgi?id=pha000152
Metabolic	Adiposity	allrankbmi2205	rank adj bmi, offsp 5 & cohort 22 exams	http://www.ncbi.nlm.nih.gov/projects/gap/cgi-bin/analysis.cgi?id=pha000154
Metabolic	Adiposity	allrankbmi2406	rank adj bmi, offsp 6 & cohort 24 exams	http://www.ncbi.nlm.nih.gov/projects/gap/cgi-bin/analysis.cgi?id=pha000156
Metabolic	Adiposity	allrankbmi2607	rank adj bmi, offsp 7 & cohort 26 exams	http://www.ncbi.nlm.nih.gov/projects/gap/cgi-bin/analysis.cgi?id=pha000158
Metabolic	Adiposity	allrankchgbmi	rank adj bmi change, offsp 1-7 & cohort exams 10-26	http://www.ncbi.nlm.nih.gov/projects/gap/cgi-bin/analysis.cgi?id=pha000160
Metabolic	Adiposity	allrankchgwgt	rank adj weight change, offsp 1-7 & cohort exams 10-26	http://www.ncbi.nlm.nih.gov/projects/gap/cgi-bin/analysis.cgi?id=pha000162
Metabolic	Adiposity	allrankmeanbmi	rank adj mean bmi, offsp 1-7 & cohort 10,16,18,20,22,24,26 exams	http://www.ncbi.nlm.nih.gov/projects/gap/cgi-bin/analysis.cgi?id=pha000164
Metabolic	Adiposity	allrankmeanwgt	rank adj mean weight, offsp 1-7 & cohort 10,16,18,20,22,24,26 exams	http://www.ncbi.nlm.nih.gov/projects/gap/cgi-bin/analysis.cgi?id=pha000166
Metabolic	Adiposity	allrankochgwst	rank adj waist change, offsp 4 to 7	http://www.ncbi.nlm.nih.gov/projects/gap/cgi-bin/analysis.cgi?id=pha000168
Metabolic	Adiposity	allrankomeanwst	rank adj mean waist, offsp 4 to 7	http://www.ncbi.nlm.nih.gov/projects/gap/cgi-bin/analysis.cgi?id=pha000170
Metabolic	Adiposity	allrankwgt1602	rank adj weight, offsp 2 & cohort 16 exams	http://www.ncbi.nlm.nih.gov/projects/gap/cgi-bin/analysis.cgi?id=pha000172
Metabolic	Adiposity	allrankwgt1803	rank adj weight, offsp 3 & cohort 18 exams	http://www.ncbi.nlm.nih.gov/projects/gap/cgi-bin/analysis.cgi?id=pha000174
Metabolic	Adiposity	allrankwgt1x01	rank adj weight, offsp 1 & cohort 10 exams	http://www.ncbi.nlm.nih.gov/projects/gap/cgi-bin/analysis.cgi?id=pha000176
Metabolic	Adiposity	allrankwgt2004	rank adj weight, offsp 4 & cohort 20 exams	http://www.ncbi.nlm.nih.gov/projects/gap/cgi-bin/analysis.cgi?id=pha000178
Metabolic	Adiposity	allrankwgt2205	rank adj weight, offsp 5 & cohort 22 exams	http://www.ncbi.nlm.nih.gov/projects/gap/cgi-bin/analysis.cgi?id=pha000180
Metabolic	Adiposity	allrankwgt2406	rank adj weight, offsp 6 & cohort 24 exams	http://www.ncbi.nlm.nih.gov/projects/gap/cgi-bin/analysis.cgi?id=pha000182
Metabolic	Adiposity	allrankwgt2607	rank adj weight, offsp 7 & cohort 26 exams	http://www.ncbi.nlm.nih.gov/projects/gap/cgi-bin/analysis.cgi?id=pha000184
Metabolic	Adiposity	allrankwst2306	rank adj waist, offsp 6 & cohort exam 23	http://www.ncbi.nlm.nih.gov/projects/gap/cgi-bin/analysis.cgi?id=pha000186
Metabolic	Adiposity	allwgt1602	adj weight, offsp 2 & cohort 16 exams	http://www.ncbi.nlm.nih.gov/projects/gap/cgi-bin/analysis.cgi?id=pha000188
Metabolic	Adiposity	allwgt1803	adj weight, offsp 3 & cohort 18 exams	http://www.ncbi.nlm.nih.gov/projects/gap/cgi-bin/analysis.cgi?id=pha000190
Metabolic	Adiposity	allwgt1x01	adj weight, offsp 1 & cohort 10 exams	http://www.ncbi.nlm.nih.gov/projects/gap/cgi-bin/analysis.cgi?id=pha000192
Metabolic	Adiposity	allwgt2004	adj weight, offsp 4 & cohort 20 exams	http://www.ncbi.nlm.nih.gov/projects/gap/cgi-bin/analysis.cgi?id=pha000194
Metabolic	Adiposity	allwgt2205	adj weight, offsp 5 & cohort 22 exams	http://www.ncbi.nlm.nih.gov/projects/gap/cgi-bin/analysis.cgi?id=pha000196
Metabolic	Adiposity	allwgt2406	adj weight, offsp 6 & cohort 24 exams	http://www.ncbi.nlm.nih.gov/projects/gap/cgi-bin/analysis.cgi?id=pha000198
Metabolic	Adiposity	allwgt2607	adj weight, offsp 7 & cohort 26 exams	http://www.ncbi.nlm.nih.gov/projects/gap/cgi-bin/analysis.cgi?id=pha000200
Metabolic	Adiposity	allwst2004	adj waist, offsp 4 & cohort 20 exams	http://www.ncbi.nlm.nih.gov/projects/gap/cgi-bin/analysis.cgi?id=pha000202

Group	Category	Trait Label	Name	GEE Link
Metabolic	Adiposity	allwst2205	adj waist, offsp 5 & cohort 22 exams	http://www.ncbi.nlm.nih.gov/projects/gap/cgi-bin/analysis.cgi?id=pha000204
Metabolic	Adiposity	allwst2306	adj waist, offsp 6 & cohort exam 23	http://www.ncbi.nlm.nih.gov/projects/gap/cgi-bin/analysis.cgi?id=pha000206
Metabolic	Adiposity	fembmi1602	Female adj bmi, offsp 2 & cohort 16 exams	http://www.ncbi.nlm.nih.gov/projects/gap/cgi-bin/analysis.cgi?id=pha000208
Metabolic	Adiposity	fembmi1803	Female adj bmi, offsp 3 & cohort 18 exams	http://www.ncbi.nlm.nih.gov/projects/gap/cgi-bin/analysis.cgi?id=pha000210
Metabolic	Adiposity	fembmi1x01	Female adj bmi, offsp 1 & cohort 10 exams	http://www.ncbi.nlm.nih.gov/projects/gap/cgi-bin/analysis.cgi?id=pha000212
Metabolic	Adiposity	fembmi2004	Female adj bmi, offsp 4 & cohort 20 exams	http://www.ncbi.nlm.nih.gov/projects/gap/cgi-bin/analysis.cgi?id=pha000214
Metabolic	Adiposity	fembmi2205	Female adj bmi, offsp 5 & cohort 22 exams	http://www.ncbi.nlm.nih.gov/projects/gap/cgi-bin/analysis.cgi?id=pha000216
Metabolic	Adiposity	fembmi2406	Female adj bmi, offsp 6 & cohort 24 exams	http://www.ncbi.nlm.nih.gov/projects/gap/cgi-bin/analysis.cgi?id=pha000218
Metabolic	Adiposity	fembmi2607	Female adj bmi, offsp 7 & cohort 26 exams	http://www.ncbi.nlm.nih.gov/projects/gap/cgi-bin/analysis.cgi?id=pha000220
Metabolic	Adiposity	femchgbmi	Female adj bmi change, offsp 1-7 & cohort exams 10-26	http://www.ncbi.nlm.nih.gov/projects/gap/cgi-bin/analysis.cgi?id=pha000222
Metabolic	Adiposity	femchgwgt	Female adj weight change, offsp 1-7 & cohort exams 10-26	http://www.ncbi.nlm.nih.gov/projects/gap/cgi-bin/analysis.cgi?id=pha000224
Metabolic	Adiposity	femhgt1602	Female adj height, offsp 2 & cohort 16 exams	http://www.ncbi.nlm.nih.gov/projects/gap/cgi-bin/analysis.cgi?id=pha000226
Metabolic	Adiposity	femhgt1803	Female adj height, offsp 3 & cohort 18 exams	http://www.ncbi.nlm.nih.gov/projects/gap/cgi-bin/analysis.cgi?id=pha000228
Metabolic	Adiposity	femhgt1x01	Female adj height, offsp 1 & cohort 10 exams	http://www.ncbi.nlm.nih.gov/projects/gap/cgi-bin/analysis.cgi?id=pha000230
Metabolic	Adiposity	femhgt2004	Female adj height, offsp 4 & cohort 20 exams	http://www.ncbi.nlm.nih.gov/projects/gap/cgi-bin/analysis.cgi?id=pha000232
Metabolic	Adiposity	femhgt2205	Female adj height, offsp 5 & cohort 22 exams	http://www.ncbi.nlm.nih.gov/projects/gap/cgi-bin/analysis.cgi?id=pha000234
Metabolic	Adiposity	femhgt2406	Female adj height, offsp 6 & cohort 24 exams	http://www.ncbi.nlm.nih.gov/projects/gap/cgi-bin/analysis.cgi?id=pha000236
Metabolic	Adiposity	femhgt2607	Female adj height, offsp 7 & cohort 26 exams	http://www.ncbi.nlm.nih.gov/projects/gap/cgi-bin/analysis.cgi?id=pha000238
Metabolic	Adiposity	femmeanbmi	Female adj mean bmi, offsp 1-7 & cohort 10,16,18,20,22,24,26 exams	http://www.ncbi.nlm.nih.gov/projects/gap/cgi-bin/analysis.cgi?id=pha000240
Metabolic	Adiposity	femmeanhgt	Female adj mean height, offsp 1-7 & cohort 10,16,18,20,22,24,26 exams	http://www.ncbi.nlm.nih.gov/projects/gap/cgi-bin/analysis.cgi?id=pha000242
Metabolic	Adiposity	femmeanwgt	Female adj mean weight, offsp 1-7 & cohort 10,16,18,20,22,24,26 exams	http://www.ncbi.nlm.nih.gov/projects/gap/cgi-bin/analysis.cgi?id=pha000244
Metabolic	Adiposity	femochgwst	Female adj waist change, offsp 4 to 7	http://www.ncbi.nlm.nih.gov/projects/gap/cgi-bin/analysis.cgi?id=pha000246
Metabolic	Adiposity	femomeanwst	Female adj mean waist, offsp 4 to 7	http://www.ncbi.nlm.nih.gov/projects/gap/cgi-bin/analysis.cgi?id=pha000248
Metabolic	Adiposity	femowst0007	Female adj waist, offsp 7	http://www.ncbi.nlm.nih.gov/projects/gap/cgi-bin/analysis.cgi?id=pha000250
Metabolic	Adiposity	femrankbmi1602	Female rank adj bmi, offsp 2 & cohort 16 exams	http://www.ncbi.nlm.nih.gov/projects/gap/cgi-bin/analysis.cgi?id=pha000252
Metabolic	Adiposity	femrankbmi1803	Female rank adj bmi, offsp 3 & cohort 18 exams	http://www.ncbi.nlm.nih.gov/projects/gap/cgi-bin/analysis.cgi?id=pha000254
Metabolic	Adiposity	femrankbmi1x01	Female rank adj bmi, offsp 1 & cohort 10 exams	http://www.ncbi.nlm.nih.gov/projects/gap/cgi-bin/analysis.cgi?id=pha000256
Metabolic	Adiposity	femrankbmi2004	Female rank adj bmi, offsp 4 & cohort 20 exams	http://www.ncbi.nlm.nih.gov/projects/gap/cgi-bin/analysis.cgi?id=pha000258
Metabolic	Adiposity	femrankbmi2205	Female rank adj bmi, offsp 5 & cohort 22 exams	http://www.ncbi.nlm.nih.gov/projects/gap/cgi-bin/analysis.cgi?id=pha000260
Metabolic	Adiposity	femrankbmi2406	Female rank adj bmi, offsp 6 & cohort 24 exams	http://www.ncbi.nlm.nih.gov/projects/gap/cgi-bin/analysis.cgi?id=pha000262
Metabolic	Adiposity	femrankbmi2607	Female rank adj bmi, offsp 7 & cohort 26 exams	http://www.ncbi.nlm.nih.gov/projects/gap/cgi-bin/analysis.cgi?id=pha000264
Metabolic	Adiposity	femrankchgwgt	Female rank adj weight change, offsp 1-7 & cohort exams 10-26	http://www.ncbi.nlm.nih.gov/projects/gap/cgi-bin/analysis.cgi?id=pha000266
Metabolic	Adiposity	femrankmeanbmi	Female rank adj mean bmi, offsp 1-7 & cohort 10,16,18,20,22,24,26 exams	http://www.ncbi.nlm.nih.gov/projects/gap/cgi-bin/analysis.cgi?id=pha000268
Metabolic	Adiposity	femrankmeanwgt	Female rank adj mean weight, offsp 1-7 & cohort 10,16,18,20,22,24,26 exams	http://www.ncbi.nlm.nih.gov/projects/gap/cgi-bin/analysis.cgi?id=pha000270
Metabolic	Adiposity	femrankomeanwst	Female rank adj mean waist, offsp 4 to 7	http://www.ncbi.nlm.nih.gov/projects/gap/cgi-bin/analysis.cgi?id=pha000272
Metabolic	Adiposity	femrankwgt1602	Female rank adj weight, offsp 2 & cohort 16 exams	http://www.ncbi.nlm.nih.gov/projects/gap/cgi-bin/analysis.cgi?id=pha000274
Metabolic	Adiposity	femrankwgt1803	Female rank adj weight, offsp 3 & cohort 18 exams	http://www.ncbi.nlm.nih.gov/projects/gap/cgi-bin/analysis.cgi?id=pha000276
Metabolic	Adiposity	femrankwgt1x01	Female rank adj weight, offsp 1 & cohort 10 exams	http://www.ncbi.nlm.nih.gov/projects/gap/cgi-bin/analysis.cgi?id=pha000278
Metabolic	Adiposity	femrankwgt2004	Female rank adj weight, offsp 4 & cohort 20 exams	http://www.ncbi.nlm.nih.gov/projects/gap/cgi-bin/analysis.cgi?id=pha000280
Metabolic	Adiposity	femrankwgt2205	Female rank adj weight, offsp 5 & cohort 22 exams	http://www.ncbi.nlm.nih.gov/projects/gap/cgi-bin/analysis.cgi?id=pha000282
Metabolic	Adiposity	femrankwgt2406	Female rank adj weight, offsp 6 & cohort 24 exams	http://www.ncbi.nlm.nih.gov/projects/gap/cgi-bin/analysis.cgi?id=pha000284
Metabolic	Adiposity	femrankwgt2607	Female rank adj weight, offsp 7 & cohort 26 exams	http://www.ncbi.nlm.nih.gov/projects/gap/cgi-bin/analysis.cgi?id=pha000286
Metabolic	Adiposity	femwgt1602	Female adj weight, offsp 2 & cohort 16 exams	http://www.ncbi.nlm.nih.gov/projects/gap/cgi-bin/analysis.cgi?id=pha000288
Metabolic	Adiposity	femwgt1803	Female adj weight, offsp 3 & cohort 18 exams	http://www.ncbi.nlm.nih.gov/projects/gap/cgi-bin/analysis.cgi?id=pha000290
Metabolic	Adiposity	femwgt1x01	Female adj weight, offsp 1 & cohort 10 exams	http://www.ncbi.nlm.nih.gov/projects/gap/cgi-bin/analysis.cgi?id=pha000292
Metabolic	Adiposity	femwgt2004	Female adj weight, offsp 4 & cohort 20 exams	http://www.ncbi.nlm.nih.gov/projects/gap/cgi-bin/analysis.cgi?id=pha000294
Metabolic	Adiposity	femwgt2205	Female adj weight, offsp 5 & cohort 22 exams	http://www.ncbi.nlm.nih.gov/projects/gap/cgi-bin/analysis.cgi?id=pha000296
Metabolic	Adiposity	femwgt2406	Female adj weight, offsp 6 & cohort 24 exams	http://www.ncbi.nlm.nih.gov/projects/gap/cgi-bin/analysis.cgi?id=pha000298
Metabolic	Adiposity	femwgt2607	Female adj weight, offsp 7 & cohort 26 exams	http://www.ncbi.nlm.nih.gov/projects/gap/cgi-bin/analysis.cgi?id=pha000300
Metabolic	Adiposity	femwst2004	Female adj waist, offsp 4 & cohort 20 exams	http://www.ncbi.nlm.nih.gov/projects/gap/cgi-bin/analysis.cgi?id=pha000302
Metabolic	Adiposity	femwst2205	Female adj waist, offsp 5 & cohort 22 exams	http://www.ncbi.nlm.nih.gov/projects/gap/cgi-bin/analysis.cgi?id=pha000304
Metabolic	Adiposity	femwst2306	Female adj waist, offsp 6 & cohort exam 23	http://www.ncbi.nlm.nih.gov/projects/gap/cgi-bin/analysis.cgi?id=pha000306
Metabolic	Adiposity	malebmi1602	Male adj bmi, offsp 2 & cohort 16 exams	http://www.ncbi.nlm.nih.gov/projects/gap/cgi-bin/analysis.cgi?id=pha000308
Metabolic	Adiposity	malebmi1803	Male adj bmi, offsp 3 & cohort 18 exams	http://www.ncbi.nlm.nih.gov/projects/gap/cgi-bin/analysis.cgi?id=pha000310
Metabolic	Adiposity	malebmi1x01	Male adj bmi, offsp 1 & cohort 10 exams	http://www.ncbi.nlm.nih.gov/projects/gap/cgi-bin/analysis.cgi?id=pha000312

Group	Category	Trait Label	Name	GEE Link
Metabolic	Adiposity	malebmi2004	Male adj bmi, offsp 4 & cohort 20 exams	http://www.ncbi.nlm.nih.gov/projects/gap/cgi-bin/analysis.cgi?id=pha000314
Metabolic	Adiposity	malebmi2205	Male adj bmi, offsp 5 & cohort 22 exams	http://www.ncbi.nlm.nih.gov/projects/gap/cgi-bin/analysis.cgi?id=pha000316
Metabolic	Adiposity	malebmi2406	Male adj bmi, offsp 6 & cohort 24 exams	http://www.ncbi.nlm.nih.gov/projects/gap/cgi-bin/analysis.cgi?id=pha000318
Metabolic	Adiposity	malebmi2607	Male adj bmi, offsp 7 & cohort 26 exams	http://www.ncbi.nlm.nih.gov/projects/gap/cgi-bin/analysis.cgi?id=pha000320
Metabolic	Adiposity	malechgbmi	Male adj bmi change, offsp 1-7 & cohort exams 10-26	http://www.ncbi.nlm.nih.gov/projects/gap/cgi-bin/analysis.cgi?id=pha000322
Metabolic	Adiposity	malechgwgt	Male adj weight change, offsp 1-7 & cohort exams 10-26	http://www.ncbi.nlm.nih.gov/projects/gap/cgi-bin/analysis.cgi?id=pha000324
Metabolic	Adiposity	malehgt1602	Male adj height, offsp 2 & cohort 16 exams	http://www.ncbi.nlm.nih.gov/projects/gap/cgi-bin/analysis.cgi?id=pha000326
Metabolic	Adiposity	malehgt1803	Male adj height, offsp 3 & cohort 18 exams	http://www.ncbi.nlm.nih.gov/projects/gap/cgi-bin/analysis.cgi?id=pha000328
Metabolic	Adiposity	malehgt1x01	Male adj height, offsp 1 & cohort 10 exams	http://www.ncbi.nlm.nih.gov/projects/gap/cgi-bin/analysis.cgi?id=pha000330
Metabolic	Adiposity	malehgt2004	Male adj height, offsp 4 & cohort 20 exams	http://www.ncbi.nlm.nih.gov/projects/gap/cgi-bin/analysis.cgi?id=pha000332
Metabolic	Adiposity	malehgt2205	Male adj height, offsp 5 & cohort 22 exams	http://www.ncbi.nlm.nih.gov/projects/gap/cgi-bin/analysis.cgi?id=pha000334
Metabolic	Adiposity	malehgt2406	Male adj height, offsp 6 & cohort 24 exams	http://www.ncbi.nlm.nih.gov/projects/gap/cgi-bin/analysis.cgi?id=pha000336
Metabolic	Adiposity	malehgt2607	Male adj height, offsp 7 & cohort 26 exams	http://www.ncbi.nlm.nih.gov/projects/gap/cgi-bin/analysis.cgi?id=pha000338
Metabolic	Adiposity	malemeanbmi	Male adj mean bmi, offsp 1-7 & cohort 10,16,18,20,22,24,26 exams	http://www.ncbi.nlm.nih.gov/projects/gap/cgi-bin/analysis.cgi?id=pha000340
Metabolic	Adiposity	malemeanhgt	Male adj mean hgt, offsp 1-7 & cohort 10,16,18,20,22,24,26 exams	http://www.ncbi.nlm.nih.gov/projects/gap/cgi-bin/analysis.cgi?id=pha000342
Metabolic	Adiposity	malemeanwgt	Male adj mean weight, offsp 1-7 & cohort 10,16,18,20,22,24,26 exams	http://www.ncbi.nlm.nih.gov/projects/gap/cgi-bin/analysis.cgi?id=pha000344
Metabolic	Adiposity	maleochgwst	Male adj waist change, offsp 4 to 7	http://www.ncbi.nlm.nih.gov/projects/gap/cgi-bin/analysis.cgi?id=pha000346
Metabolic	Adiposity	maleomeanwst	Male adj mean waist, offsp 4 to 7	http://www.ncbi.nlm.nih.gov/projects/gap/cgi-bin/analysis.cgi?id=pha000348
Metabolic	Adiposity	maleowst0007	Male adj waist, offsp 7	http://www.ncbi.nlm.nih.gov/projects/gap/cgi-bin/analysis.cgi?id=pha000350
Metabolic	Adiposity	malerankbmi1602	Male rank adj bmi, offsp 2 & cohort 16 exams	http://www.ncbi.nlm.nih.gov/projects/gap/cgi-bin/analysis.cgi?id=pha000352
Metabolic	Adiposity	malerankbmi1803	Male rank adj bmi, offsp 3 & cohort 18 exams	http://www.ncbi.nlm.nih.gov/projects/gap/cgi-bin/analysis.cgi?id=pha000354
Metabolic	Adiposity	malerankbmi2004	Male rank adj bmi, offsp 4 & cohort 20 exams	http://www.ncbi.nlm.nih.gov/projects/gap/cgi-bin/analysis.cgi?id=pha000356
Metabolic	Adiposity	malerankbmi2205	Male rank adj bmi, offsp 5 & cohort 22 exams	http://www.ncbi.nlm.nih.gov/projects/gap/cgi-bin/analysis.cgi?id=pha000358
Metabolic	Adiposity	malerankbmi2406	Male rank adj bmi, offsp 6 & cohort 24 exams	http://www.ncbi.nlm.nih.gov/projects/gap/cgi-bin/analysis.cgi?id=pha000360
Metabolic	Adiposity	malerankbmi2607	Male rank adj bmi, offsp 7 & cohort 26 exams	http://www.ncbi.nlm.nih.gov/projects/gap/cgi-bin/analysis.cgi?id=pha000362
Metabolic	Adiposity	malerankchgbmi	Male rank adj bmi change, offsp 1-7 & cohort exams 10-26	http://www.ncbi.nlm.nih.gov/projects/gap/cgi-bin/analysis.cgi?id=pha000364
Metabolic	Adiposity	malerankchgwgt	Male rank adj weight change, offsp 1-7 & cohort exams 10-26	http://www.ncbi.nlm.nih.gov/projects/gap/cgi-bin/analysis.cgi?id=pha000366
Metabolic	Adiposity	malerankhgt2406	Male rank adj height, offsp 6 & cohort 24 exams	http://www.ncbi.nlm.nih.gov/projects/gap/cgi-bin/analysis.cgi?id=pha000368
Metabolic	Adiposity	malerankochgwst	Male rank adj waist change, offsp 4 to 7	http://www.ncbi.nlm.nih.gov/projects/gap/cgi-bin/analysis.cgi?id=pha000370
Metabolic	Adiposity	malerankwgt1602	Male rank adj weight, offsp 2 & cohort 16 exams	http://www.ncbi.nlm.nih.gov/projects/gap/cgi-bin/analysis.cgi?id=pha000372
Metabolic	Adiposity	malerankwgt2004	Male rank adj weight, offsp 4 & cohort 20 exams	http://www.ncbi.nlm.nih.gov/projects/gap/cgi-bin/analysis.cgi?id=pha000374
Metabolic	Adiposity	malerankwgt2205	Male rank adj weight, offsp 5 & cohort 22 exams	http://www.ncbi.nlm.nih.gov/projects/gap/cgi-bin/analysis.cgi?id=pha000376
Metabolic	Adiposity	malerankwst2306	Male rank adj waist, offsp 6 & cohort exam 23	http://www.ncbi.nlm.nih.gov/projects/gap/cgi-bin/analysis.cgi?id=pha000378
Metabolic	Adiposity	malewgt1602	Male adj weight, offsp 2 & cohort 16 exams	http://www.ncbi.nlm.nih.gov/projects/gap/cgi-bin/analysis.cgi?id=pha000380
Metabolic	Adiposity	malewgt1803	Male adj weight, offsp 3 & cohort 18 exams	http://www.ncbi.nlm.nih.gov/projects/gap/cgi-bin/analysis.cgi?id=pha000382
Metabolic	Adiposity	malewgt1x01	Male adj weight, offsp 1 & cohort 10 exams	http://www.ncbi.nlm.nih.gov/projects/gap/cgi-bin/analysis.cgi?id=pha000384
Metabolic	Adiposity	malewgt2004	Male adj weight, offsp 4 & cohort 20 exams	http://www.ncbi.nlm.nih.gov/projects/gap/cgi-bin/analysis.cgi?id=pha000386
Metabolic	Adiposity	malewgt2205	Male adj weight, offsp 5 & cohort 22 exams	http://www.ncbi.nlm.nih.gov/projects/gap/cgi-bin/analysis.cgi?id=pha000388
Metabolic	Adiposity	malewgt2406	Male adj weight, offsp 6 & cohort 24 exams	http://www.ncbi.nlm.nih.gov/projects/gap/cgi-bin/analysis.cgi?id=pha000390
Metabolic	Adiposity	malewgt2607	Male adj weight, offsp 7 & cohort 26 exams	http://www.ncbi.nlm.nih.gov/projects/gap/cgi-bin/analysis.cgi?id=pha000392
Metabolic	Adiposity	malewst2004	Male adj waist, offsp 4 & cohort 20 exams	http://www.ncbi.nlm.nih.gov/projects/gap/cgi-bin/analysis.cgi?id=pha000394
Metabolic	Adiposity	malewst2205	Male adj waist, offsp 5 & cohort 22 exams	http://www.ncbi.nlm.nih.gov/projects/gap/cgi-bin/analysis.cgi?id=pha000396
Metabolic	Adiposity	malewst2306	Male adj waist, offsp 6 & cohort exam 23	http://www.ncbi.nlm.nih.gov/projects/gap/cgi-bin/analysis.cgi?id=pha000398
Metabolic	Adiposity	SATAS	SAT adj for age, age sqr, sex	http://www.ncbi.nlm.nih.gov/projects/gap/cgi-bin/analysis.cgi?id=pha000400
Metabolic	Adiposity	SATMV	SAT adj for age, age square, sex, meno status, smoking	http://www.ncbi.nlm.nih.gov/projects/gap/cgi-bin/analysis.cgi?id=pha000402
Metabolic	Adiposity	SDAS	Sagittal diameter adj for age, age square, sex	http://www.ncbi.nlm.nih.gov/projects/gap/cgi-bin/analysis.cgi?id=pha000404
Metabolic	Adiposity	SDMV	Sagittal diameter adj for age, age square, sex, meno status, smoking	http://www.ncbi.nlm.nih.gov/projects/gap/cgi-bin/analysis.cgi?id=pha000406
Metabolic	Adiposity	VATAS	VAT adj for age, age squared, sex	http://www.ncbi.nlm.nih.gov/projects/gap/cgi-bin/analysis.cgi?id=pha000408
Metabolic	Adiposity	VATMV	VAT adj for age, age squared, sex, meno status, smoking	http://www.ncbi.nlm.nih.gov/projects/gap/cgi-bin/analysis.cgi?id=pha000410
Metabolic	Adiposity	WCAS	Waist by CT adj for age, age squared, sex	http://www.ncbi.nlm.nih.gov/projects/gap/cgi-bin/analysis.cgi?id=pha000412
Metabolic	Adiposity	WCMV	Waist by CT adj for age, age squared, sex, meno status, smoking	http://www.ncbi.nlm.nih.gov/projects/gap/cgi-bin/analysis.cgi?id=pha000414
Metabolic	Glycemic	DiabSurv1sexage	Diabetes incidence to ex7, age-sex adjusted in FBAT, Cox model for GEE	http://www.ncbi.nlm.nih.gov/projects/gap/cgi-bin/analysis.cgi?id=pha000416
Metabolic	Glycemic	DiabSurv2sexagebmi	Diabetes incidence to ex7, age-sex-bmi adjusted in FBAT, Cox model for GEE	http://www.ncbi.nlm.nih.gov/projects/gap/cgi-bin/analysis.cgi?id=pha000418
Metabolic	Glycemic	res1ladipoq7o	Age-sex adjusted Adiponectin ex7 offspring	http://www.ncbi.nlm.nih.gov/projects/gap/cgi-bin/analysis.cgi?id=pha000420
Metabolic	Glycemic	res1lfglu5o	Age-sex adjusted Fasting Plasma Glucose ex5 offspring	http://www.ncbi.nlm.nih.gov/projects/gap/cgi-bin/analysis.cgi?id=pha000422

Group	Category	Trait Label	Name	GEE Link
Metabolic	Glycemic	res1lfglu7o	Age-sex adjusted Fasting Plasma Glucose ex7 offspring	http://www.ncbi.nlm.nih.gov/projects/gap/cgi-bin/analysis.cgi?id=pha000424
Metabolic	Glycemic	res1lfins5o	Age-sex adjusted Fasting Insulin ex5 offspring	http://www.ncbi.nlm.nih.gov/projects/gap/cgi-bin/analysis.cgi?id=pha000426
Metabolic	Glycemic	res1lfins7o	Age-sex adjusted Fasting Insulin ex7 offspring	http://www.ncbi.nlm.nih.gov/projects/gap/cgi-bin/analysis.cgi?id=pha000428
Metabolic	Glycemic	res1lgutt5o	Age-sex adjusted Insulin Sensitivity ex5 offspring	http://www.ncbi.nlm.nih.gov/projects/gap/cgi-bin/analysis.cgi?id=pha000430
Metabolic	Glycemic	res1lhba1c5o	Age-sex adjusted Fasting HbA1c ex5 offspring	http://www.ncbi.nlm.nih.gov/projects/gap/cgi-bin/analysis.cgi?id=pha000432
Metabolic	Glycemic	res1lhba1c7o	Age-sex adjusted HbA1c ex7 offspring	http://www.ncbi.nlm.nih.gov/projects/gap/cgi-bin/analysis.cgi?id=pha000434
Metabolic	Glycemic	res1lhir5o	Age-sex adjusted HOMA-IR ex5 offspring	http://www.ncbi.nlm.nih.gov/projects/gap/cgi-bin/analysis.cgi?id=pha000436
Metabolic	Glycemic	res1lhir7o	Age-sex adjusted HOMA-IR ex7 offspring	http://www.ncbi.nlm.nih.gov/projects/gap/cgi-bin/analysis.cgi?id=pha000438
Metabolic	Glycemic	res1lmfglu17o	Age-sex adjusted Mean Plasma Glucose ex1-7 offspring	http://www.ncbi.nlm.nih.gov/projects/gap/cgi-bin/analysis.cgi?id=pha000440
Metabolic	Glycemic	res1lretn7o	Age-sex adjusted Resistin ex7 offspring	http://www.ncbi.nlm.nih.gov/projects/gap/cgi-bin/analysis.cgi?id=pha000442
Metabolic	Glycemic	res2ladipoq7o	Multivariable adjusted Adiponectin ex7 offspring	http://www.ncbi.nlm.nih.gov/projects/gap/cgi-bin/analysis.cgi?id=pha000444
Metabolic	Glycemic	res2lfglu5o	Multivariable adjusted Fasting Plasma Glucose ex5 offspring	http://www.ncbi.nlm.nih.gov/projects/gap/cgi-bin/analysis.cgi?id=pha000446
Metabolic	Glycemic	res2lfglu7o	Multivariable adjusted Fasting Plasma Glucose ex7 offspring	http://www.ncbi.nlm.nih.gov/projects/gap/cgi-bin/analysis.cgi?id=pha000448
Metabolic	Glycemic	res2lfins5o	Multivariable adjusted Fasting Insulin ex5 offspring	http://www.ncbi.nlm.nih.gov/projects/gap/cgi-bin/analysis.cgi?id=pha000450
Metabolic	Glycemic	res2lfins7o	Multivariable adjusted Fasting Insulin ex7 offspring	http://www.ncbi.nlm.nih.gov/projects/gap/cgi-bin/analysis.cgi?id=pha000452
Metabolic	Glycemic	res2lgutt5o	Multivariable adjusted Insulin Sensitivity ex5 offspring	http://www.ncbi.nlm.nih.gov/projects/gap/cgi-bin/analysis.cgi?id=pha000454
Metabolic	Glycemic	res2lhba1c5o	Multivariable adjusted Fasting HbA1c ex5 offspring	http://www.ncbi.nlm.nih.gov/projects/gap/cgi-bin/analysis.cgi?id=pha000456
Metabolic	Glycemic	res2lhba1c7o	Multivariable adjusted HbA1c ex7 offspring	http://www.ncbi.nlm.nih.gov/projects/gap/cgi-bin/analysis.cgi?id=pha000458
Metabolic	Glycemic	res2lhir5o	Multivariable adjusted HOMA-IR ex5 offspring	http://www.ncbi.nlm.nih.gov/projects/gap/cgi-bin/analysis.cgi?id=pha000460
Metabolic	Glycemic	res2lhir7o	Multivariable adjusted HOMA-IR ex7 offspring	http://www.ncbi.nlm.nih.gov/projects/gap/cgi-bin/analysis.cgi?id=pha000462
Metabolic	Glycemic	res2lmfglu17o	Multivariable adjusted Mean Plasma Glucose exam1-7 offspring	http://www.ncbi.nlm.nih.gov/projects/gap/cgi-bin/analysis.cgi?id=pha000464
Metabolic	Glycemic	res2lretn7o	Multivariable adjusted Resistin ex7 offspring	http://www.ncbi.nlm.nih.gov/projects/gap/cgi-bin/analysis.cgi?id=pha000466
Metabolic	Lipids	ApoA14a	Multivariable adj Plasma ApoA1 Level Offsp ex4	http://www.ncbi.nlm.nih.gov/projects/gap/cgi-bin/analysis.cgi?id=pha000468
Metabolic	Lipids	ApoA14b	Age-sex adj Plasma ApoA1 Level Offsp ex4	http://www.ncbi.nlm.nih.gov/projects/gap/cgi-bin/analysis.cgi?id=pha000470
Metabolic	Lipids	ApoB4a	Multivariable adj Plasma ApoB Level Offsp ex4	http://www.ncbi.nlm.nih.gov/projects/gap/cgi-bin/analysis.cgi?id=pha000472
Metabolic	Lipids	ApoB4b	Age-sex adj Plasma ApoB Level Offsp ex4	http://www.ncbi.nlm.nih.gov/projects/gap/cgi-bin/analysis.cgi?id=pha000474
Metabolic	Lipids	ApoC3a	Multivariable adj Plasma ApoCIII Level Offsp ex5	http://www.ncbi.nlm.nih.gov/projects/gap/cgi-bin/analysis.cgi?id=pha000476
Metabolic	Lipids	ApoC3b	Age-sex adj Plasma ApoCIII Level Offsp ex5	http://www.ncbi.nlm.nih.gov/projects/gap/cgi-bin/analysis.cgi?id=pha000478
Metabolic	Lipids	chol1a	Multivariable adj Chol Offsp ex1	http://www.ncbi.nlm.nih.gov/projects/gap/cgi-bin/analysis.cgi?id=pha000480
Metabolic	Lipids	chol1b	Age-sex adj Chol Offsp ex1	http://www.ncbi.nlm.nih.gov/projects/gap/cgi-bin/analysis.cgi?id=pha000482
Metabolic	Lipids	chol2b	Age-sex adj Chol Offsp ex2	http://www.ncbi.nlm.nih.gov/projects/gap/cgi-bin/analysis.cgi?id=pha000484
Metabolic	Lipids	chol3b	Age-sex adj Chol Offsp ex3	http://www.ncbi.nlm.nih.gov/projects/gap/cgi-bin/analysis.cgi?id=pha000486
Metabolic	Lipids	chol4b	Age-sex adj Chol Offsp ex4	http://www.ncbi.nlm.nih.gov/projects/gap/cgi-bin/analysis.cgi?id=pha000488
Metabolic	Lipids	chol5b	Age-sex adj Chol Offsp ex5	http://www.ncbi.nlm.nih.gov/projects/gap/cgi-bin/analysis.cgi?id=pha000490
Metabolic	Lipids	chol6b	Age-sex adj Chol Offsp ex6	http://www.ncbi.nlm.nih.gov/projects/gap/cgi-bin/analysis.cgi?id=pha000492
Metabolic	Lipids	chol7b	Age-sex adj Chol Offsp ex7	http://www.ncbi.nlm.nih.gov/projects/gap/cgi-bin/analysis.cgi?id=pha000494
Metabolic	Lipids	cholhdl1a	Multvariable adj Chol-HDL Ratio Offsp ex1	http://www.ncbi.nlm.nih.gov/projects/gap/cgi-bin/analysis.cgi?id=pha000496
Metabolic	Lipids	cholhdl1b	Age-sex adj Chol-HDL Ratio Offsp ex1	http://www.ncbi.nlm.nih.gov/projects/gap/cgi-bin/analysis.cgi?id=pha000498
Metabolic	Lipids	cholhdl2b	Age-sex adj Chol-HDL Ratio Offsp ex2	http://www.ncbi.nlm.nih.gov/projects/gap/cgi-bin/analysis.cgi?id=pha000500
Metabolic	Lipids	cholhdl3b	Age-sex adj Chol-HDL Ratio Offsp ex3	http://www.ncbi.nlm.nih.gov/projects/gap/cgi-bin/analysis.cgi?id=pha000502
Metabolic	Lipids	cholhdl4b	Age-sex adj Chol-HDL Ratio Offsp ex4	http://www.ncbi.nlm.nih.gov/projects/gap/cgi-bin/analysis.cgi?id=pha000504
Metabolic	Lipids	cholhdl5b	Age-sex adj Chol-HDL Ratio Offsp ex5	http://www.ncbi.nlm.nih.gov/projects/gap/cgi-bin/analysis.cgi?id=pha000506
Metabolic	Lipids	cholhdl6b	Age-sex adj Chol-HDL Ratio Offsp ex6	http://www.ncbi.nlm.nih.gov/projects/gap/cgi-bin/analysis.cgi?id=pha000508
Metabolic	Lipids	cholhdl7b	Age-sex adj Chol-HDL Ratio Offsp ex7	http://www.ncbi.nlm.nih.gov/projects/gap/cgi-bin/analysis.cgi?id=pha000510
Metabolic	Lipids	hdl1a	Multivariable adj HDL Offsp ex1	http://www.ncbi.nlm.nih.gov/projects/gap/cgi-bin/analysis.cgi?id=pha000512
Metabolic	Lipids	hdl1b	Age-sex adj HDL Offsp ex1	http://www.ncbi.nlm.nih.gov/projects/gap/cgi-bin/analysis.cgi?id=pha000514
Metabolic	Lipids	hdl24a	Multivariable adj HDL2 Offsp ex4	http://www.ncbi.nlm.nih.gov/projects/gap/cgi-bin/analysis.cgi?id=pha000516
Metabolic	Lipids	hdl24b	Age-sex adj HDL2 Offsp ex4	http://www.ncbi.nlm.nih.gov/projects/gap/cgi-bin/analysis.cgi?id=pha000518
Metabolic	Lipids	hdl25a	Multivariable adj HDL2 Offsp ex5	http://www.ncbi.nlm.nih.gov/projects/gap/cgi-bin/analysis.cgi?id=pha000520
Metabolic	Lipids	hdl25b	Age-sex adj HDL2 Offsp ex5	http://www.ncbi.nlm.nih.gov/projects/gap/cgi-bin/analysis.cgi?id=pha000522
Metabolic	Lipids	hdl2b	Age-sex adj HDL Offsp ex2	http://www.ncbi.nlm.nih.gov/projects/gap/cgi-bin/analysis.cgi?id=pha000524
Metabolic	Lipids	hdl34a	Multivariable adj HDL3 Offsp ex4	http://www.ncbi.nlm.nih.gov/projects/gap/cgi-bin/analysis.cgi?id=pha000526
Metabolic	Lipids	hdl34b	Age-sex adj HDL3 Offsp ex4	http://www.ncbi.nlm.nih.gov/projects/gap/cgi-bin/analysis.cgi?id=pha000528
Metabolic	Lipids	hdl35a	Multivariable adj HDL3 Offsp ex5	http://www.ncbi.nlm.nih.gov/projects/gap/cgi-bin/analysis.cgi?id=pha000530
Metabolic	Lipids	hdl35b	Age-sex adj HDL3 Offsp ex5	http://www.ncbi.nlm.nih.gov/projects/gap/cgi-bin/analysis.cgi?id=pha000532

Group	Category	Trait Label	Name	GEE Link
Metabolic	Lipids	hdl3b	Age-sex adj HDL Offsp ex3	http://www.ncbi.nlm.nih.gov/projects/gap/cgi-bin/analysis.cgi?id=pha000534
Metabolic	Lipids	hdl4b	Age-sex adj HDL Offsp ex4	http://www.ncbi.nlm.nih.gov/projects/gap/cgi-bin/analysis.cgi?id=pha000536
Metabolic	Lipids	hdl5b	Age-sex adj HDL Offsp ex5	http://www.ncbi.nlm.nih.gov/projects/gap/cgi-bin/analysis.cgi?id=pha000538
Metabolic	Lipids	hdl6b	Age-sex adj HDL Offsp ex6	http://www.ncbi.nlm.nih.gov/projects/gap/cgi-bin/analysis.cgi?id=pha000540
Metabolic	Lipids	hdl7b	Age-sex adj HDL Offsp ex7	http://www.ncbi.nlm.nih.gov/projects/gap/cgi-bin/analysis.cgi?id=pha000542
Metabolic	Lipids	HDLNMRint4a	Multivariable adj NMR HDL Int Offsp ex4 or TG > 400	http://www.ncbi.nlm.nih.gov/projects/gap/cgi-bin/analysis.cgi?id=pha000544
Metabolic	Lipids	HDLNMRint4b	Age-sex adj NMR HDL Int Offsp ex4 or TG > 400	http://www.ncbi.nlm.nih.gov/projects/gap/cgi-bin/analysis.cgi?id=pha000546
Metabolic	Lipids	HDLNMRlg4a	Multivariable adj NMR HDL Lg Offsp ex4 or TG > 400	http://www.ncbi.nlm.nih.gov/projects/gap/cgi-bin/analysis.cgi?id=pha000548
Metabolic	Lipids	HDLNMRlg4b	Age-sex adj NMR HDL Lg Offsp ex4 or TG > 400	http://www.ncbi.nlm.nih.gov/projects/gap/cgi-bin/analysis.cgi?id=pha000550
Metabolic	Lipids	HDLNMRsm4a	Multivariable adj NMR HDL Sm Offsp ex4 or TG > 400	http://www.ncbi.nlm.nih.gov/projects/gap/cgi-bin/analysis.cgi?id=pha000552
Metabolic	Lipids	HDLNMRsm4b	Age-sex adj NMR HDL Sm Offsp ex4 or TG > 400	http://www.ncbi.nlm.nih.gov/projects/gap/cgi-bin/analysis.cgi?id=pha000554
Metabolic	Lipids	HDLNMRsz4a	Multivariable adj NMR HDL Size Offsp ex4 or TG > 400	http://www.ncbi.nlm.nih.gov/projects/gap/cgi-bin/analysis.cgi?id=pha000556
Metabolic	Lipids	HDLNMRsz4b	Age-sex adj NMR HDL Size Offsp ex4 or TG > 400	http://www.ncbi.nlm.nih.gov/projects/gap/cgi-bin/analysis.cgi?id=pha000558
Metabolic	Lipids	IDLNMR4a	Multivariable adj NMR IDL Offsp ex4 or TG > 400	http://www.ncbi.nlm.nih.gov/projects/gap/cgi-bin/analysis.cgi?id=pha000560
Metabolic	Lipids	IDLNMR4b	Age-sex adj NMR IDL Offsp ex4 or TG > 400	http://www.ncbi.nlm.nih.gov/projects/gap/cgi-bin/analysis.cgi?id=pha000562
Metabolic	Lipids	ldl1a	Multivariable adj LDL Offsp ex1	http://www.ncbi.nlm.nih.gov/projects/gap/cgi-bin/analysis.cgi?id=pha000564
Metabolic	Lipids	ldl1b	Age-sex adj LDL Offsp ex1	http://www.ncbi.nlm.nih.gov/projects/gap/cgi-bin/analysis.cgi?id=pha000566
Metabolic	Lipids	ldl2b	Age-sex adj LDL Offsp ex2	http://www.ncbi.nlm.nih.gov/projects/gap/cgi-bin/analysis.cgi?id=pha000568
Metabolic	Lipids	ldl3b	Age-sex adj LDL Offsp ex3	http://www.ncbi.nlm.nih.gov/projects/gap/cgi-bin/analysis.cgi?id=pha000570
Metabolic	Lipids	ldl4b	Age-sex adj LDL Offsp ex4	http://www.ncbi.nlm.nih.gov/projects/gap/cgi-bin/analysis.cgi?id=pha000572
Metabolic	Lipids	ldl5b	Age-sex adj LDL Offsp ex5	http://www.ncbi.nlm.nih.gov/projects/gap/cgi-bin/analysis.cgi?id=pha000574
Metabolic	Lipids	ldl6b	Age-sex adj LDL Offsp ex6	http://www.ncbi.nlm.nih.gov/projects/gap/cgi-bin/analysis.cgi?id=pha000576
Metabolic	Lipids	ldl7b	Age-sex adj LDL Offsp ex7	http://www.ncbi.nlm.nih.gov/projects/gap/cgi-bin/analysis.cgi?id=pha000578
Metabolic	Lipids	LDLNMRlg4a	Multivariable adj NMR LDL Lg Offsp ex4 or TG > 400	http://www.ncbi.nlm.nih.gov/projects/gap/cgi-bin/analysis.cgi?id=pha000580
Metabolic	Lipids	LDLNMRlg4b	Age-sex adj NMR LDL Lg Offsp ex4 or TG > 400	http://www.ncbi.nlm.nih.gov/projects/gap/cgi-bin/analysis.cgi?id=pha000582
Metabolic	Lipids	LDLNMRsm4a	Multivariable adj NMR LDL Sm Offsp ex4 or TG > 400	http://www.ncbi.nlm.nih.gov/projects/gap/cgi-bin/analysis.cgi?id=pha000584
Metabolic	Lipids	LDLNMRsm4b	Age-sex adj NMR LDL Sm Offsp ex4 or TG > 400	http://www.ncbi.nlm.nih.gov/projects/gap/cgi-bin/analysis.cgi?id=pha000586
Metabolic	Lipids	LDLNMRsz4a	Multivariable adj NMR LDL Size Offsp ex4 or TG > 400	http://www.ncbi.nlm.nih.gov/projects/gap/cgi-bin/analysis.cgi?id=pha000588
Metabolic	Lipids	LDLNMRsz4b	Age-sex adj NMR LDL Size Offsp ex4 or TG > 400	http://www.ncbi.nlm.nih.gov/projects/gap/cgi-bin/analysis.cgi?id=pha000590
Metabolic	Lipids	lpa3a	Multivariable adj Lpa Offsp ex3	http://www.ncbi.nlm.nih.gov/projects/gap/cgi-bin/analysis.cgi?id=pha000592
Metabolic	Lipids	lpa3b	Age-sex adj Lpa Offsp ex3	http://www.ncbi.nlm.nih.gov/projects/gap/cgi-bin/analysis.cgi?id=pha000594
Metabolic	Lipids	meanchola	Multivariable adj NMR Chol Offsp ex1-7	http://www.ncbi.nlm.nih.gov/projects/gap/cgi-bin/analysis.cgi?id=pha000596
Metabolic	Lipids	mean cholb	Age-sex adj NMR Chol Offsp ex1-7	http://www.ncbi.nlm.nih.gov/projects/gap/cgi-bin/analysis.cgi?id=pha000598
Metabolic	Lipids	meanhdla	Multivariable adj NMR HDL Offsp ex1-7	http://www.ncbi.nlm.nih.gov/projects/gap/cgi-bin/analysis.cgi?id=pha000600
Metabolic	Lipids	meanhdlb	Age-sex adj NMR HDL Offsp ex1-7	http://www.ncbi.nlm.nih.gov/projects/gap/cgi-bin/analysis.cgi?id=pha000602
Metabolic	Lipids	meanldla	Multivariable adj NMR Calc LDL Offsp ex1-7	http://www.ncbi.nlm.nih.gov/projects/gap/cgi-bin/analysis.cgi?id=pha000604
Metabolic	Lipids	meanldlb	Age-sex adj NMR Calc LDL Offsp ex1-7	http://www.ncbi.nlm.nih.gov/projects/gap/cgi-bin/analysis.cgi?id=pha000606
Metabolic	Lipids	meantga	Multivariable adj NMR (TG) Offsp ex1-7	http://www.ncbi.nlm.nih.gov/projects/gap/cgi-bin/analysis.cgi?id=pha000608
Metabolic	Lipids	meantgb	Age-sex adj NMR (TG) Offsp ex1-7	http://www.ncbi.nlm.nih.gov/projects/gap/cgi-bin/analysis.cgi?id=pha000610
Metabolic	Lipids	PlasmaApoEa	Multivariable adj Plasma ApoE Level Offsp ex5	http://www.ncbi.nlm.nih.gov/projects/gap/cgi-bin/analysis.cgi?id=pha000612
Metabolic	Lipids	PlasmaApoEb	Age-sex adj Plasma ApoE Level Offsp ex5	http://www.ncbi.nlm.nih.gov/projects/gap/cgi-bin/analysis.cgi?id=pha000614
Metabolic	Lipids	RLPChol4a	Multivariable adj Remnant LP Chol Offsp ex4	http://www.ncbi.nlm.nih.gov/projects/gap/cgi-bin/analysis.cgi?id=pha000924
Metabolic	Lipids	RLPChol4b	Age-sex adj Remnant LP Chol Offsp ex4	http://www.ncbi.nlm.nih.gov/projects/gap/cgi-bin/analysis.cgi?id=pha000926
Metabolic	Lipids	RLPTG4a	Remnant LP TG Offsp ex4	http://www.ncbi.nlm.nih.gov/projects/gap/cgi-bin/analysis.cgi?id=pha000618
Metabolic	Lipids	RLPTG4b	Remnant LP Age-sex adj TG Offsp ex4	http://www.ncbi.nlm.nih.gov/projects/gap/cgi-bin/analysis.cgi?id=pha000616
Metabolic	Lipids	tg1a	Multivariable adj TG Offsp ex1	http://www.ncbi.nlm.nih.gov/projects/gap/cgi-bin/analysis.cgi?id=pha000620
Metabolic	Lipids	tg1b	Age-sex adj TG Offsp ex1	http://www.ncbi.nlm.nih.gov/projects/gap/cgi-bin/analysis.cgi?id=pha000622
Metabolic	Lipids	tg2b	Age-sex adj TG Offsp ex2	http://www.ncbi.nlm.nih.gov/projects/gap/cgi-bin/analysis.cgi?id=pha000624
Metabolic	Lipids	tg3b	Age-sex adj TG Offsp ex3	http://www.ncbi.nlm.nih.gov/projects/gap/cgi-bin/analysis.cgi?id=pha000626
Metabolic	Lipids	tg4b	Age-sex adj TG Offsp ex4	http://www.ncbi.nlm.nih.gov/projects/gap/cgi-bin/analysis.cgi?id=pha000628
Metabolic	Lipids	tg5b	Age-sex adj TG Offsp ex5	http://www.ncbi.nlm.nih.gov/projects/gap/cgi-bin/analysis.cgi?id=pha000630
Metabolic	Lipids	tg6b	Age-sex adj TG Offsp ex6	http://www.ncbi.nlm.nih.gov/projects/gap/cgi-bin/analysis.cgi?id=pha000632
Metabolic	Lipids	tg7b	Age-sex adj TG Offsp ex7	http://www.ncbi.nlm.nih.gov/projects/gap/cgi-bin/analysis.cgi?id=pha000634
Metabolic	Lipids	tghdl1a	Multivariable adj TG-HDL Ratio Offsp ex1	http://www.ncbi.nlm.nih.gov/projects/gap/cgi-bin/analysis.cgi?id=pha000668
Metabolic	Lipids	tghdl1b	Age-sex adj TG-HDL Ratio Offsp ex1	http://www.ncbi.nlm.nih.gov/projects/gap/cgi-bin/analysis.cgi?id=pha000914

Group	Category	Trait Label	Name	GEE Link
Metabolic	Lipids	tghdl2b	Age-sex adj TG-HDL Ratio Offsp ex2	http://www.ncbi.nlm.nih.gov/projects/gap/cgi-bin/analysis.cgi?id=pha000670
Metabolic	Lipids	tghdl3b	Age-sex adj TG-HDL Ratio Offsp ex3	http://www.ncbi.nlm.nih.gov/projects/gap/cgi-bin/analysis.cgi?id=pha000672
Metabolic	Lipids	tghdl4b	Age-sex adj TG-HDL Ratio Offsp ex4	http://www.ncbi.nlm.nih.gov/projects/gap/cgi-bin/analysis.cgi?id=pha000674
Metabolic	Lipids	tghdl5b	Age-sex adj TG-HDL Ratio Offsp ex5	http://www.ncbi.nlm.nih.gov/projects/gap/cgi-bin/analysis.cgi?id=pha000676
Metabolic	Lipids	tghdl6b	Age-sex adj TG-HDL Ratio Offsp ex6	http://www.ncbi.nlm.nih.gov/projects/gap/cgi-bin/analysis.cgi?id=pha000678
Metabolic	Lipids	tghdl7b	Age-sex adj TG-HDL Ratio Offsp ex7	http://www.ncbi.nlm.nih.gov/projects/gap/cgi-bin/analysis.cgi?id=pha000680
Metabolic	Lipids	VLDLNMRint4a	Multivariable adj NMR VLDL Int Offsp ex4	http://www.ncbi.nlm.nih.gov/projects/gap/cgi-bin/analysis.cgi?id=pha000866
Metabolic	Lipids	VLDLNMRint4b	Age-sex adj NMR VLDL Int Offsp ex4	http://www.ncbi.nlm.nih.gov/projects/gap/cgi-bin/analysis.cgi?id=pha000868
Metabolic	Lipids	VLDLNMRlg4a	Multivariable adj NMR VLDL Lg Offsp ex4	http://www.ncbi.nlm.nih.gov/projects/gap/cgi-bin/analysis.cgi?id=pha000878
Metabolic	Lipids	VLDLNMRlg4b	Age-sex adj NMR VLDL Lg Offsp ex4	http://www.ncbi.nlm.nih.gov/projects/gap/cgi-bin/analysis.cgi?id=pha000880
Metabolic	Lipids	VLDLNMRsm4a	Multivariable adj NMR VLDL Sm Offsp ex4	http://www.ncbi.nlm.nih.gov/projects/gap/cgi-bin/analysis.cgi?id=pha000902
Metabolic	Lipids	VLDLNMRsm4b	Age-sex adj NMR VLDL Sm Offsp ex4	http://www.ncbi.nlm.nih.gov/projects/gap/cgi-bin/analysis.cgi?id=pha000904
Metabolic	Lipids	VLDLNMRsz4a	Multivariable adj NMR VLDL Size Offsp ex4	http://www.ncbi.nlm.nih.gov/projects/gap/cgi-bin/analysis.cgi?id=pha000890
Metabolic	Lipids	VLDLNMRsz4b	Age-sex adj NMR VLDL Size Offsp ex4	http://www.ncbi.nlm.nih.gov/projects/gap/cgi-bin/analysis.cgi?id=pha000892
Metabolic	Lipidsmen	menApoB4a	Multivariable adj Plasma ApoB Level Offsp men ex4	http://www.ncbi.nlm.nih.gov/projects/gap/cgi-bin/analysis.cgi?id=pha000916
Metabolic	Lipidsmen	menApoB4b	Age-sex adj Plasma ApoB Level Offsp men ex4	http://www.ncbi.nlm.nih.gov/projects/gap/cgi-bin/analysis.cgi?id=pha000918
Metabolic	Lipidsmen	menchol1a	Multivariable adj Chol Offsp men ex1	http://www.ncbi.nlm.nih.gov/projects/gap/cgi-bin/analysis.cgi?id=pha000710
Metabolic	Lipidsmen	menchol1b	Age-sex adj Chol Offsp men ex1	http://www.ncbi.nlm.nih.gov/projects/gap/cgi-bin/analysis.cgi?id=pha000712
Metabolic	Lipidsmen	menchol2b	Age-sex adj Chol Offsp men ex2	http://www.ncbi.nlm.nih.gov/projects/gap/cgi-bin/analysis.cgi?id=pha000714
Metabolic	Lipidsmen	menchol3b	Age-sex adj Chol Offsp men ex3	http://www.ncbi.nlm.nih.gov/projects/gap/cgi-bin/analysis.cgi?id=pha000716
Metabolic	Lipidsmen	menchol4b	Age-sex adj Chol Offsp men ex4	http://www.ncbi.nlm.nih.gov/projects/gap/cgi-bin/analysis.cgi?id=pha000718
Metabolic	Lipidsmen	menchol5b	Age-sex adj Chol Offsp men ex5	http://www.ncbi.nlm.nih.gov/projects/gap/cgi-bin/analysis.cgi?id=pha000720
Metabolic	Lipidsmen	menchol6b	Age-sex adj Chol Offsp men ex6	http://www.ncbi.nlm.nih.gov/projects/gap/cgi-bin/analysis.cgi?id=pha000722
Metabolic	Lipidsmen	menchol7b	Age-sex adj Chol Offsp men ex7	http://www.ncbi.nlm.nih.gov/projects/gap/cgi-bin/analysis.cgi?id=pha000724
Metabolic	Lipidsmen	menhdl1a	Multivariable adj HDL Offsp men ex1	http://www.ncbi.nlm.nih.gov/projects/gap/cgi-bin/analysis.cgi?id=pha000742
Metabolic	Lipidsmen	menhdl1b	Age-sex adj HDL Offsp men ex1	http://www.ncbi.nlm.nih.gov/projects/gap/cgi-bin/analysis.cgi?id=pha000744
Metabolic	Lipidsmen	menhdl2b	Age-sex adj HDL Offsp men ex2	http://www.ncbi.nlm.nih.gov/projects/gap/cgi-bin/analysis.cgi?id=pha000746
Metabolic	Lipidsmen	menhdl3b	Age-sex adj HDL Offsp men ex3	http://www.ncbi.nlm.nih.gov/projects/gap/cgi-bin/analysis.cgi?id=pha000748
Metabolic	Lipidsmen	menhdl4b	Age-sex adj HDL Offsp men ex4	http://www.ncbi.nlm.nih.gov/projects/gap/cgi-bin/analysis.cgi?id=pha000750
Metabolic	Lipidsmen	menhdl5b	Age-sex adj HDL Offsp men ex5	http://www.ncbi.nlm.nih.gov/projects/gap/cgi-bin/analysis.cgi?id=pha000752
Metabolic	Lipidsmen	menhdl6b	Age-sex adj HDL Offsp men ex6	http://www.ncbi.nlm.nih.gov/projects/gap/cgi-bin/analysis.cgi?id=pha000754
Metabolic	Lipidsmen	menhdl7b	Age-sex adj HDL Offsp men ex7	http://www.ncbi.nlm.nih.gov/projects/gap/cgi-bin/analysis.cgi?id=pha000756
Metabolic	Lipidsmen	menHDLNMRint4a	Multivariable adj NMR HDL Int Offsp men ex4	http://www.ncbi.nlm.nih.gov/projects/gap/cgi-bin/analysis.cgi?id=pha000810
Metabolic	Lipidsmen	menHDLNMRint4b	Age-sex adj NMR HDL Int Offsp men ex4	http://www.ncbi.nlm.nih.gov/projects/gap/cgi-bin/analysis.cgi?id=pha000812
Metabolic	Lipidsmen	menHDLNMRlg4a	Multivariable adj NMR HDL Lg Offsp men ex4	http://www.ncbi.nlm.nih.gov/projects/gap/cgi-bin/analysis.cgi?id=pha000818
Metabolic	Lipidsmen	menHDLNMRlg4b	Age-sex adj NMR HDL Lg Offsp men ex4	http://www.ncbi.nlm.nih.gov/projects/gap/cgi-bin/analysis.cgi?id=pha000820
Metabolic	Lipidsmen	menHDLNMRsm4a	Multivariable adj NMR HDL Sm Offsp men ex4	http://www.ncbi.nlm.nih.gov/projects/gap/cgi-bin/analysis.cgi?id=pha000834
Metabolic	Lipidsmen	menHDLNMRsm4b	Age-sex adj NMR HDL Sm Offsp men ex4	http://www.ncbi.nlm.nih.gov/projects/gap/cgi-bin/analysis.cgi?id=pha000836
Metabolic	Lipidsmen	menHDLNMRsz4a	Multivariable adj NMR HDL Size Offsp men ex4	http://www.ncbi.nlm.nih.gov/projects/gap/cgi-bin/analysis.cgi?id=pha000826
Metabolic	Lipidsmen	menHDLNMRsz4b	Age-sex adj NMR HDL Size Offsp men ex4	http://www.ncbi.nlm.nih.gov/projects/gap/cgi-bin/analysis.cgi?id=pha000828
Metabolic	Lipidsmen	menldl1a	Multivariable adj LDL Offsp men ex1	http://www.ncbi.nlm.nih.gov/projects/gap/cgi-bin/analysis.cgi?id=pha000682
Metabolic	Lipidsmen	menldl1b	Age-sex adj LDL Offsp men ex1	http://www.ncbi.nlm.nih.gov/projects/gap/cgi-bin/analysis.cgi?id=pha000774
Metabolic	Lipidsmen	menldl2b	Age-sex adj LDL Offsp men ex2	http://www.ncbi.nlm.nih.gov/projects/gap/cgi-bin/analysis.cgi?id=pha000684
Metabolic	Lipidsmen	menldl3b	Age-sex adj LDL Offsp men ex3	http://www.ncbi.nlm.nih.gov/projects/gap/cgi-bin/analysis.cgi?id=pha000686
Metabolic	Lipidsmen	menldl4b	Age-sex adj LDL Offsp men ex4	http://www.ncbi.nlm.nih.gov/projects/gap/cgi-bin/analysis.cgi?id=pha000688
Metabolic	Lipidsmen	menldl5b	Age-sex adj LDL Offsp men ex5	http://www.ncbi.nlm.nih.gov/projects/gap/cgi-bin/analysis.cgi?id=pha000690
Metabolic	Lipidsmen	menldl6b	Age-sex adj LDL Offsp men ex6	http://www.ncbi.nlm.nih.gov/projects/gap/cgi-bin/analysis.cgi?id=pha000692
Metabolic	Lipidsmen	menldl7b	Age-sex adj LDL Offsp men ex7	http://www.ncbi.nlm.nih.gov/projects/gap/cgi-bin/analysis.cgi?id=pha000694
Metabolic	Lipidsmen	menLDLNMRlg4a	Multivariable adj NMR LDL Lg Offsp men ex4	http://www.ncbi.nlm.nih.gov/projects/gap/cgi-bin/analysis.cgi?id=pha000842
Metabolic	Lipidsmen	menLDLNMRlg4b	Age-sex adj NMR LDL Lg Offsp men ex4	http://www.ncbi.nlm.nih.gov/projects/gap/cgi-bin/analysis.cgi?id=pha000844
Metabolic	Lipidsmen	menLDLNMRsm4a	Multivariable adj NMR LDL Sm Offsp men ex4	http://www.ncbi.nlm.nih.gov/projects/gap/cgi-bin/analysis.cgi?id=pha000858
Metabolic	Lipidsmen	menLDLNMRsm4b	Age-sex adj NMR LDL Sm Offsp men ex4	http://www.ncbi.nlm.nih.gov/projects/gap/cgi-bin/analysis.cgi?id=pha000860
Metabolic	Lipidsmen	menLDLNMRsz4a	Multivariable adj NMR LDL Size Offsp men ex4	http://www.ncbi.nlm.nih.gov/projects/gap/cgi-bin/analysis.cgi?id=pha000850
Metabolic	Lipidsmen	menLDLNMRsz4b	Age-sex adj NMR LDL Size Offsp men ex4	http://www.ncbi.nlm.nih.gov/projects/gap/cgi-bin/analysis.cgi?id=pha000852
Metabolic	Lipidsmen	menmeanchola	Multivariable adj Mean Chol Offsp men ex1-7	http://www.ncbi.nlm.nih.gov/projects/gap/cgi-bin/analysis.cgi?id=pha000794

Group	Category	Trait Label	Name	GEE Link
Metabolic	Lipidsmen	menmeancholb	Age-sex adj Mean Chol Offsp men ex1-7	http://www.ncbi.nlm.nih.gov/projects/gap/cgi-bin/analysis.cgi?id=pha000796
Metabolic	Lipidsmen	menmeanhdla	Multivariable adj Mean HDL Offsp men ex1-7	http://www.ncbi.nlm.nih.gov/projects/gap/cgi-bin/analysis.cgi?id=pha000802
Metabolic	Lipidsmen	menmeanhdlb	Age-sex adj Mean HDL Offsp men ex1-7	http://www.ncbi.nlm.nih.gov/projects/gap/cgi-bin/analysis.cgi?id=pha000804
Metabolic	Lipidsmen	menmeanldla	Multivariable adj Mean LDL Offsp men ex1-7	http://www.ncbi.nlm.nih.gov/projects/gap/cgi-bin/analysis.cgi?id=pha000786
Metabolic	Lipidsmen	menmeanldlb	Age-sex adj Mean LDL Offsp men ex1-7	http://www.ncbi.nlm.nih.gov/projects/gap/cgi-bin/analysis.cgi?id=pha000788
Metabolic	Lipidsmen	menmeantga	Multivariable adj Mean TG Offsp men ex1-7	http://www.ncbi.nlm.nih.gov/projects/gap/cgi-bin/analysis.cgi?id=pha000778
Metabolic	Lipidsmen	menmeantgb	Age-sex adj Mean TG Offsp men ex1-7	http://www.ncbi.nlm.nih.gov/projects/gap/cgi-bin/analysis.cgi?id=pha000780
Metabolic	Lipidsmen	mentg1a	Multivariable adj TG Offsp men ex1	http://www.ncbi.nlm.nih.gov/projects/gap/cgi-bin/analysis.cgi?id=pha000636
Metabolic	Lipidsmen	mentg1b	Age-sex adj TG Offsp men ex1	http://www.ncbi.nlm.nih.gov/projects/gap/cgi-bin/analysis.cgi?id=pha000638
Metabolic	Lipidsmen	mentg2b	Age-sex adj TG Offsp men ex2	http://www.ncbi.nlm.nih.gov/projects/gap/cgi-bin/analysis.cgi?id=pha000640
Metabolic	Lipidsmen	mentg3b	Age-sex adj TG Offsp men ex3	http://www.ncbi.nlm.nih.gov/projects/gap/cgi-bin/analysis.cgi?id=pha000642
Metabolic	Lipidsmen	mentg4b	Age-sex adj TG Offsp men ex4	http://www.ncbi.nlm.nih.gov/projects/gap/cgi-bin/analysis.cgi?id=pha000644
Metabolic	Lipidsmen	mentg5b	Age-sex adj TG Offsp men ex5	http://www.ncbi.nlm.nih.gov/projects/gap/cgi-bin/analysis.cgi?id=pha000646
Metabolic	Lipidsmen	mentg6b	Age-sex adj TG Offsp men ex6	http://www.ncbi.nlm.nih.gov/projects/gap/cgi-bin/analysis.cgi?id=pha000648
Metabolic	Lipidsmen	mentg7b	Age-sex adj TG Offsp men ex7	http://www.ncbi.nlm.nih.gov/projects/gap/cgi-bin/analysis.cgi?id=pha000650
Metabolic	Lipidsmen	menVLDLNMRint4a	Multivariable adj NMR VLDL Int Offsp men ex4	http://www.ncbi.nlm.nih.gov/projects/gap/cgi-bin/analysis.cgi?id=pha000870
Metabolic	Lipidsmen	menVLDLNMRint4b	Age-sex adj NMR VLDL Int Offsp men ex4	http://www.ncbi.nlm.nih.gov/projects/gap/cgi-bin/analysis.cgi?id=pha000872
Metabolic	Lipidsmen	menVLDLNMRlg4a	Multivariable adj NMR VLDL Lg Offsp men ex4	http://www.ncbi.nlm.nih.gov/projects/gap/cgi-bin/analysis.cgi?id=pha000882
Metabolic	Lipidsmen	menVLDLNMRlg4b	Age-sex adj NMR VLDL Lg Offsp men ex4	http://www.ncbi.nlm.nih.gov/projects/gap/cgi-bin/analysis.cgi?id=pha000884
Metabolic	Lipidsmen	menVLDLNMRsm4a	Multivariable adj NMR VLDL Sm Offsp men ex4	http://www.ncbi.nlm.nih.gov/projects/gap/cgi-bin/analysis.cgi?id=pha000906
Metabolic	Lipidsmen	menVLDLNMRsm4b	Age-sex adj NMR VLDL Sm Offsp men ex4	http://www.ncbi.nlm.nih.gov/projects/gap/cgi-bin/analysis.cgi?id=pha000908
Metabolic	Lipidsmen	menVLDLNMRsz4a	Multivariable adj NMR VLDL Size Offsp men ex4	http://www.ncbi.nlm.nih.gov/projects/gap/cgi-bin/analysis.cgi?id=pha000894
Metabolic	Lipidsmen	menVLDLNMRsz4b	Age-sex adj NMR VLDL Size Offsp men ex4	http://www.ncbi.nlm.nih.gov/projects/gap/cgi-bin/analysis.cgi?id=pha000896
Metabolic	Lipidswomen	womenApoB4a	Multivariable adj Plasma ApoB Level Offsp wom ex4	http://www.ncbi.nlm.nih.gov/projects/gap/cgi-bin/analysis.cgi?id=pha000920
Metabolic	Lipidswomen	womenApoB4b	Age-sex adj Plasma ApoB Level Offsp wom ex4	http://www.ncbi.nlm.nih.gov/projects/gap/cgi-bin/analysis.cgi?id=pha000922
Metabolic	Lipidswomen	womenchol1a	Multivariable adj Chol Offsp wom ex1	http://www.ncbi.nlm.nih.gov/projects/gap/cgi-bin/analysis.cgi?id=pha000726
Metabolic	Lipidswomen	womenchol1b	Age-sex adj Chol Offsp wom ex1	http://www.ncbi.nlm.nih.gov/projects/gap/cgi-bin/analysis.cgi?id=pha000728
Metabolic	Lipidswomen	womenchol2b	Age-sex adj Chol Offsp wom ex2	http://www.ncbi.nlm.nih.gov/projects/gap/cgi-bin/analysis.cgi?id=pha000730
Metabolic	Lipidswomen	womenchol3b	Age-sex adj Chol Offsp wom ex3	http://www.ncbi.nlm.nih.gov/projects/gap/cgi-bin/analysis.cgi?id=pha000732
Metabolic	Lipidswomen	womenchol4b	Age-sex adj Chol Offsp wom ex4	http://www.ncbi.nlm.nih.gov/projects/gap/cgi-bin/analysis.cgi?id=pha000734
Metabolic	Lipidswomen	womenchol5b	Age-sex adj Chol Offsp wom ex5	http://www.ncbi.nlm.nih.gov/projects/gap/cgi-bin/analysis.cgi?id=pha000736
Metabolic	Lipidswomen	womenchol6b	Age-sex adj Chol Offsp wom ex6	http://www.ncbi.nlm.nih.gov/projects/gap/cgi-bin/analysis.cgi?id=pha000738
Metabolic	Lipidswomen	womenchol7b	Age-sex adj Chol Offsp wom ex7	http://www.ncbi.nlm.nih.gov/projects/gap/cgi-bin/analysis.cgi?id=pha000740
Metabolic	Lipidswomen	womenhdl1a	Multivariable adj HDL Offsp wom ex1	http://www.ncbi.nlm.nih.gov/projects/gap/cgi-bin/analysis.cgi?id=pha000758
Metabolic	Lipidswomen	womenhdl1b	Age-sex adj HDL Offsp wom ex1	http://www.ncbi.nlm.nih.gov/projects/gap/cgi-bin/analysis.cgi?id=pha000760
Metabolic	Lipidswomen	womenhdl2b	Age-sex adj HDL Offsp wom ex2	http://www.ncbi.nlm.nih.gov/projects/gap/cgi-bin/analysis.cgi?id=pha000762
Metabolic	Lipidswomen	womenhdl3b	Age-sex adj HDL Offsp wom ex3	http://www.ncbi.nlm.nih.gov/projects/gap/cgi-bin/analysis.cgi?id=pha000764
Metabolic	Lipidswomen	womenhdl4b	Age-sex adj HDL Offsp wom ex4	http://www.ncbi.nlm.nih.gov/projects/gap/cgi-bin/analysis.cgi?id=pha000766
Metabolic	Lipidswomen	womenhdl5b	Age-sex adj HDL Offsp wom ex5	http://www.ncbi.nlm.nih.gov/projects/gap/cgi-bin/analysis.cgi?id=pha000768
Metabolic	Lipidswomen	womenhdl6b	Age-sex adj HDL Offsp wom ex6	http://www.ncbi.nlm.nih.gov/projects/gap/cgi-bin/analysis.cgi?id=pha000770
Metabolic	Lipidswomen	womenhdl7b	Age-sex adj HDL Offsp wom ex7	http://www.ncbi.nlm.nih.gov/projects/gap/cgi-bin/analysis.cgi?id=pha000772
Metabolic	Lipidswomen	womenHDLNMRint4a	Multivariable adj NMR HDL Int Offsp wom ex4	http://www.ncbi.nlm.nih.gov/projects/gap/cgi-bin/analysis.cgi?id=pha000814
Metabolic	Lipidswomen	womenHDLNMRint4b	Age-sex adj NMR HDL Int Offsp wom ex4	http://www.ncbi.nlm.nih.gov/projects/gap/cgi-bin/analysis.cgi?id=pha000816
Metabolic	Lipidswomen	womenHDLNMRlg4a	Multivariable adj NMR HDL Lg Offsp wom ex4	http://www.ncbi.nlm.nih.gov/projects/gap/cgi-bin/analysis.cgi?id=pha000822
Metabolic	Lipidswomen	womenHDLNMRlg4b	Age-sex adj NMR HDL Lg Offsp wom ex4	http://www.ncbi.nlm.nih.gov/projects/gap/cgi-bin/analysis.cgi?id=pha000824
Metabolic	Lipidswomen	womenHDLNMRsm4a	Multivariable adj NMR HDL Sm Offsp wom ex4	http://www.ncbi.nlm.nih.gov/projects/gap/cgi-bin/analysis.cgi?id=pha000838
Metabolic	Lipidswomen	womenHDLNMRsm4b	Age-sex adj NMR HDL Sm Offsp wom ex4	http://www.ncbi.nlm.nih.gov/projects/gap/cgi-bin/analysis.cgi?id=pha000840
Metabolic	Lipidswomen	womenHDLNMRsz4a	Multivariable adj NMR HDL Size Offsp wom ex4	http://www.ncbi.nlm.nih.gov/projects/gap/cgi-bin/analysis.cgi?id=pha000830
Metabolic	Lipidswomen	womenHDLNMRsz4b	Age-sex adj NMR HDL Size Offsp wom ex4	http://www.ncbi.nlm.nih.gov/projects/gap/cgi-bin/analysis.cgi?id=pha000832
Metabolic	Lipidswomen	womenldl1a	Multivariable adj LDL Offsp wom ex1	http://www.ncbi.nlm.nih.gov/projects/gap/cgi-bin/analysis.cgi?id=pha000696
Metabolic	Lipidswomen	womenldl1b	Age-sex adj LDL Offsp wom ex1	http://www.ncbi.nlm.nih.gov/projects/gap/cgi-bin/analysis.cgi?id=pha000776
Metabolic	Lipidswomen	womenldl2b	Age-sex adj LDL Offsp wom ex2	http://www.ncbi.nlm.nih.gov/projects/gap/cgi-bin/analysis.cgi?id=pha000698
Metabolic	Lipidswomen	womenldl3b	Age-sex adj LDL Offsp wom ex3	http://www.ncbi.nlm.nih.gov/projects/gap/cgi-bin/analysis.cgi?id=pha000700
Metabolic	Lipidswomen	womenldl4b	Age-sex adj LDL Offsp wom ex4	http://www.ncbi.nlm.nih.gov/projects/gap/cgi-bin/analysis.cgi?id=pha000702
Metabolic	Lipidswomen	womenldl5b	Age-sex adj LDL Offsp wom ex5	http://www.ncbi.nlm.nih.gov/projects/gap/cgi-bin/analysis.cgi?id=pha000704

Group	Category	Trait Label	Name	GEE Link
Metabolic	Lipidswomen	womenldl6b	Age-sex adj LDL Offsp wom ex6	http://www.ncbi.nlm.nih.gov/projects/gap/cgi-bin/analysis.cgi?id=pha000706
Metabolic	Lipidswomen	womenldl7b	Age-sex adj LDL Offsp wom ex7	http://www.ncbi.nlm.nih.gov/projects/gap/cgi-bin/analysis.cgi?id=pha000708
Metabolic	Lipidswomen	womenLDLNMRlg4a	Multivariable adj NMR LDL Lg Offsp wom ex4	http://www.ncbi.nlm.nih.gov/projects/gap/cgi-bin/analysis.cgi?id=pha000846
Metabolic	Lipidswomen	womenLDLNMRlg4b	Age-sex adj NMR LDL Lg Offsp wom ex4	http://www.ncbi.nlm.nih.gov/projects/gap/cgi-bin/analysis.cgi?id=pha000848
Metabolic	Lipidswomen	womenLDLNMRsm4a	Multivariable adj NMR LDL Sm Offsp wom ex4	http://www.ncbi.nlm.nih.gov/projects/gap/cgi-bin/analysis.cgi?id=pha000862
Metabolic	Lipidswomen	womenLDLNMRsm4b	Age-sex adj NMR LDL Sm Offsp wom ex4	http://www.ncbi.nlm.nih.gov/projects/gap/cgi-bin/analysis.cgi?id=pha000864
Metabolic	Lipidswomen	womenLDLNMRsz4a	Multivariable adj NMR LDL Size Offsp wom ex4	http://www.ncbi.nlm.nih.gov/projects/gap/cgi-bin/analysis.cgi?id=pha000854
Metabolic	Lipidswomen	womenLDLNMRsz4b	Age-sex adj NMR LDL Size Offsp wom ex4	http://www.ncbi.nlm.nih.gov/projects/gap/cgi-bin/analysis.cgi?id=pha000856
Metabolic	Lipidswomen	womenmeanchola	Multivariable adj Mean Chol Offsp wom ex1-7	http://www.ncbi.nlm.nih.gov/projects/gap/cgi-bin/analysis.cgi?id=pha000798
Metabolic	Lipidswomen	womenmeancholb	Age-sex adj Mean Chol Offsp wom ex1-7	http://www.ncbi.nlm.nih.gov/projects/gap/cgi-bin/analysis.cgi?id=pha000800
Metabolic	Lipidswomen	womenmeanhdla	Multivariable adj Mean HDL Offsp wom ex1-7	http://www.ncbi.nlm.nih.gov/projects/gap/cgi-bin/analysis.cgi?id=pha000806
Metabolic	Lipidswomen	womenmeanhdlb	Age-sex adj Mean HDL Offsp wom ex1-7	http://www.ncbi.nlm.nih.gov/projects/gap/cgi-bin/analysis.cgi?id=pha000808
Metabolic	Lipidswomen	womenmeanldla	Multivariable adj Mean LDL Offsp wom ex1-7	http://www.ncbi.nlm.nih.gov/projects/gap/cgi-bin/analysis.cgi?id=pha000790
Metabolic	Lipidswomen	womenmeanldlb	Age-sex adj Mean LDL Offsp wom ex1-7	http://www.ncbi.nlm.nih.gov/projects/gap/cgi-bin/analysis.cgi?id=pha000792
Metabolic	Lipidswomen	womenmeantga	Multivariable adj Mean TG Offsp wom ex1-7	http://www.ncbi.nlm.nih.gov/projects/gap/cgi-bin/analysis.cgi?id=pha000782
Metabolic	Lipidswomen	womenmeantgb	Age-sex adj Mean TG Offsp wom ex1-7	http://www.ncbi.nlm.nih.gov/projects/gap/cgi-bin/analysis.cgi?id=pha000784
Metabolic	Lipidswomen	womentg1a	Multivariable adj TG Offsp wom ex1	http://www.ncbi.nlm.nih.gov/projects/gap/cgi-bin/analysis.cgi?id=pha000652
Metabolic	Lipidswomen	womentg1b	Age-sex adj TG Offsp wom ex1	http://www.ncbi.nlm.nih.gov/projects/gap/cgi-bin/analysis.cgi?id=pha000654
Metabolic	Lipidswomen	womentg2b	Age-sex adj TG Offsp wom ex2	http://www.ncbi.nlm.nih.gov/projects/gap/cgi-bin/analysis.cgi?id=pha000656
Metabolic	Lipidswomen	womentg3b	Age-sex adj TG Offsp wom ex3	http://www.ncbi.nlm.nih.gov/projects/gap/cgi-bin/analysis.cgi?id=pha000658
Metabolic	Lipidswomen	womentg4b	Age-sex adj TG Offsp wom ex4	http://www.ncbi.nlm.nih.gov/projects/gap/cgi-bin/analysis.cgi?id=pha000660
Metabolic	Lipidswomen	womentg5b	Age-sex adj TG Offsp wom ex5	http://www.ncbi.nlm.nih.gov/projects/gap/cgi-bin/analysis.cgi?id=pha000662
Metabolic	Lipidswomen	womentg6b	Age-sex adj TG Offsp wom ex6	http://www.ncbi.nlm.nih.gov/projects/gap/cgi-bin/analysis.cgi?id=pha000664
Metabolic	Lipidswomen	womentg7b	Age-sex adj TG Offsp wom ex7	http://www.ncbi.nlm.nih.gov/projects/gap/cgi-bin/analysis.cgi?id=pha000666
Metabolic	Lipidswomen	womenVLDLNMRint4a	Multivariable adj NMR VLDL Int Offsp wom ex4	http://www.ncbi.nlm.nih.gov/projects/gap/cgi-bin/analysis.cgi?id=pha000874
Metabolic	Lipidswomen	womenVLDLNMRint4b	Age-sex adj NMR VLDL Int Offsp wom ex4	http://www.ncbi.nlm.nih.gov/projects/gap/cgi-bin/analysis.cgi?id=pha000876
Metabolic	Lipidswomen	womenVLDLNMRlg4a	Multivariable adj NMR VLDL Lg Offsp wom ex4	http://www.ncbi.nlm.nih.gov/projects/gap/cgi-bin/analysis.cgi?id=pha000886
Metabolic	Lipidswomen	womenVLDLNMRlg4b	Age-sex adj NMR VLDL Lg Offsp wom ex4	http://www.ncbi.nlm.nih.gov/projects/gap/cgi-bin/analysis.cgi?id=pha000888
Metabolic	Lipidswomen	womenVLDLNMRsm4a	Multivariable adj NMR VLDL Sm Offsp wom ex4	http://www.ncbi.nlm.nih.gov/projects/gap/cgi-bin/analysis.cgi?id=pha000910
Metabolic	Lipidswomen	womenVLDLNMRsm4b	Age-sex NMR VLDL Sm Offsp wom ex4	http://www.ncbi.nlm.nih.gov/projects/gap/cgi-bin/analysis.cgi?id=pha000912
Metabolic	Lipidswomen	womenVLDLNMRsz4a	Multivariable adj NMR VLDL Size Offsp wom ex4	http://www.ncbi.nlm.nih.gov/projects/gap/cgi-bin/analysis.cgi?id=pha000898
Metabolic	Lipidswomen	womenVLDLNMRsz4b	Age-sex adj NMR VLDL Size Offsp wom ex4	http://www.ncbi.nlm.nih.gov/projects/gap/cgi-bin/analysis.cgi?id=pha000900
Pulmonary	Circadian	bedtime	usual weekday bedtime unadjusted	http://www.ncbi.nlm.nih.gov/projects/gap/cgi-bin/analysis.cgi?id=pha000006
Pulmonary	Circadian	bedtimeresid	usual weekday bedtime adjusted	http://www.ncbi.nlm.nih.gov/projects/gap/cgi-bin/analysis.cgi?id=pha000008
Pulmonary	Circadian	sleepdur	usual weekday sleep duration unadjusted	http://www.ncbi.nlm.nih.gov/projects/gap/cgi-bin/analysis.cgi?id=pha000010
Pulmonary	Circadian	sleepdurresid	usual weekday sleep duration adjusted	http://www.ncbi.nlm.nih.gov/projects/gap/cgi-bin/analysis.cgi?id=pha000012
Pulmonary	longitudinal	fef2575long	rate of decline of FEF(25-75)	http://www.ncbi.nlm.nih.gov/projects/gap/cgi-bin/analysis.cgi?id=pha000014
Pulmonary	longitudinal	fef2575longsmoke10	rate of decline of FEF(25-75) in 10+ packyear smokers	http://www.ncbi.nlm.nih.gov/projects/gap/cgi-bin/analysis.cgi?id=pha000016
Pulmonary	longitudinal	feffvclong	rate of decline of FEF(25-75)/FVC	http://www.ncbi.nlm.nih.gov/projects/gap/cgi-bin/analysis.cgi?id=pha000018
Pulmonary	longitudinal	feffvclongsmoke10	rate of decline of FEF(25-75)/FVC in 10+ packyear smokers	http://www.ncbi.nlm.nih.gov/projects/gap/cgi-bin/analysis.cgi?id=pha000020
Pulmonary	longitudinal	fev1fvclong	rate of decline of FEV1/FVC	http://www.ncbi.nlm.nih.gov/projects/gap/cgi-bin/analysis.cgi?id=pha000022
Pulmonary	longitudinal	fev1fvclongsmoke10	rate of decline of FEV1/FVC in 10+ packyear smokers	http://www.ncbi.nlm.nih.gov/projects/gap/cgi-bin/analysis.cgi?id=pha000024
Pulmonary	longitudinal	fev1long	rate of decline of FEV1	http://www.ncbi.nlm.nih.gov/projects/gap/cgi-bin/analysis.cgi?id=pha000026
Pulmonary	longitudinal	fev1longsmoke10	rate of decline of FEV1 in 10+ packyear smokers	http://www.ncbi.nlm.nih.gov/projects/gap/cgi-bin/analysis.cgi?id=pha000028
Pulmonary	longitudinal	fvclong	rate of decline of FVC	http://www.ncbi.nlm.nih.gov/projects/gap/cgi-bin/analysis.cgi?id=pha000030
Pulmonary	longitudinal	fvclongsmoke10	rate of decline of FVC in 10+ packyear smokers	http://www.ncbi.nlm.nih.gov/projects/gap/cgi-bin/analysis.cgi?id=pha000032
Pulmonary	mean	genomefev1	mean FEV1 from 2 examinations	http://www.ncbi.nlm.nih.gov/projects/gap/cgi-bin/analysis.cgi?id=pha000034
Pulmonary	mean	genomefvc	mean FVC from 2 examinations	http://www.ncbi.nlm.nih.gov/projects/gap/cgi-bin/analysis.cgi?id=pha000036
Pulmonary	mean	genomeratio	mean FEV1/FVC from 2 examinations	http://www.ncbi.nlm.nih.gov/projects/gap/cgi-bin/analysis.cgi?id=pha000038
Pulmonary	ppcrosssectional	ppfefadj	percent predicted FEF(25-75) at latest exam	http://www.ncbi.nlm.nih.gov/projects/gap/cgi-bin/analysis.cgi?id=pha000040
Pulmonary	ppcrosssectional	ppfefadjsm10	percent predicted FEF(25-75) at latest exam in 10+ packyear smokers	http://www.ncbi.nlm.nih.gov/projects/gap/cgi-bin/analysis.cgi?id=pha000042
Pulmonary	ppcrosssectional	ppfefratadj	percent predicted FEF(25-75)/FVC at latest exam	http://www.ncbi.nlm.nih.gov/projects/gap/cgi-bin/analysis.cgi?id=pha000044
Pulmonary	ppcrosssectional	ppfefratadjsm10	percent predicted FEF(25-75)/FVC latest exam, 10+ packyear smokers	http://www.ncbi.nlm.nih.gov/projects/gap/cgi-bin/analysis.cgi?id=pha000046
Pulmonary	ppcrosssectional	ppfev1adj	percent predicted FEV1 at latest exam	http://www.ncbi.nlm.nih.gov/projects/gap/cgi-bin/analysis.cgi?id=pha000048
Pulmonary	ppcrosssectional	ppfev1adjsm10	percent predicted FEV1 at latest exam in 10+ packyear smokers	http://www.ncbi.nlm.nih.gov/projects/gap/cgi-bin/analysis.cgi?id=pha000050

Group	Category	Trait Label	Name	GEE Link
Pulmonary	ppcrosssectional	ppfvcadj	percent predicted FVC at latest exam	http://www.ncbi.nlm.nih.gov/projects/gap/cgi-bin/analysis.cgi?id=pha000052
Pulmonary	ppcrosssectional	ppfvcadjsm10	percent predicted FVC at latest exam in 10+ packyear smokers	http://www.ncbi.nlm.nih.gov/projects/gap/cgi-bin/analysis.cgi?id=pha000054
Pulmonary	ppcrosssectional	ppratioadj	percent predicted FEV1/FVC at latest exam	http://www.ncbi.nlm.nih.gov/projects/gap/cgi-bin/analysis.cgi?id=pha000056
Pulmonary	ppcrosssectional	ppratioadjsm10	percent predicted FEV1/FVC at latest exam in 10+ packyear smokers	http://www.ncbi.nlm.nih.gov/projects/gap/cgi-bin/analysis.cgi?id=pha000058
Pulmonary	rcrosssectional	rfefadj	residual from predicted FEF(25-75) at latest exam	http://www.ncbi.nlm.nih.gov/projects/gap/cgi-bin/analysis.cgi?id=pha000060
Pulmonary	rcrosssectional	rfefadjsm10	residual from predicted FEF(25-75) at latest exam in 10+ packyear smokers	http://www.ncbi.nlm.nih.gov/projects/gap/cgi-bin/analysis.cgi?id=pha000062
Pulmonary	rcrosssectional	rfefratadj	residual from predicted FEF(25-75)/FVC at latest exam	http://www.ncbi.nlm.nih.gov/projects/gap/cgi-bin/analysis.cgi?id=pha000064
Pulmonary	rcrosssectional	rfefratadjsm10	residual from predicted FEF(25-75)/FVC latest exam in 10+ packyear smokers	http://www.ncbi.nlm.nih.gov/projects/gap/cgi-bin/analysis.cgi?id=pha000066
Pulmonary	rcrosssectional	rfev1adj	residual from predicted FEV1 at latest exam	http://www.ncbi.nlm.nih.gov/projects/gap/cgi-bin/analysis.cgi?id=pha000068
Pulmonary	rcrosssectional	rfev1adjsm10	residual from predicted FEV1 at latest exam in 10+ packyear smokers	http://www.ncbi.nlm.nih.gov/projects/gap/cgi-bin/analysis.cgi?id=pha000070
Pulmonary	rcrosssectional	rfvcadj	residual from predicted FVC at latest exam	http://www.ncbi.nlm.nih.gov/projects/gap/cgi-bin/analysis.cgi?id=pha000072
Pulmonary	rcrosssectional	rfvcadjsm10	residual from predicted FVC at latest exam in 10+ packyear smokers	http://www.ncbi.nlm.nih.gov/projects/gap/cgi-bin/analysis.cgi?id=pha000074
Pulmonary	rcrosssectional	rratioadj	residual from predicted FEV1/FVC at latest exam	http://www.ncbi.nlm.nih.gov/projects/gap/cgi-bin/analysis.cgi?id=pha000076
Pulmonary	rcrosssectional	rratioadjsm10	residual from predicted FEV1/FVC at latest exam in 10+ packyear smokers	http://www.ncbi.nlm.nih.gov/projects/gap/cgi-bin/analysis.cgi?id=pha000078
Pulmonary	SDB	rdiresid	apnea-hypopnea index with 4% desaturation adjusted	http://www.ncbi.nlm.nih.gov/projects/gap/cgi-bin/analysis.cgi?id=pha000080
Pulmonary	singleexam	ppfev1single	percent predicted FEV1, exam: offspring 6, cohort 17	http://www.ncbi.nlm.nih.gov/projects/gap/cgi-bin/analysis.cgi?id=pha000082
Pulmonary	singleexam	ppfvcsingle	percent predicted FVC, exam: offspring 6, cohort 17	http://www.ncbi.nlm.nih.gov/projects/gap/cgi-bin/analysis.cgi?id=pha000084
Pulmonary	singleexam	ppratiosingle	percent predicted FEV1/FVC, exam: offspring 6, cohort 17	http://www.ncbi.nlm.nih.gov/projects/gap/cgi-bin/analysis.cgi?id=pha000086
Pulmonary	singleexam	rfev1single	residual from predicted FEV1, exam: offspring 6, cohort 17	http://www.ncbi.nlm.nih.gov/projects/gap/cgi-bin/analysis.cgi?id=pha000088
Pulmonary	singleexam	rfvcsingle	residual from predicted FVC, exam: offspring 6, cohort 17	http://www.ncbi.nlm.nih.gov/projects/gap/cgi-bin/analysis.cgi?id=pha000090
Pulmonary	singleexam	rratiosingle	residual from predicted FEV1/FVC, exam: offspring 6, cohort 17	http://www.ncbi.nlm.nih.gov/projects/gap/cgi-bin/analysis.cgi?id=pha000092
Pulmonary	Sleepiness	ess	Epworth Sleepiness Scale, unadjusted	http://www.ncbi.nlm.nih.gov/projects/gap/cgi-bin/analysis.cgi?id=pha000094
Pulmonary	Sleepiness	essresid1	Epworth Sleepiness Scale, adjusted A	http://www.ncbi.nlm.nih.gov/projects/gap/cgi-bin/analysis.cgi?id=pha000096
Pulmonary	Sleepiness	essresid2	Epworth Sleepiness Scale, adjusted B	http://www.ncbi.nlm.nih.gov/projects/gap/cgi-bin/analysis.cgi?id=pha000098
Pulmonary	Sleepiness	essresid3	Epworth Sleepiness Scale, adjusted C	http://www.ncbi.nlm.nih.gov/projects/gap/cgi-bin/analysis.cgi?id=pha000100
RENALENDO	ENDO	DHEASAS3	DHEAS adjusted for age and sex	http://www.ncbi.nlm.nih.gov/projects/gap/cgi-bin/analysis.cgi?id=pha001220
RENALENDO	ENDO	DHEASMV3	DHEAS MV adjusted	http://www.ncbi.nlm.nih.gov/projects/gap/cgi-bin/analysis.cgi?id=pha001222
RENALENDO	ENDO	FSHAS3	FSH in men or post meno women no hormones, age-sex adjusted	http://www.ncbi.nlm.nih.gov/projects/gap/cgi-bin/analysis.cgi?id=pha001224
RENALENDO	ENDO	FSHMV3	FSH in men or post meno women no hormones, MV adjusted	http://www.ncbi.nlm.nih.gov/projects/gap/cgi-bin/analysis.cgi?id=pha001226
RENALENDO	ENDO	LHAS3	LH in men or post-meno women no hormones, age-sex adjusted	http://www.ncbi.nlm.nih.gov/projects/gap/cgi-bin/analysis.cgi?id=pha001228
RENALENDO	ENDO	LHMV3	LH in men or post-meno women no hormones, MV adjusted	http://www.ncbi.nlm.nih.gov/projects/gap/cgi-bin/analysis.cgi?id=pha001230
RENALENDO	ENDO	TSHLNAS3	TSH from ex3 log transformed, age-sex adjusted	http://www.ncbi.nlm.nih.gov/projects/gap/cgi-bin/analysis.cgi?id=pha001232
RENALENDO	ENDO	TSHLNAS4	TSH from ex4 log transformed, age-sex adjusted	http://www.ncbi.nlm.nih.gov/projects/gap/cgi-bin/analysis.cgi?id=pha001234
RENALENDO	ENDO	TSHLNMV3	TSH ex3 log transformed, MV adjusted	http://www.ncbi.nlm.nih.gov/projects/gap/cgi-bin/analysis.cgi?id=pha001236
RENALENDO	ENDO	TSHLNMV4	TSH ex4 log transformed, MV adjusted	http://www.ncbi.nlm.nih.gov/projects/gap/cgi-bin/analysis.cgi?id=pha001238
RENALENDO	ENDO	TSHMEAN34LNAS	Mean TSH log transformed, mean of ex3 and ex4, age sex adjusted	http://www.ncbi.nlm.nih.gov/projects/gap/cgi-bin/analysis.cgi?id=pha001240
RENALENDO	ENDO	TSHMEAN34LNMV	Mean TSH ex3 and 4, MV adjusted	http://www.ncbi.nlm.nih.gov/projects/gap/cgi-bin/analysis.cgi?id=pha001242
RENALENDO	RENAL	CALCIUMAS2	Calcium, ex2, age-sex adjusted	http://www.ncbi.nlm.nih.gov/projects/gap/cgi-bin/analysis.cgi?id=pha001244
RENALENDO	RENAL	CALCIUMMV2	Calcium, ex2, age-sex-creatinine adjusted	http://www.ncbi.nlm.nih.gov/projects/gap/cgi-bin/analysis.cgi?id=pha001246
RENALENDO	RENAL	CHNGSCR27ASWIN	Change in creatinine ex2 to 7, age-sex adjusted	http://www.ncbi.nlm.nih.gov/projects/gap/cgi-bin/analysis.cgi?id=pha001248
RENALENDO	RENAL	CHNGSCR27MVWIN	Change in creatinine ex2 to 7, MV adjusted	http://www.ncbi.nlm.nih.gov/projects/gap/cgi-bin/analysis.cgi?id=pha001250
RENALENDO	RENAL	CKDAS7	CKD ex 7, age-sex adjusted	http://www.ncbi.nlm.nih.gov/projects/gap/cgi-bin/analysis.cgi?id=pha001252
RENALENDO	RENAL	CKDMV7	CKD ex 7, MV adjusted	http://www.ncbi.nlm.nih.gov/projects/gap/cgi-bin/analysis.cgi?id=pha001254
RENALENDO	RENAL	CYSCAS7	Cystatin C, ex 7, age-sex adjusted	http://www.ncbi.nlm.nih.gov/projects/gap/cgi-bin/analysis.cgi?id=pha001328
RENALENDO	RENAL	CYSCMV7	Cystatin C, ex 7, MV adjusted	http://www.ncbi.nlm.nih.gov/projects/gap/cgi-bin/analysis.cgi?id=pha001330
RENALENDO	RENAL	GFRAS5	GFR ex 5, age-sex adjusted	http://www.ncbi.nlm.nih.gov/projects/gap/cgi-bin/analysis.cgi?id=pha001256
RENALENDO	RENAL	GFRAS6	GFR ex 6, age-sex adjusted	http://www.ncbi.nlm.nih.gov/projects/gap/cgi-bin/analysis.cgi?id=pha001258
RENALENDO	RENAL	GFRAS7	GFR ex 7, age-sex adjusted	http://www.ncbi.nlm.nih.gov/projects/gap/cgi-bin/analysis.cgi?id=pha001260
RENALENDO	RENAL	GFRASWIN2	GFR ex2, winsorized, age-sex adjusted	http://www.ncbi.nlm.nih.gov/projects/gap/cgi-bin/analysis.cgi?id=pha001262
RENALENDO	RENAL	GFRMV5	GFR ex5, MV adjusted	http://www.ncbi.nlm.nih.gov/projects/gap/cgi-bin/analysis.cgi?id=pha001264
RENALENDO	RENAL	GFRMV6	GFR ex6, MV adjusted	http://www.ncbi.nlm.nih.gov/projects/gap/cgi-bin/analysis.cgi?id=pha001266
RENALENDO	RENAL	GFRMV7	GFR ex7, MV adjusted	http://www.ncbi.nlm.nih.gov/projects/gap/cgi-bin/analysis.cgi?id=pha001268
RENALENDO	RENAL	GFRMVWIN2	GFR ex2, winsorized, MV adjusted	http://www.ncbi.nlm.nih.gov/projects/gap/cgi-bin/analysis.cgi?id=pha001270
RENALENDO	RENAL	MeanGFRAS2567WIN	Mean GFR ex 2,5,6,7 age-sex adjusted	http://www.ncbi.nlm.nih.gov/projects/gap/cgi-bin/analysis.cgi?id=pha001272
RENALENDO	RENAL	MeanGFRMV2567WIN	Mean GFR ex 2,5,6,7,MV adjusted	http://www.ncbi.nlm.nih.gov/projects/gap/cgi-bin/analysis.cgi?id=pha001274

Group	Category	Trait Label	Name	GEE Link
RENALENDO	RENAL	MeanSCR2567LNASWIN	Mean creatinine ex 2,5,6,7 age-sex adjusted	http://www.ncbi.nlm.nih.gov/projects/gap/cgi-bin/analysis.cgi?id=pha001276
RENALENDO	RENAL	MeanSCR2567LNMVWIN	Mean creatinine ex 2,5,6,7, MV adjusted	http://www.ncbi.nlm.nih.gov/projects/gap/cgi-bin/analysis.cgi?id=pha001278
RENALENDO	RENAL	MeanURICACID12AS	Mean uric acid, ex 1 and 2, age-sex adjusted	http://www.ncbi.nlm.nih.gov/projects/gap/cgi-bin/analysis.cgi?id=pha001280
RENALENDO	RENAL	MeanURICACID12MV	Mean uric acid, ex 1 and 2, MV adjusted	http://www.ncbi.nlm.nih.gov/projects/gap/cgi-bin/analysis.cgi?id=pha001282
RENALENDO	RENAL	PHOSAS2	Phos, ex 2, age-sex adjusted	http://www.ncbi.nlm.nih.gov/projects/gap/cgi-bin/analysis.cgi?id=pha001284
RENALENDO	RENAL	PHOSMV2	Phos, ex 2, MV adjusted	http://www.ncbi.nlm.nih.gov/projects/gap/cgi-bin/analysis.cgi?id=pha001286
RENALENDO	RENAL	SCRLNAS5	Creatinine, ex5, age-sex adjusted	http://www.ncbi.nlm.nih.gov/projects/gap/cgi-bin/analysis.cgi?id=pha001288
RENALENDO	RENAL	SCRLNAS6	Creatinine, ex6, age-sex adjusted	http://www.ncbi.nlm.nih.gov/projects/gap/cgi-bin/analysis.cgi?id=pha001290
RENALENDO	RENAL	SCRLNASWIN2	Creatinine ex2, log transformed and winsorized, age-sex adjusted	http://www.ncbi.nlm.nih.gov/projects/gap/cgi-bin/analysis.cgi?id=pha001292
RENALENDO	RENAL	SCRLNASWIN7	Creatinine ex7, log transformed and winsorized, age-sex adjusted	http://www.ncbi.nlm.nih.gov/projects/gap/cgi-bin/analysis.cgi?id=pha001294
RENALENDO	RENAL	SCRLNMV5	GFR ex 5 uncalibrated creatinine, MV adjusted	http://www.ncbi.nlm.nih.gov/projects/gap/cgi-bin/analysis.cgi?id=pha001296
RENALENDO	RENAL	SCRLNMV6	GFR ex 6 uncalibrated creatinine, MV adjusted	http://www.ncbi.nlm.nih.gov/projects/gap/cgi-bin/analysis.cgi?id=pha001298
RENALENDO	RENAL	SCRLNMVWIN2	Creatinine ex 2 log transformed winsorized MV adjusted	http://www.ncbi.nlm.nih.gov/projects/gap/cgi-bin/analysis.cgi?id=pha001300
RENALENDO	RENAL	SCRLNMVWIN7	Creatinine ex 7 uncalibrated MV adjusted	http://www.ncbi.nlm.nih.gov/projects/gap/cgi-bin/analysis.cgi?id=pha001302
RENALENDO	RENAL	UAEGE30HTNAS6	UAE of at least 30 in enriched hypertensive sample, age-sex adjusted	http://www.ncbi.nlm.nih.gov/projects/gap/cgi-bin/analysis.cgi?id=pha001304
RENALENDO	RENAL	UAEGE30HTNMV6	UAE of at least 30 in enriched hypertensive sample, age-sex adjusted	http://www.ncbi.nlm.nih.gov/projects/gap/cgi-bin/analysis.cgi?id=pha001306
RENALENDO	RENAL	UAELNAS6	Log-transformed UAE, age-sex adjusted	http://www.ncbi.nlm.nih.gov/projects/gap/cgi-bin/analysis.cgi?id=pha001308
RENALENDO	RENAL	UAELNHTNAS6	Log-transformed UAE in HTN enriched sample, age-sex adjusted	http://www.ncbi.nlm.nih.gov/projects/gap/cgi-bin/analysis.cgi?id=pha001310
RENALENDO	RENAL	UAELNHTNMV6	Log-transformed UAE in HTN enriched sample, MV adjusted	http://www.ncbi.nlm.nih.gov/projects/gap/cgi-bin/analysis.cgi?id=pha001312
RENALENDO	RENAL	UAELNMV6	Log-transformed UAE, MV adjusted	http://www.ncbi.nlm.nih.gov/projects/gap/cgi-bin/analysis.cgi?id=pha001314
RENALENDO	RENAL	UNALNASWIN6	Urinary sodium, age-sex adjusted	http://www.ncbi.nlm.nih.gov/projects/gap/cgi-bin/analysis.cgi?id=pha001316
RENALENDO	RENAL	UNALNMVWIN6	Urinary sodium, MV adjusted	http://www.ncbi.nlm.nih.gov/projects/gap/cgi-bin/analysis.cgi?id=pha001318
RENALENDO	RENAL	URICACIDAS1	Uric acid, ex 1, age-sex adjusted	http://www.ncbi.nlm.nih.gov/projects/gap/cgi-bin/analysis.cgi?id=pha001320
RENALENDO	RENAL	URICACIDAS2	Uric acid, ex 2, age-sex adjusted	http://www.ncbi.nlm.nih.gov/projects/gap/cgi-bin/analysis.cgi?id=pha001322
RENALENDO	RENAL	URICACIDMV1	Uric acid, ex1, MV adjusted	http://www.ncbi.nlm.nih.gov/projects/gap/cgi-bin/analysis.cgi?id=pha001324
RENALENDO	RENAL	URICACIDMV2	Uric acid, ex2, MV adjusted	http://www.ncbi.nlm.nih.gov/projects/gap/cgi-bin/analysis.cgi?id=pha001326
SubclinCVD	Ankle-brachialindex	RANKLEBI6	Ankle-brachial index, cycle 6, age and sex-djusted	http://www.ncbi.nlm.nih.gov/projects/gap/cgi-bin/analysis.cgi?id=pha000928
SubclinCVD	Ankle-brachialindex	RANKLEBI6MV	Ankle-brachial index, cycle 6, multivariable-adjusted	http://www.ncbi.nlm.nih.gov/projects/gap/cgi-bin/analysis.cgi?id=pha000930
SubclinCVD	Ankle-brachialindex	RANKLEBI7	Ankle-brachial index, cycle 7, age- and sex-adjusted	http://www.ncbi.nlm.nih.gov/projects/gap/cgi-bin/analysis.cgi?id=pha000932
SubclinCVD	Ankle-brachialindex	RANKLEBI7MV	Ankle-brachial index, cycle 7, multivariable-adjusted	http://www.ncbi.nlm.nih.gov/projects/gap/cgi-bin/analysis.cgi?id=pha000934
SubclinCVD	BrainMRI	BMRILWMHVC	Log white matter hyperintensity to intracranial volume ratio, age and sex	http://www.ncbi.nlm.nih.gov/projects/gap/cgi-bin/analysis.cgi?id=pha000936
SubclinCVD	BrainMRI	BMRILWMHVMV	Log white matter hyperintensity to intracranial volume ratio, multivariable	http://www.ncbi.nlm.nih.gov/projects/gap/cgi-bin/analysis.cgi?id=pha000938
SubclinCVD	BrainMRI	BMRIZLWMHVC	Z-score log white matter hyperintensity volume ratio, sex adjusted	http://www.ncbi.nlm.nih.gov/projects/gap/cgi-bin/analysis.cgi?id=pha000940
SubclinCVD	BrainMRI	BMRIZLWMHVMV	Z-score log white matter hyperintensity volume ratio, multivariable	http://www.ncbi.nlm.nih.gov/projects/gap/cgi-bin/analysis.cgi?id=pha000942
SubclinCVD	Carotid	RNKCAROTBULBAS6	Carotid bulb IMT mean max, cycle 6, log transformed age and sex-adjusted	http://www.ncbi.nlm.nih.gov/projects/gap/cgi-bin/analysis.cgi?id=pha000944
SubclinCVD	Carotid	RNKCAROTBULBMV6	Carotid bulb IMT mean max, cycle 6, log transformed multivariable-adjusted	http://www.ncbi.nlm.nih.gov/projects/gap/cgi-bin/analysis.cgi?id=pha000946
SubclinCVD	Carotid	RNKCAROTCCAMAXAS6	Common carotid IMT mean max, cycle 6, log transformed age and sex-adjusted	http://www.ncbi.nlm.nih.gov/projects/gap/cgi-bin/analysis.cgi?id=pha000948
SubclinCVD	Carotid	RNKCAROTCCAMAXMV6	Common carotid IMT mean max, cycle 6, log transformed multivariable-adjusted	http://www.ncbi.nlm.nih.gov/projects/gap/cgi-bin/analysis.cgi?id=pha000950
SubclinCVD	Carotid	RNKCAROTCCAMEANAS6	Common carotid IMT mean mean, cycle 6, log transformed age and sex-adjusted	http://www.ncbi.nlm.nih.gov/projects/gap/cgi-bin/analysis.cgi?id=pha000952
SubclinCVD	Carotid	RNKCAROTCCAMEANMV6	Common carotid IMT mean mean, cycle 6, log transformed multivariable-adjusted	http://www.ncbi.nlm.nih.gov/projects/gap/cgi-bin/analysis.cgi?id=pha000954
SubclinCVD	Carotid	RNKCAROTICAMAXAS6	Internal carotid IMT mean max, cycle 6, log transformed age and sex-adjusted	http://www.ncbi.nlm.nih.gov/projects/gap/cgi-bin/analysis.cgi?id=pha000956
SubclinCVD	Carotid	RNKCAROTICAMAXMV6	Internal carotid IMT mean max, cycle 6, log transformed multivariable-adjusted	http://www.ncbi.nlm.nih.gov/projects/gap/cgi-bin/analysis.cgi?id=pha000958
SubclinCVD	Carotid	RNKCAROTICAMEANAS6	Internal carotid IMT mean mean, cycle 6, log transformed age and sex-adjusted	http://www.ncbi.nlm.nih.gov/projects/gap/cgi-bin/analysis.cgi?id=pha000960
SubclinCVD	Carotid	RNKCAROTICAMEANMV6	Internal carotid IMT mean mean, cycle 6, log transformed multivariable-adjusted	http://www.ncbi.nlm.nih.gov/projects/gap/cgi-bin/analysis.cgi?id=pha000962
SubclinCVD	Carotid	RNKCAROTSTENAS6	Maximum Carotid Stenosis, cycle 6, age and sex-adjusted	http://www.ncbi.nlm.nih.gov/projects/gap/cgi-bin/analysis.cgi?id=pha000964
SubclinCVD	Carotid	RNKCAROTSTENMV6	Maximum Carotid Stenosis, cycle 6 multivariable-adjusted	http://www.ncbi.nlm.nih.gov/projects/gap/cgi-bin/analysis.cgi?id=pha000966
SubclinCVD	CT	RESMDCTAACAS7	Mean Agatston AAC score, MDCT ~cycle 7, log transform, age and sex-adjusted	http://www.ncbi.nlm.nih.gov/projects/gap/cgi-bin/analysis.cgi?id=pha000968
SubclinCVD	CT	RESMDCTAACMV7	Mean Agatston AAC score, MDCT ~cycle 7, log transform multivariable-adjusted	http://www.ncbi.nlm.nih.gov/projects/gap/cgi-bin/analysis.cgi?id=pha000970
SubclinCVD	CT	RESMDCTCACAS7	Mean Agatston CAC score, MDCT ~cycle 7, log transform age and sex-adjusted	http://www.ncbi.nlm.nih.gov/projects/gap/cgi-bin/analysis.cgi?id=pha000972
SubclinCVD	CT	RESMDCTCACMAXAS7	Max Agatston CAC score, MDCT ~cycle 7, log transform age and sex-adjusted	http://www.ncbi.nlm.nih.gov/projects/gap/cgi-bin/analysis.cgi?id=pha000974
SubclinCVD	CT	RESMDCTCACMAXMV7	Max Agatston CAC score, MDCT ~cycle 7, log transform multivariable-adjusted	http://www.ncbi.nlm.nih.gov/projects/gap/cgi-bin/analysis.cgi?id=pha000976
SubclinCVD	CT	RESMDCTCACMV7	Mean Agatston CAC score, MDCT ~cycle 7, log transform multivariable-adjusted	http://www.ncbi.nlm.nih.gov/projects/gap/cgi-bin/analysis.cgi?id=pha000978
