# Supplementary material for: The Framingham Heart Study 100K SNP genome-wide association study resource: overview of 17 phenotype working group reports
Source: BMC Med Genet. 2007 Sep 19;8(Suppl 1):S1. doi: 10.1186/1471-2350-8-S1-S1 (PMC1995613; doi:10.1186/1471-2350-8-S1-S1)
Supplement: Additional file 2 — Phenotypes for family-based FBAT analyses. [file 1471-2350-8-S1-S1-S2.pdf]

Online Table 2: Phenotypes Evaluated for Association using Family-based Analysis (FBAT)

Group	Category	Trait Label	Name	FBAT Link
Aging	BoneAging	BUA	Bone Ultrasound attenuation measured by QUS, multivariable adjusted	http://www.ncbi.nlm.nih.gov/projects/gap/cgi-bin/analysis.cgi?id=pha001753
Aging	BoneAging	deltaOSSR	Bone age by osseographic scoring system, multivariable adjusted	http://www.ncbi.nlm.nih.gov/projects/gap/cgi-bin/analysis.cgi?id=pha001787
Aging	BoneAging	deltaOSSrf	Bone age by osseographic scoring system (Women), multivariable adjusted	http://www.ncbi.nlm.nih.gov/projects/gap/cgi-bin/analysis.cgi?id=pha001789
Aging	BoneAging	deltaOSSrm	Bone age by osseographic scoring system (Men, multivariable adjusted)	http://www.ncbi.nlm.nih.gov/projects/gap/cgi-bin/analysis.cgi?id=pha001791
Aging	BoneAging	FNBMD	Femoral Neck BMD measured by DXA, multivariable adjusted	http://www.ncbi.nlm.nih.gov/projects/gap/cgi-bin/analysis.cgi?id=pha001755
Aging	BoneAging	FNBMDf	Femoral Neck BMD measured by DXA (Women), multivariable adjusted	http://www.ncbi.nlm.nih.gov/projects/gap/cgi-bin/analysis.cgi?id=pha001793
Aging	BoneAging	FNBMDm	Femoral Neck BMD measured by DXA (Men), multivariable adjusted	http://www.ncbi.nlm.nih.gov/projects/gap/cgi-bin/analysis.cgi?id=pha001795
Aging	BoneAging	ITAvgBR1rf	Hip geometry (IT BucklingRatio, Women), multivariable adjusted	http://www.ncbi.nlm.nih.gov/projects/gap/cgi-bin/analysis.cgi?id=pha001797
Aging	BoneAging	ITAvgBR1rm	Hip geometry (IT BucklingRatio, Men), multivariable adjusted	http://www.ncbi.nlm.nih.gov/projects/gap/cgi-bin/analysis.cgi?id=pha001799
Aging	BoneAging	ITAvgBRrf	Hip geometry (IT BucklingRatio, Women), age- adjusted	http://www.ncbi.nlm.nih.gov/projects/gap/cgi-bin/analysis.cgi?id=pha001801
Aging	BoneAging	ITAvgBRrm	Hip geometry (IT BucklingRatio, Men), age- adjusted	http://www.ncbi.nlm.nih.gov/projects/gap/cgi-bin/analysis.cgi?id=pha001803
Aging	BoneAging	LSBMD	Lumbar Spine BMD measured by DXA, multivariable adjusted	http://www.ncbi.nlm.nih.gov/projects/gap/cgi-bin/analysis.cgi?id=pha001757
Aging	BoneAging	LSBMDf	Spine Neck BMD measured by DXA (Women), multivariable adjusted	http://www.ncbi.nlm.nih.gov/projects/gap/cgi-bin/analysis.cgi?id=pha001817
Aging	BoneAging	LSBMDm	Spine Neck BMD measured by DXA (Men), multivariable adjusted	http://www.ncbi.nlm.nih.gov/projects/gap/cgi-bin/analysis.cgi?id=pha001819
Aging	BoneAging	NeckCSMI	Hip geometry by DXA (Neck Moment of Inertia), age- and sex-adjusted	http://www.ncbi.nlm.nih.gov/projects/gap/cgi-bin/analysis.cgi?id=pha001759
Aging	BoneAging	NeckCSMI1	Hip geometry by DXA (Neck Moment of Inertia), multivariable adjusted	http://www.ncbi.nlm.nih.gov/projects/gap/cgi-bin/analysis.cgi?id=pha001761
Aging	BoneAging	NeckLeng	Hip geometry by DXA (Neck Length), age- and sex-adjusted	http://www.ncbi.nlm.nih.gov/projects/gap/cgi-bin/analysis.cgi?id=pha001763
Aging	BoneAging	NeckLeng1	Hip geometry by DXA (Neck Length), multivariable adjusted	http://www.ncbi.nlm.nih.gov/projects/gap/cgi-bin/analysis.cgi?id=pha001765
Aging	BoneAging	NeckLengf	Hip geometry (Neck Length, Women), multivariable adjusted	http://www.ncbi.nlm.nih.gov/projects/gap/cgi-bin/analysis.cgi?id=pha001821
Aging	BoneAging	NeckLengm	Hip geometry (Neck Length, Men), multivariable adjusted	http://www.ncbi.nlm.nih.gov/projects/gap/cgi-bin/analysis.cgi?id=pha001823
Aging	BoneAging	NeckW	Hip geometry by DXA (Neck Width), age- and sex-adjusted	http://www.ncbi.nlm.nih.gov/projects/gap/cgi-bin/analysis.cgi?id=pha001767
Aging	BoneAging	NeckW1	Hip geometry by DXA (Neck Width), multivariable adjusted	http://www.ncbi.nlm.nih.gov/projects/gap/cgi-bin/analysis.cgi?id=pha001769
Aging	BoneAging	NeckW1rf	Hip geometry (Neck Width, Women), multivariable adjusted	http://www.ncbi.nlm.nih.gov/projects/gap/cgi-bin/analysis.cgi?id=pha001825
Aging	BoneAging	NeckW1rm	Hip geometry (Neck Width, Men), multivariable adjusted	http://www.ncbi.nlm.nih.gov/projects/gap/cgi-bin/analysis.cgi?id=pha001827
Aging	BoneAging	NeckWrf	Hip geometry (Neck Width, Women), age-adjusted	http://www.ncbi.nlm.nih.gov/projects/gap/cgi-bin/analysis.cgi?id=pha001829
Aging	BoneAging	NeckWrm	Hip geometry (Neck Width, Men), age-adjusted	http://www.ncbi.nlm.nih.gov/projects/gap/cgi-bin/analysis.cgi?id=pha001831
Aging	BoneAging	NeckZ	Hip geometry by DXA (Neck Section Modulus), age- and sex-adjusted	http://www.ncbi.nlm.nih.gov/projects/gap/cgi-bin/analysis.cgi?id=pha001771
Aging	BoneAging	NeckZ1	Hip geometry by DXA (Neck Section Modulus), multivariable adjusted	http://www.ncbi.nlm.nih.gov/projects/gap/cgi-bin/analysis.cgi?id=pha001773
Aging	BoneAging	NeckZ1rf	Hip geometry (Neck Section Modulus, Women), multivariable adjusted	http://www.ncbi.nlm.nih.gov/projects/gap/cgi-bin/analysis.cgi?id=pha001833
Aging	BoneAging	NeckZ1rm	Hip geometry (Neck Section Modulus, Men), multivariable adjusted	http://www.ncbi.nlm.nih.gov/projects/gap/cgi-bin/analysis.cgi?id=pha001835
Aging	BoneAging	NeckZrf	Hip geometry (Neck Section Modulus, Women), age-adjusted	http://www.ncbi.nlm.nih.gov/projects/gap/cgi-bin/analysis.cgi?id=pha001837
Aging	BoneAging	NeckZrm	Hip geometry (Neck Section Modulus, Men), age-adjusted	http://www.ncbi.nlm.nih.gov/projects/gap/cgi-bin/analysis.cgi?id=pha001839
Aging	BoneAging	NNAvgBR1rf	Hip geometry (NN BucklingRatio, Women), multivariable adjusted	http://www.ncbi.nlm.nih.gov/projects/gap/cgi-bin/analysis.cgi?id=pha001841
Aging	BoneAging	NNAvgBR1rm	Hip geometry (NN BucklingRatio, Men), multivariable adjusted	http://www.ncbi.nlm.nih.gov/projects/gap/cgi-bin/analysis.cgi?id=pha001843
Aging	BoneAging	NNAvgBRrf	Hip geometry (NN BucklingRatio, Women), age-adjusted	http://www.ncbi.nlm.nih.gov/projects/gap/cgi-bin/analysis.cgi?id=pha001845
Aging	BoneAging	NNAvgBRrm	Hip geometry (NN BucklingRatio, Men), age-adjusted	http://www.ncbi.nlm.nih.gov/projects/gap/cgi-bin/analysis.cgi?id=pha001847
Aging	BoneAging	NNCSMI1rf	Hip geometry (Neck CSMI, Women), multivariable adjusted	http://www.ncbi.nlm.nih.gov/projects/gap/cgi-bin/analysis.cgi?id=pha001849
Aging	BoneAging	NNCSMI1rm	Hip geometry (Neck CSMI, Men), multivariable adjusted	http://www.ncbi.nlm.nih.gov/projects/gap/cgi-bin/analysis.cgi?id=pha001851
Aging	BoneAging	NNCSMIrf	Hip geometry (Neck CSMI, Women), age-adjusted	http://www.ncbi.nlm.nih.gov/projects/gap/cgi-bin/analysis.cgi?id=pha001853
Aging	BoneAging	NNCSMIrm	Hip geometry (Neck CSMI, Men), age-adjusted	http://www.ncbi.nlm.nih.gov/projects/gap/cgi-bin/analysis.cgi?id=pha001855
Aging	BoneAging	NSA	Hip geometry by DXA (Neck-Shaft Angle), age- and sex-adjusted	http://www.ncbi.nlm.nih.gov/projects/gap/cgi-bin/analysis.cgi?id=pha001775
Aging	BoneAging	NSA1	Hip geometry by DXA (Neck-Shaft Angle), multivariable adjusted	http://www.ncbi.nlm.nih.gov/projects/gap/cgi-bin/analysis.cgi?id=pha001777
Aging	BoneAging	NSAf	Hip geometry (Neck-Shaft Angle, Women), multivariable adjusted	http://www.ncbi.nlm.nih.gov/projects/gap/cgi-bin/analysis.cgi?id=pha001857
Aging	BoneAging	NSAm	Hip geometry (Neck-Shaft Angle, Men), multivariable adjusted	http://www.ncbi.nlm.nih.gov/projects/gap/cgi-bin/analysis.cgi?id=pha001859
Aging	BoneAging	ShaftAvgBRr1f	Hip geometry (Shaft BucklingRatio, Women), multivariable adjusted	http://www.ncbi.nlm.nih.gov/projects/gap/cgi-bin/analysis.cgi?id=pha001861
Aging	BoneAging	ShaftAvgBRr1m	Hip geometry (Shaft BucklingRatio, Men), multivariable adjusted	http://www.ncbi.nlm.nih.gov/projects/gap/cgi-bin/analysis.cgi?id=pha001863
Aging	BoneAging	ShaftAvgBRrf	Hip geometry (Shaft BucklingRatio, Women), age-adjusted	http://www.ncbi.nlm.nih.gov/projects/gap/cgi-bin/analysis.cgi?id=pha001865
Aging	BoneAging	ShaftAvgBRrm	Hip geometry (Shaft BucklingRatio, Men), age-adjusted	http://www.ncbi.nlm.nih.gov/projects/gap/cgi-bin/analysis.cgi?id=pha001867
Aging	BoneAging	ShaftCSMI1R	Hip geometry (Shaft CSMI), multivariable adjusted	http://www.ncbi.nlm.nih.gov/projects/gap/cgi-bin/analysis.cgi?id=pha001869
Aging	BoneAging	ShaftCSMIrf	Hip geometry (Shaft CSMI, Women), multivariable adjusted	http://www.ncbi.nlm.nih.gov/projects/gap/cgi-bin/analysis.cgi?id=pha001871
Aging	BoneAging	ShaftCSMIrm	Hip geometry (Shaft CSMI, Men), multivariable adjusted	http://www.ncbi.nlm.nih.gov/projects/gap/cgi-bin/analysis.cgi?id=pha001873
Aging	BoneAging	ShaftW	Hip geometry by DXA (Shaft Width), age- and sex-adjusted	http://www.ncbi.nlm.nih.gov/projects/gap/cgi-bin/analysis.cgi?id=pha001779
Aging	BoneAging	ShaftW1	Hip geometry by DXA (Shaft Width), multivariable adjusted	http://www.ncbi.nlm.nih.gov/projects/gap/cgi-bin/analysis.cgi?id=pha001781
Aging	BoneAging	ShaftW1f	Hip geometry (Shaft Width, Women), multivariable adjusted	http://www.ncbi.nlm.nih.gov/projects/gap/cgi-bin/analysis.cgi?id=pha001875
Aging	BoneAging	ShaftW1m	Hip geometry (Shaft Width, Men), multivariable adjusted	http://www.ncbi.nlm.nih.gov/projects/gap/cgi-bin/analysis.cgi?id=pha001877
Aging	BoneAging	ShaftWf	Hip geometry (Shaft Width, Women), age-adjusted	http://www.ncbi.nlm.nih.gov/projects/gap/cgi-bin/analysis.cgi?id=pha001879

Group	Category	Trait Label	Name	FBAT Link
Aging	BoneAging	ShaftWm	Hip geometry (Shaft Width, Men), age-adjusted	http://www.ncbi.nlm.nih.gov/projects/gap/cgi-bin/analysis.cgi?id=pha001881
Aging	BoneAging	ShaftZ1R	Hip geometry (Shaft Section Modulus), multivariable adjusted	http://www.ncbi.nlm.nih.gov/projects/gap/cgi-bin/analysis.cgi?id=pha001883
Aging	BoneAging	ShaftZ1rf	Hip geometry (Shaft Section Modulus, Women), multivariable adjusted	http://www.ncbi.nlm.nih.gov/projects/gap/cgi-bin/analysis.cgi?id=pha001885
Aging	BoneAging	ShaftZ1rm	Hip geometry (Shaft Section Modulus, Men), multivariable adjusted	http://www.ncbi.nlm.nih.gov/projects/gap/cgi-bin/analysis.cgi?id=pha001887
Aging	BoneAging	SOS	Bone Ultrasound speed measured by QUS, multivariable adjusted	http://www.ncbi.nlm.nih.gov/projects/gap/cgi-bin/analysis.cgi?id=pha001783
Aging	BoneAging	TRBMD	Trochanter BMD measured by DXA, multivariable adjusted	http://www.ncbi.nlm.nih.gov/projects/gap/cgi-bin/analysis.cgi?id=pha001785
Aging	BoneAging	TRBMDf	Troch Neck BMD measured by DXA (Women), multivariable adjusted	http://www.ncbi.nlm.nih.gov/projects/gap/cgi-bin/analysis.cgi?id=pha001889
Aging	BoneAging	TRBMDm	Troch Neck BMD measured by DXA (Men), multivariable adjusted	http://www.ncbi.nlm.nih.gov/projects/gap/cgi-bin/analysis.cgi?id=pha001891
Aging	BrainMRI	AFBV	Frontal lobe to intracranial volume ratio, multivariable adjusted	http://www.ncbi.nlm.nih.gov/projects/gap/cgi-bin/analysis.cgi?id=pha001893
Aging	BrainMRI	AHPV	Hippocampal to intracranial volume ratio, multivariable adjusted	http://www.ncbi.nlm.nih.gov/projects/gap/cgi-bin/analysis.cgi?id=pha001895
Aging	BrainMRI	AHPVapoe1	Hippocampal to intracranial volume ratio, multivariable with APOE	http://www.ncbi.nlm.nih.gov/projects/gap/cgi-bin/analysis.cgi?id=pha001929
Aging	BrainMRI	AHPVr	Hippocampal to total cerebral volume ratio, multivariable adjusted	http://www.ncbi.nlm.nih.gov/projects/gap/cgi-bin/analysis.cgi?id=pha001897
Aging	BrainMRI	AHPVrapoe1	Hippocampal to total cerebral volume ratio, multivariable with APOE	http://www.ncbi.nlm.nih.gov/projects/gap/cgi-bin/analysis.cgi?id=pha001931
Aging	BrainMRI	ALLV	Log lateral ventricular to intracranial volume ratio, multivariable adjusted	http://www.ncbi.nlm.nih.gov/projects/gap/cgi-bin/analysis.cgi?id=pha001899
Aging	BrainMRI	ALTHBV	Log temporal horn to intracranial volume ratio, multivariable adjusted	http://www.ncbi.nlm.nih.gov/projects/gap/cgi-bin/analysis.cgi?id=pha001901
Aging	BrainMRI	ALWMHIVapoe1	Log white matter hyperintensity to cranial ratio, multivariable with APOE	http://www.ncbi.nlm.nih.gov/projects/gap/cgi-bin/analysis.cgi?id=pha001933
Aging	BrainMRI	AOBV	Occipital lobe to intracranial volume ratio, multivariable adjusted	http://www.ncbi.nlm.nih.gov/projects/gap/cgi-bin/analysis.cgi?id=pha001903
Aging	BrainMRI	APBV	Parietal lobe to intracranial volume ratio, multivariable adjusted	http://www.ncbi.nlm.nih.gov/projects/gap/cgi-bin/analysis.cgi?id=pha001905
Aging	BrainMRI	ATBV	Temporal lobe to intracranial volume ratio, multivariable adjusted	http://www.ncbi.nlm.nih.gov/projects/gap/cgi-bin/analysis.cgi?id=pha001907
Aging	BrainMRI	ATCBV	Total cerebral brain to intracranial volume ratio, multivariable adjusted	http://www.ncbi.nlm.nih.gov/projects/gap/cgi-bin/analysis.cgi?id=pha001909
Aging	BrainMRI	ATCBVapoe1	Total cerebral brain to intracranial volume ratio, multivariable with APOE	http://www.ncbi.nlm.nih.gov/projects/gap/cgi-bin/analysis.cgi?id=pha001935
Aging	BrainMRI	CFBV	Frontal lobe to intracranial volume ratio, age and sex adjusted	http://www.ncbi.nlm.nih.gov/projects/gap/cgi-bin/analysis.cgi?id=pha001911
Aging	BrainMRI	CHPV	Hippocampal to intracranial volume ratio, age and sex adjusted	http://www.ncbi.nlm.nih.gov/projects/gap/cgi-bin/analysis.cgi?id=pha001913
Aging	BrainMRI	CHPVr	Hippocampal to total cerebral volume ratio, age and sex adjusted	http://www.ncbi.nlm.nih.gov/projects/gap/cgi-bin/analysis.cgi?id=pha001915
Aging	BrainMRI	CLLV	Log lateral ventricular to intracranial volume ratio, age and sex adjusted	http://www.ncbi.nlm.nih.gov/projects/gap/cgi-bin/analysis.cgi?id=pha001917
Aging	BrainMRI	CLTHBV	Log temporal horn to intracranial volume ratio, age and sex adjusted	http://www.ncbi.nlm.nih.gov/projects/gap/cgi-bin/analysis.cgi?id=pha001919
Aging	BrainMRI	COBV	Occipital lobe to intracranial volume ratio, age and sex adjusted	http://www.ncbi.nlm.nih.gov/projects/gap/cgi-bin/analysis.cgi?id=pha001921
Aging	BrainMRI	CPBV	Parietal lobe to intracranial volume ratio, age and sex adjusted	http://www.ncbi.nlm.nih.gov/projects/gap/cgi-bin/analysis.cgi?id=pha001923
Aging	BrainMRI	CTBV	Temporal lobe to intracranial volume ratio, age and sex adjusted	http://www.ncbi.nlm.nih.gov/projects/gap/cgi-bin/analysis.cgi?id=pha001925
Aging	BrainMRI	CTCBV	Total cerebral brain to intracranial volume ratio, age and sex adjusted	http://www.ncbi.nlm.nih.gov/projects/gap/cgi-bin/analysis.cgi?id=pha001927
Aging	CognitiveFunction	F1	Visual memory composite score, multivariable adjusted	http://www.ncbi.nlm.nih.gov/projects/gap/cgi-bin/analysis.cgi?id=pha001937
Aging	CognitiveFunction	F2	Visuospatial memory & organization, multivariable adjusted	http://www.ncbi.nlm.nih.gov/projects/gap/cgi-bin/analysis.cgi?id=pha001939
Aging	CognitiveFunction	F3	Visual scanning & motor speed, multivariable adjusted	http://www.ncbi.nlm.nih.gov/projects/gap/cgi-bin/analysis.cgi?id=pha001941
Aging	CognitiveFunction	MMSE5MVX	MMSE score, offspring exam 5, multivariable- adjusted	http://www.ncbi.nlm.nih.gov/projects/gap/cgi-bin/analysis.cgi?id=pha001949
Aging	CognitiveFunction	MMSE5to7MVX	average MMSE score, offspring exams 5 & 7, multivariable-adjusted	http://www.ncbi.nlm.nih.gov/projects/gap/cgi-bin/analysis.cgi?id=pha001951
Aging	CognitiveFunction	MMSE5to7X	average MMSE score, offspring exam 5 & 7, age-adjusted	http://www.ncbi.nlm.nih.gov/projects/gap/cgi-bin/analysis.cgi?id=pha001953
Aging	CognitiveFunction	MMSE5X	MMSE score, offspring exam 5, age-adjusted	http://www.ncbi.nlm.nih.gov/projects/gap/cgi-bin/analysis.cgi?id=pha001955
Aging	CognitiveFunction	MMSE65MVX	MMSE score at age 65, offspring & cohort pooled, multivariable-adjusted	http://www.ncbi.nlm.nih.gov/projects/gap/cgi-bin/analysis.cgi?id=pha001957
Aging	CognitiveFunction	MMSE65X	MMSE score at age 65, offspring & cohort pooled, birth cohort-adjusted	http://www.ncbi.nlm.nih.gov/projects/gap/cgi-bin/analysis.cgi?id=pha001959
Aging	CognitiveFunction	MMSE7MV	MMSE score, offspring exam 7, multivariable- adjusted	http://www.ncbi.nlm.nih.gov/projects/gap/cgi-bin/analysis.cgi?id=pha002836
Aging	CognitiveFunction	MMSE7X	MMSE score, offspring exam 7, age-adjusted	http://www.ncbi.nlm.nih.gov/projects/gap/cgi-bin/analysis.cgi?id=pha001961
Aging	CognitiveFunction	Nam	Boston Naming Test score without cues, multivariable adjusted	http://www.ncbi.nlm.nih.gov/projects/gap/cgi-bin/analysis.cgi?id=pha001943
Aging	CognitiveFunction	Sim	Similarities raw score, multivariable adjusted	http://www.ncbi.nlm.nih.gov/projects/gap/cgi-bin/analysis.cgi?id=pha001945
Aging	CognitiveFunction	WRAT	Wide Range Achievement Test, multivariable adjusted	http://www.ncbi.nlm.nih.gov/projects/gap/cgi-bin/analysis.cgi?id=pha001947
Aging	Hearing	SPTA	Pure tone audiometry average over medium frequencies, age and sex adjusted	http://www.ncbi.nlm.nih.gov/projects/gap/cgi-bin/analysis.cgi?id=pha001963
Aging	Hearing	SPTAHI	Pure tone audiometry average over high frequencies, age and sex adjusted	http://www.ncbi.nlm.nih.gov/projects/gap/cgi-bin/analysis.cgi?id=pha001965
Aging	Hearing	SPTALO	Pure tone audiometry average over low frequencies, age and sex adjusted	http://www.ncbi.nlm.nih.gov/projects/gap/cgi-bin/analysis.cgi?id=pha001967
Aging	Morbidity-freesurvival	Morbidityfree65MVX	morbidity-free survival at age 65, offspring & cohort pooled, multivariable-adjusted	http://www.ncbi.nlm.nih.gov/projects/gap/cgi-bin/analysis.cgi?id=pha001969
Aging	Morbidity-freesurvival	Morbidityfree65X	morbidity-free survival at age 65, offspring & cohort pooled, birth cohort- adjusted	http://www.ncbi.nlm.nih.gov/projects/gap/cgi-bin/analysis.cgi?id=pha001971
Aging	PhysicalDisability	Handgrip727X	hand grip, offspring exam 7 & cohort exam 27	http://www.ncbi.nlm.nih.gov/projects/gap/cgi-bin/analysis.cgi?id=pha001977
Aging	PhysicalDisability	Handgrip7X	hand grip, offspring exam 7	http://www.ncbi.nlm.nih.gov/projects/gap/cgi-bin/analysis.cgi?id=pha001979
Aging	PhysicalDisability	Walkingspeed727X	walking speed, offspring exam 7 & cohort exam 27	http://www.ncbi.nlm.nih.gov/projects/gap/cgi-bin/analysis.cgi?id=pha001981
Aging	PhysicalDisability	Walkingspeed7X	walking speed, offspring exam 7	http://www.ncbi.nlm.nih.gov/projects/gap/cgi-bin/analysis.cgi?id=pha001983
Aging	ReproductiveTraits	MenoageMVX	age at natural menopause, adjusted	http://www.ncbi.nlm.nih.gov/projects/gap/cgi-bin/analysis.cgi?id=pha001985
Aging	ReproductiveTraits	MenoageX	age at natural menopause, crude	http://www.ncbi.nlm.nih.gov/projects/gap/cgi-bin/analysis.cgi?id=pha001987
Aging	Survival	DeathageMV	age at death, offspring & cohort pooled, multivariable-adjusted	http://www.ncbi.nlm.nih.gov/projects/gap/cgi-bin/analysis.cgi?id=pha001989

Group	Category	Trait Label	Name	FBAT Link
Aging	Survival	DeathageX	age at death, offspring & cohort pooled, adjusted for birth cohort	http://www.ncbi.nlm.nih.gov/projects/gap/cgi-bin/analysis.cgi?id=pha001991
Aging	Survival	DeathpastALEMV	death past average life expectancy, offspring & cohort pooled, multivariable-adjusted	http://www.ncbi.nlm.nih.gov/projects/gap/cgi-bin/analysis.cgi?id=pha001993
Aging	Survival	DeathpastALEX	death past average life expectancy, offspring & cohort pooled, birth cohort adjusted	http://www.ncbi.nlm.nih.gov/projects/gap/cgi-bin/analysis.cgi?id=pha001995
BloodPressure	Bloodpressure	DBP17AVGIMPAS	diastolic BP average age-sex-adjusted residual exams 1-7	http://www.ncbi.nlm.nih.gov/projects/gap/cgi-bin/analysis.cgi?id=pha001417
BloodPressure	Bloodpressure	DBP17AVGIMPMV	diastolic BP average multivariable-adjusted residual exams 1-7	http://www.ncbi.nlm.nih.gov/projects/gap/cgi-bin/analysis.cgi?id=pha001419
BloodPressure	Bloodpressure	DBP1IMPMEANAS	diastolic BP exam 1, age- sex- adjusted	http://www.ncbi.nlm.nih.gov/projects/gap/cgi-bin/analysis.cgi?id=pha001421
BloodPressure	Bloodpressure	DBP1IMPMEANMV	diastolic BP exam 1, multivariable-adjusted	http://www.ncbi.nlm.nih.gov/projects/gap/cgi-bin/analysis.cgi?id=pha001423
BloodPressure	Bloodpressure	DBP2IMPMEANAS	diastolic BP exam 2, age- sex- adjusted	http://www.ncbi.nlm.nih.gov/projects/gap/cgi-bin/analysis.cgi?id=pha001425
BloodPressure	Bloodpressure	DBP2IMPMEANMV	diastolic BP exam 2, multivariable-adjusted	http://www.ncbi.nlm.nih.gov/projects/gap/cgi-bin/analysis.cgi?id=pha001427
BloodPressure	Bloodpressure	DBP3IMPMEANAS	diastolic BP exam 3, age- sex- adjusted	http://www.ncbi.nlm.nih.gov/projects/gap/cgi-bin/analysis.cgi?id=pha001429
BloodPressure	Bloodpressure	DBP3IMPMEANMV	diastolic BP exam 3, multivariable-adjusted	http://www.ncbi.nlm.nih.gov/projects/gap/cgi-bin/analysis.cgi?id=pha001431
BloodPressure	Bloodpressure	DBP4IMPMEANAS	diastolic BP exam 4, age- sex- adjusted	http://www.ncbi.nlm.nih.gov/projects/gap/cgi-bin/analysis.cgi?id=pha001433
BloodPressure	Bloodpressure	DBP4IMPMEANMV	diastolic BP exam 4, multivariable-adjusted	http://www.ncbi.nlm.nih.gov/projects/gap/cgi-bin/analysis.cgi?id=pha001435
BloodPressure	Bloodpressure	DBP5IMPMEANAS	diastolic BP exam 5, age- sex- adjusted	http://www.ncbi.nlm.nih.gov/projects/gap/cgi-bin/analysis.cgi?id=pha001437
BloodPressure	Bloodpressure	DBP5IMPMEANMV	diastolic BP exam 5, multivariable-adjusted	http://www.ncbi.nlm.nih.gov/projects/gap/cgi-bin/analysis.cgi?id=pha001439
BloodPressure	Bloodpressure	DBP6IMPMEANAS	diastolic BP exam 6, age- sex- adjusted	http://www.ncbi.nlm.nih.gov/projects/gap/cgi-bin/analysis.cgi?id=pha001441
BloodPressure	Bloodpressure	DBP6IMPMEANMV	diastolic BP exam 6, multivariable-adjusted	http://www.ncbi.nlm.nih.gov/projects/gap/cgi-bin/analysis.cgi?id=pha001443
BloodPressure	Bloodpressure	DBP7IMPMEANAS	diastolic BP exam 7, age- sex- adjusted	http://www.ncbi.nlm.nih.gov/projects/gap/cgi-bin/analysis.cgi?id=pha001445
BloodPressure	Bloodpressure	DBP7IMPMEANMV	diastolic BP exam 7, multivariable-adjusted	http://www.ncbi.nlm.nih.gov/projects/gap/cgi-bin/analysis.cgi?id=pha001447
BloodPressure	Bloodpressure	PP17AVGIMPAS	pulse pressure average age-sex-adjusted residual exams 1-7	http://www.ncbi.nlm.nih.gov/projects/gap/cgi-bin/analysis.cgi?id=pha001449
BloodPressure	Bloodpressure	PP17AVGIMPMV	pulse pressure average multivariable-adjusted residual exams 1-7	http://www.ncbi.nlm.nih.gov/projects/gap/cgi-bin/analysis.cgi?id=pha001451
BloodPressure	Bloodpressure	PP1IMPMEANAS	pulse pressure exam 1, age- sex- adjusted	http://www.ncbi.nlm.nih.gov/projects/gap/cgi-bin/analysis.cgi?id=pha001453
BloodPressure	Bloodpressure	PP1IMPMEANMV	pulse pressure exam 1, multivariable- adjusted	http://www.ncbi.nlm.nih.gov/projects/gap/cgi-bin/analysis.cgi?id=pha001455
BloodPressure	Bloodpressure	PP2IMPMEANAS	pulse pressure exam 2, age- sex- adjusted	http://www.ncbi.nlm.nih.gov/projects/gap/cgi-bin/analysis.cgi?id=pha001457
BloodPressure	Bloodpressure	PP2IMPMEANMV	pulse pressure exam 2, multivariable- adjusted	http://www.ncbi.nlm.nih.gov/projects/gap/cgi-bin/analysis.cgi?id=pha001459
BloodPressure	Bloodpressure	PP3IMPMEANAS	pulse pressure exam 3, age- sex- adjusted	http://www.ncbi.nlm.nih.gov/projects/gap/cgi-bin/analysis.cgi?id=pha001461
BloodPressure	Bloodpressure	PP3IMPMEANMV	pulse pressure exam 3, multivariable- adjusted	http://www.ncbi.nlm.nih.gov/projects/gap/cgi-bin/analysis.cgi?id=pha001463
BloodPressure	Bloodpressure	PP4IMPMEANAS	pulse pressure exam 4, age- sex- adjusted	http://www.ncbi.nlm.nih.gov/projects/gap/cgi-bin/analysis.cgi?id=pha001465
BloodPressure	Bloodpressure	PP4IMPMEANMV	pulse pressure exam 4, multivariable- adjusted	http://www.ncbi.nlm.nih.gov/projects/gap/cgi-bin/analysis.cgi?id=pha001467
BloodPressure	Bloodpressure	PP5IMPMEANAS	pulse pressure exam 5, age- sex- adjusted	http://www.ncbi.nlm.nih.gov/projects/gap/cgi-bin/analysis.cgi?id=pha001469
BloodPressure	Bloodpressure	PP5IMPMEANMV	pulse pressure exam 5, multivariable- adjusted	http://www.ncbi.nlm.nih.gov/projects/gap/cgi-bin/analysis.cgi?id=pha001471
BloodPressure	Bloodpressure	PP6IMPMEANAS	pulse pressure exam 6, age- sex- adjusted	http://www.ncbi.nlm.nih.gov/projects/gap/cgi-bin/analysis.cgi?id=pha001473
BloodPressure	Bloodpressure	PP6IMPMEANMV	pulse pressure exam 6, multivariable- adjusted	http://www.ncbi.nlm.nih.gov/projects/gap/cgi-bin/analysis.cgi?id=pha001475
BloodPressure	Bloodpressure	PP7IMPMEANAS	pulse pressure exam 7, age- sex- adjusted	http://www.ncbi.nlm.nih.gov/projects/gap/cgi-bin/analysis.cgi?id=pha001477
BloodPressure	Bloodpressure	PP7IMPMEANMV	pulse pressure exam 7, multivariable- adjusted	http://www.ncbi.nlm.nih.gov/projects/gap/cgi-bin/analysis.cgi?id=pha001479
BloodPressure	Bloodpressure	SBP17AVGIMPAS	systolic BP average age-sex-adjusted residual exams 1-7	http://www.ncbi.nlm.nih.gov/projects/gap/cgi-bin/analysis.cgi?id=pha001481
BloodPressure	Bloodpressure	SBP17AVGIMPMV	systolic BP average multivariable-adjusted residual exams 1-7	http://www.ncbi.nlm.nih.gov/projects/gap/cgi-bin/analysis.cgi?id=pha001483
BloodPressure	Bloodpressure	SBP1IMPMEANAS	systolic BP exam 1, age- sex- adjusted	http://www.ncbi.nlm.nih.gov/projects/gap/cgi-bin/analysis.cgi?id=pha001485
BloodPressure	Bloodpressure	SBP1IMPMEANMV	systolic BP exam 1, multivariable-adjusted	http://www.ncbi.nlm.nih.gov/projects/gap/cgi-bin/analysis.cgi?id=pha001487
BloodPressure	Bloodpressure	SBP2IMPMEANAS	systolic BP exam 2, age- sex- adjusted	http://www.ncbi.nlm.nih.gov/projects/gap/cgi-bin/analysis.cgi?id=pha001489
BloodPressure	Bloodpressure	SBP2IMPMEANMV	systolic BP exam 2, multivariable-adjusted	http://www.ncbi.nlm.nih.gov/projects/gap/cgi-bin/analysis.cgi?id=pha001491
BloodPressure	Bloodpressure	SBP3IMPMEANAS	systolic BP exam 3, age- sex- adjusted	http://www.ncbi.nlm.nih.gov/projects/gap/cgi-bin/analysis.cgi?id=pha001493
BloodPressure	Bloodpressure	SBP3IMPMEANMV	systolic BP exam 3, multivariable-adjusted	http://www.ncbi.nlm.nih.gov/projects/gap/cgi-bin/analysis.cgi?id=pha001495
BloodPressure	Bloodpressure	SBP4IMPMEANAS	systolic BP exam 4, age- sex- adjusted	http://www.ncbi.nlm.nih.gov/projects/gap/cgi-bin/analysis.cgi?id=pha001497
BloodPressure	Bloodpressure	SBP4IMPMEANMV	systolic BP exam 4, multivariable-adjusted	http://www.ncbi.nlm.nih.gov/projects/gap/cgi-bin/analysis.cgi?id=pha001499
BloodPressure	Bloodpressure	SBP5IMPMEANAS	systolic BP exam 5, age- sex- adjusted	http://www.ncbi.nlm.nih.gov/projects/gap/cgi-bin/analysis.cgi?id=pha001501
BloodPressure	Bloodpressure	SBP5IMPMEANMV	systolic BP exam 5, multivariable-adjusted	http://www.ncbi.nlm.nih.gov/projects/gap/cgi-bin/analysis.cgi?id=pha001503
BloodPressure	Bloodpressure	SBP6IMPMEANAS	systolic BP exam 6, age- sex- adjusted	http://www.ncbi.nlm.nih.gov/projects/gap/cgi-bin/analysis.cgi?id=pha001505
BloodPressure	Bloodpressure	SBP6IMPMEANMV	systolic BP exam 6, multivariable-adjusted	http://www.ncbi.nlm.nih.gov/projects/gap/cgi-bin/analysis.cgi?id=pha001507
BloodPressure	Bloodpressure	SBP7IMPMEANAS	systolic BP exam 7, age- sex- adjusted	http://www.ncbi.nlm.nih.gov/projects/gap/cgi-bin/analysis.cgi?id=pha001509
BloodPressure	Bloodpressure	SBP7IMPMEANMV	systolic BP exam 7, multivariable-adjusted	http://www.ncbi.nlm.nih.gov/projects/gap/cgi-bin/analysis.cgi?id=pha001511
BloodPressure	echocardiography	AOR26AVGAS	M-mode Echo aortic root diastolic diameter, avg age-sex resid, exams 2,4,5,6	http://www.ncbi.nlm.nih.gov/projects/gap/cgi-bin/analysis.cgi?id=pha001513
BloodPressure	echocardiography	AOR26AVGMV	M-mode Echo aortic root diastolic diameter, avg multivar resid, exams 2,4,5,6	http://www.ncbi.nlm.nih.gov/projects/gap/cgi-bin/analysis.cgi?id=pha001515
BloodPressure	echocardiography	AOR2AS	M-mode Echo aortic root diastolic diameter, exam 2, age-sex-adjusted	http://www.ncbi.nlm.nih.gov/projects/gap/cgi-bin/analysis.cgi?id=pha001517
BloodPressure	echocardiography	AOR2MV	M-mode Echo aortic root diastolic diameter, exam 2, multivariable-adjusted	http://www.ncbi.nlm.nih.gov/projects/gap/cgi-bin/analysis.cgi?id=pha001519
BloodPressure	echocardiography	AOR4AS	M-mode Echo aortic root diastolic diameter, exam 4, age-sex-adjusted	http://www.ncbi.nlm.nih.gov/projects/gap/cgi-bin/analysis.cgi?id=pha001521

Group	Category	Trait Label	Name	FBAT Link
BloodPressure	echocardiography	AOR4MV	M-mode Echo aortic root diastolic diameter, exam 4, multivariable-adjusted	http://www.ncbi.nlm.nih.gov/projects/gap/cgi-bin/analysis.cgi?id=pha001523
BloodPressure	echocardiography	AOR5AS	M-mode Echo aortic root diastolic diameter, exam 5, age-sex-adjusted	http://www.ncbi.nlm.nih.gov/projects/gap/cgi-bin/analysis.cgi?id=pha001525
BloodPressure	echocardiography	AOR5MV	M-mode Echo aortic root diastolic diameter, exam 5, multivariable-adjusted	http://www.ncbi.nlm.nih.gov/projects/gap/cgi-bin/analysis.cgi?id=pha001527
BloodPressure	echocardiography	AOR6AS	M-mode Echo aortic root diastolic diameter, exam 6, age-sex-adjusted	http://www.ncbi.nlm.nih.gov/projects/gap/cgi-bin/analysis.cgi?id=pha001529
BloodPressure	echocardiography	AOR6MV	M-mode Echo aortic root diastolic diameter, exam 6, multivariable-adjusted	http://www.ncbi.nlm.nih.gov/projects/gap/cgi-bin/analysis.cgi?id=pha001531
BloodPressure	echocardiography	LAD26AVGAS	M-mode Echo Left atrial systolic diameter, avg age-sex resid, exams 2,4,5,6	http://www.ncbi.nlm.nih.gov/projects/gap/cgi-bin/analysis.cgi?id=pha001533
BloodPressure	echocardiography	LAD26AVGMV	M-mode Echo Left atrial systolic diameter, avg multivar resid, exams 2,4,5,6	http://www.ncbi.nlm.nih.gov/projects/gap/cgi-bin/analysis.cgi?id=pha001535
BloodPressure	echocardiography	LAD2AS	M-mode Echo Left atrial systolic diameter, exam 2, age-sex-adjusted	http://www.ncbi.nlm.nih.gov/projects/gap/cgi-bin/analysis.cgi?id=pha001537
BloodPressure	echocardiography	LAD2MV	M-mode Echo Left atrial systolic diameter, exam 2, multivariable-adjusted	http://www.ncbi.nlm.nih.gov/projects/gap/cgi-bin/analysis.cgi?id=pha001539
BloodPressure	echocardiography	LAD4AS	M-mode Echo Left atrial systolic diameter, exam 4, age-sex-adjusted	http://www.ncbi.nlm.nih.gov/projects/gap/cgi-bin/analysis.cgi?id=pha001541
BloodPressure	echocardiography	LAD4MV	M-mode Echo Left atrial systolic diameter, exam 4, multivariable-adjusted	http://www.ncbi.nlm.nih.gov/projects/gap/cgi-bin/analysis.cgi?id=pha001543
BloodPressure	echocardiography	LAD5AS	M-mode Echo Left atrial systolic diameter, exam 5, age-sex-adjusted	http://www.ncbi.nlm.nih.gov/projects/gap/cgi-bin/analysis.cgi?id=pha001545
BloodPressure	echocardiography	LAD5MV	M-mode Echo Left atrial systolic diameter, exam 5, multivariable-adjusted	http://www.ncbi.nlm.nih.gov/projects/gap/cgi-bin/analysis.cgi?id=pha001547
BloodPressure	echocardiography	LAD6AS	M-mode Echo Left atrial systolic diameter, exam 6, age-sex-adjusted	http://www.ncbi.nlm.nih.gov/projects/gap/cgi-bin/analysis.cgi?id=pha001549
BloodPressure	echocardiography	LAD6MV	M-mode Echo Left atrial systolic diameter, exam 6, multivariable-adjusted	http://www.ncbi.nlm.nih.gov/projects/gap/cgi-bin/analysis.cgi?id=pha001551
BloodPressure	echocardiography	LVDD26AVGAS	M-mode Echo LV diastolic diameter, avg age-sex resid, exams 2,4,5,6	http://www.ncbi.nlm.nih.gov/projects/gap/cgi-bin/analysis.cgi?id=pha001553
BloodPressure	echocardiography	LVDD26AVGMV	M-mode Echo LV diastolic diameter, avg multivar resid, exams 2,4,5,6	http://www.ncbi.nlm.nih.gov/projects/gap/cgi-bin/analysis.cgi?id=pha001555
BloodPressure	echocardiography	LVDD2AS	M-mode Echo LV diastolic diameter, exam 2, age-sex-adjusted	http://www.ncbi.nlm.nih.gov/projects/gap/cgi-bin/analysis.cgi?id=pha001557
BloodPressure	echocardiography	LVDD2MV	M-mode Echo LV diastolic diameter, exam 2, multivariable-adjusted	http://www.ncbi.nlm.nih.gov/projects/gap/cgi-bin/analysis.cgi?id=pha001559
BloodPressure	echocardiography	LVDD4AS	M-mode Echo LV diastolic diameter, exam 4, age-sex-adjusted	http://www.ncbi.nlm.nih.gov/projects/gap/cgi-bin/analysis.cgi?id=pha001561
BloodPressure	echocardiography	LVDD4MV	M-mode Echo LV diastolic diameter, exam 4, multivariable-adjusted	http://www.ncbi.nlm.nih.gov/projects/gap/cgi-bin/analysis.cgi?id=pha001563
BloodPressure	echocardiography	LVDD5AS	M-mode Echo LV diastolic diameter, exam 5, age-sex-adjusted	http://www.ncbi.nlm.nih.gov/projects/gap/cgi-bin/analysis.cgi?id=pha001565
BloodPressure	echocardiography	LVDD5MV	M-mode Echo LV diastolic diameter, exam 5, multivariable-adjusted	http://www.ncbi.nlm.nih.gov/projects/gap/cgi-bin/analysis.cgi?id=pha001567
BloodPressure	echocardiography	LVDD6AS	M-mode Echo LV diastolic diameter, exam 6, age-sex-adjusted	http://www.ncbi.nlm.nih.gov/projects/gap/cgi-bin/analysis.cgi?id=pha001569
BloodPressure	echocardiography	LVDD6MV	M-mode Echo LV diastolic diameter, exam 6, multivariable-adjusted	http://www.ncbi.nlm.nih.gov/projects/gap/cgi-bin/analysis.cgi?id=pha001571
BloodPressure	echocardiography	LVDS26AVGAS	M-mode Echo LV systolic diameter, avg age-sex resid, exams 2,4,5,6	http://www.ncbi.nlm.nih.gov/projects/gap/cgi-bin/analysis.cgi?id=pha001573
BloodPressure	echocardiography	LVDS26AVGMV	M-mode Echo LV systolic diameter, avg multivar resid, exams 2,4,5,6	http://www.ncbi.nlm.nih.gov/projects/gap/cgi-bin/analysis.cgi?id=pha001575
BloodPressure	echocardiography	LVDS2AS	M-mode Echo LV systolic diameter, exam 2, age-sex-adjusted	http://www.ncbi.nlm.nih.gov/projects/gap/cgi-bin/analysis.cgi?id=pha001577
BloodPressure	echocardiography	LVDS2MV	M-mode Echo LV systolic diameter, exam 2, multivariable-adjusted	http://www.ncbi.nlm.nih.gov/projects/gap/cgi-bin/analysis.cgi?id=pha001579
BloodPressure	echocardiography	LVDS4AS	M-mode Echo LV systolic diameter, exam 4, age-sex-adjusted	http://www.ncbi.nlm.nih.gov/projects/gap/cgi-bin/analysis.cgi?id=pha001581
BloodPressure	echocardiography	LVDS4MV	M-mode Echo LV systolic diameter, exam 4, multivariable-adjusted	http://www.ncbi.nlm.nih.gov/projects/gap/cgi-bin/analysis.cgi?id=pha001583
BloodPressure	echocardiography	LVDS5AS	M-mode Echo LV systolic diameter, exam 5, age-sex-adjusted	http://www.ncbi.nlm.nih.gov/projects/gap/cgi-bin/analysis.cgi?id=pha001585
BloodPressure	echocardiography	LVDS5MV	M-mode Echo LV systolic diameter, exam 5, multivariable-adjusted	http://www.ncbi.nlm.nih.gov/projects/gap/cgi-bin/analysis.cgi?id=pha001587
BloodPressure	echocardiography	LVDS6AS	M-mode Echo LV systolic diameter, exam 6, age-sex-adjusted	http://www.ncbi.nlm.nih.gov/projects/gap/cgi-bin/analysis.cgi?id=pha001589
BloodPressure	echocardiography	LVDS6MV	M-mode Echo LV systolic diameter, exam 6, multivariable-adjusted	http://www.ncbi.nlm.nih.gov/projects/gap/cgi-bin/analysis.cgi?id=pha001591
BloodPressure	echocardiography	LVFS26AVGAS	M-mode Echo LV fractional shortening, avg age-sex resid, exams 2,4,5,6	http://www.ncbi.nlm.nih.gov/projects/gap/cgi-bin/analysis.cgi?id=pha001593
BloodPressure	echocardiography	LVFS26AVGMV	M-mode Echo LV fractional shortening, avg multivar resid, exams 2,4,5,6	http://www.ncbi.nlm.nih.gov/projects/gap/cgi-bin/analysis.cgi?id=pha001595
BloodPressure	echocardiography	LVFS2AS	M-mode Echo LV fractional shortening, exam 2, age-sex-adjusted	http://www.ncbi.nlm.nih.gov/projects/gap/cgi-bin/analysis.cgi?id=pha001597
BloodPressure	echocardiography	LVFS2MV	M-mode Echo LV fractional shortening, exam 2, multivariable-adjusted	http://www.ncbi.nlm.nih.gov/projects/gap/cgi-bin/analysis.cgi?id=pha001599
BloodPressure	echocardiography	LVFS4AS	M-mode Echo LV fractional shortening, exam 4, age-sex-adjusted	http://www.ncbi.nlm.nih.gov/projects/gap/cgi-bin/analysis.cgi?id=pha001601
BloodPressure	echocardiography	LVFS4MV	M-mode Echo LV fractional shortening, exam 4, multivariable-adjusted	http://www.ncbi.nlm.nih.gov/projects/gap/cgi-bin/analysis.cgi?id=pha001603
BloodPressure	echocardiography	LVFS5AS	M-mode Echo LV fractional shortening, exam 5, age-sex-adjusted	http://www.ncbi.nlm.nih.gov/projects/gap/cgi-bin/analysis.cgi?id=pha001605
BloodPressure	echocardiography	LVFS5MV	M-mode Echo LV fractional shortening, exam 5, multivariable-adjusted	http://www.ncbi.nlm.nih.gov/projects/gap/cgi-bin/analysis.cgi?id=pha001607
BloodPressure	echocardiography	LVFS6AS	M-mode Echo LV fractional shortening, exam 6, age-sex-adjusted	http://www.ncbi.nlm.nih.gov/projects/gap/cgi-bin/analysis.cgi?id=pha001609
BloodPressure	echocardiography	LVFS6MV	M-mode Echo LV fractional shortening, exam 6, multivariable-adjusted	http://www.ncbi.nlm.nih.gov/projects/gap/cgi-bin/analysis.cgi?id=pha001611
BloodPressure	echocardiography	LVM26AVGAS	M-mode Echo LV mass, avg age-sex resid, exams 2,4,5,6	http://www.ncbi.nlm.nih.gov/projects/gap/cgi-bin/analysis.cgi?id=pha001613
BloodPressure	echocardiography	LVM26AVGMV	M-mode Echo LV mass, avg multivar resid, exams 2,4,5,6	http://www.ncbi.nlm.nih.gov/projects/gap/cgi-bin/analysis.cgi?id=pha001615
BloodPressure	echocardiography	LVM2AS	M-mode Echo LV mass, exam 2, age-sex-adjusted	http://www.ncbi.nlm.nih.gov/projects/gap/cgi-bin/analysis.cgi?id=pha001617
BloodPressure	echocardiography	LVM2MV	M-mode Echo LV mass, exam 2, multivariable-adjusted	http://www.ncbi.nlm.nih.gov/projects/gap/cgi-bin/analysis.cgi?id=pha001619
BloodPressure	echocardiography	LVM4AS	M-mode Echo LV mass, exam 4, age-sex-adjusted	http://www.ncbi.nlm.nih.gov/projects/gap/cgi-bin/analysis.cgi?id=pha001621
BloodPressure	echocardiography	LVM4MV	M-mode Echo LV mass, exam 4, multivariable-adjusted	http://www.ncbi.nlm.nih.gov/projects/gap/cgi-bin/analysis.cgi?id=pha001623
BloodPressure	echocardiography	LVM5AS	M-mode Echo LV mass, exam 5, age-sex-adjusted	http://www.ncbi.nlm.nih.gov/projects/gap/cgi-bin/analysis.cgi?id=pha001625
BloodPressure	echocardiography	LVM5MV	M-mode Echo LV mass, exam 5, multivariable-adjusted	http://www.ncbi.nlm.nih.gov/projects/gap/cgi-bin/analysis.cgi?id=pha001627
BloodPressure	echocardiography	LVM6AS	M-mode Echo LV mass, exam 6, age-sex-adjusted	http://www.ncbi.nlm.nih.gov/projects/gap/cgi-bin/analysis.cgi?id=pha001629
BloodPressure	echocardiography	LVM6MV	M-mode Echo LV mass, exam 6, multivariable-adjusted	http://www.ncbi.nlm.nih.gov/projects/gap/cgi-bin/analysis.cgi?id=pha001631
BloodPressure	echocardiography	LVWT26AVGAS	M-mode Echo LV diastolic wall thickness, avg age-sex resid, exams 2,4,5,6	http://www.ncbi.nlm.nih.gov/projects/gap/cgi-bin/analysis.cgi?id=pha001633

Group	Category	Trait Label	Name	FBAT Link
BloodPressure	echocardiography	LVWT26AVGMV	M-mode Echo LV diastolic wall thickness, avg multivar resid, exams 2,4,5,6	http://www.ncbi.nlm.nih.gov/projects/gap/cgi-bin/analysis.cgi?id=pha001635
BloodPressure	echocardiography	LVWT2AS	M-mode Echo LV diastolic wall thickness, exam 2, age-sex-adjusted	http://www.ncbi.nlm.nih.gov/projects/gap/cgi-bin/analysis.cgi?id=pha001637
BloodPressure	echocardiography	LVWT2MV	M-mode Echo LV diastolic wall thickness, exam 2, multivariable-adjusted	http://www.ncbi.nlm.nih.gov/projects/gap/cgi-bin/analysis.cgi?id=pha001639
BloodPressure	echocardiography	LVWT4AS	M-mode Echo LV diastolic wall thickness, exam 4, age-sex-adjusted	http://www.ncbi.nlm.nih.gov/projects/gap/cgi-bin/analysis.cgi?id=pha001641
BloodPressure	echocardiography	LVWT4MV	M-mode Echo LV diastolic wall thickness, exam 4, multivariable-adjusted	http://www.ncbi.nlm.nih.gov/projects/gap/cgi-bin/analysis.cgi?id=pha001643
BloodPressure	echocardiography	LVWT5AS	M-mode Echo LV diastolic wall thickness, exam 5, age-sex-adjusted	http://www.ncbi.nlm.nih.gov/projects/gap/cgi-bin/analysis.cgi?id=pha001645
BloodPressure	echocardiography	LVWT5MV	M-mode Echo LV diastolic wall thickness, exam 5, multivariable-adjusted	http://www.ncbi.nlm.nih.gov/projects/gap/cgi-bin/analysis.cgi?id=pha001647
BloodPressure	echocardiography	LVWT6AS	M-mode Echo LV diastolic wall thickness, exam 6, age-sex-adjusted	http://www.ncbi.nlm.nih.gov/projects/gap/cgi-bin/analysis.cgi?id=pha001649
BloodPressure	echocardiography	LVWT6MV	M-mode Echo LV diastolic wall thickness, exam 6, multivariable-adjusted	http://www.ncbi.nlm.nih.gov/projects/gap/cgi-bin/analysis.cgi?id=pha001651
BloodPressure	Endothelialfunction	BASEFLOW7AS	Brachial artery Baseline flow velocity, exam 7, age-sex-adjusted	http://www.ncbi.nlm.nih.gov/projects/gap/cgi-bin/analysis.cgi?id=pha001653
BloodPressure	Endothelialfunction	BASEFLOW7MV	Brachial artery Baseline flow velocity, exam 7, multivariable-adjusted	http://www.ncbi.nlm.nih.gov/projects/gap/cgi-bin/analysis.cgi?id=pha001655
BloodPressure	Endothelialfunction	BASELINEDIAMETERAS7	baseline brachial artery diameter, exam 7, age and sex-adjusted	http://www.ncbi.nlm.nih.gov/projects/gap/cgi-bin/analysis.cgi?id=pha001657
BloodPressure	Endothelialfunction	BASELINEDIAMETERMV7	baseline brachial artery diameter, exam 7, multivariable-adjusted	http://www.ncbi.nlm.nih.gov/projects/gap/cgi-bin/analysis.cgi?id=pha001659
BloodPressure	Endothelialfunction	FMD7PCTAS	Brachial artery Flow mediated dilation, exam 7, age-adjusted	http://www.ncbi.nlm.nih.gov/projects/gap/cgi-bin/analysis.cgi?id=pha001661
BloodPressure	Endothelialfunction	FMD7PCTMV	Brachial artery Flow mediated dilation, exam 7, multivariable-adjusted	http://www.ncbi.nlm.nih.gov/projects/gap/cgi-bin/analysis.cgi?id=pha001663
BloodPressure	Endothelialfunction	HYPERFLOW7AS	Brachial artery hyperemic flow velocity, exam 7, age-sex-adjusted	http://www.ncbi.nlm.nih.gov/projects/gap/cgi-bin/analysis.cgi?id=pha001665
BloodPressure	Endothelialfunction	HYPERFLOW7MV	Brachial artery hyperemic flow velocity, exam 7, multivariable-adjusted	http://www.ncbi.nlm.nih.gov/projects/gap/cgi-bin/analysis.cgi?id=pha001667
BloodPressure	ExerciseTest	ETT2DBPREC3AS	Exercise recovery 3-min diastolic BP, exam 2, age-sex-adjusted	http://www.ncbi.nlm.nih.gov/projects/gap/cgi-bin/analysis.cgi?id=pha001669
BloodPressure	ExerciseTest	ETT2DBPREC3MV	Exercise recovery 3-min diastolic BP, exam 2, multivariable-adjusted	http://www.ncbi.nlm.nih.gov/projects/gap/cgi-bin/analysis.cgi?id=pha001671
BloodPressure	ExerciseTest	ETT2DBPSTG2AS	Exercise Stage 2 diastolic BP, exam 2, age-sex-adjusted	http://www.ncbi.nlm.nih.gov/projects/gap/cgi-bin/analysis.cgi?id=pha001673
BloodPressure	ExerciseTest	ETT2DBPSTG2MV	Exercise Stage 2 diastolic BP, exam 2, multivariable-adjusted	http://www.ncbi.nlm.nih.gov/projects/gap/cgi-bin/analysis.cgi?id=pha001675
BloodPressure	ExerciseTest	ETT2HRREC3AS	Exercise recovery 3-min heart rate, exam 2, age-sex-adjusted	http://www.ncbi.nlm.nih.gov/projects/gap/cgi-bin/analysis.cgi?id=pha001677
BloodPressure	ExerciseTest	ETT2HRREC3MV	Exercise recovery 3-min heart rate, exam 2, multivariable-adjusted	http://www.ncbi.nlm.nih.gov/projects/gap/cgi-bin/analysis.cgi?id=pha001679
BloodPressure	ExerciseTest	ETT2HRSTG2AS	Exercise Stage 2 heart rate, exam 2, age-sex-adjusted	http://www.ncbi.nlm.nih.gov/projects/gap/cgi-bin/analysis.cgi?id=pha001681
BloodPressure	ExerciseTest	ETT2HRSTG2MV	Exercise Stage 2 heart rate, exam 2, multivariable-adjusted	http://www.ncbi.nlm.nih.gov/projects/gap/cgi-bin/analysis.cgi?id=pha001683
BloodPressure	ExerciseTest	ETT2SBPREC3AS	Exercise recovery 3-min systolic BP, exam 2, age-sex-adjusted	http://www.ncbi.nlm.nih.gov/projects/gap/cgi-bin/analysis.cgi?id=pha001685
BloodPressure	ExerciseTest	ETT2SBPREC3MV	Exercise recovery 3-min systolic BP, exam 2, multivariable-adjusted	http://www.ncbi.nlm.nih.gov/projects/gap/cgi-bin/analysis.cgi?id=pha001687
BloodPressure	ExerciseTest	ETT2SBPSTG2AS	Exercise Stage 2 systolic BP, exam 2, age-sex-adjusted	http://www.ncbi.nlm.nih.gov/projects/gap/cgi-bin/analysis.cgi?id=pha001689
BloodPressure	ExerciseTest	ETT2SBPSTG2MV	Exercise Stage 2 systolic BP, exam 2, multivariable-adjusted	http://www.ncbi.nlm.nih.gov/projects/gap/cgi-bin/analysis.cgi?id=pha001691
BloodPressure	tonometry	AI7AS	augmentation index, exam 7, age-sex-adjusted	http://www.ncbi.nlm.nih.gov/projects/gap/cgi-bin/analysis.cgi?id=pha001693
BloodPressure	tonometry	AI7MV	augmentation index, exam 7, multivariable-adjusted	http://www.ncbi.nlm.nih.gov/projects/gap/cgi-bin/analysis.cgi?id=pha001695
BloodPressure	tonometry	CBPWV7AS	carotid-brachial pulse wave velocity, exam 7, age-sex-adjusted	http://www.ncbi.nlm.nih.gov/projects/gap/cgi-bin/analysis.cgi?id=pha001697
BloodPressure	tonometry	CBPWV7MV	carotid-brachial pulse wave velocity, exam 7, multivariable-adjusted	http://www.ncbi.nlm.nih.gov/projects/gap/cgi-bin/analysis.cgi?id=pha001699
BloodPressure	tonometry	CFPWV7AS	carotid-femoral pulse wave velocity, exam 7, age-sex-adjusted	http://www.ncbi.nlm.nih.gov/projects/gap/cgi-bin/analysis.cgi?id=pha001701
BloodPressure	tonometry	CFPWV7MV	carotid-femoral pulse wave velocity, exam 7, multivariable-adjusted	http://www.ncbi.nlm.nih.gov/projects/gap/cgi-bin/analysis.cgi?id=pha001703
BloodPressure	tonometry	CPP7AS	central pulse pressure, exam 7, age-sex-adjusted	http://www.ncbi.nlm.nih.gov/projects/gap/cgi-bin/analysis.cgi?id=pha001705
BloodPressure	tonometry	CPP7MV	central pulse pressure, exam 7, multivariable-adjusted	http://www.ncbi.nlm.nih.gov/projects/gap/cgi-bin/analysis.cgi?id=pha001707
BloodPressure	tonometry	CRPW7VAS	carotid-radial pulse wave velocity, exam 7, age-sex-adjusted	http://www.ncbi.nlm.nih.gov/projects/gap/cgi-bin/analysis.cgi?id=pha001709
BloodPressure	tonometry	CRPWV7MV	carotid-radial pulse wave velocity, exam 7, multivariable-adjusted	http://www.ncbi.nlm.nih.gov/projects/gap/cgi-bin/analysis.cgi?id=pha001711
BloodPressure	tonometry	DBP7BRAOSCAS	Diastolic BP Brachial oscillometric, exam 7, age-sex-adjusted	http://www.ncbi.nlm.nih.gov/projects/gap/cgi-bin/analysis.cgi?id=pha001713
BloodPressure	tonometry	DBP7BRAOSCMV	Diastolic BP Brachial oscillometric, exam 7, multivariable-adjusted	http://www.ncbi.nlm.nih.gov/projects/gap/cgi-bin/analysis.cgi?id=pha001715
BloodPressure	tonometry	FWDWAVE7AS	forward wave amplitude, exam 7, age-sex-adjusted	http://www.ncbi.nlm.nih.gov/projects/gap/cgi-bin/analysis.cgi?id=pha001717
BloodPressure	tonometry	FWDWAVE7MV	forward wave amplitude, exam 7, multivariable-adjusted	http://www.ncbi.nlm.nih.gov/projects/gap/cgi-bin/analysis.cgi?id=pha001719
BloodPressure	tonometry	INVCFPWV7AS	LV ejection time, exam 7, age-sex-adjusted	http://www.ncbi.nlm.nih.gov/projects/gap/cgi-bin/analysis.cgi?id=pha001721
BloodPressure	tonometry	INVCFPWV7MV	LV ejection time, exam 7, multivariable-adjusted	http://www.ncbi.nlm.nih.gov/projects/gap/cgi-bin/analysis.cgi?id=pha001723
BloodPressure	tonometry	MAP7AS	mean arterial pressure, exam 7, age-sex-adjusted	http://www.ncbi.nlm.nih.gov/projects/gap/cgi-bin/analysis.cgi?id=pha001725
BloodPressure	tonometry	MAP7MV	mean arterial pressure, exam 7, multivariable-adjusted	http://www.ncbi.nlm.nih.gov/projects/gap/cgi-bin/analysis.cgi?id=pha001727
BloodPressure	tonometry	PERAMPAP7AS	Peripheral amplification apparent, exam 7, age-sex-adjusted	http://www.ncbi.nlm.nih.gov/projects/gap/cgi-bin/analysis.cgi?id=pha001729
BloodPressure	tonometry	PERAMPAP7MV	Peripheral amplification apparent, exam 7, multivariable-adjusted	http://www.ncbi.nlm.nih.gov/projects/gap/cgi-bin/analysis.cgi?id=pha001731
BloodPressure	tonometry	PERAMPTRU7AS	Peripheral amplification true, exam 7, age-sex-adjusted	http://www.ncbi.nlm.nih.gov/projects/gap/cgi-bin/analysis.cgi?id=pha001733
BloodPressure	tonometry	PERAMPTRU7MV	Peripheral amplification true, exam 7, multivariable-adjusted	http://www.ncbi.nlm.nih.gov/projects/gap/cgi-bin/analysis.cgi?id=pha001735
BloodPressure	tonometry	PP7BRAOSCAS	Pulse pressure Brachial oscillometric, exam 7, age-sex-adjusted	http://www.ncbi.nlm.nih.gov/projects/gap/cgi-bin/analysis.cgi?id=pha001737
BloodPressure	tonometry	PP7BRAOSCMV	Pulse pressure Brachial oscillometric, exam 7, multivariable-adjusted	http://www.ncbi.nlm.nih.gov/projects/gap/cgi-bin/analysis.cgi?id=pha001739
BloodPressure	tonometry	REFWAVE7AS	reflected wave amplitude, exam 7, age-sex-adjusted	http://www.ncbi.nlm.nih.gov/projects/gap/cgi-bin/analysis.cgi?id=pha001741
BloodPressure	tonometry	REFWAVE7MV	reflected wave amplitude, exam 7, multivariable-adjusted	http://www.ncbi.nlm.nih.gov/projects/gap/cgi-bin/analysis.cgi?id=pha001743
BloodPressure	tonometry	RWTT7AS	reflected wave transit time, exam 7, age-sex-adjusted	http://www.ncbi.nlm.nih.gov/projects/gap/cgi-bin/analysis.cgi?id=pha001745

Online Table 2: Phenotypes Evaluated for Association using Family-based Analysis (FBAT)

Group	Category	Trait Label	Name	FBAT Link
BloodPressure	tonometry	RWTT7MV	reflected wave transit time, exam 7, multivariable-adjusted	http://www.ncbi.nlm.nih.gov/projects/gap/cgi-bin/analysis.cgi?id=pha001747
BloodPressure	tonometry	SBP7BRAOSCAS	Systolic BP Brachial oscillometric, exam 7, age-sex-adjusted	http://www.ncbi.nlm.nih.gov/projects/gap/cgi-bin/analysis.cgi?id=pha001749
BloodPressure	tonometry	SBP7BRAOSCMV	Systolic BP Brachial oscillometric, exam 7, multivariable-adjusted	http://www.ncbi.nlm.nih.gov/projects/gap/cgi-bin/analysis.cgi?id=pha001751
CVDMiscTraits	Cancer	ALLCANCER1	all cancer, adjusted for age & sex	http://www.ncbi.nlm.nih.gov/projects/gap/cgi-bin/analysis.cgi?id=pha001331
CVDMiscTraits	Cancer	ALLCANCER2	all cancer, fully adjusted	http://www.ncbi.nlm.nih.gov/projects/gap/cgi-bin/analysis.cgi?id=pha001333
CVDMiscTraits	Cancer	BREASTCANCER1	breast cancer (women only), age-adjusted	http://www.ncbi.nlm.nih.gov/projects/gap/cgi-bin/analysis.cgi?id=pha001335
CVDMiscTraits	Cancer	BREASTCANCER2	breast cancer (women only), adjusted for age, parity, BMI at entry	http://www.ncbi.nlm.nih.gov/projects/gap/cgi-bin/analysis.cgi?id=pha001337
CVDMiscTraits	Cancer	PROSTATECANCER1	prostate cancer, adjusted for age at entry	http://www.ncbi.nlm.nih.gov/projects/gap/cgi-bin/analysis.cgi?id=pha001339
CVDMiscTraits	CVDbySOE	AllatheroCVD1D	Age- & sex- adjusted incident CHD, ABI, TIA, or IC	http://www.ncbi.nlm.nih.gov/projects/gap/cgi-bin/analysis.cgi?id=pha001341
CVDMiscTraits	CVDbySOE	AllatheroCVD2D	multivariable-adjusted incident CHD, ABI, TIA, or IC	http://www.ncbi.nlm.nih.gov/projects/gap/cgi-bin/analysis.cgi?id=pha001343
CVDMiscTraits	CVDbySOE	AllCHD1D	Age- & sex-adjusted incident MI, AP, CI, or CHD death	http://www.ncbi.nlm.nih.gov/projects/gap/cgi-bin/analysis.cgi?id=pha001345
CVDMiscTraits	CVDbySOE	AllCHD2D	multivariable-adjusted incident MI, AP, CI, or CHD death	http://www.ncbi.nlm.nih.gov/projects/gap/cgi-bin/analysis.cgi?id=pha001347
CVDMiscTraits	CVDbySOE	ALLCHF1D	Age- & sex-adjusted incident heart failure (free of MI)	http://www.ncbi.nlm.nih.gov/projects/gap/cgi-bin/analysis.cgi?id=pha001349
CVDMiscTraits	CVDbySOE	ALLCHF2D	multivariable-adjusted incident heart failure (free of MI)	http://www.ncbi.nlm.nih.gov/projects/gap/cgi-bin/analysis.cgi?id=pha001351
CVDMiscTraits	CVDbySOE	HardatheroCVD1D	Age- & sex-adjusted incident MI, CI, CHD death, or ABI	http://www.ncbi.nlm.nih.gov/projects/gap/cgi-bin/analysis.cgi?id=pha001357
CVDMiscTraits	CVDbySOE	HardatheroCVD2D	multivariable-adjusted incident MI, CI, CHD death, or ABI	http://www.ncbi.nlm.nih.gov/projects/gap/cgi-bin/analysis.cgi?id=pha001359
CVDMiscTraits	CVDbySOE	HardCHD1D	Age- & sex-adjusted incident MI, CI, or CHD death	http://www.ncbi.nlm.nih.gov/projects/gap/cgi-bin/analysis.cgi?id=pha001361
CVDMiscTraits	CVDbySOE	HardCHD2D	multivariable-adjusted incident MI, CI, or CHD death	http://www.ncbi.nlm.nih.gov/projects/gap/cgi-bin/analysis.cgi?id=pha001363
CVDMiscTraits	CVDbySOE	NoMICHF1D	Age- & sex-adjusted incident heart failure, censored at interval MI	http://www.ncbi.nlm.nih.gov/projects/gap/cgi-bin/analysis.cgi?id=pha001365
CVDMiscTraits	CVDbySOE	NoMICHF2D	multivariable-adjusted incident heart failure, censored at interval MI	http://www.ncbi.nlm.nih.gov/projects/gap/cgi-bin/analysis.cgi?id=pha001367
CVDMiscTraits	ECGtraits	PRAdjRRPOOL	Age & RR adjusted PR interval cohort ex 11 offspring ex 1	http://www.ncbi.nlm.nih.gov/projects/gap/cgi-bin/analysis.cgi?id=pha001369
CVDMiscTraits	ECGtraits	QTMEN	Age & RR adjusted QT interval men cohort ex 11 offspring ex 1	http://www.ncbi.nlm.nih.gov/projects/gap/cgi-bin/analysis.cgi?id=pha001371
CVDMiscTraits	ECGtraits	QTPOOL	Age & RR adjusted QT interval men & women cohort ex 11 offspring ex 1	http://www.ncbi.nlm.nih.gov/projects/gap/cgi-bin/analysis.cgi?id=pha001373
CVDMiscTraits	ECGtraits	QTWOMEN	Age & RR adjusted QT interval women cohort ex 11 offspring ex 1	http://www.ncbi.nlm.nih.gov/projects/gap/cgi-bin/analysis.cgi?id=pha001375
CVDMiscTraits	ECGtraits	RRMEN	Age adjusted RR interval men cohort ex 11 offspring ex 1	http://www.ncbi.nlm.nih.gov/projects/gap/cgi-bin/analysis.cgi?id=pha001377
CVDMiscTraits	ECGtraits	RRPOOL	Age adjusted RR interval men & women cohort ex 11 offspring ex 1	http://www.ncbi.nlm.nih.gov/projects/gap/cgi-bin/analysis.cgi?id=pha001379
CVDMiscTraits	ECGtraits	RRWOMEN	Age adjusted RR interval women cohort ex 11 offspring ex 1	http://www.ncbi.nlm.nih.gov/projects/gap/cgi-bin/analysis.cgi?id=pha001381
CVDMiscTraits	HRVtraits	HFHRV	Age & HR adjusted high freq power HRV exams 18 (cohort), 3 (offspring)	http://www.ncbi.nlm.nih.gov/projects/gap/cgi-bin/analysis.cgi?id=pha001383
CVDMiscTraits	HRVtraits	LFHFHRV	Age & HR adjusted low:high freq power HRV exams 18 (cohort), 3 (offspring)	http://www.ncbi.nlm.nih.gov/projects/gap/cgi-bin/analysis.cgi?id=pha001385
CVDMiscTraits	HRVtraits	LFHRV	Age & HR adjusted low frequency power HRV exams 18 (cohort), 3 (offspring)	http://www.ncbi.nlm.nih.gov/projects/gap/cgi-bin/analysis.cgi?id=pha001387
CVDMiscTraits	HRVtraits	PNN50HRV	Age & HR adjusted PNN50 HRV exams 18 (cohort), 3 (offspring)	http://www.ncbi.nlm.nih.gov/projects/gap/cgi-bin/analysis.cgi?id=pha001389
CVDMiscTraits	HRVtraits	SDNNHRV	Age & HR adjsuted SDNN HRV exams 18 (cohort), 3 (offspring)	http://www.ncbi.nlm.nih.gov/projects/gap/cgi-bin/analysis.cgi?id=pha001391
CVDMiscTraits	HRVtraits	TOTPWRHRV	Age & HR adjsuted total power HRV exams 18 (cohort), 3 (offspring)	http://www.ncbi.nlm.nih.gov/projects/gap/cgi-bin/analysis.cgi?id=pha001393
CVDMiscTraits	HRVtraits	VLFHRV	Age & HR adjusted very low freq power HRV exams 18 (cohort), 3 (offspring)	http://www.ncbi.nlm.nih.gov/projects/gap/cgi-bin/analysis.cgi?id=pha001395
CVDMiscTraits	Otherclinicaltraits	ALCOHOL1	Age- & sex-adjusted drinks/day ex 1	http://www.ncbi.nlm.nih.gov/projects/gap/cgi-bin/analysis.cgi?id=pha001397
CVDMiscTraits	Otherclinicaltraits	ALCOHOL2	Age- & sex-adjusted drinks/day across all exams	http://www.ncbi.nlm.nih.gov/projects/gap/cgi-bin/analysis.cgi?id=pha001399
CVDMiscTraits	Otherclinicaltraits	ALCOHOL3	Age- & sex-adjusted max drinks/day at any exam	http://www.ncbi.nlm.nih.gov/projects/gap/cgi-bin/analysis.cgi?id=pha001401
CVDMiscTraits	Otherclinicaltraits	GALLBLADDER	Age- & sex-adjusted gallbladder disease	http://www.ncbi.nlm.nih.gov/projects/gap/cgi-bin/analysis.cgi?id=pha001403
CVDMiscTraits	Otherclinicaltraits	GOUT	Age-, sex- & BMI-adjusted gout	http://www.ncbi.nlm.nih.gov/projects/gap/cgi-bin/analysis.cgi?id=pha001405
CVDMiscTraits	Othervasctraits	ALLAF1	Age- & sex-adjusted incident atrial fibrillation	http://www.ncbi.nlm.nih.gov/projects/gap/cgi-bin/analysis.cgi?id=pha001407
CVDMiscTraits	Othervasctraits	ALLAF2	multivariable-adjusted incident atrial fibrillation	http://www.ncbi.nlm.nih.gov/projects/gap/cgi-bin/analysis.cgi?id=pha001409
CVDMiscTraits	Othervasctraits	VARICOSEVEINS	age- & sex-adjusted varicose veins, men and women	http://www.ncbi.nlm.nih.gov/projects/gap/cgi-bin/analysis.cgi?id=pha001411
CVDMiscTraits	Othervasctraits	VARICOSEVEINSF	age- & #childbirths-adjusted varicose veins in women	http://www.ncbi.nlm.nih.gov/projects/gap/cgi-bin/analysis.cgi?id=pha001413
CVDMiscTraits	Othervasctraits	VARICOSEVEINSM	age-adjusted varicose veins in men	http://www.ncbi.nlm.nih.gov/projects/gap/cgi-bin/analysis.cgi?id=pha001415
ITBiomarkers	Hematological	Hctavg12as	Hematocrit, averaged residuals of exam 1 & 2, age & sex	http://www.ncbi.nlm.nih.gov/projects/gap/cgi-bin/analysis.cgi?id=pha000979
ITBiomarkers	Hematological	Hctavg12mv	Hematocrit, averaged residuals of exam 1 & 2, multivariable	http://www.ncbi.nlm.nih.gov/projects/gap/cgi-bin/analysis.cgi?id=pha000981
ITBiomarkers	Hematological	Hctex1as	Hematocrit exam 1 age & sex	http://www.ncbi.nlm.nih.gov/projects/gap/cgi-bin/analysis.cgi?id=pha000983
ITBiomarkers	Hematological	Hctex1mv	Hematocrit exam 1 multivariable	http://www.ncbi.nlm.nih.gov/projects/gap/cgi-bin/analysis.cgi?id=pha000985
ITBiomarkers	Hematological	Hctex2as	Hematocrit exam 2 age & sex	http://www.ncbi.nlm.nih.gov/projects/gap/cgi-bin/analysis.cgi?id=pha000987
ITBiomarkers	Hematological	Hctex2mv	Hematocrit exam 2 multivariable	http://www.ncbi.nlm.nih.gov/projects/gap/cgi-bin/analysis.cgi?id=pha000989
ITBiomarkers	Hematological	Hgbavg12as	Hemoglobin, averaged residuals of exam 1 & 2, age & sex	http://www.ncbi.nlm.nih.gov/projects/gap/cgi-bin/analysis.cgi?id=pha000991
ITBiomarkers	Hematological	Hgbavg12mv	Hemoglobin averaged residuals of exam 1 & 2, multivariable	http://www.ncbi.nlm.nih.gov/projects/gap/cgi-bin/analysis.cgi?id=pha000993
ITBiomarkers	Hematological	Hgbex1as	Hemoglobin exam 1 age & sex	http://www.ncbi.nlm.nih.gov/projects/gap/cgi-bin/analysis.cgi?id=pha000995
ITBiomarkers	Hematological	Hgbex1mv	Hemoglobin exam 1 multivariable	http://www.ncbi.nlm.nih.gov/projects/gap/cgi-bin/analysis.cgi?id=pha000997
ITBiomarkers	Hematological	Hgbex2as	Hemoglobin exam 2 age & sex	http://www.ncbi.nlm.nih.gov/projects/gap/cgi-bin/analysis.cgi?id=pha000999
ITBiomarkers	Hematological	Hgbex2mv	Hemoglobin exam 2 multivariable	http://www.ncbi.nlm.nih.gov/projects/gap/cgi-bin/analysis.cgi?id=pha001001

Group	Category	Trait Label	Name	FBAT Link
ITBiomarkers	Hematological	MCHCavg12as	Mean corpuscular hemoglobin concentration avg. residuals exam 1&2 age & sex	http://www.ncbi.nlm.nih.gov/projects/gap/cgi-bin/analysis.cgi?id=pha001003
ITBiomarkers	Hematological	MCHCavg12mv	Mean corpuscular hemoglobin concentration avg. residuals of exam 1&2 MV	http://www.ncbi.nlm.nih.gov/projects/gap/cgi-bin/analysis.cgi?id=pha001005
ITBiomarkers	Hematological	MCHCex1as	Mean corpuscular hemoglobin concentration exam 1 age & sex	http://www.ncbi.nlm.nih.gov/projects/gap/cgi-bin/analysis.cgi?id=pha001007
ITBiomarkers	Hematological	MCHCex1mv	Mean corpuscular hemoglobin concentration exam 1 multivariable	http://www.ncbi.nlm.nih.gov/projects/gap/cgi-bin/analysis.cgi?id=pha001009
ITBiomarkers	Hematological	MCHCex2as	Mean corpuscular hemoglobin concentration exam 2 age & sex	http://www.ncbi.nlm.nih.gov/projects/gap/cgi-bin/analysis.cgi?id=pha001011
ITBiomarkers	Hematological	MCHCex2mv	Mean corpuscular hemoglobin concentration exam 2 multivariable	http://www.ncbi.nlm.nih.gov/projects/gap/cgi-bin/analysis.cgi?id=pha001013
ITBiomarkers	Hematological	MCVavg12as	Mean corpuscular volume averaged residuals of exam 1 &2, age & sex	http://www.ncbi.nlm.nih.gov/projects/gap/cgi-bin/analysis.cgi?id=pha001015
ITBiomarkers	Hematological	MCVavg12mv	Mean corpuscular volume averaged residuals of exam 1 and 2, multivariable	http://www.ncbi.nlm.nih.gov/projects/gap/cgi-bin/analysis.cgi?id=pha001017
ITBiomarkers	Hematological	MCVex1as	Mean corpuscular volume exam 1 age & sex	http://www.ncbi.nlm.nih.gov/projects/gap/cgi-bin/analysis.cgi?id=pha001019
ITBiomarkers	Hematological	MCVex1mv	Mean corpuscular volume multivariable	http://www.ncbi.nlm.nih.gov/projects/gap/cgi-bin/analysis.cgi?id=pha001021
ITBiomarkers	Hematological	MCVex2as	Mean corpuscular volume exam 2 age & sex	http://www.ncbi.nlm.nih.gov/projects/gap/cgi-bin/analysis.cgi?id=pha001023
ITBiomarkers	Hematological	MCVex2mv	Mean corpuscular volume multivariable exam 2	http://www.ncbi.nlm.nih.gov/projects/gap/cgi-bin/analysis.cgi?id=pha001025
ITBiomarkers	Hematological	RBCCavg12as	Red blood cell count averaged residuals of exam 1 & 2, age & sex	http://www.ncbi.nlm.nih.gov/projects/gap/cgi-bin/analysis.cgi?id=pha001027
ITBiomarkers	Hematological	RBCCavg12mv	Red blood cell count averaged residuals exam 1 & 2, multivariable	http://www.ncbi.nlm.nih.gov/projects/gap/cgi-bin/analysis.cgi?id=pha001029
ITBiomarkers	Hematological	RBCCex1as	Red blood cell count exam 1 age & sex	http://www.ncbi.nlm.nih.gov/projects/gap/cgi-bin/analysis.cgi?id=pha001031
ITBiomarkers	Hematological	RBCCex1mv	Red blood cell count exam 1 multivariable	http://www.ncbi.nlm.nih.gov/projects/gap/cgi-bin/analysis.cgi?id=pha001033
ITBiomarkers	Hematological	RBCCex2as	Red blood cell count exam 2 age & sex	http://www.ncbi.nlm.nih.gov/projects/gap/cgi-bin/analysis.cgi?id=pha001035
ITBiomarkers	Hematological	RBCCex2asWIN	Red blood cell count exam 2 age & sex, Winsorized	http://www.ncbi.nlm.nih.gov/projects/gap/cgi-bin/analysis.cgi?id=pha001037
ITBiomarkers	Hematological	RBCCex2mv	Red blood cell count exam 2 multivariable	http://www.ncbi.nlm.nih.gov/projects/gap/cgi-bin/analysis.cgi?id=pha001039
ITBiomarkers	Hematological	RBCCex2mvWIN	Red blood cell count exam 2 multivariable, Winsorized	http://www.ncbi.nlm.nih.gov/projects/gap/cgi-bin/analysis.cgi?id=pha001041
ITBiomarkers	Hematological	WBCavg12as	White blood cell count averaged residuals of exam 1 & 2, age & sex	http://www.ncbi.nlm.nih.gov/projects/gap/cgi-bin/analysis.cgi?id=pha001043
ITBiomarkers	Hematological	WBCavg12mv	White blood cell count, averaged residuals of exam 1 & 2, multivariable	http://www.ncbi.nlm.nih.gov/projects/gap/cgi-bin/analysis.cgi?id=pha001045
ITBiomarkers	Hematological	WBCex1as	White blood cell count exam 1 age & sex, log transformed	http://www.ncbi.nlm.nih.gov/projects/gap/cgi-bin/analysis.cgi?id=pha001047
ITBiomarkers	Hematological	WBCex1mv	White blood cell count exam 1 multivariable, log transformed	http://www.ncbi.nlm.nih.gov/projects/gap/cgi-bin/analysis.cgi?id=pha001049
ITBiomarkers	Hematological	WBCex2as	White blood cell count exam 2 age & sex	http://www.ncbi.nlm.nih.gov/projects/gap/cgi-bin/analysis.cgi?id=pha001051
ITBiomarkers	Hematological	WBCex2mv	White blood cell count exam 2 multivariable	http://www.ncbi.nlm.nih.gov/projects/gap/cgi-bin/analysis.cgi?id=pha001053
ITBiomarkers	Hemostatic	dDimerex5as	D dimer at exam 6 age & sex, log transformed	http://www.ncbi.nlm.nih.gov/projects/gap/cgi-bin/analysis.cgi?id=pha001055
ITBiomarkers	Hemostatic	dDimerex5mv	D dimer at exam 6 multivariable, log transformed	http://www.ncbi.nlm.nih.gov/projects/gap/cgi-bin/analysis.cgi?id=pha001057
ITBiomarkers	Hemostatic	FibrinogenAvg567as	Fibrinogen averaged residuals of exam 5, 6 & 7 age & sex, log transformed	http://www.ncbi.nlm.nih.gov/projects/gap/cgi-bin/analysis.cgi?id=pha001059
ITBiomarkers	Hemostatic	FibrinogenAvg567mv	Fibrinogen averaged residuals exam 5, 6 & 7 multivariable, log transformed	http://www.ncbi.nlm.nih.gov/projects/gap/cgi-bin/analysis.cgi?id=pha001061
ITBiomarkers	Hemostatic	Fibrinogenex5as	Fibrinogen at exam 5 age & sex, log transformed	http://www.ncbi.nlm.nih.gov/projects/gap/cgi-bin/analysis.cgi?id=pha001063
ITBiomarkers	Hemostatic	Fibrinogenex5mv	Fibrinogen at exam 5 multivariable, log transformed	http://www.ncbi.nlm.nih.gov/projects/gap/cgi-bin/analysis.cgi?id=pha001065
ITBiomarkers	Hemostatic	Fibrinogenex6as	Fibrinogen at exam 6 age & sex, log transformed	http://www.ncbi.nlm.nih.gov/projects/gap/cgi-bin/analysis.cgi?id=pha001067
ITBiomarkers	Hemostatic	Fibrinogenex6mv	Fibrinogen at exam 6 multivariable, log transformed	http://www.ncbi.nlm.nih.gov/projects/gap/cgi-bin/analysis.cgi?id=pha001069
ITBiomarkers	Hemostatic	Fibrinogenex7as	Fibrinogen at exam 7 age & sex, log transformed	http://www.ncbi.nlm.nih.gov/projects/gap/cgi-bin/analysis.cgi?id=pha001071
ITBiomarkers	Hemostatic	Fibrinogenex7mv	Fibrinogen at exam 7 multivariable, log transformed	http://www.ncbi.nlm.nih.gov/projects/gap/cgi-bin/analysis.cgi?id=pha001073
ITBiomarkers	Hemostatic	FVIIex5as	Factor VII at exam 5 age & sex	http://www.ncbi.nlm.nih.gov/projects/gap/cgi-bin/analysis.cgi?id=pha001075
ITBiomarkers	Hemostatic	FVIIex5mv	Factor VII at exam 5 multivariable	http://www.ncbi.nlm.nih.gov/projects/gap/cgi-bin/analysis.cgi?id=pha001077
ITBiomarkers	Hemostatic	PAI1ex56Avgas	Log plasminogen Activator 1 avg. residuals exam 5& 6 age & sex	http://www.ncbi.nlm.nih.gov/projects/gap/cgi-bin/analysis.cgi?id=pha001079
ITBiomarkers	Hemostatic	PAI1ex56Avgmv	Log plasminogen Activator 1 avg. residuals exam 5 & 6 multivariable	http://www.ncbi.nlm.nih.gov/projects/gap/cgi-bin/analysis.cgi?id=pha001081
ITBiomarkers	Hemostatic	PAI1ex5as	Plasminogen Activator 1 at exam 5 age & sex, log transformed	http://www.ncbi.nlm.nih.gov/projects/gap/cgi-bin/analysis.cgi?id=pha001083
ITBiomarkers	Hemostatic	PAI1ex5mv	Plasminogen Activator 1 at exam 5 multivariable, log transformed	http://www.ncbi.nlm.nih.gov/projects/gap/cgi-bin/analysis.cgi?id=pha001085
ITBiomarkers	Hemostatic	PAI1ex6as	Plasminogen Activator 1 at exam 6 age & sex, log transformed	http://www.ncbi.nlm.nih.gov/projects/gap/cgi-bin/analysis.cgi?id=pha001087
ITBiomarkers	Hemostatic	PAI1ex6mv	Plasminogen Activator 1 at exam 6 multivariable, log transformed	http://www.ncbi.nlm.nih.gov/projects/gap/cgi-bin/analysis.cgi?id=pha001089
ITBiomarkers	Hemostatic	PlateletAgADPex5as	Platelet Aggregation to ADP exam 5, age & sex, log transformed	http://www.ncbi.nlm.nih.gov/projects/gap/cgi-bin/analysis.cgi?id=pha001091
ITBiomarkers	Hemostatic	PlateletAgADPex5mv	Log ADP platelet aggregation, exam 5, multivariable	http://www.ncbi.nlm.nih.gov/projects/gap/cgi-bin/analysis.cgi?id=pha001093
ITBiomarkers	Hemostatic	PlateletAgCollex5as	Platelet Aggregation to collagen, exam 5, age & sex, log transformed	http://www.ncbi.nlm.nih.gov/projects/gap/cgi-bin/analysis.cgi?id=pha001095
ITBiomarkers	Hemostatic	PlateletAgCollex5mv	Platelet Aggregation to collagen, exam 5, multivariable, log transformed	http://www.ncbi.nlm.nih.gov/projects/gap/cgi-bin/analysis.cgi?id=pha001097
ITBiomarkers	Hemostatic	PlateletAgEpiex5as	Platelet Aggregation to Epinephrine at exam 5 age & sex, log transformed	http://www.ncbi.nlm.nih.gov/projects/gap/cgi-bin/analysis.cgi?id=pha001099
ITBiomarkers	Hemostatic	PlateletAgEpiex5mv	Platelet Aggregation to Epinephrine exam 5 multivariable, log transformed	http://www.ncbi.nlm.nih.gov/projects/gap/cgi-bin/analysis.cgi?id=pha001101
ITBiomarkers	Hemostatic	tPAex5as	tPA Antigen at exam 5 age & sex, log transformed	http://www.ncbi.nlm.nih.gov/projects/gap/cgi-bin/analysis.cgi?id=pha001103
ITBiomarkers	Hemostatic	tPAex5mv	tPA Antigen at exam 5 multivariable, log transformed	http://www.ncbi.nlm.nih.gov/projects/gap/cgi-bin/analysis.cgi?id=pha001105
ITBiomarkers	Hemostatic	Viscoscityex5as	Viscosity I at exam 5 age & sex	http://www.ncbi.nlm.nih.gov/projects/gap/cgi-bin/analysis.cgi?id=pha001107
ITBiomarkers	Hemostatic	Viscoscityex5mv	Viscosity I at exam 5 multivariable	http://www.ncbi.nlm.nih.gov/projects/gap/cgi-bin/analysis.cgi?id=pha001109
ITBiomarkers	Hemostatic	wVWex5as	von Willebrand Factor at exam 5 age & sex	http://www.ncbi.nlm.nih.gov/projects/gap/cgi-bin/analysis.cgi?id=pha001111
ITBiomarkers	Hemostatic	wVWex5mv	von Willebrand Factor at exam 5 multivariable	http://www.ncbi.nlm.nih.gov/projects/gap/cgi-bin/analysis.cgi?id=pha001113

Group	Category	Trait Label	Name	FBAT Link
ITBiomarkers	Inflammation	CD40Lplasmaex7as	CD40Ligand plasma exam 7, age- & sex-adjusted, log transformed	http://www.ncbi.nlm.nih.gov/projects/gap/cgi-bin/analysis.cgi?id=pha001115
ITBiomarkers	Inflammation	CD40Lplasmaex7mv	CD40Ligand plasma exam 7 multivariable-adjusted, log transformed	http://www.ncbi.nlm.nih.gov/projects/gap/cgi-bin/analysis.cgi?id=pha001117
ITBiomarkers	Inflammation	CD40Lserumex7as	CD40Ligand serum exam 7, age- & sex-adjusted, log transformed	http://www.ncbi.nlm.nih.gov/projects/gap/cgi-bin/analysis.cgi?id=pha001119
ITBiomarkers	Inflammation	CD40Lserumex7mv	CD40Ligand serum, exam 7 multivariable-adjusted, log transformed	http://www.ncbi.nlm.nih.gov/projects/gap/cgi-bin/analysis.cgi?id=pha001121
ITBiomarkers	Inflammation	CRPavg267asB	Log C-reactive protein average exam 2, 6 & 7 age- & sex-adjusted	http://www.ncbi.nlm.nih.gov/projects/gap/cgi-bin/analysis.cgi?id=pha001171
ITBiomarkers	Inflammation	CRPavg267mvB	Log C-reactive protein average exam 2, 6 & 7, multivariable-adjusted	http://www.ncbi.nlm.nih.gov/projects/gap/cgi-bin/analysis.cgi?id=pha001173
ITBiomarkers	Inflammation	CRPex2asB	Log C-reactive protein exam 2, age- & sex-adjusted	http://www.ncbi.nlm.nih.gov/projects/gap/cgi-bin/analysis.cgi?id=pha001175
ITBiomarkers	Inflammation	CRPex2mvB	Log C-reactive protein exam 2, multivariable-adjusted	http://www.ncbi.nlm.nih.gov/projects/gap/cgi-bin/analysis.cgi?id=pha001177
ITBiomarkers	Inflammation	CRPex5as	Log C-reactive protein Hemagen Assay exam 5, age- & sex-adjusted	http://www.ncbi.nlm.nih.gov/projects/gap/cgi-bin/analysis.cgi?id=pha001123
ITBiomarkers	Inflammation	CRPex5mv	Log C-reactive protein Hemagen Assay exam 5, multivariable-adjusted	http://www.ncbi.nlm.nih.gov/projects/gap/cgi-bin/analysis.cgi?id=pha001125
ITBiomarkers	Inflammation	CRPex6as	Log C-reactive protein exam 6, age- & sex-adjusted	http://www.ncbi.nlm.nih.gov/projects/gap/cgi-bin/analysis.cgi?id=pha001127
ITBiomarkers	Inflammation	CRPex6mv	Log C-reactive protein exam 6, multivariable-adjusted	http://www.ncbi.nlm.nih.gov/projects/gap/cgi-bin/analysis.cgi?id=pha001129
ITBiomarkers	Inflammation	CRPex7as	Log C-reactive protein exam 7, age- & sex-adjusted	http://www.ncbi.nlm.nih.gov/projects/gap/cgi-bin/analysis.cgi?id=pha001131
ITBiomarkers	Inflammation	CRPex7mv	Log C-reactive protein exam 7, multivariable-adjusted	http://www.ncbi.nlm.nih.gov/projects/gap/cgi-bin/analysis.cgi?id=pha001133
ITBiomarkers	Inflammation	ICAM1ex7as	Log intercellular adhesion molecule-1 exam 7, age- & sex-adjusted	http://www.ncbi.nlm.nih.gov/projects/gap/cgi-bin/analysis.cgi?id=pha001135
ITBiomarkers	Inflammation	ICAM1ex7mv	Log intercellular adhesion molecule-1 exam 7, multivariable-adjusted	http://www.ncbi.nlm.nih.gov/projects/gap/cgi-bin/analysis.cgi?id=pha001137
ITBiomarkers	Inflammation	IL6ex7as	Log interleukin 6 Exam 7,age- & sex-adjusted	http://www.ncbi.nlm.nih.gov/projects/gap/cgi-bin/analysis.cgi?id=pha001139
ITBiomarkers	Inflammation	IL6ex7mv	Log interleukin 6 Exam 7,multivariable-adjusted	http://www.ncbi.nlm.nih.gov/projects/gap/cgi-bin/analysis.cgi?id=pha001141
ITBiomarkers	Inflammation	IsoCrUrinePex7as	Log urinary isoprostanes/creatinine exam 7, age- & sex-adjusted	http://www.ncbi.nlm.nih.gov/projects/gap/cgi-bin/analysis.cgi?id=pha001143
ITBiomarkers	Inflammation	IsoCrUrinePex7mv	Log urinary isoprostanes/creatinine exam 7, multivariable-adjusted	http://www.ncbi.nlm.nih.gov/projects/gap/cgi-bin/analysis.cgi?id=pha001145
ITBiomarkers	Inflammation	MCP1ex7as	Log monocyte chemoattractant protein 1 exam 7, age- & sex-adjusted	http://www.ncbi.nlm.nih.gov/projects/gap/cgi-bin/analysis.cgi?id=pha001147
ITBiomarkers	Inflammation	MCP1ex7mv	Log monocyte chemoattractant protein 1 exam 7, multivariable-adjusted	http://www.ncbi.nlm.nih.gov/projects/gap/cgi-bin/analysis.cgi?id=pha001149
ITBiomarkers	Inflammation	MPOex7as	Log myeloperoxidase exam 7, age- & sex-adjusted	http://www.ncbi.nlm.nih.gov/projects/gap/cgi-bin/analysis.cgi?id=pha001151
ITBiomarkers	Inflammation	MPOex7mv	Log myeloperoxidase exam 7, multivariable-adjusted	http://www.ncbi.nlm.nih.gov/projects/gap/cgi-bin/analysis.cgi?id=pha001153
ITBiomarkers	Inflammation	OPGex7as	Log osteoprotegerin exam 7, age- & sex-adjusted	http://www.ncbi.nlm.nih.gov/projects/gap/cgi-bin/analysis.cgi?id=pha001155
ITBiomarkers	Inflammation	OPGex7mv	Log osteoprotegerin exam 7, multivariable-adjusted	http://www.ncbi.nlm.nih.gov/projects/gap/cgi-bin/analysis.cgi?id=pha001157
ITBiomarkers	Inflammation	Pselectinex7as	Log P-selectin exam 7, age- & sex-adjusted	http://www.ncbi.nlm.nih.gov/projects/gap/cgi-bin/analysis.cgi?id=pha001159
ITBiomarkers	Inflammation	Pselectinex7mv	Log P-selectin exam 7, multivariable-adjusted	http://www.ncbi.nlm.nih.gov/projects/gap/cgi-bin/analysis.cgi?id=pha001161
ITBiomarkers	Inflammation	TNFAex7as	Log tumor necrosis factor alpha exam 7, age- & sex-adjusted	http://www.ncbi.nlm.nih.gov/projects/gap/cgi-bin/analysis.cgi?id=pha001163
ITBiomarkers	Inflammation	TNFAex7mv	Log tumor necrosis factor alpha exam 7, multivariable-adjusted	http://www.ncbi.nlm.nih.gov/projects/gap/cgi-bin/analysis.cgi?id=pha001165
ITBiomarkers	Inflammation	TNFRIIex7as	Log tumor necrosis factor receptor II exam 7, age- & sex-adjusted	http://www.ncbi.nlm.nih.gov/projects/gap/cgi-bin/analysis.cgi?id=pha001167
ITBiomarkers	Inflammation	TNFRIIex7mv	Log tumor necrosis factor receptor II exam 7 multivariable-adjusted	http://www.ncbi.nlm.nih.gov/projects/gap/cgi-bin/analysis.cgi?id=pha001169
ITBiomarkers	LFT	AlkPhosex2as	Log alkaline phosphatase exam 2 age- & sex-adjusted	http://www.ncbi.nlm.nih.gov/projects/gap/cgi-bin/analysis.cgi?id=pha001179
ITBiomarkers	LFT	AlkPhosex2mv	Log alkaline phosphatase exam 2, multivariable-adjusted	http://www.ncbi.nlm.nih.gov/projects/gap/cgi-bin/analysis.cgi?id=pha001181
ITBiomarkers	LFT	ALTex2as	Log alanine transaminase exam 2, age- & sex-adjusted	http://www.ncbi.nlm.nih.gov/projects/gap/cgi-bin/analysis.cgi?id=pha001183
ITBiomarkers	LFT	ALTex2mv	Log alanine transaminase exam 2; multivariable-adjusted	http://www.ncbi.nlm.nih.gov/projects/gap/cgi-bin/analysis.cgi?id=pha001185
ITBiomarkers	LFT	ASTex2as	Log aspartate aminotransferase exam, age- & sex-adjusted	http://www.ncbi.nlm.nih.gov/projects/gap/cgi-bin/analysis.cgi?id=pha001187
ITBiomarkers	LFT	ASTex2mv	Log aspartate aminotransferase exam 2 multivariable-adjusted	http://www.ncbi.nlm.nih.gov/projects/gap/cgi-bin/analysis.cgi?id=pha001189
ITBiomarkers	LFT	Bilirubinex2as	Log bilirubin exam 2, age- & sex-adjusted	http://www.ncbi.nlm.nih.gov/projects/gap/cgi-bin/analysis.cgi?id=pha001191
ITBiomarkers	LFT	Bilirubinex2mv	Log bilirubin exam 2 multivariable	http://www.ncbi.nlm.nih.gov/projects/gap/cgi-bin/analysis.cgi?id=pha001193
ITBiomarkers	LFT	GGTex2as	Log gamma-glutamyl transferase exam 2, age- & sex-adjusted	http://www.ncbi.nlm.nih.gov/projects/gap/cgi-bin/analysis.cgi?id=pha001195
ITBiomarkers	LFT	GGTex2mv	Log Gamma-glutamyl transferase exam 2, Multivariable,adjusted	http://www.ncbi.nlm.nih.gov/projects/gap/cgi-bin/analysis.cgi?id=pha001197
ITBiomarkers	Neurohumoral	ANPex6as	Log atrial natriuretic peptide exam 6, age- & sex-adjusted Tobit-model	http://www.ncbi.nlm.nih.gov/projects/gap/cgi-bin/analysis.cgi?id=pha001199
ITBiomarkers	Neurohumoral	ANPex6mv	Log atrial natriuretic peptide exam 6, multivariable-adjusted Tobit model	http://www.ncbi.nlm.nih.gov/projects/gap/cgi-bin/analysis.cgi?id=pha001201
ITBiomarkers	Neurohumoral	BNPex6as	Log brain natriuretic peptide exam 6, age- & sex-adjusted Tobit model	http://www.ncbi.nlm.nih.gov/projects/gap/cgi-bin/analysis.cgi?id=pha001203
ITBiomarkers	Neurohumoral	BNPex6mv	Log brain natriuretic peptide exam 6, multivariable-adjusted Tobit model	http://www.ncbi.nlm.nih.gov/projects/gap/cgi-bin/analysis.cgi?id=pha001205
ITBiomarkers	Vitamins	VitD25OHex6or7as	25(OH)-D exam 6 or 7, age & sex adjusted	http://www.ncbi.nlm.nih.gov/projects/gap/cgi-bin/analysis.cgi?id=pha001207
ITBiomarkers	Vitamins	VitD25OHex6or7mv	25(OH)-D exam 6 or 7, multivariable-adjusted; covariates from vit D exam	http://www.ncbi.nlm.nih.gov/projects/gap/cgi-bin/analysis.cgi?id=pha001209
ITBiomarkers	Vitamins	VitKPhylloqex6or7as	Vit K exam 6 or 7, age & sex adjusted, log transformed, no warfarin	http://www.ncbi.nlm.nih.gov/projects/gap/cgi-bin/analysis.cgi?id=pha001211
ITBiomarkers	Vitamins	VitKPhylloqex6or7mv	Vit K exam 6 or 7, multivariable-adjusted, log transformed, no warfarin	http://www.ncbi.nlm.nih.gov/projects/gap/cgi-bin/analysis.cgi?id=pha001213
ITBiomarkers	Vitamins	VitKPucOCex6or7as	%ucOC, exam 6 or 7, age, sex adjust, log transformed; no warfarin	http://www.ncbi.nlm.nih.gov/projects/gap/cgi-bin/analysis.cgi?id=pha001215
ITBiomarkers	Vitamins	VitKPucOCex6or7mv	%ucOC, exam 6 or 7, multivariable, log-transformed, no warfarin use	http://www.ncbi.nlm.nih.gov/projects/gap/cgi-bin/analysis.cgi?id=pha001217
Metabolic	Adiposity	allbmi1602	adj bmi, offsp 2 & cohort 16 exams	http://www.ncbi.nlm.nih.gov/projects/gap/cgi-bin/analysis.cgi?id=pha000101
Metabolic	Adiposity	allbmi1803	adj bmi, offsp 3 & cohort 18 exams	http://www.ncbi.nlm.nih.gov/projects/gap/cgi-bin/analysis.cgi?id=pha000103
Metabolic	Adiposity	allbmi1x01	adj bmi, offsp 1 & cohort 10 exams	http://www.ncbi.nlm.nih.gov/projects/gap/cgi-bin/analysis.cgi?id=pha000105
Metabolic	Adiposity	allbmi2004	adj bmi, offsp 4 & cohort 20 exams	http://www.ncbi.nlm.nih.gov/projects/gap/cgi-bin/analysis.cgi?id=pha000107

Group	Category	Trait Label	Name	FBAT Link
Metabolic	Adiposity	allbmi2205	adj bmi, offsp 5 & cohort 22 exams	http://www.ncbi.nlm.nih.gov/projects/gap/cgi-bin/analysis.cgi?id=pha000109
Metabolic	Adiposity	allbmi2406	adj bmi, offsp 6 & cohort 24 exams	http://www.ncbi.nlm.nih.gov/projects/gap/cgi-bin/analysis.cgi?id=pha000111
Metabolic	Adiposity	allbmi2607	adj bmi, offsp 7 & cohort 26 exams	http://www.ncbi.nlm.nih.gov/projects/gap/cgi-bin/analysis.cgi?id=pha000113
Metabolic	Adiposity	allchgbmi	adj bmi change, offsp 1-7 & cohort exams 10-26	http://www.ncbi.nlm.nih.gov/projects/gap/cgi-bin/analysis.cgi?id=pha000115
Metabolic	Adiposity	allchgwgt	adj weight change, offsp 1-7 & cohort exams 10-26	http://www.ncbi.nlm.nih.gov/projects/gap/cgi-bin/analysis.cgi?id=pha000117
Metabolic	Adiposity	allhgt1602	adj height, offsp 2 & cohort 16 exams	http://www.ncbi.nlm.nih.gov/projects/gap/cgi-bin/analysis.cgi?id=pha000119
Metabolic	Adiposity	allhgt1803	adj height, offsp 3 & cohort 18 exams	http://www.ncbi.nlm.nih.gov/projects/gap/cgi-bin/analysis.cgi?id=pha000121
Metabolic	Adiposity	allhgt1x01	adj height, offsp 1 & cohort 10 exams	http://www.ncbi.nlm.nih.gov/projects/gap/cgi-bin/analysis.cgi?id=pha000123
Metabolic	Adiposity	allhgt2004	adj height, offsp 4 & cohort 20 exams	http://www.ncbi.nlm.nih.gov/projects/gap/cgi-bin/analysis.cgi?id=pha000125
Metabolic	Adiposity	allhgt2205	adj height, offsp 5 & cohort 22 exams	http://www.ncbi.nlm.nih.gov/projects/gap/cgi-bin/analysis.cgi?id=pha000127
Metabolic	Adiposity	allhgt2406	adj height, offsp 6 & cohort 24 exams	http://www.ncbi.nlm.nih.gov/projects/gap/cgi-bin/analysis.cgi?id=pha000129
Metabolic	Adiposity	allhgt2607	adj height, offsp 7 & cohort 26 exams	http://www.ncbi.nlm.nih.gov/projects/gap/cgi-bin/analysis.cgi?id=pha000131
Metabolic	Adiposity	allmeanbmi	adj mean bmi, offsp 1-7 & cohort 10,16,18,20,22,24,26 exams	http://www.ncbi.nlm.nih.gov/projects/gap/cgi-bin/analysis.cgi?id=pha000133
Metabolic	Adiposity	allmeanhgt	adj mean height, offsp 1-7 & cohort 10,16,18,20,22,24,26 exams	http://www.ncbi.nlm.nih.gov/projects/gap/cgi-bin/analysis.cgi?id=pha000135
Metabolic	Adiposity	allmeanwgt	adj mean weight, offsp 1-7 & cohort 10,16,18,20,22,24,26 exams	http://www.ncbi.nlm.nih.gov/projects/gap/cgi-bin/analysis.cgi?id=pha000137
Metabolic	Adiposity	allochgwst	adj waist change, offsp 4 to 7	http://www.ncbi.nlm.nih.gov/projects/gap/cgi-bin/analysis.cgi?id=pha000139
Metabolic	Adiposity	allomeanwst	adj mean waist, offsp 4 to 7	http://www.ncbi.nlm.nih.gov/projects/gap/cgi-bin/analysis.cgi?id=pha000141
Metabolic	Adiposity	allowst0007	adj waist, offsp 7	http://www.ncbi.nlm.nih.gov/projects/gap/cgi-bin/analysis.cgi?id=pha000143
Metabolic	Adiposity	allrankbmi1602	rank adj bmi, offsp 2 & cohort 16 exams	http://www.ncbi.nlm.nih.gov/projects/gap/cgi-bin/analysis.cgi?id=pha000145
Metabolic	Adiposity	allrankbmi1803	rank adj bmi, offsp 3 & cohort 18 exams	http://www.ncbi.nlm.nih.gov/projects/gap/cgi-bin/analysis.cgi?id=pha000147
Metabolic	Adiposity	allrankbmi1x01	rank adj bmi, offsp 1 & cohort 10 exams	http://www.ncbi.nlm.nih.gov/projects/gap/cgi-bin/analysis.cgi?id=pha000149
Metabolic	Adiposity	allrankbmi2004	rank adj bmi, offsp 4 & cohort 20 exams	http://www.ncbi.nlm.nih.gov/projects/gap/cgi-bin/analysis.cgi?id=pha000151
Metabolic	Adiposity	allrankbmi2205	rank adj bmi, offsp 5 & cohort 22 exams	http://www.ncbi.nlm.nih.gov/projects/gap/cgi-bin/analysis.cgi?id=pha000153
Metabolic	Adiposity	allrankbmi2406	rank adj bmi, offsp 6 & cohort 24 exams	http://www.ncbi.nlm.nih.gov/projects/gap/cgi-bin/analysis.cgi?id=pha000155
Metabolic	Adiposity	allrankbmi2607	rank adj bmi, offsp 7 & cohort 26 exams	http://www.ncbi.nlm.nih.gov/projects/gap/cgi-bin/analysis.cgi?id=pha000157
Metabolic	Adiposity	allrankchgbmi	rank adj bmi change, offsp 1-7 & cohort exams 10-26	http://www.ncbi.nlm.nih.gov/projects/gap/cgi-bin/analysis.cgi?id=pha000159
Metabolic	Adiposity	allrankchgwgt	rank adj weight change, offsp 1-7 & cohort exams 10-26	http://www.ncbi.nlm.nih.gov/projects/gap/cgi-bin/analysis.cgi?id=pha000161
Metabolic	Adiposity	allrankmeanbmi	rank adj mean bmi, offsp 1-7 & cohort 10,16,18,20,22,24,26 exams	http://www.ncbi.nlm.nih.gov/projects/gap/cgi-bin/analysis.cgi?id=pha000163
Metabolic	Adiposity	allrankmeanwgt	rank adj mean weight, offsp 1-7 & cohort 10,16,18,20,22,24,26 exams	http://www.ncbi.nlm.nih.gov/projects/gap/cgi-bin/analysis.cgi?id=pha000165
Metabolic	Adiposity	allrankochgwst	rank adj waist change, offsp 4 to 7	http://www.ncbi.nlm.nih.gov/projects/gap/cgi-bin/analysis.cgi?id=pha000167
Metabolic	Adiposity	allrankomeanwst	rank adj mean waist, offsp 4 to 7	http://www.ncbi.nlm.nih.gov/projects/gap/cgi-bin/analysis.cgi?id=pha000169
Metabolic	Adiposity	allrankwgt1602	rank adj weight, offsp 2 & cohort 16 exams	http://www.ncbi.nlm.nih.gov/projects/gap/cgi-bin/analysis.cgi?id=pha000171
Metabolic	Adiposity	allrankwgt1803	rank adj weight, offsp 3 & cohort 18 exams	http://www.ncbi.nlm.nih.gov/projects/gap/cgi-bin/analysis.cgi?id=pha000173
Metabolic	Adiposity	allrankwgt1x01	rank adj weight, offsp 1 & cohort 10 exams	http://www.ncbi.nlm.nih.gov/projects/gap/cgi-bin/analysis.cgi?id=pha000175
Metabolic	Adiposity	allrankwgt2004	rank adj weight, offsp 4 & cohort 20 exams	http://www.ncbi.nlm.nih.gov/projects/gap/cgi-bin/analysis.cgi?id=pha000177
Metabolic	Adiposity	allrankwgt2205	rank adj weight, offsp 5 & cohort 22 exams	http://www.ncbi.nlm.nih.gov/projects/gap/cgi-bin/analysis.cgi?id=pha000179
Metabolic	Adiposity	allrankwgt2406	rank adj weight, offsp 6 & cohort 24 exams	http://www.ncbi.nlm.nih.gov/projects/gap/cgi-bin/analysis.cgi?id=pha000181
Metabolic	Adiposity	allrankwgt2607	rank adj weight, offsp 7 & cohort 26 exams	http://www.ncbi.nlm.nih.gov/projects/gap/cgi-bin/analysis.cgi?id=pha000183
Metabolic	Adiposity	allrankwst2306	rank adj waist, offsp 6 & cohort exam 23	http://www.ncbi.nlm.nih.gov/projects/gap/cgi-bin/analysis.cgi?id=pha000185
Metabolic	Adiposity	allwgt1602	adj weight, offsp 2 & cohort 16 exams	http://www.ncbi.nlm.nih.gov/projects/gap/cgi-bin/analysis.cgi?id=pha000187
Metabolic	Adiposity	allwgt1803	adj weight, offsp 3 & cohort 18 exams	http://www.ncbi.nlm.nih.gov/projects/gap/cgi-bin/analysis.cgi?id=pha000189
Metabolic	Adiposity	allwgt1x01	adj weight, offsp 1 & cohort 10 exams	http://www.ncbi.nlm.nih.gov/projects/gap/cgi-bin/analysis.cgi?id=pha000191
Metabolic	Adiposity	allwgt2004	adj weight, offsp 4 & cohort 20 exams	http://www.ncbi.nlm.nih.gov/projects/gap/cgi-bin/analysis.cgi?id=pha000193
Metabolic	Adiposity	allwgt2205	adj weight, offsp 5 & cohort 22 exams	http://www.ncbi.nlm.nih.gov/projects/gap/cgi-bin/analysis.cgi?id=pha000195
Metabolic	Adiposity	allwgt2406	adj weight, offsp 6 & cohort 24 exams	http://www.ncbi.nlm.nih.gov/projects/gap/cgi-bin/analysis.cgi?id=pha000197
Metabolic	Adiposity	allwgt2607	adj weight, offsp 7 & cohort 26 exams	http://www.ncbi.nlm.nih.gov/projects/gap/cgi-bin/analysis.cgi?id=pha000199
Metabolic	Adiposity	allwst2004	adj waist, offsp 4 & cohort 20 exams	http://www.ncbi.nlm.nih.gov/projects/gap/cgi-bin/analysis.cgi?id=pha000201
Metabolic	Adiposity	allwst2205	adj waist, offsp 5 & cohort 22 exams	http://www.ncbi.nlm.nih.gov/projects/gap/cgi-bin/analysis.cgi?id=pha000203
Metabolic	Adiposity	allwst2306	adj waist, offsp 6 & cohort exam 23	http://www.ncbi.nlm.nih.gov/projects/gap/cgi-bin/analysis.cgi?id=pha000205
Metabolic	Adiposity	fembmi1602	Female adj bmi, offsp 2 & cohort 16 exams	http://www.ncbi.nlm.nih.gov/projects/gap/cgi-bin/analysis.cgi?id=pha000207
Metabolic	Adiposity	fembmi1803	Female adj bmi, offsp 3 & cohort 18 exams	http://www.ncbi.nlm.nih.gov/projects/gap/cgi-bin/analysis.cgi?id=pha000209
Metabolic	Adiposity	fembmi1x01	Female adj bmi, offsp 1 & cohort 10 exams	http://www.ncbi.nlm.nih.gov/projects/gap/cgi-bin/analysis.cgi?id=pha000211
Metabolic	Adiposity	fembmi2004	Female adj bmi, offsp 4 & cohort 20 exams	http://www.ncbi.nlm.nih.gov/projects/gap/cgi-bin/analysis.cgi?id=pha000213
Metabolic	Adiposity	fembmi2205	Female adj bmi, offsp 5 & cohort 22 exams	http://www.ncbi.nlm.nih.gov/projects/gap/cgi-bin/analysis.cgi?id=pha000215
Metabolic	Adiposity	fembmi2406	Female adj bmi, offsp 6 & cohort 24 exams	http://www.ncbi.nlm.nih.gov/projects/gap/cgi-bin/analysis.cgi?id=pha000217
Metabolic	Adiposity	fembmi2607	Female adj bmi, offsp 7 & cohort 26 exams	http://www.ncbi.nlm.nih.gov/projects/gap/cgi-bin/analysis.cgi?id=pha000219

Group	Category	Trait Label	Name	FBAT Link
Metabolic	Adiposity	femchgbmi	Female adj bmi change, offsp 1-7 & cohort exams 10-26	http://www.ncbi.nlm.nih.gov/projects/gap/cgi-bin/analysis.cgi?id=pha000221
Metabolic	Adiposity	femchgwgt	Female adj weight change, offsp 1-7 & cohort exams 10-26	http://www.ncbi.nlm.nih.gov/projects/gap/cgi-bin/analysis.cgi?id=pha000223
Metabolic	Adiposity	femhgt1602	Female adj height, offsp 2 & cohort 16 exams	http://www.ncbi.nlm.nih.gov/projects/gap/cgi-bin/analysis.cgi?id=pha000225
Metabolic	Adiposity	femhgt1803	Female adj height, offsp 3 & cohort 18 exams	http://www.ncbi.nlm.nih.gov/projects/gap/cgi-bin/analysis.cgi?id=pha000227
Metabolic	Adiposity	femhgt1x01	Female adj height, offsp 1 & cohort 10 exams	http://www.ncbi.nlm.nih.gov/projects/gap/cgi-bin/analysis.cgi?id=pha000229
Metabolic	Adiposity	femhgt2004	Female adj height, offsp 4 & cohort 20 exams	http://www.ncbi.nlm.nih.gov/projects/gap/cgi-bin/analysis.cgi?id=pha000231
Metabolic	Adiposity	femhgt2205	Female adj height, offsp 5 & cohort 22 exams	http://www.ncbi.nlm.nih.gov/projects/gap/cgi-bin/analysis.cgi?id=pha000233
Metabolic	Adiposity	femhgt2406	Female adj height, offsp 6 & cohort 24 exams	http://www.ncbi.nlm.nih.gov/projects/gap/cgi-bin/analysis.cgi?id=pha000235
Metabolic	Adiposity	femhgt2607	Female adj height, offsp 7 & cohort 26 exams	http://www.ncbi.nlm.nih.gov/projects/gap/cgi-bin/analysis.cgi?id=pha000237
Metabolic	Adiposity	femmeanbmi	Female adj mean bmi, offsp 1-7 & cohort 10,16,18,20,22,24,26 exams	http://www.ncbi.nlm.nih.gov/projects/gap/cgi-bin/analysis.cgi?id=pha000239
Metabolic	Adiposity	femmeanhgt	Female adj mean height, offsp 1-7 & cohort 10,16,18,20,22,24,26 exams	http://www.ncbi.nlm.nih.gov/projects/gap/cgi-bin/analysis.cgi?id=pha000241
Metabolic	Adiposity	femmeanwgt	Female adj mean weight, offsp 1-7 & cohort 10,16,18,20,22,24,26 exams	http://www.ncbi.nlm.nih.gov/projects/gap/cgi-bin/analysis.cgi?id=pha000243
Metabolic	Adiposity	femochgwst	Female adj waist change, offsp 4 to 7	http://www.ncbi.nlm.nih.gov/projects/gap/cgi-bin/analysis.cgi?id=pha000245
Metabolic	Adiposity	femomeanwst	Female adj mean waist, offsp 4 to 7	http://www.ncbi.nlm.nih.gov/projects/gap/cgi-bin/analysis.cgi?id=pha000247
Metabolic	Adiposity	femowst0007	Female adj waist, offsp 7	http://www.ncbi.nlm.nih.gov/projects/gap/cgi-bin/analysis.cgi?id=pha000249
Metabolic	Adiposity	femrankbmi1602	Female rank adj bmi, offsp 2 & cohort 16 exams	http://www.ncbi.nlm.nih.gov/projects/gap/cgi-bin/analysis.cgi?id=pha000251
Metabolic	Adiposity	femrankbmi1803	Female rank adj bmi, offsp 3 & cohort 18 exams	http://www.ncbi.nlm.nih.gov/projects/gap/cgi-bin/analysis.cgi?id=pha000253
Metabolic	Adiposity	femrankbmi1x01	Female rank adj bmi, offsp 1 & cohort 10 exams	http://www.ncbi.nlm.nih.gov/projects/gap/cgi-bin/analysis.cgi?id=pha000255
Metabolic	Adiposity	femrankbmi2004	Female rank adj bmi, offsp 4 & cohort 20 exams	http://www.ncbi.nlm.nih.gov/projects/gap/cgi-bin/analysis.cgi?id=pha000257
Metabolic	Adiposity	femrankbmi2205	Female rank adj bmi, offsp 5 & cohort 22 exams	http://www.ncbi.nlm.nih.gov/projects/gap/cgi-bin/analysis.cgi?id=pha000259
Metabolic	Adiposity	femrankbmi2406	Female rank adj bmi, offsp 6 & cohort 24 exams	http://www.ncbi.nlm.nih.gov/projects/gap/cgi-bin/analysis.cgi?id=pha000261
Metabolic	Adiposity	femrankbmi2607	Female rank adj bmi, offsp 7 & cohort 26 exams	http://www.ncbi.nlm.nih.gov/projects/gap/cgi-bin/analysis.cgi?id=pha000263
Metabolic	Adiposity	femrankchgwgt	Female rank adj weight change, offsp 1-7 & cohort exams 10-26	http://www.ncbi.nlm.nih.gov/projects/gap/cgi-bin/analysis.cgi?id=pha000265
Metabolic	Adiposity	femrankmeanbmi	Female rank adj mean bmi, offsp 1-7 & cohort 10,16,18,20,22,24,26 exams	http://www.ncbi.nlm.nih.gov/projects/gap/cgi-bin/analysis.cgi?id=pha000267
Metabolic	Adiposity	femrankmeanwgt	Female rank adj mean weight, offsp 1-7 & cohort 10,16,18,20,22,24,26 exams	http://www.ncbi.nlm.nih.gov/projects/gap/cgi-bin/analysis.cgi?id=pha000269
Metabolic	Adiposity	femrankomeanwst	Female rank adj mean waist, offsp 4 to 7	http://www.ncbi.nlm.nih.gov/projects/gap/cgi-bin/analysis.cgi?id=pha000271
Metabolic	Adiposity	femrankwgt1602	Female rank adj weight, offsp 2 & cohort 16 exams	http://www.ncbi.nlm.nih.gov/projects/gap/cgi-bin/analysis.cgi?id=pha000273
Metabolic	Adiposity	femrankwgt1803	Female rank adj weight, offsp 3 & cohort 18 exams	http://www.ncbi.nlm.nih.gov/projects/gap/cgi-bin/analysis.cgi?id=pha000275
Metabolic	Adiposity	femrankwgt1x01	Female rank adj weight, offsp 1 & cohort 10 exams	http://www.ncbi.nlm.nih.gov/projects/gap/cgi-bin/analysis.cgi?id=pha000277
Metabolic	Adiposity	femrankwgt2004	Female rank adj weight, offsp 4 & cohort 20 exams	http://www.ncbi.nlm.nih.gov/projects/gap/cgi-bin/analysis.cgi?id=pha000279
Metabolic	Adiposity	femrankwgt2205	Female rank adj weight, offsp 5 & cohort 22 exams	http://www.ncbi.nlm.nih.gov/projects/gap/cgi-bin/analysis.cgi?id=pha000281
Metabolic	Adiposity	femrankwgt2406	Female rank adj weight, offsp 6 & cohort 24 exams	http://www.ncbi.nlm.nih.gov/projects/gap/cgi-bin/analysis.cgi?id=pha000283
Metabolic	Adiposity	femrankwgt2607	Female rank adj weight, offsp 7 & cohort 26 exams	http://www.ncbi.nlm.nih.gov/projects/gap/cgi-bin/analysis.cgi?id=pha000285
Metabolic	Adiposity	femwgt1602	Female adj weight, offsp 2 & cohort 16 exams	http://www.ncbi.nlm.nih.gov/projects/gap/cgi-bin/analysis.cgi?id=pha000287
Metabolic	Adiposity	femwgt1803	Female adj weight, offsp 3 & cohort 18 exams	http://www.ncbi.nlm.nih.gov/projects/gap/cgi-bin/analysis.cgi?id=pha000289
Metabolic	Adiposity	femwgt1x01	Female adj weight, offsp 1 & cohort 10 exams	http://www.ncbi.nlm.nih.gov/projects/gap/cgi-bin/analysis.cgi?id=pha000291
Metabolic	Adiposity	femwgt2004	Female adj weight, offsp 4 & cohort 20 exams	http://www.ncbi.nlm.nih.gov/projects/gap/cgi-bin/analysis.cgi?id=pha000293
Metabolic	Adiposity	femwgt2205	Female adj weight, offsp 5 & cohort 22 exams	http://www.ncbi.nlm.nih.gov/projects/gap/cgi-bin/analysis.cgi?id=pha000295
Metabolic	Adiposity	femwgt2406	Female adj weight, offsp 6 & cohort 24 exams	http://www.ncbi.nlm.nih.gov/projects/gap/cgi-bin/analysis.cgi?id=pha000297
Metabolic	Adiposity	femwgt2607	Female adj weight, offsp 7 & cohort 26 exams	http://www.ncbi.nlm.nih.gov/projects/gap/cgi-bin/analysis.cgi?id=pha000299
Metabolic	Adiposity	femwst2004	Female adj waist, offsp 4 & cohort 20 exams	http://www.ncbi.nlm.nih.gov/projects/gap/cgi-bin/analysis.cgi?id=pha000301
Metabolic	Adiposity	femwst2205	Female adj waist, offsp 5 & cohort 22 exams	http://www.ncbi.nlm.nih.gov/projects/gap/cgi-bin/analysis.cgi?id=pha000303
Metabolic	Adiposity	femwst2306	Female adj waist, offsp 6 & cohort exam 23	http://www.ncbi.nlm.nih.gov/projects/gap/cgi-bin/analysis.cgi?id=pha000305
Metabolic	Adiposity	malebmi1602	Male adj bmi, offsp 2 & cohort 16 exams	http://www.ncbi.nlm.nih.gov/projects/gap/cgi-bin/analysis.cgi?id=pha000307
Metabolic	Adiposity	malebmi1803	Male adj bmi, offsp 3 & cohort 18 exams	http://www.ncbi.nlm.nih.gov/projects/gap/cgi-bin/analysis.cgi?id=pha000309
Metabolic	Adiposity	malebmi1x01	Male adj bmi, offsp 1 & cohort 10 exams	http://www.ncbi.nlm.nih.gov/projects/gap/cgi-bin/analysis.cgi?id=pha000311
Metabolic	Adiposity	malebmi2004	Male adj bmi, offsp 4 & cohort 20 exams	http://www.ncbi.nlm.nih.gov/projects/gap/cgi-bin/analysis.cgi?id=pha000313
Metabolic	Adiposity	malebmi2205	Male adj bmi, offsp 5 & cohort 22 exams	http://www.ncbi.nlm.nih.gov/projects/gap/cgi-bin/analysis.cgi?id=pha000315
Metabolic	Adiposity	malebmi2406	Male adj bmi, offsp 6 & cohort 24 exams	http://www.ncbi.nlm.nih.gov/projects/gap/cgi-bin/analysis.cgi?id=pha000317
Metabolic	Adiposity	malebmi2607	Male adj bmi, offsp 7 & cohort 26 exams	http://www.ncbi.nlm.nih.gov/projects/gap/cgi-bin/analysis.cgi?id=pha000319
Metabolic	Adiposity	malechgbmi	Male adj bmi change, offsp 1-7 & cohort exams 10-26	http://www.ncbi.nlm.nih.gov/projects/gap/cgi-bin/analysis.cgi?id=pha000321
Metabolic	Adiposity	malechgwgt	Male adj weight change, offsp 1-7 & cohort exams 10-26	http://www.ncbi.nlm.nih.gov/projects/gap/cgi-bin/analysis.cgi?id=pha000323
Metabolic	Adiposity	malehgt1602	Male adj height, offsp 2 & cohort 16 exams	http://www.ncbi.nlm.nih.gov/projects/gap/cgi-bin/analysis.cgi?id=pha000325
Metabolic	Adiposity	malehgt1803	Male adj height, offsp 3 & cohort 18 exams	http://www.ncbi.nlm.nih.gov/projects/gap/cgi-bin/analysis.cgi?id=pha000327
Metabolic	Adiposity	malehgt1x01	Male adj height, offsp 1 & cohort 10 exams	http://www.ncbi.nlm.nih.gov/projects/gap/cgi-bin/analysis.cgi?id=pha000329
Metabolic	Adiposity	malehgt2004	Male adj height, offsp 4 & cohort 20 exams	http://www.ncbi.nlm.nih.gov/projects/gap/cgi-bin/analysis.cgi?id=pha000331

Group	Category	Trait Label	Name	FBAT Link
Metabolic	Adiposity	malehgt2205	Male adj height, offsp 5 & cohort 22 exams	http://www.ncbi.nlm.nih.gov/projects/gap/cgi-bin/analysis.cgi?id=pha000333
Metabolic	Adiposity	malehgt2406	Male adj height, offsp 6 & cohort 24 exams	http://www.ncbi.nlm.nih.gov/projects/gap/cgi-bin/analysis.cgi?id=pha000335
Metabolic	Adiposity	malehgt2607	Male adj height, offsp 7 & cohort 26 exams	http://www.ncbi.nlm.nih.gov/projects/gap/cgi-bin/analysis.cgi?id=pha000337
Metabolic	Adiposity	malemeanbmi	Male adj mean bmi, offsp 1-7 & cohort 10,16,18,20,22,24,26 exams	http://www.ncbi.nlm.nih.gov/projects/gap/cgi-bin/analysis.cgi?id=pha000339
Metabolic	Adiposity	malemeanhgt	Male adj mean hgt, offsp 1-7 & cohort 10,16,18,20,22,24,26 exams	http://www.ncbi.nlm.nih.gov/projects/gap/cgi-bin/analysis.cgi?id=pha000341
Metabolic	Adiposity	malemeanwgt	Male adj mean weight, offsp 1-7 & cohort 10,16,18,20,22,24,26 exams	http://www.ncbi.nlm.nih.gov/projects/gap/cgi-bin/analysis.cgi?id=pha000343
Metabolic	Adiposity	maleochgwst	Male adj waist change, offsp 4 to 7	http://www.ncbi.nlm.nih.gov/projects/gap/cgi-bin/analysis.cgi?id=pha000345
Metabolic	Adiposity	maleomeanwst	Male adj mean waist, offsp 4 to 7	http://www.ncbi.nlm.nih.gov/projects/gap/cgi-bin/analysis.cgi?id=pha000347
Metabolic	Adiposity	maleowst0007	Male adj waist, offsp 7	http://www.ncbi.nlm.nih.gov/projects/gap/cgi-bin/analysis.cgi?id=pha000349
Metabolic	Adiposity	malerankbmi1602	Male rank adj bmi, offsp 2 & cohort 16 exams	http://www.ncbi.nlm.nih.gov/projects/gap/cgi-bin/analysis.cgi?id=pha000351
Metabolic	Adiposity	malerankbmi1803	Male rank adj bmi, offsp 3 & cohort 18 exams	http://www.ncbi.nlm.nih.gov/projects/gap/cgi-bin/analysis.cgi?id=pha000353
Metabolic	Adiposity	malerankbmi2004	Male rank adj bmi, offsp 4 & cohort 20 exams	http://www.ncbi.nlm.nih.gov/projects/gap/cgi-bin/analysis.cgi?id=pha000355
Metabolic	Adiposity	malerankbmi2205	Male rank adj bmi, offsp 5 & cohort 22 exams	http://www.ncbi.nlm.nih.gov/projects/gap/cgi-bin/analysis.cgi?id=pha000357
Metabolic	Adiposity	malerankbmi2406	Male rank adj bmi, offsp 6 & cohort 24 exams	http://www.ncbi.nlm.nih.gov/projects/gap/cgi-bin/analysis.cgi?id=pha000359
Metabolic	Adiposity	malerankbmi2607	Male rank adj bmi, offsp 7 & cohort 26 exams	http://www.ncbi.nlm.nih.gov/projects/gap/cgi-bin/analysis.cgi?id=pha000361
Metabolic	Adiposity	malerankchgbmi	Male rank adj bmi change, offsp 1-7 & cohort exams 10-26	http://www.ncbi.nlm.nih.gov/projects/gap/cgi-bin/analysis.cgi?id=pha000363
Metabolic	Adiposity	malerankchgwgt	Male rank adj weight change, offsp 1-7 & cohort exams 10-26	http://www.ncbi.nlm.nih.gov/projects/gap/cgi-bin/analysis.cgi?id=pha000365
Metabolic	Adiposity	malerankhgt2406	Male rank adj height, offsp 6 & cohort 24 exams	http://www.ncbi.nlm.nih.gov/projects/gap/cgi-bin/analysis.cgi?id=pha000367
Metabolic	Adiposity	malerankochgwst	Male rank adj waist change, offsp 4 to 7	http://www.ncbi.nlm.nih.gov/projects/gap/cgi-bin/analysis.cgi?id=pha000369
Metabolic	Adiposity	malerankwgt1602	Male rank adj weight, offsp 2 & cohort 16 exams	http://www.ncbi.nlm.nih.gov/projects/gap/cgi-bin/analysis.cgi?id=pha000371
Metabolic	Adiposity	malerankwgt2004	Male rank adj weight, offsp 4 & cohort 20 exams	http://www.ncbi.nlm.nih.gov/projects/gap/cgi-bin/analysis.cgi?id=pha000373
Metabolic	Adiposity	malerankwgt2205	Male rank adj weight, offsp 5 & cohort 22 exams	http://www.ncbi.nlm.nih.gov/projects/gap/cgi-bin/analysis.cgi?id=pha000375
Metabolic	Adiposity	malerankwst2306	Male rank adj waist, offsp 6 & cohort exam 23	http://www.ncbi.nlm.nih.gov/projects/gap/cgi-bin/analysis.cgi?id=pha000377
Metabolic	Adiposity	malewgt1602	Male adj weight, offsp 2 & cohort 16 exams	http://www.ncbi.nlm.nih.gov/projects/gap/cgi-bin/analysis.cgi?id=pha000379
Metabolic	Adiposity	malewgt1803	Male adj weight, offsp 3 & cohort 18 exams	http://www.ncbi.nlm.nih.gov/projects/gap/cgi-bin/analysis.cgi?id=pha000381
Metabolic	Adiposity	malewgt1x01	Male adj weight, offsp 1 & cohort 10 exams	http://www.ncbi.nlm.nih.gov/projects/gap/cgi-bin/analysis.cgi?id=pha000383
Metabolic	Adiposity	malewgt2004	Male adj weight, offsp 4 & cohort 20 exams	http://www.ncbi.nlm.nih.gov/projects/gap/cgi-bin/analysis.cgi?id=pha000385
Metabolic	Adiposity	malewgt2205	Male adj weight, offsp 5 & cohort 22 exams	http://www.ncbi.nlm.nih.gov/projects/gap/cgi-bin/analysis.cgi?id=pha000387
Metabolic	Adiposity	malewgt2406	Male adj weight, offsp 6 & cohort 24 exams	http://www.ncbi.nlm.nih.gov/projects/gap/cgi-bin/analysis.cgi?id=pha000389
Metabolic	Adiposity	malewgt2607	Male adj weight, offsp 7 & cohort 26 exams	http://www.ncbi.nlm.nih.gov/projects/gap/cgi-bin/analysis.cgi?id=pha000391
Metabolic	Adiposity	malewst2004	Male adj waist, offsp 4 & cohort 20 exams	http://www.ncbi.nlm.nih.gov/projects/gap/cgi-bin/analysis.cgi?id=pha000393
Metabolic	Adiposity	malewst2205	Male adj waist, offsp 5 & cohort 22 exams	http://www.ncbi.nlm.nih.gov/projects/gap/cgi-bin/analysis.cgi?id=pha000395
Metabolic	Adiposity	malewst2306	Male adj waist, offsp 6 & cohort exam 23	http://www.ncbi.nlm.nih.gov/projects/gap/cgi-bin/analysis.cgi?id=pha000397
Metabolic	Adiposity	SATAS	SAT adj for age, age sqr, sex	http://www.ncbi.nlm.nih.gov/projects/gap/cgi-bin/analysis.cgi?id=pha000399
Metabolic	Adiposity	SATMV	SAT adj for age, age square, sex, meno status, smoking	http://www.ncbi.nlm.nih.gov/projects/gap/cgi-bin/analysis.cgi?id=pha000401
Metabolic	Adiposity	SDAS	Sagittal diameter adj for age, age square, sex	http://www.ncbi.nlm.nih.gov/projects/gap/cgi-bin/analysis.cgi?id=pha000403
Metabolic	Adiposity	SDMV	Sagittal diameter adj for age, age square, sex, meno status, smoking	http://www.ncbi.nlm.nih.gov/projects/gap/cgi-bin/analysis.cgi?id=pha000405
Metabolic	Adiposity	VATAS	VAT adj for age, age squared, sex	http://www.ncbi.nlm.nih.gov/projects/gap/cgi-bin/analysis.cgi?id=pha000407
Metabolic	Adiposity	VATMV	VAT adj for age, age squared, sex, meno status, smoking	http://www.ncbi.nlm.nih.gov/projects/gap/cgi-bin/analysis.cgi?id=pha000409
Metabolic	Adiposity	WCAS	Waist by CT adj for age, age squared, sex	http://www.ncbi.nlm.nih.gov/projects/gap/cgi-bin/analysis.cgi?id=pha000411
Metabolic	Adiposity	WCMV	Waist by CT adj for age, age squared, sex, meno status, smoking	http://www.ncbi.nlm.nih.gov/projects/gap/cgi-bin/analysis.cgi?id=pha000413
Metabolic	Glycemic	DiabSurv1sexage	Diabetes incidence to ex7, age-sex adjusted in FBAT, Cox model for GEE	http://www.ncbi.nlm.nih.gov/projects/gap/cgi-bin/analysis.cgi?id=pha000415
Metabolic	Glycemic	DiabSurv2sexagebmi	Diabetes incidence to ex7, age-sex-bmi adjusted in FBAT, Cox model for GEE	http://www.ncbi.nlm.nih.gov/projects/gap/cgi-bin/analysis.cgi?id=pha000417
Metabolic	Glycemic	res1ladipoq7o	Age-sex adjusted Adiponectin ex7 offspring	http://www.ncbi.nlm.nih.gov/projects/gap/cgi-bin/analysis.cgi?id=pha000419
Metabolic	Glycemic	res1lfglu5o	Age-sex adjusted Fasting Plasma Glucose ex5 offspring	http://www.ncbi.nlm.nih.gov/projects/gap/cgi-bin/analysis.cgi?id=pha000421
Metabolic	Glycemic	res1lfglu7o	Age-sex adjusted Fasting Plasma Glucose ex7 offspring	http://www.ncbi.nlm.nih.gov/projects/gap/cgi-bin/analysis.cgi?id=pha000423
Metabolic	Glycemic	res1lfins5o	Age-sex adjusted Fasting Insulin ex5 offspring	http://www.ncbi.nlm.nih.gov/projects/gap/cgi-bin/analysis.cgi?id=pha000425
Metabolic	Glycemic	res1lfins7o	Age-sex adjusted Fasting Insulin ex7 offspring	http://www.ncbi.nlm.nih.gov/projects/gap/cgi-bin/analysis.cgi?id=pha000427
Metabolic	Glycemic	res1lgutt5o	Age-sex adjusted Insulin Sensitivity ex5 offspring	http://www.ncbi.nlm.nih.gov/projects/gap/cgi-bin/analysis.cgi?id=pha000429
Metabolic	Glycemic	res1lhba1c5o	Age-sex adjusted Fasting HbA1c ex5 offspring	http://www.ncbi.nlm.nih.gov/projects/gap/cgi-bin/analysis.cgi?id=pha000431
Metabolic	Glycemic	res1lhba1c7o	Age-sex adjusted HbA1c ex7 offspring	http://www.ncbi.nlm.nih.gov/projects/gap/cgi-bin/analysis.cgi?id=pha000433
Metabolic	Glycemic	res1lhir5o	Age-sex adjusted HOMA-IR ex5 offspring	http://www.ncbi.nlm.nih.gov/projects/gap/cgi-bin/analysis.cgi?id=pha000435
Metabolic	Glycemic	res1lhir7o	Age-sex adjusted HOMA-IR ex7 offspring	http://www.ncbi.nlm.nih.gov/projects/gap/cgi-bin/analysis.cgi?id=pha000437
Metabolic	Glycemic	res1lmfglu17o	Age-sex adjusted Mean Plasma Glucose ex1-7 offspring	http://www.ncbi.nlm.nih.gov/projects/gap/cgi-bin/analysis.cgi?id=pha000439
Metabolic	Glycemic	res1lretn7o	Age-sex adjusted Resistin ex7 offspring	http://www.ncbi.nlm.nih.gov/projects/gap/cgi-bin/analysis.cgi?id=pha000441
Metabolic	Glycemic	res2ladipoq7o	Multivariable adjusted Adiponectin ex7 offspring	http://www.ncbi.nlm.nih.gov/projects/gap/cgi-bin/analysis.cgi?id=pha000443

Group	Category	Trait Label	Name	FBAT Link
Metabolic	Glycemic	res2lfglu5o	Multivariable adjusted Fasting Plasma Glucose ex5 offspring	http://www.ncbi.nlm.nih.gov/projects/gap/cgi-bin/analysis.cgi?id=pha000445
Metabolic	Glycemic	res2lfglu7o	Multivariable adjusted Fasting Plasma Glucose ex7 offspring	http://www.ncbi.nlm.nih.gov/projects/gap/cgi-bin/analysis.cgi?id=pha000447
Metabolic	Glycemic	res2lfins5o	Multivariable adjusted Fasting Insulin ex5 offspring	http://www.ncbi.nlm.nih.gov/projects/gap/cgi-bin/analysis.cgi?id=pha000449
Metabolic	Glycemic	res2lfins7o	Multivariable adjusted Fasting Insulin ex7 offspring	http://www.ncbi.nlm.nih.gov/projects/gap/cgi-bin/analysis.cgi?id=pha000451
Metabolic	Glycemic	res2lgutt5o	Multivariable adjusted Insulin Sensitivity ex5 offspring	http://www.ncbi.nlm.nih.gov/projects/gap/cgi-bin/analysis.cgi?id=pha000453
Metabolic	Glycemic	res2lhba1c5o	Multivariable adjusted Fasting HbA1c ex5 offspring	http://www.ncbi.nlm.nih.gov/projects/gap/cgi-bin/analysis.cgi?id=pha000455
Metabolic	Glycemic	res2lhba1c7o	Multivariable adjusted HbA1c ex7 offspring	http://www.ncbi.nlm.nih.gov/projects/gap/cgi-bin/analysis.cgi?id=pha000457
Metabolic	Glycemic	res2lhir5o	Multivariable adjusted HOMA-IR ex5 offspring	http://www.ncbi.nlm.nih.gov/projects/gap/cgi-bin/analysis.cgi?id=pha000459
Metabolic	Glycemic	res2lhir7o	Multivariable adjusted HOMA-IR ex7 offspring	http://www.ncbi.nlm.nih.gov/projects/gap/cgi-bin/analysis.cgi?id=pha000461
Metabolic	Glycemic	res2lmfglu17o	Multivariable adjusted Mean Plasma Glucose exam1-7 offspring	http://www.ncbi.nlm.nih.gov/projects/gap/cgi-bin/analysis.cgi?id=pha000463
Metabolic	Glycemic	res2lretn7o	Multivariable adjusted Resistin ex7 offspring	http://www.ncbi.nlm.nih.gov/projects/gap/cgi-bin/analysis.cgi?id=pha000465
Metabolic	Lipids	ApoA14a	Multivariable adj Plasma ApoA1 Level Offsp ex4	http://www.ncbi.nlm.nih.gov/projects/gap/cgi-bin/analysis.cgi?id=pha000467
Metabolic	Lipids	ApoA14b	Age-sex adj Plasma ApoA1 Level Offsp ex4	http://www.ncbi.nlm.nih.gov/projects/gap/cgi-bin/analysis.cgi?id=pha000469
Metabolic	Lipids	ApoB4a	Multivariable adj Plasma ApoB Level Offsp ex4	http://www.ncbi.nlm.nih.gov/projects/gap/cgi-bin/analysis.cgi?id=pha000471
Metabolic	Lipids	ApoB4b	Age-sex adj Plasma ApoB Level Offsp ex4	http://www.ncbi.nlm.nih.gov/projects/gap/cgi-bin/analysis.cgi?id=pha000473
Metabolic	Lipids	ApoC3a	Multivariable adj Plasma ApoCIII Level Offsp ex5	http://www.ncbi.nlm.nih.gov/projects/gap/cgi-bin/analysis.cgi?id=pha000475
Metabolic	Lipids	ApoC3b	Age-sex adj Plasma ApoCIII Level Offsp ex5	http://www.ncbi.nlm.nih.gov/projects/gap/cgi-bin/analysis.cgi?id=pha000477
Metabolic	Lipids	chol1a	Multivariable adj Chol Offsp ex1	http://www.ncbi.nlm.nih.gov/projects/gap/cgi-bin/analysis.cgi?id=pha000479
Metabolic	Lipids	chol1b	Age-sex adj Chol Offsp ex1	http://www.ncbi.nlm.nih.gov/projects/gap/cgi-bin/analysis.cgi?id=pha000481
Metabolic	Lipids	chol2b	Age-sex adj Chol Offsp ex2	http://www.ncbi.nlm.nih.gov/projects/gap/cgi-bin/analysis.cgi?id=pha000483
Metabolic	Lipids	chol3b	Age-sex adj Chol Offsp ex3	http://www.ncbi.nlm.nih.gov/projects/gap/cgi-bin/analysis.cgi?id=pha000485
Metabolic	Lipids	chol4b	Age-sex adj Chol Offsp ex4	http://www.ncbi.nlm.nih.gov/projects/gap/cgi-bin/analysis.cgi?id=pha000487
Metabolic	Lipids	chol5b	Age-sex adj Chol Offsp ex5	http://www.ncbi.nlm.nih.gov/projects/gap/cgi-bin/analysis.cgi?id=pha000489
Metabolic	Lipids	chol6b	Age-sex adj Chol Offsp ex6	http://www.ncbi.nlm.nih.gov/projects/gap/cgi-bin/analysis.cgi?id=pha000491
Metabolic	Lipids	chol7b	Age-sex adj Chol Offsp ex7	http://www.ncbi.nlm.nih.gov/projects/gap/cgi-bin/analysis.cgi?id=pha000493
Metabolic	Lipids	cholhdl1a	Multvariable adj Chol-HDL Ratio Offsp ex1	http://www.ncbi.nlm.nih.gov/projects/gap/cgi-bin/analysis.cgi?id=pha000495
Metabolic	Lipids	cholhdl1b	Age-sex adj Chol-HDL Ratio Offsp ex1	http://www.ncbi.nlm.nih.gov/projects/gap/cgi-bin/analysis.cgi?id=pha000497
Metabolic	Lipids	cholhdl2b	Age-sex adj Chol-HDL Ratio Offsp ex2	http://www.ncbi.nlm.nih.gov/projects/gap/cgi-bin/analysis.cgi?id=pha000499
Metabolic	Lipids	cholhdl3b	Age-sex adj Chol-HDL Ratio Offsp ex3	http://www.ncbi.nlm.nih.gov/projects/gap/cgi-bin/analysis.cgi?id=pha000501
Metabolic	Lipids	cholhdl4b	Age-sex adj Chol-HDL Ratio Offsp ex4	http://www.ncbi.nlm.nih.gov/projects/gap/cgi-bin/analysis.cgi?id=pha000503
Metabolic	Lipids	cholhdl5b	Age-sex adj Chol-HDL Ratio Offsp ex5	http://www.ncbi.nlm.nih.gov/projects/gap/cgi-bin/analysis.cgi?id=pha000505
Metabolic	Lipids	cholhdl6b	Age-sex adj Chol-HDL Ratio Offsp ex6	http://www.ncbi.nlm.nih.gov/projects/gap/cgi-bin/analysis.cgi?id=pha000507
Metabolic	Lipids	cholhdl7b	Age-sex adj Chol-HDL Ratio Offsp ex7	http://www.ncbi.nlm.nih.gov/projects/gap/cgi-bin/analysis.cgi?id=pha000509
Metabolic	Lipids	hdl1a	Multivariable adj HDL Offsp ex1	http://www.ncbi.nlm.nih.gov/projects/gap/cgi-bin/analysis.cgi?id=pha000511
Metabolic	Lipids	hdl1b	Age-sex adj HDL Offsp ex1	http://www.ncbi.nlm.nih.gov/projects/gap/cgi-bin/analysis.cgi?id=pha000513
Metabolic	Lipids	hdl24a	Multivariable adj HDL2 Offsp ex4	http://www.ncbi.nlm.nih.gov/projects/gap/cgi-bin/analysis.cgi?id=pha000515
Metabolic	Lipids	hdl24b	Age-sex adj HDL2 Offsp ex4	http://www.ncbi.nlm.nih.gov/projects/gap/cgi-bin/analysis.cgi?id=pha000517
Metabolic	Lipids	hdl25a	Multivariable adj HDL2 Offsp ex5	http://www.ncbi.nlm.nih.gov/projects/gap/cgi-bin/analysis.cgi?id=pha000519
Metabolic	Lipids	hdl25b	Age-sex adj HDL2 Offsp ex5	http://www.ncbi.nlm.nih.gov/projects/gap/cgi-bin/analysis.cgi?id=pha000521
Metabolic	Lipids	hdl2b	Age-sex adj HDL Offsp ex2	http://www.ncbi.nlm.nih.gov/projects/gap/cgi-bin/analysis.cgi?id=pha000523
Metabolic	Lipids	hdl34a	Multivariable adj HDL3 Offsp ex4	http://www.ncbi.nlm.nih.gov/projects/gap/cgi-bin/analysis.cgi?id=pha000525
Metabolic	Lipids	hdl34b	Age-sex adj HDL3 Offsp ex4	http://www.ncbi.nlm.nih.gov/projects/gap/cgi-bin/analysis.cgi?id=pha000527
Metabolic	Lipids	hdl35a	Multivariable adj HDL3 Offsp ex5	http://www.ncbi.nlm.nih.gov/projects/gap/cgi-bin/analysis.cgi?id=pha000529
Metabolic	Lipids	hdl35b	Age-sex adj HDL3 Offsp ex5	http://www.ncbi.nlm.nih.gov/projects/gap/cgi-bin/analysis.cgi?id=pha000531
Metabolic	Lipids	hdl3b	Age-sex adj HDL Offsp ex3	http://www.ncbi.nlm.nih.gov/projects/gap/cgi-bin/analysis.cgi?id=pha000533
Metabolic	Lipids	hdl4b	Age-sex adj HDL Offsp ex4	http://www.ncbi.nlm.nih.gov/projects/gap/cgi-bin/analysis.cgi?id=pha000535
Metabolic	Lipids	hdl5b	Age-sex adj HDL Offsp ex5	http://www.ncbi.nlm.nih.gov/projects/gap/cgi-bin/analysis.cgi?id=pha000537
Metabolic	Lipids	hdl6b	Age-sex adj HDL Offsp ex6	http://www.ncbi.nlm.nih.gov/projects/gap/cgi-bin/analysis.cgi?id=pha000539
Metabolic	Lipids	hdl7b	Age-sex adj HDL Offsp ex7	http://www.ncbi.nlm.nih.gov/projects/gap/cgi-bin/analysis.cgi?id=pha000541
Metabolic	Lipids	HDLNMRint4a	Multivariable adj NMR HDL Int Offsp ex4 or TG > 400	http://www.ncbi.nlm.nih.gov/projects/gap/cgi-bin/analysis.cgi?id=pha000543
Metabolic	Lipids	HDLNMRint4b	Age-sex adj NMR HDL Int Offsp ex4 or TG > 400	http://www.ncbi.nlm.nih.gov/projects/gap/cgi-bin/analysis.cgi?id=pha000545
Metabolic	Lipids	HDLNMRlg4a	Multivariable adj NMR HDL Lg Offsp ex4 or TG > 400	http://www.ncbi.nlm.nih.gov/projects/gap/cgi-bin/analysis.cgi?id=pha000547
Metabolic	Lipids	HDLNMRlg4b	Age-sex adj NMR HDL Lg Offsp ex4 or TG > 400	http://www.ncbi.nlm.nih.gov/projects/gap/cgi-bin/analysis.cgi?id=pha000549
Metabolic	Lipids	HDLNMRsm4a	Multivariable adj NMR HDL Sm Offsp ex4 or TG > 400	http://www.ncbi.nlm.nih.gov/projects/gap/cgi-bin/analysis.cgi?id=pha000551
Metabolic	Lipids	HDLNMRsm4b	Age-sex adj NMR HDL Sm Offsp ex4 or TG > 400	http://www.ncbi.nlm.nih.gov/projects/gap/cgi-bin/analysis.cgi?id=pha000553
Metabolic	Lipids	HDLNMRsz4a	Multivariable adj NMR HDL Size Offsp ex4 or TG > 400	http://www.ncbi.nlm.nih.gov/projects/gap/cgi-bin/analysis.cgi?id=pha000555

Group	Category	Trait Label	Name	FBAT Link
Metabolic	Lipids	HDLNMRsz4b	Age-sex adj NMR HDL Size Offsp ex4 or TG > 400	http://www.ncbi.nlm.nih.gov/projects/gap/cgi-bin/analysis.cgi?id=pha000557
Metabolic	Lipids	IDLNMR4a	Multivariable adj NMR IDL Offsp ex4 or TG > 400	http://www.ncbi.nlm.nih.gov/projects/gap/cgi-bin/analysis.cgi?id=pha000559
Metabolic	Lipids	IDLNMR4b	Age-sex adj NMR IDL Offsp ex4 or TG > 400	http://www.ncbi.nlm.nih.gov/projects/gap/cgi-bin/analysis.cgi?id=pha000561
Metabolic	Lipids	ldl1a	Multivariable adj LDL Offsp ex1	http://www.ncbi.nlm.nih.gov/projects/gap/cgi-bin/analysis.cgi?id=pha000563
Metabolic	Lipids	ldl1b	Age-sex adj LDL Offsp ex1	http://www.ncbi.nlm.nih.gov/projects/gap/cgi-bin/analysis.cgi?id=pha000565
Metabolic	Lipids	ldl2b	Age-sex adj LDL Offsp ex2	http://www.ncbi.nlm.nih.gov/projects/gap/cgi-bin/analysis.cgi?id=pha000567
Metabolic	Lipids	ldl3b	Age-sex adj LDL Offsp ex3	http://www.ncbi.nlm.nih.gov/projects/gap/cgi-bin/analysis.cgi?id=pha000569
Metabolic	Lipids	ldl4b	Age-sex adj LDL Offsp ex4	http://www.ncbi.nlm.nih.gov/projects/gap/cgi-bin/analysis.cgi?id=pha000571
Metabolic	Lipids	ldl5b	Age-sex adj LDL Offsp ex5	http://www.ncbi.nlm.nih.gov/projects/gap/cgi-bin/analysis.cgi?id=pha000573
Metabolic	Lipids	ldl6b	Age-sex adj LDL Offsp ex6	http://www.ncbi.nlm.nih.gov/projects/gap/cgi-bin/analysis.cgi?id=pha000575
Metabolic	Lipids	ldl7b	Age-sex adj LDL Offsp ex7	http://www.ncbi.nlm.nih.gov/projects/gap/cgi-bin/analysis.cgi?id=pha000577
Metabolic	Lipids	LDLNMRlg4a	Multivariable adj NMR LDL Lg Offsp ex4 or TG > 400	http://www.ncbi.nlm.nih.gov/projects/gap/cgi-bin/analysis.cgi?id=pha000579
Metabolic	Lipids	LDLNMRlg4b	Age-sex adj NMR LDL Lg Offsp ex4 or TG > 400	http://www.ncbi.nlm.nih.gov/projects/gap/cgi-bin/analysis.cgi?id=pha000581
Metabolic	Lipids	LDLNMRsm4a	Multivariable adj NMR LDL Sm Offsp ex4 or TG > 400	http://www.ncbi.nlm.nih.gov/projects/gap/cgi-bin/analysis.cgi?id=pha000583
Metabolic	Lipids	LDLNMRsm4b	Age-sex adj NMR LDL Sm Offsp ex4 or TG > 400	http://www.ncbi.nlm.nih.gov/projects/gap/cgi-bin/analysis.cgi?id=pha000585
Metabolic	Lipids	LDLNMRsz4a	Multivariable adj NMR LDL Size Offsp ex4 or TG > 400	http://www.ncbi.nlm.nih.gov/projects/gap/cgi-bin/analysis.cgi?id=pha000587
Metabolic	Lipids	LDLNMRsz4b	Age-sex adj NMR LDL Size Offsp ex4 or TG > 400	http://www.ncbi.nlm.nih.gov/projects/gap/cgi-bin/analysis.cgi?id=pha000589
Metabolic	Lipids	lpa3a	Multivariable adj Lpa Offsp ex3	http://www.ncbi.nlm.nih.gov/projects/gap/cgi-bin/analysis.cgi?id=pha000591
Metabolic	Lipids	lpa3b	Age-sex adj Lpa Offsp ex3	http://www.ncbi.nlm.nih.gov/projects/gap/cgi-bin/analysis.cgi?id=pha000593
Metabolic	Lipids	meanchola	Multivariable adj NMR Chol Offsp ex1-7	http://www.ncbi.nlm.nih.gov/projects/gap/cgi-bin/analysis.cgi?id=pha000595
Metabolic	Lipids	meancholb	Age-sex adj NMR Chol Offsp ex1-7	http://www.ncbi.nlm.nih.gov/projects/gap/cgi-bin/analysis.cgi?id=pha000597
Metabolic	Lipids	meanhdla	Multivariable adj NMR HDL Offsp ex1-7	http://www.ncbi.nlm.nih.gov/projects/gap/cgi-bin/analysis.cgi?id=pha000599
Metabolic	Lipids	meanhdlb	Age-sex adj NMR HDL Offsp ex1-7	http://www.ncbi.nlm.nih.gov/projects/gap/cgi-bin/analysis.cgi?id=pha000601
Metabolic	Lipids	meanldla	Multivariable adj NMR Calc LDL Offsp ex1-7	http://www.ncbi.nlm.nih.gov/projects/gap/cgi-bin/analysis.cgi?id=pha000603
Metabolic	Lipids	meanldlb	Age-sex adj NMR Calc LDL Offsp ex1-7	http://www.ncbi.nlm.nih.gov/projects/gap/cgi-bin/analysis.cgi?id=pha000605
Metabolic	Lipids	meantga	Multivariable adj NMR (TG) Offsp ex1-7	http://www.ncbi.nlm.nih.gov/projects/gap/cgi-bin/analysis.cgi?id=pha000607
Metabolic	Lipids	meantgb	Age-sex adj NMR (TG) Offsp ex1-7	http://www.ncbi.nlm.nih.gov/projects/gap/cgi-bin/analysis.cgi?id=pha000609
Metabolic	Lipids	PlasmaApoEa	Multivariable adj Plasma ApoE Level Offsp ex5	http://www.ncbi.nlm.nih.gov/projects/gap/cgi-bin/analysis.cgi?id=pha000611
Metabolic	Lipids	PlasmaApoEb	Age-sex adj Plasma ApoE Level Offsp ex5	http://www.ncbi.nlm.nih.gov/projects/gap/cgi-bin/analysis.cgi?id=pha000613
Metabolic	Lipids	RLPChol4a	Multivariable adj Remnant LP Chol Offsp ex4	http://www.ncbi.nlm.nih.gov/projects/gap/cgi-bin/analysis.cgi?id=pha000923
Metabolic	Lipids	RLPChol4b	Age-sex adj Remnant LP Chol Offsp ex4	http://www.ncbi.nlm.nih.gov/projects/gap/cgi-bin/analysis.cgi?id=pha000925
Metabolic	Lipids	RLPTG4a	Remnant LP TG Offsp ex4	http://www.ncbi.nlm.nih.gov/projects/gap/cgi-bin/analysis.cgi?id=pha000617
Metabolic	Lipids	RLPTG4b	Remnant LP Age-sex adj TG Offsp ex4	http://www.ncbi.nlm.nih.gov/projects/gap/cgi-bin/analysis.cgi?id=pha000615
Metabolic	Lipids	tg1a	Multivariable adj TG Offsp ex1	http://www.ncbi.nlm.nih.gov/projects/gap/cgi-bin/analysis.cgi?id=pha000619
Metabolic	Lipids	tg1b	Age-sex adj TG Offsp ex1	http://www.ncbi.nlm.nih.gov/projects/gap/cgi-bin/analysis.cgi?id=pha000621
Metabolic	Lipids	tg2b	Age-sex adj TG Offsp ex2	http://www.ncbi.nlm.nih.gov/projects/gap/cgi-bin/analysis.cgi?id=pha000623
Metabolic	Lipids	tg3b	Age-sex adj TG Offsp ex3	http://www.ncbi.nlm.nih.gov/projects/gap/cgi-bin/analysis.cgi?id=pha000625
Metabolic	Lipids	tg4b	Age-sex adj TG Offsp ex4	http://www.ncbi.nlm.nih.gov/projects/gap/cgi-bin/analysis.cgi?id=pha000627
Metabolic	Lipids	tg5b	Age-sex adj TG Offsp ex5	http://www.ncbi.nlm.nih.gov/projects/gap/cgi-bin/analysis.cgi?id=pha000629
Metabolic	Lipids	tg6b	Age-sex adj TG Offsp ex6	http://www.ncbi.nlm.nih.gov/projects/gap/cgi-bin/analysis.cgi?id=pha000631
Metabolic	Lipids	tg7b	Age-sex adj TG Offsp ex7	http://www.ncbi.nlm.nih.gov/projects/gap/cgi-bin/analysis.cgi?id=pha000633
Metabolic	Lipids	tghdl1a	Multivariable adj TG-HDL Ratio Offsp ex1	http://www.ncbi.nlm.nih.gov/projects/gap/cgi-bin/analysis.cgi?id=pha000667
Metabolic	Lipids	tghdl1b	Age-sex adj TG-HDL Ratio Offsp ex1	http://www.ncbi.nlm.nih.gov/projects/gap/cgi-bin/analysis.cgi?id=pha000913
Metabolic	Lipids	tghdl2b	Age-sex adj TG-HDL Ratio Offsp ex2	http://www.ncbi.nlm.nih.gov/projects/gap/cgi-bin/analysis.cgi?id=pha000669
Metabolic	Lipids	tghdl3b	Age-sex adj TG-HDL Ratio Offsp ex3	http://www.ncbi.nlm.nih.gov/projects/gap/cgi-bin/analysis.cgi?id=pha000671
Metabolic	Lipids	tghdl4b	Age-sex adj TG-HDL Ratio Offsp ex4	http://www.ncbi.nlm.nih.gov/projects/gap/cgi-bin/analysis.cgi?id=pha000673
Metabolic	Lipids	tghdl5b	Age-sex adj TG-HDL Ratio Offsp ex5	http://www.ncbi.nlm.nih.gov/projects/gap/cgi-bin/analysis.cgi?id=pha000675
Metabolic	Lipids	tghdl6b	Age-sex adj TG-HDL Ratio Offsp ex6	http://www.ncbi.nlm.nih.gov/projects/gap/cgi-bin/analysis.cgi?id=pha000677
Metabolic	Lipids	tghdl7b	Age-sex adj TG-HDL Ratio Offsp ex7	http://www.ncbi.nlm.nih.gov/projects/gap/cgi-bin/analysis.cgi?id=pha000679
Metabolic	Lipids	VLDLNMRint4a	Multivariable adj NMR VLDL Int Offsp ex4	http://www.ncbi.nlm.nih.gov/projects/gap/cgi-bin/analysis.cgi?id=pha000865
Metabolic	Lipids	VLDLNMRint4b	Age-sex adj NMR VLDL Int Offsp ex4	http://www.ncbi.nlm.nih.gov/projects/gap/cgi-bin/analysis.cgi?id=pha000867
Metabolic	Lipids	VLDLNMRlg4a	Multivariable adj NMR VLDL Lg Offsp ex4	http://www.ncbi.nlm.nih.gov/projects/gap/cgi-bin/analysis.cgi?id=pha000877
Metabolic	Lipids	VLDLNMRlg4b	Age-sex adj NMR VLDL Lg Offsp ex4	http://www.ncbi.nlm.nih.gov/projects/gap/cgi-bin/analysis.cgi?id=pha000879
Metabolic	Lipids	VLDLNMRsm4a	Multivariable adj NMR VLDL Sm Offsp ex4	http://www.ncbi.nlm.nih.gov/projects/gap/cgi-bin/analysis.cgi?id=pha000901
Metabolic	Lipids	VLDLNMRsm4b	Age-sex adj NMR VLDL Sm Offsp ex4	http://www.ncbi.nlm.nih.gov/projects/gap/cgi-bin/analysis.cgi?id=pha000903
Metabolic	Lipids	VLDLNMRsz4a	Multivariable adj NMR VLDL Size Offsp ex4	http://www.ncbi.nlm.nih.gov/projects/gap/cgi-bin/analysis.cgi?id=pha000889

Group	Category	Trait Label	Name	FBAT Link
Metabolic	Lipids	VLDLNMRsz4b	Age-sex adj NMR VLDL Size Offsp ex4	http://www.ncbi.nlm.nih.gov/projects/gap/cgi-bin/analysis.cgi?id=pha000891
Metabolic	Lipidsmen	menApoB4a	Multivariable adj Plasma ApoB Level Offsp men ex4	http://www.ncbi.nlm.nih.gov/projects/gap/cgi-bin/analysis.cgi?id=pha000915
Metabolic	Lipidsmen	menApoB4b	Age-sex adj Plasma ApoB Level Offsp men ex4	http://www.ncbi.nlm.nih.gov/projects/gap/cgi-bin/analysis.cgi?id=pha000917
Metabolic	Lipidsmen	menchol1a	Multivariable adj Chol Offsp men ex1	http://www.ncbi.nlm.nih.gov/projects/gap/cgi-bin/analysis.cgi?id=pha000709
Metabolic	Lipidsmen	menchol1b	Age-sex adj Chol Offsp men ex1	http://www.ncbi.nlm.nih.gov/projects/gap/cgi-bin/analysis.cgi?id=pha000711
Metabolic	Lipidsmen	menchol2b	Age-sex adj Chol Offsp men ex2	http://www.ncbi.nlm.nih.gov/projects/gap/cgi-bin/analysis.cgi?id=pha000713
Metabolic	Lipidsmen	menchol3b	Age-sex adj Chol Offsp men ex3	http://www.ncbi.nlm.nih.gov/projects/gap/cgi-bin/analysis.cgi?id=pha000715
Metabolic	Lipidsmen	menchol4b	Age-sex adj Chol Offsp men ex4	http://www.ncbi.nlm.nih.gov/projects/gap/cgi-bin/analysis.cgi?id=pha000717
Metabolic	Lipidsmen	menchol5b	Age-sex adj Chol Offsp men ex5	http://www.ncbi.nlm.nih.gov/projects/gap/cgi-bin/analysis.cgi?id=pha000719
Metabolic	Lipidsmen	menchol6b	Age-sex adj Chol Offsp men ex6	http://www.ncbi.nlm.nih.gov/projects/gap/cgi-bin/analysis.cgi?id=pha000721
Metabolic	Lipidsmen	menchol7b	Age-sex adj Chol Offsp men ex7	http://www.ncbi.nlm.nih.gov/projects/gap/cgi-bin/analysis.cgi?id=pha000723
Metabolic	Lipidsmen	menhdl1a	Multivariable adj HDL Offsp men ex1	http://www.ncbi.nlm.nih.gov/projects/gap/cgi-bin/analysis.cgi?id=pha000741
Metabolic	Lipidsmen	menhdl1b	Age-sex adj HDL Offsp men ex1	http://www.ncbi.nlm.nih.gov/projects/gap/cgi-bin/analysis.cgi?id=pha000743
Metabolic	Lipidsmen	menhdl2b	Age-sex adj HDL Offsp men ex2	http://www.ncbi.nlm.nih.gov/projects/gap/cgi-bin/analysis.cgi?id=pha000745
Metabolic	Lipidsmen	menhdl3b	Age-sex adj HDL Offsp men ex3	http://www.ncbi.nlm.nih.gov/projects/gap/cgi-bin/analysis.cgi?id=pha000747
Metabolic	Lipidsmen	menhdl4b	Age-sex adj HDL Offsp men ex4	http://www.ncbi.nlm.nih.gov/projects/gap/cgi-bin/analysis.cgi?id=pha000749
Metabolic	Lipidsmen	menhdl5b	Age-sex adj HDL Offsp men ex5	http://www.ncbi.nlm.nih.gov/projects/gap/cgi-bin/analysis.cgi?id=pha000751
Metabolic	Lipidsmen	menhdl6b	Age-sex adj HDL Offsp men ex6	http://www.ncbi.nlm.nih.gov/projects/gap/cgi-bin/analysis.cgi?id=pha000753
Metabolic	Lipidsmen	menhdl7b	Age-sex adj HDL Offsp men ex7	http://www.ncbi.nlm.nih.gov/projects/gap/cgi-bin/analysis.cgi?id=pha000755
Metabolic	Lipidsmen	menHDLNMRint4a	Multivariable adj NMR HDL Int Offsp men ex4	http://www.ncbi.nlm.nih.gov/projects/gap/cgi-bin/analysis.cgi?id=pha000809
Metabolic	Lipidsmen	menHDLNMRint4b	Age-sex adj NMR HDL Int Offsp men ex4	http://www.ncbi.nlm.nih.gov/projects/gap/cgi-bin/analysis.cgi?id=pha000811
Metabolic	Lipidsmen	menHDLNMRlg4a	Multivariable adj NMR HDL Lg Offsp men ex4	http://www.ncbi.nlm.nih.gov/projects/gap/cgi-bin/analysis.cgi?id=pha000817
Metabolic	Lipidsmen	menHDLNMRlg4b	Age-sex adj NMR HDL Lg Offsp men ex4	http://www.ncbi.nlm.nih.gov/projects/gap/cgi-bin/analysis.cgi?id=pha000819
Metabolic	Lipidsmen	menHDLNMRsm4a	Multivariable adj NMR HDL Sm Offsp men ex4	http://www.ncbi.nlm.nih.gov/projects/gap/cgi-bin/analysis.cgi?id=pha000833
Metabolic	Lipidsmen	menHDLNMRsm4b	Age-sex adj NMR HDL Sm Offsp men ex4	http://www.ncbi.nlm.nih.gov/projects/gap/cgi-bin/analysis.cgi?id=pha000835
Metabolic	Lipidsmen	menHDLNMRsz4a	Multivariable adj NMR HDL Size Offsp men ex4	http://www.ncbi.nlm.nih.gov/projects/gap/cgi-bin/analysis.cgi?id=pha000825
Metabolic	Lipidsmen	menHDLNMRsz4b	Age-sex adj NMR HDL Size Offsp men ex4	http://www.ncbi.nlm.nih.gov/projects/gap/cgi-bin/analysis.cgi?id=pha000827
Metabolic	Lipidsmen	menldl1a	Multivariable adj LDL Offsp men ex1	http://www.ncbi.nlm.nih.gov/projects/gap/cgi-bin/analysis.cgi?id=pha000681
Metabolic	Lipidsmen	menldl1b	Age-sex adj LDL Offsp men ex1	http://www.ncbi.nlm.nih.gov/projects/gap/cgi-bin/analysis.cgi?id=pha000773
Metabolic	Lipidsmen	menldl2b	Age-sex adj LDL Offsp men ex2	http://www.ncbi.nlm.nih.gov/projects/gap/cgi-bin/analysis.cgi?id=pha000683
Metabolic	Lipidsmen	menldl3b	Age-sex adj LDL Offsp men ex3	http://www.ncbi.nlm.nih.gov/projects/gap/cgi-bin/analysis.cgi?id=pha000685
Metabolic	Lipidsmen	menldl4b	Age-sex adj LDL Offsp men ex4	http://www.ncbi.nlm.nih.gov/projects/gap/cgi-bin/analysis.cgi?id=pha000687
Metabolic	Lipidsmen	menldl5b	Age-sex adj LDL Offsp men ex5	http://www.ncbi.nlm.nih.gov/projects/gap/cgi-bin/analysis.cgi?id=pha000689
Metabolic	Lipidsmen	menldl6b	Age-sex adj LDL Offsp men ex6	http://www.ncbi.nlm.nih.gov/projects/gap/cgi-bin/analysis.cgi?id=pha000691
Metabolic	Lipidsmen	menldl7b	Age-sex adj LDL Offsp men ex7	http://www.ncbi.nlm.nih.gov/projects/gap/cgi-bin/analysis.cgi?id=pha000693
Metabolic	Lipidsmen	menLDLNMRlg4a	Multivariable adj NMR LDL Lg Offsp men ex4	http://www.ncbi.nlm.nih.gov/projects/gap/cgi-bin/analysis.cgi?id=pha000841
Metabolic	Lipidsmen	menLDLNMRlg4b	Age-sex adj NMR LDL Lg Offsp men ex4	http://www.ncbi.nlm.nih.gov/projects/gap/cgi-bin/analysis.cgi?id=pha000843
Metabolic	Lipidsmen	menLDLNMRsm4a	Multivariable adj NMR LDL Sm Offsp men ex4	http://www.ncbi.nlm.nih.gov/projects/gap/cgi-bin/analysis.cgi?id=pha000857
Metabolic	Lipidsmen	menLDLNMRsm4b	Age-sex adj NMR LDL Sm Offsp men ex4	http://www.ncbi.nlm.nih.gov/projects/gap/cgi-bin/analysis.cgi?id=pha000859
Metabolic	Lipidsmen	menLDLNMRsz4a	Multivariable adj NMR LDL Size Offsp men ex4	http://www.ncbi.nlm.nih.gov/projects/gap/cgi-bin/analysis.cgi?id=pha000849
Metabolic	Lipidsmen	menLDLNMRsz4b	Age-sex adj NMR LDL Size Offsp men ex4	http://www.ncbi.nlm.nih.gov/projects/gap/cgi-bin/analysis.cgi?id=pha000851
Metabolic	Lipidsmen	menmeanchola	Multivariable adj Mean Chol Offsp men ex1-7	http://www.ncbi.nlm.nih.gov/projects/gap/cgi-bin/analysis.cgi?id=pha000793
Metabolic	Lipidsmen	menmeancholb	Age-sex adj Mean Chol Offsp men ex1-7	http://www.ncbi.nlm.nih.gov/projects/gap/cgi-bin/analysis.cgi?id=pha000795
Metabolic	Lipidsmen	menmeanhdla	Multivariable adj Mean HDL Offsp men ex1-7	http://www.ncbi.nlm.nih.gov/projects/gap/cgi-bin/analysis.cgi?id=pha000801
Metabolic	Lipidsmen	menmeanhdlb	Age-sex adj Mean HDL Offsp men ex1-7	http://www.ncbi.nlm.nih.gov/projects/gap/cgi-bin/analysis.cgi?id=pha000803
Metabolic	Lipidsmen	menmeanldla	Multivariable adj Mean LDL Offsp men ex1-7	http://www.ncbi.nlm.nih.gov/projects/gap/cgi-bin/analysis.cgi?id=pha000785
Metabolic	Lipidsmen	menmeanldlb	Age-sex adj Mean LDL Offsp men ex1-7	http://www.ncbi.nlm.nih.gov/projects/gap/cgi-bin/analysis.cgi?id=pha000787
Metabolic	Lipidsmen	menmeantga	Multivariable adj Mean TG Offsp men ex1-7	http://www.ncbi.nlm.nih.gov/projects/gap/cgi-bin/analysis.cgi?id=pha000777
Metabolic	Lipidsmen	menmeantgb	Age-sex adj Mean TG Offsp men ex1-7	http://www.ncbi.nlm.nih.gov/projects/gap/cgi-bin/analysis.cgi?id=pha000779
Metabolic	Lipidsmen	mentg1a	Multivariable adj TG Offsp men ex1	http://www.ncbi.nlm.nih.gov/projects/gap/cgi-bin/analysis.cgi?id=pha000635
Metabolic	Lipidsmen	mentg1b	Age-sex adj TG Offsp men ex1	http://www.ncbi.nlm.nih.gov/projects/gap/cgi-bin/analysis.cgi?id=pha000637
Metabolic	Lipidsmen	mentg2b	Age-sex adj TG Offsp men ex2	http://www.ncbi.nlm.nih.gov/projects/gap/cgi-bin/analysis.cgi?id=pha000639
Metabolic	Lipidsmen	mentg3b	Age-sex adj TG Offsp men ex3	http://www.ncbi.nlm.nih.gov/projects/gap/cgi-bin/analysis.cgi?id=pha000641
Metabolic	Lipidsmen	mentg4b	Age-sex adj TG Offsp men ex4	http://www.ncbi.nlm.nih.gov/projects/gap/cgi-bin/analysis.cgi?id=pha000643
Metabolic	Lipidsmen	mentg5b	Age-sex adj TG Offsp men ex5	http://www.ncbi.nlm.nih.gov/projects/gap/cgi-bin/analysis.cgi?id=pha000645
Metabolic	Lipidsmen	mentg6b	Age-sex adj TG Offsp men ex6	http://www.ncbi.nlm.nih.gov/projects/gap/cgi-bin/analysis.cgi?id=pha000647

Group	Category	Trait Label	Name	FBAT Link
Metabolic	Lipidsmen	mentg7b	Age-sex adj TG Offsp men ex7	http://www.ncbi.nlm.nih.gov/projects/gap/cgi-bin/analysis.cgi?id=pha000649
Metabolic	Lipidsmen	menVLDLNMRint4a	Multivariable adj NMR VLDL Int Offsp men ex4	http://www.ncbi.nlm.nih.gov/projects/gap/cgi-bin/analysis.cgi?id=pha000869
Metabolic	Lipidsmen	menVLDLNMRint4b	Age-sex adj NMR VLDL Int Offsp men ex4	http://www.ncbi.nlm.nih.gov/projects/gap/cgi-bin/analysis.cgi?id=pha000871
Metabolic	Lipidsmen	menVLDLNMRlg4a	Multivariable adj NMR VLDL Lg Offsp men ex4	http://www.ncbi.nlm.nih.gov/projects/gap/cgi-bin/analysis.cgi?id=pha000881
Metabolic	Lipidsmen	menVLDLNMRlg4b	Age-sex adj NMR VLDL Lg Offsp men ex4	http://www.ncbi.nlm.nih.gov/projects/gap/cgi-bin/analysis.cgi?id=pha000883
Metabolic	Lipidsmen	menVLDLNMRsm4a	Multivariable adj NMR VLDL Sm Offsp men ex4	http://www.ncbi.nlm.nih.gov/projects/gap/cgi-bin/analysis.cgi?id=pha000905
Metabolic	Lipidsmen	menVLDLNMRsm4b	Age-sex adj NMR VLDL Sm Offsp men ex4	http://www.ncbi.nlm.nih.gov/projects/gap/cgi-bin/analysis.cgi?id=pha000907
Metabolic	Lipidsmen	menVLDLNMRsz4a	Multivariable adj NMR VLDL Size Offsp men ex4	http://www.ncbi.nlm.nih.gov/projects/gap/cgi-bin/analysis.cgi?id=pha000893
Metabolic	Lipidsmen	menVLDLNMRsz4b	Age-sex adj NMR VLDL Size Offsp men ex4	http://www.ncbi.nlm.nih.gov/projects/gap/cgi-bin/analysis.cgi?id=pha000895
Metabolic	Lipidswomen	womenApoB4a	Multivariable adj Plasma ApoB Level Offsp wom ex4	http://www.ncbi.nlm.nih.gov/projects/gap/cgi-bin/analysis.cgi?id=pha000919
Metabolic	Lipidswomen	womenApoB4b	Age-sex adj Plasma ApoB Level Offsp wom ex4	http://www.ncbi.nlm.nih.gov/projects/gap/cgi-bin/analysis.cgi?id=pha000921
Metabolic	Lipidswomen	womenchol1a	Multivariable adj Chol Offsp wom ex1	http://www.ncbi.nlm.nih.gov/projects/gap/cgi-bin/analysis.cgi?id=pha000725
Metabolic	Lipidswomen	womenchol1b	Age-sex adj Chol Offsp wom ex1	http://www.ncbi.nlm.nih.gov/projects/gap/cgi-bin/analysis.cgi?id=pha000727
Metabolic	Lipidswomen	womenchol2b	Age-sex adj Chol Offsp wom ex2	http://www.ncbi.nlm.nih.gov/projects/gap/cgi-bin/analysis.cgi?id=pha000729
Metabolic	Lipidswomen	womenchol3b	Age-sex adj Chol Offsp wom ex3	http://www.ncbi.nlm.nih.gov/projects/gap/cgi-bin/analysis.cgi?id=pha000731
Metabolic	Lipidswomen	womenchol4b	Age-sex adj Chol Offsp wom ex4	http://www.ncbi.nlm.nih.gov/projects/gap/cgi-bin/analysis.cgi?id=pha000733
Metabolic	Lipidswomen	womenchol5b	Age-sex adj Chol Offsp wom ex5	http://www.ncbi.nlm.nih.gov/projects/gap/cgi-bin/analysis.cgi?id=pha000735
Metabolic	Lipidswomen	womenchol6b	Age-sex adj Chol Offsp wom ex6	http://www.ncbi.nlm.nih.gov/projects/gap/cgi-bin/analysis.cgi?id=pha000737
Metabolic	Lipidswomen	womenchol7b	Age-sex adj Chol Offsp wom ex7	http://www.ncbi.nlm.nih.gov/projects/gap/cgi-bin/analysis.cgi?id=pha000739
Metabolic	Lipidswomen	womenhdl1a	Multivariable adj HDL Offsp wom ex1	http://www.ncbi.nlm.nih.gov/projects/gap/cgi-bin/analysis.cgi?id=pha000757
Metabolic	Lipidswomen	womenhdl1b	Age-sex adj HDL Offsp wom ex1	http://www.ncbi.nlm.nih.gov/projects/gap/cgi-bin/analysis.cgi?id=pha000759
Metabolic	Lipidswomen	womenhdl2b	Age-sex adj HDL Offsp wom ex2	http://www.ncbi.nlm.nih.gov/projects/gap/cgi-bin/analysis.cgi?id=pha000761
Metabolic	Lipidswomen	womenhdl3b	Age-sex adj HDL Offsp wom ex3	http://www.ncbi.nlm.nih.gov/projects/gap/cgi-bin/analysis.cgi?id=pha000763
Metabolic	Lipidswomen	womenhdl4b	Age-sex adj HDL Offsp wom ex4	http://www.ncbi.nlm.nih.gov/projects/gap/cgi-bin/analysis.cgi?id=pha000765
Metabolic	Lipidswomen	womenhdl5b	Age-sex adj HDL Offsp wom ex5	http://www.ncbi.nlm.nih.gov/projects/gap/cgi-bin/analysis.cgi?id=pha000767
Metabolic	Lipidswomen	womenhdl6b	Age-sex adj HDL Offsp wom ex6	http://www.ncbi.nlm.nih.gov/projects/gap/cgi-bin/analysis.cgi?id=pha000769
Metabolic	Lipidswomen	womenhdl7b	Age-sex adj HDL Offsp wom ex7	http://www.ncbi.nlm.nih.gov/projects/gap/cgi-bin/analysis.cgi?id=pha000771
Metabolic	Lipidswomen	womenHDLNMRint4a	Multivariable adj NMR HDL Int Offsp wom ex4	http://www.ncbi.nlm.nih.gov/projects/gap/cgi-bin/analysis.cgi?id=pha000813
Metabolic	Lipidswomen	womenHDLNMRint4b	Age-sex adj NMR HDL Int Offsp wom ex4	http://www.ncbi.nlm.nih.gov/projects/gap/cgi-bin/analysis.cgi?id=pha000815
Metabolic	Lipidswomen	womenHDLNMRlg4a	Multivariable adj NMR HDL Lg Offsp wom ex4	http://www.ncbi.nlm.nih.gov/projects/gap/cgi-bin/analysis.cgi?id=pha000821
Metabolic	Lipidswomen	womenHDLNMRlg4b	Age-sex adj NMR HDL Lg Offsp wom ex4	http://www.ncbi.nlm.nih.gov/projects/gap/cgi-bin/analysis.cgi?id=pha000823
Metabolic	Lipidswomen	womenHDLNMRsm4a	Multivariable adj NMR HDL Sm Offsp wom ex4	http://www.ncbi.nlm.nih.gov/projects/gap/cgi-bin/analysis.cgi?id=pha000837
Metabolic	Lipidswomen	womenHDLNMRsm4b	Age-sex adj NMR HDL Sm Offsp wom ex4	http://www.ncbi.nlm.nih.gov/projects/gap/cgi-bin/analysis.cgi?id=pha000839
Metabolic	Lipidswomen	womenHDLNMRsz4a	Multivariable adj NMR HDL Size Offsp wom ex4	http://www.ncbi.nlm.nih.gov/projects/gap/cgi-bin/analysis.cgi?id=pha000829
Metabolic	Lipidswomen	womenHDLNMRsz4b	Age-sex adj NMR HDL Size Offsp wom ex4	http://www.ncbi.nlm.nih.gov/projects/gap/cgi-bin/analysis.cgi?id=pha000831
Metabolic	Lipidswomen	womenldl1a	Multivariable adj LDL Offsp wom ex1	http://www.ncbi.nlm.nih.gov/projects/gap/cgi-bin/analysis.cgi?id=pha000695
Metabolic	Lipidswomen	womenldl1b	Age-sex adj LDL Offsp wom ex1	http://www.ncbi.nlm.nih.gov/projects/gap/cgi-bin/analysis.cgi?id=pha000775
Metabolic	Lipidswomen	womenldl2b	Age-sex adj LDL Offsp wom ex2	http://www.ncbi.nlm.nih.gov/projects/gap/cgi-bin/analysis.cgi?id=pha000697
Metabolic	Lipidswomen	womenldl3b	Age-sex adj LDL Offsp wom ex3	http://www.ncbi.nlm.nih.gov/projects/gap/cgi-bin/analysis.cgi?id=pha000699
Metabolic	Lipidswomen	womenldl4b	Age-sex adj LDL Offsp wom ex4	http://www.ncbi.nlm.nih.gov/projects/gap/cgi-bin/analysis.cgi?id=pha000701
Metabolic	Lipidswomen	womenldl5b	Age-sex adj LDL Offsp wom ex5	http://www.ncbi.nlm.nih.gov/projects/gap/cgi-bin/analysis.cgi?id=pha000703
Metabolic	Lipidswomen	womenldl6b	Age-sex adj LDL Offsp wom ex6	http://www.ncbi.nlm.nih.gov/projects/gap/cgi-bin/analysis.cgi?id=pha000705
Metabolic	Lipidswomen	womenldl7b	Age-sex adj LDL Offsp wom ex7	http://www.ncbi.nlm.nih.gov/projects/gap/cgi-bin/analysis.cgi?id=pha000707
Metabolic	Lipidswomen	womenLDLNMRlg4a	Multivariable adj NMR LDL Lg Offsp wom ex4	http://www.ncbi.nlm.nih.gov/projects/gap/cgi-bin/analysis.cgi?id=pha000845
Metabolic	Lipidswomen	womenLDLNMRlg4b	Age-sex adj NMR LDL Lg Offsp wom ex4	http://www.ncbi.nlm.nih.gov/projects/gap/cgi-bin/analysis.cgi?id=pha000847
Metabolic	Lipidswomen	womenLDLNMRsm4a	Multivariable adj NMR LDL Sm Offsp wom ex4	http://www.ncbi.nlm.nih.gov/projects/gap/cgi-bin/analysis.cgi?id=pha000861
Metabolic	Lipidswomen	womenLDLNMRsm4b	Age-sex adj NMR LDL Sm Offsp wom ex4	http://www.ncbi.nlm.nih.gov/projects/gap/cgi-bin/analysis.cgi?id=pha000863
Metabolic	Lipidswomen	womenLDLNMRsz4a	Multivariable adj NMR LDL Size Offsp wom ex4	http://www.ncbi.nlm.nih.gov/projects/gap/cgi-bin/analysis.cgi?id=pha000853
Metabolic	Lipidswomen	womenLDLNMRsz4b	Age-sex adj NMR LDL Size Offsp wom ex4	http://www.ncbi.nlm.nih.gov/projects/gap/cgi-bin/analysis.cgi?id=pha000855
Metabolic	Lipidswomen	womenmeanchola	Multivariable adj Mean Chol Offsp wom ex1-7	http://www.ncbi.nlm.nih.gov/projects/gap/cgi-bin/analysis.cgi?id=pha000797
Metabolic	Lipidswomen	womenmeancholb	Age-sex adj Mean Chol Offsp wom ex1-7	http://www.ncbi.nlm.nih.gov/projects/gap/cgi-bin/analysis.cgi?id=pha000799
Metabolic	Lipidswomen	womenmeanhdla	Multivariable adj Mean HDL Offsp wom ex1-7	http://www.ncbi.nlm.nih.gov/projects/gap/cgi-bin/analysis.cgi?id=pha000805
Metabolic	Lipidswomen	womenmeanhdlb	Age-sex adj Mean HDL Offsp wom ex1-7	http://www.ncbi.nlm.nih.gov/projects/gap/cgi-bin/analysis.cgi?id=pha000807
Metabolic	Lipidswomen	womenmeanldla	Multivariable adj Mean LDL Offsp wom ex1-7	http://www.ncbi.nlm.nih.gov/projects/gap/cgi-bin/analysis.cgi?id=pha000789
Metabolic	Lipidswomen	womenmeanldlb	Age-sex adj Mean LDL Offsp wom ex1-7	http://www.ncbi.nlm.nih.gov/projects/gap/cgi-bin/analysis.cgi?id=pha000791
Metabolic	Lipidswomen	womenmeantga	Multivariable adj Mean TG Offsp wom ex1-7	http://www.ncbi.nlm.nih.gov/projects/gap/cgi-bin/analysis.cgi?id=pha000781

Group	Category	Trait Label	Name	FBAT Link
Metabolic	Lipidswomen	womenmeantgb	Age-sex adj Mean TG Offsp wom ex1-7	http://www.ncbi.nlm.nih.gov/projects/gap/cgi-bin/analysis.cgi?id=pha000783
Metabolic	Lipidswomen	womentg1a	Multivariable adj TG Offsp wom ex1	http://www.ncbi.nlm.nih.gov/projects/gap/cgi-bin/analysis.cgi?id=pha000651
Metabolic	Lipidswomen	womentg1b	Age-sex adj TG Offsp wom ex1	http://www.ncbi.nlm.nih.gov/projects/gap/cgi-bin/analysis.cgi?id=pha000653
Metabolic	Lipidswomen	womentg2b	Age-sex adj TG Offsp wom ex2	http://www.ncbi.nlm.nih.gov/projects/gap/cgi-bin/analysis.cgi?id=pha000655
Metabolic	Lipidswomen	womentg3b	Age-sex adj TG Offsp wom ex3	http://www.ncbi.nlm.nih.gov/projects/gap/cgi-bin/analysis.cgi?id=pha000657
Metabolic	Lipidswomen	womentg4b	Age-sex adj TG Offsp wom ex4	http://www.ncbi.nlm.nih.gov/projects/gap/cgi-bin/analysis.cgi?id=pha000659
Metabolic	Lipidswomen	womentg5b	Age-sex adj TG Offsp wom ex5	http://www.ncbi.nlm.nih.gov/projects/gap/cgi-bin/analysis.cgi?id=pha000661
Metabolic	Lipidswomen	womentg6b	Age-sex adj TG Offsp wom ex6	http://www.ncbi.nlm.nih.gov/projects/gap/cgi-bin/analysis.cgi?id=pha000663
Metabolic	Lipidswomen	womentg7b	Age-sex adj TG Offsp wom ex7	http://www.ncbi.nlm.nih.gov/projects/gap/cgi-bin/analysis.cgi?id=pha000665
Metabolic	Lipidswomen	womenVLDLNMRint4a	Multivariable adj NMR VLDL Int Offsp wom ex4	http://www.ncbi.nlm.nih.gov/projects/gap/cgi-bin/analysis.cgi?id=pha000873
Metabolic	Lipidswomen	womenVLDLNMRint4b	Age-sex adj NMR VLDL Int Offsp wom ex4	http://www.ncbi.nlm.nih.gov/projects/gap/cgi-bin/analysis.cgi?id=pha000875
Metabolic	Lipidswomen	womenVLDLNMRlg4a	Multivariable adj NMR VLDL Lg Offsp wom ex4	http://www.ncbi.nlm.nih.gov/projects/gap/cgi-bin/analysis.cgi?id=pha000885
Metabolic	Lipidswomen	womenVLDLNMRlg4b	Age-sex adj NMR VLDL Lg Offsp wom ex4	http://www.ncbi.nlm.nih.gov/projects/gap/cgi-bin/analysis.cgi?id=pha000887
Metabolic	Lipidswomen	womenVLDLNMRsm4a	Multivariable adj NMR VLDL Sm Offsp wom ex4	http://www.ncbi.nlm.nih.gov/projects/gap/cgi-bin/analysis.cgi?id=pha000909
Metabolic	Lipidswomen	womenVLDLNMRsm4b	Age-sex NMR VLDL Sm Offsp wom ex4	http://www.ncbi.nlm.nih.gov/projects/gap/cgi-bin/analysis.cgi?id=pha000911
Metabolic	Lipidswomen	womenVLDLNMRsz4a	Multivariable adj NMR VLDL Size Offsp wom ex4	http://www.ncbi.nlm.nih.gov/projects/gap/cgi-bin/analysis.cgi?id=pha000897
Metabolic	Lipidswomen	womenVLDLNMRsz4b	Age-sex adj NMR VLDL Size Offsp wom ex4	http://www.ncbi.nlm.nih.gov/projects/gap/cgi-bin/analysis.cgi?id=pha000899
Pulmonary	Circadian	bedtime	usual weekday bedtime unadjusted	http://www.ncbi.nlm.nih.gov/projects/gap/cgi-bin/analysis.cgi?id=pha000005
Pulmonary	Circadian	bedtimeresid	usual weekday bedtime adjusted	http://www.ncbi.nlm.nih.gov/projects/gap/cgi-bin/analysis.cgi?id=pha000007
Pulmonary	Circadian	sleepdur	usual weekday sleep duration unadjusted	http://www.ncbi.nlm.nih.gov/projects/gap/cgi-bin/analysis.cgi?id=pha000009
Pulmonary	Circadian	sleepdurresid	usual weekday sleep duration adjusted	http://www.ncbi.nlm.nih.gov/projects/gap/cgi-bin/analysis.cgi?id=pha000011
Pulmonary	longitudinal	fef2575long	rate of decline of FEF(25-75)	http://www.ncbi.nlm.nih.gov/projects/gap/cgi-bin/analysis.cgi?id=pha000013
Pulmonary	longitudinal	fef2575longsmoke10	rate of decline of FEF(25-75) in 10+ packyear smokers	http://www.ncbi.nlm.nih.gov/projects/gap/cgi-bin/analysis.cgi?id=pha000015
Pulmonary	longitudinal	feffvclong	rate of decline of FEF(25-75)/FVC	http://www.ncbi.nlm.nih.gov/projects/gap/cgi-bin/analysis.cgi?id=pha000017
Pulmonary	longitudinal	feffvclongsmoke10	rate of decline of FEF(25-75)/FVC in 10+ packyear smokers	http://www.ncbi.nlm.nih.gov/projects/gap/cgi-bin/analysis.cgi?id=pha000019
Pulmonary	longitudinal	fev1fvclong	rate of decline of FEV1/FVC	http://www.ncbi.nlm.nih.gov/projects/gap/cgi-bin/analysis.cgi?id=pha000021
Pulmonary	longitudinal	fev1fvclongsmoke10	rate of decline of FEV1/FVC in 10+ packyear smokers	http://www.ncbi.nlm.nih.gov/projects/gap/cgi-bin/analysis.cgi?id=pha000023
Pulmonary	longitudinal	fev1long	rate of decline of FEV1	http://www.ncbi.nlm.nih.gov/projects/gap/cgi-bin/analysis.cgi?id=pha000025
Pulmonary	longitudinal	fev1longsmoke10	rate of decline of FEV1 in 10+ packyear smokers	http://www.ncbi.nlm.nih.gov/projects/gap/cgi-bin/analysis.cgi?id=pha000027
Pulmonary	longitudinal	fvclong	rate of decline of FVC	http://www.ncbi.nlm.nih.gov/projects/gap/cgi-bin/analysis.cgi?id=pha000029
Pulmonary	longitudinal	fvclongsmoke10	rate of decline of FVC in 10+ packyear smokers	http://www.ncbi.nlm.nih.gov/projects/gap/cgi-bin/analysis.cgi?id=pha000031
Pulmonary	mean	genomefev1	mean FEV1 from 2 examinations	http://www.ncbi.nlm.nih.gov/projects/gap/cgi-bin/analysis.cgi?id=pha000033
Pulmonary	mean	genomefvc	mean FVC from 2 examinations	http://www.ncbi.nlm.nih.gov/projects/gap/cgi-bin/analysis.cgi?id=pha000035
Pulmonary	mean	genomeratio	mean FEV1/FVC from 2 examinations	http://www.ncbi.nlm.nih.gov/projects/gap/cgi-bin/analysis.cgi?id=pha000037
Pulmonary	ppcrosssectional	ppfefadj	percent predicted FEF(25-75) at latest exam	http://www.ncbi.nlm.nih.gov/projects/gap/cgi-bin/analysis.cgi?id=pha000039
Pulmonary	ppcrosssectional	ppfefadjsm10	percent predicted FEF(25-75) at latest exam in 10+ packyear smokers	http://www.ncbi.nlm.nih.gov/projects/gap/cgi-bin/analysis.cgi?id=pha000041
Pulmonary	ppcrosssectional	ppfefratadj	percent predicted FEF(25-75)/FVC at latest exam	http://www.ncbi.nlm.nih.gov/projects/gap/cgi-bin/analysis.cgi?id=pha000043
Pulmonary	ppcrosssectional	ppfefratadjsm10	percent predicted FEF(25-75)/FVC latest exam, 10+ packyear smokers	http://www.ncbi.nlm.nih.gov/projects/gap/cgi-bin/analysis.cgi?id=pha000045
Pulmonary	ppcrosssectional	ppfev1adj	percent predicted FEV1 at latest exam	http://www.ncbi.nlm.nih.gov/projects/gap/cgi-bin/analysis.cgi?id=pha000047
Pulmonary	ppcrosssectional	ppfev1adjsm10	percent predicted FEV1 at latest exam in 10+ packyear smokers	http://www.ncbi.nlm.nih.gov/projects/gap/cgi-bin/analysis.cgi?id=pha000049
Pulmonary	ppcrosssectional	ppfvcadj	percent predicted FVC at latest exam	http://www.ncbi.nlm.nih.gov/projects/gap/cgi-bin/analysis.cgi?id=pha000051
Pulmonary	ppcrosssectional	ppfvcadjsm10	percent predicted FVC at latest exam in 10+ packyear smokers	http://www.ncbi.nlm.nih.gov/projects/gap/cgi-bin/analysis.cgi?id=pha000053
Pulmonary	ppcrosssectional	ppratioadj	percent predicted FEV1/FVC at latest exam	http://www.ncbi.nlm.nih.gov/projects/gap/cgi-bin/analysis.cgi?id=pha000055
Pulmonary	ppcrosssectional	ppratioadjsm10	percent predicted FEV1/FVC at latest exam in 10+ packyear smokers	http://www.ncbi.nlm.nih.gov/projects/gap/cgi-bin/analysis.cgi?id=pha000057
Pulmonary	rcrosssectional	rfefadj	residual from predicted FEF(25-75) at latest exam	http://www.ncbi.nlm.nih.gov/projects/gap/cgi-bin/analysis.cgi?id=pha000059
Pulmonary	rcrosssectional	rfefadjsm10	residual from predicted FEF(25-75) at latest exam in 10+ packyear smokers	http://www.ncbi.nlm.nih.gov/projects/gap/cgi-bin/analysis.cgi?id=pha000061
Pulmonary	rcrosssectional	rfefratadj	residual from predicted FEF(25-75)/FVC at latest exam	http://www.ncbi.nlm.nih.gov/projects/gap/cgi-bin/analysis.cgi?id=pha000063
Pulmonary	rcrosssectional	rfefratadjsm10	residual from predicted FEF(25-75)/FVC latest exam in 10+ packyear smokers	http://www.ncbi.nlm.nih.gov/projects/gap/cgi-bin/analysis.cgi?id=pha000065
Pulmonary	rcrosssectional	rfev1adj	residual from predicted FEV1 at latest exam	http://www.ncbi.nlm.nih.gov/projects/gap/cgi-bin/analysis.cgi?id=pha000067
Pulmonary	rcrosssectional	rfev1adjsm10	residual from predicted FEV1 at latest exam in 10+ packyear smokers	http://www.ncbi.nlm.nih.gov/projects/gap/cgi-bin/analysis.cgi?id=pha000069
Pulmonary	rcrosssectional	rfvcadj	residual from predicted FVC at latest exam	http://www.ncbi.nlm.nih.gov/projects/gap/cgi-bin/analysis.cgi?id=pha000071
Pulmonary	rcrosssectional	rfvcadjsm10	residual from predicted FVC at latest exam in 10+ packyear smokers	http://www.ncbi.nlm.nih.gov/projects/gap/cgi-bin/analysis.cgi?id=pha000073
Pulmonary	rcrosssectional	rratioadj	residual from predicted FEV1/FVC at latest exam	http://www.ncbi.nlm.nih.gov/projects/gap/cgi-bin/analysis.cgi?id=pha000075
Pulmonary	rcrosssectional	rratioadjsm10	residual from predicted FEV1/FVC at latest exam in 10+ packyear smokers	http://www.ncbi.nlm.nih.gov/projects/gap/cgi-bin/analysis.cgi?id=pha000077
Pulmonary	SDB	rdiresid	apnea-hypopnea index with 4% desaturation adjusted	http://www.ncbi.nlm.nih.gov/projects/gap/cgi-bin/analysis.cgi?id=pha000079
Pulmonary	singleexam	ppfev1single	percent predicted FEV1, exam: offspring 6, cohort 17	http://www.ncbi.nlm.nih.gov/projects/gap/cgi-bin/analysis.cgi?id=pha000081

Group	Category	Trait Label	Name	FBAT Link
Pulmonary	singleexam	ppfvcsingle	percent predicted FVC, exam: offspring 6, cohort 17	http://www.ncbi.nlm.nih.gov/projects/gap/cgi-bin/analysis.cgi?id=pha000083
Pulmonary	singleexam	ppratiosingle	percent predicted FEV1/FVC, exam: offspring 6, cohort 17	http://www.ncbi.nlm.nih.gov/projects/gap/cgi-bin/analysis.cgi?id=pha000085
Pulmonary	singleexam	rfev1single	residual from predicted FEV1, exam: offspring 6, cohort 17	http://www.ncbi.nlm.nih.gov/projects/gap/cgi-bin/analysis.cgi?id=pha000087
Pulmonary	singleexam	rfvcsingle	residual from predicted FVC, exam: offspring 6, cohort 17	http://www.ncbi.nlm.nih.gov/projects/gap/cgi-bin/analysis.cgi?id=pha000089
Pulmonary	singleexam	rratiosingle	residual from predicted FEV1/FVC, exam: offspring 6, cohort 17	http://www.ncbi.nlm.nih.gov/projects/gap/cgi-bin/analysis.cgi?id=pha000091
Pulmonary	Sleepiness	ess	Epworth Sleepiness Scale, unadjusted	http://www.ncbi.nlm.nih.gov/projects/gap/cgi-bin/analysis.cgi?id=pha000093
Pulmonary	Sleepiness	essresid1	Epworth Sleepiness Scale, adjusted A	http://www.ncbi.nlm.nih.gov/projects/gap/cgi-bin/analysis.cgi?id=pha000095
Pulmonary	Sleepiness	essresid2	Epworth Sleepiness Scale, adjusted B	http://www.ncbi.nlm.nih.gov/projects/gap/cgi-bin/analysis.cgi?id=pha000097
Pulmonary	Sleepiness	essresid3	Epworth Sleepiness Scale, adjusted C	http://www.ncbi.nlm.nih.gov/projects/gap/cgi-bin/analysis.cgi?id=pha000099
RENALENDO	ENDO	DHEASAS3	DHEAS adjusted for age and sex	http://www.ncbi.nlm.nih.gov/projects/gap/cgi-bin/analysis.cgi?id=pha001219
RENALENDO	ENDO	DHEASMV3	DHEAS MV adjusted	http://www.ncbi.nlm.nih.gov/projects/gap/cgi-bin/analysis.cgi?id=pha001221
RENALENDO	ENDO	FSHAS3	FSH in men or post meno women no hormones, age-sex adjusted	http://www.ncbi.nlm.nih.gov/projects/gap/cgi-bin/analysis.cgi?id=pha001223
RENALENDO	ENDO	FSHMV3	FSH in men or post meno women no hormones, MV adjusted	http://www.ncbi.nlm.nih.gov/projects/gap/cgi-bin/analysis.cgi?id=pha001225
RENALENDO	ENDO	LHAS3	LH in men or post-meno women no hormones, age-sex adjusted	http://www.ncbi.nlm.nih.gov/projects/gap/cgi-bin/analysis.cgi?id=pha001227
RENALENDO	ENDO	LHMV3	LH in men or post-meno women no hormones, MV adjusted	http://www.ncbi.nlm.nih.gov/projects/gap/cgi-bin/analysis.cgi?id=pha001229
RENALENDO	ENDO	TSHLNAS3	TSH from ex3 log transformed, age-sex adjusted	http://www.ncbi.nlm.nih.gov/projects/gap/cgi-bin/analysis.cgi?id=pha001231
RENALENDO	ENDO	TSHLNAS4	TSH from ex4 log transformed, age-sex adjusted	http://www.ncbi.nlm.nih.gov/projects/gap/cgi-bin/analysis.cgi?id=pha001233
RENALENDO	ENDO	TSHLNMV3	TSH ex3 log transformed, MV adjusted	http://www.ncbi.nlm.nih.gov/projects/gap/cgi-bin/analysis.cgi?id=pha001235
RENALENDO	ENDO	TSHLNMV4	TSH ex4 log transformed, MV adjusted	http://www.ncbi.nlm.nih.gov/projects/gap/cgi-bin/analysis.cgi?id=pha001237
RENALENDO	ENDO	TSHMEAN34LNAS	Mean TSH log transformed, mean of ex3 and ex4, age sex adjusted	http://www.ncbi.nlm.nih.gov/projects/gap/cgi-bin/analysis.cgi?id=pha001239
RENALENDO	ENDO	TSHMEAN34LNMV	Mean TSH ex3 and 4, MV adjusted	http://www.ncbi.nlm.nih.gov/projects/gap/cgi-bin/analysis.cgi?id=pha001241
RENALENDO	RENAL	CALCIUMAS2	Calcium, ex2, age-sex adjusted	http://www.ncbi.nlm.nih.gov/projects/gap/cgi-bin/analysis.cgi?id=pha001243
RENALENDO	RENAL	CALCIUMMV2	Calcium, ex2, age-sex-creatinine adjusted	http://www.ncbi.nlm.nih.gov/projects/gap/cgi-bin/analysis.cgi?id=pha001245
RENALENDO	RENAL	CHNGSCR27ASWIN	Change in creatinine ex2 to 7, age-sex adjusted	http://www.ncbi.nlm.nih.gov/projects/gap/cgi-bin/analysis.cgi?id=pha001247
RENALENDO	RENAL	CHNGSCR27MVWIN	Change in creatinine ex2 to 7, MV adjusted	http://www.ncbi.nlm.nih.gov/projects/gap/cgi-bin/analysis.cgi?id=pha001249
RENALENDO	RENAL	CKDAS7	CKD ex 7, age-sex adjusted	http://www.ncbi.nlm.nih.gov/projects/gap/cgi-bin/analysis.cgi?id=pha001251
RENALENDO	RENAL	CKDMV7	CKD ex 7, MV adjusted	http://www.ncbi.nlm.nih.gov/projects/gap/cgi-bin/analysis.cgi?id=pha001253
RENALENDO	RENAL	CYSCAS7	Cystatin C, ex 7, age-sex adjusted	http://www.ncbi.nlm.nih.gov/projects/gap/cgi-bin/analysis.cgi?id=pha001327
RENALENDO	RENAL	CYSCMV7	Cystatin C, ex 7, MV adjusted	http://www.ncbi.nlm.nih.gov/projects/gap/cgi-bin/analysis.cgi?id=pha001329
RENALENDO	RENAL	GFRAS5	GFR ex 5, age-sex adjusted	http://www.ncbi.nlm.nih.gov/projects/gap/cgi-bin/analysis.cgi?id=pha001255
RENALENDO	RENAL	GFRAS6	GFR ex 6, age-sex adjusted	http://www.ncbi.nlm.nih.gov/projects/gap/cgi-bin/analysis.cgi?id=pha001257
RENALENDO	RENAL	GFRAS7	GFR ex 7, age-sex adjusted	http://www.ncbi.nlm.nih.gov/projects/gap/cgi-bin/analysis.cgi?id=pha001259
RENALENDO	RENAL	GFRASWIN2	GFR ex2, winsorized, age-sex adjusted	http://www.ncbi.nlm.nih.gov/projects/gap/cgi-bin/analysis.cgi?id=pha001261
RENALENDO	RENAL	GFRMV5	GFR ex5, MV adjusted	http://www.ncbi.nlm.nih.gov/projects/gap/cgi-bin/analysis.cgi?id=pha001263
RENALENDO	RENAL	GFRMV6	GFR ex6, MV adjusted	http://www.ncbi.nlm.nih.gov/projects/gap/cgi-bin/analysis.cgi?id=pha001265
RENALENDO	RENAL	GFRMV7	GFR ex7, MV adjusted	http://www.ncbi.nlm.nih.gov/projects/gap/cgi-bin/analysis.cgi?id=pha001267
RENALENDO	RENAL	GFRMVWIN2	GFR ex2, winsorized, MV adjusted	http://www.ncbi.nlm.nih.gov/projects/gap/cgi-bin/analysis.cgi?id=pha001269
RENALENDO	RENAL	MeanGFRAS2567WIN	Mean GFR ex 2,5,6,7 age-sex adjusted	http://www.ncbi.nlm.nih.gov/projects/gap/cgi-bin/analysis.cgi?id=pha001271
RENALENDO	RENAL	MeanGFRMV2567WIN	Mean GFR ex 2,5,6,7,MV adjusted	http://www.ncbi.nlm.nih.gov/projects/gap/cgi-bin/analysis.cgi?id=pha001273
RENALENDO	RENAL	MeanSCR2567LNASWIN	Mean creatinine ex 2,5,6,7 age-sex adjusted	http://www.ncbi.nlm.nih.gov/projects/gap/cgi-bin/analysis.cgi?id=pha001275
RENALENDO	RENAL	MeanSCR2567LNMVWIN	Mean creatinine ex 2,5,6,7, MV adjusted	http://www.ncbi.nlm.nih.gov/projects/gap/cgi-bin/analysis.cgi?id=pha001277
RENALENDO	RENAL	MeanURICACID12AS	Mean uric acid, ex 1 and 2, age-sex adjusted	http://www.ncbi.nlm.nih.gov/projects/gap/cgi-bin/analysis.cgi?id=pha001279
RENALENDO	RENAL	MeanURICACID12MV	Mean uric acid, ex 1 and 2, MV adjusted	http://www.ncbi.nlm.nih.gov/projects/gap/cgi-bin/analysis.cgi?id=pha001281
RENALENDO	RENAL	PHOSAS2	Phos, ex 2, age-sex adjusted	http://www.ncbi.nlm.nih.gov/projects/gap/cgi-bin/analysis.cgi?id=pha001283
RENALENDO	RENAL	PHOSMV2	Phos, ex 2, MV adjusted	http://www.ncbi.nlm.nih.gov/projects/gap/cgi-bin/analysis.cgi?id=pha001285
RENALENDO	RENAL	SCRLNAS5	Creatinine, ex5, age-sex adjusted	http://www.ncbi.nlm.nih.gov/projects/gap/cgi-bin/analysis.cgi?id=pha001287
RENALENDO	RENAL	SCRLNAS6	Creatinine, ex6, age-sex adjusted	http://www.ncbi.nlm.nih.gov/projects/gap/cgi-bin/analysis.cgi?id=pha001289
RENALENDO	RENAL	SCRLNASWIN2	Creatinine ex2, log transformed and winsorized, age-sex adjusted	http://www.ncbi.nlm.nih.gov/projects/gap/cgi-bin/analysis.cgi?id=pha001291
RENALENDO	RENAL	SCRLNASWIN7	Creatinine ex7, log transformed and winsorized, age-sex adjusted	http://www.ncbi.nlm.nih.gov/projects/gap/cgi-bin/analysis.cgi?id=pha001293
RENALENDO	RENAL	SCRLNMV5	GFR ex 5 uncalibrated creatinine, MV adjusted	http://www.ncbi.nlm.nih.gov/projects/gap/cgi-bin/analysis.cgi?id=pha001295
RENALENDO	RENAL	SCRLNMV6	GFR ex 6 uncalibrated creatinine, MV adjusted	http://www.ncbi.nlm.nih.gov/projects/gap/cgi-bin/analysis.cgi?id=pha001297
RENALENDO	RENAL	SCRLNMVWIN2	Creatinine ex 2 log transformed winsorized MV adjusted	http://www.ncbi.nlm.nih.gov/projects/gap/cgi-bin/analysis.cgi?id=pha001299
RENALENDO	RENAL	SCRLNMVWIN7	Creatinine ex 7 uncalibrated MV adjusted	http://www.ncbi.nlm.nih.gov/projects/gap/cgi-bin/analysis.cgi?id=pha001301
RENALENDO	RENAL	UAEGE30HTNAS6	UAE of at least 30 in enriched hypertensive sample, age-sex adjusted	http://www.ncbi.nlm.nih.gov/projects/gap/cgi-bin/analysis.cgi?id=pha001303
RENALENDO	RENAL	UAEGE30HTNMV6	UAE of at least 30 in enriched hypertensive sample, age-sex adjusted	http://www.ncbi.nlm.nih.gov/projects/gap/cgi-bin/analysis.cgi?id=pha001305
RENALENDO	RENAL	UAELNAS6	Log-transformed UAE, age-sex adjusted	http://www.ncbi.nlm.nih.gov/projects/gap/cgi-bin/analysis.cgi?id=pha001307

Group	Category	Trait Label	Name	FBAT Link
RENALENDO	RENAL	UAELNHTNAS6	Log-transformed UAE in HTN enriched sample, age-sex adjusted	http://www.ncbi.nlm.nih.gov/projects/gap/cgi-bin/analysis.cgi?id=pha001309
RENALENDO	RENAL	UAELNHTNMV6	Log-transformed UAE in HTN enriched sample, MV adjusted	http://www.ncbi.nlm.nih.gov/projects/gap/cgi-bin/analysis.cgi?id=pha001311
RENALENDO	RENAL	UAELNMV6	Log-transformed UAE, MV adjusted	http://www.ncbi.nlm.nih.gov/projects/gap/cgi-bin/analysis.cgi?id=pha001313
RENALENDO	RENAL	UNALNASWIN6	Urinary sodium, age-sex adjusted	http://www.ncbi.nlm.nih.gov/projects/gap/cgi-bin/analysis.cgi?id=pha001315
RENALENDO	RENAL	UNALNMVWIN6	Urinary sodium, MV adjusted	http://www.ncbi.nlm.nih.gov/projects/gap/cgi-bin/analysis.cgi?id=pha001317
RENALENDO	RENAL	URICACIDAS1	Uric acid, ex 1, age-sex adjusted	http://www.ncbi.nlm.nih.gov/projects/gap/cgi-bin/analysis.cgi?id=pha001319
RENALENDO	RENAL	URICACIDAS2	Uric acid, ex 2, age-sex adjusted	http://www.ncbi.nlm.nih.gov/projects/gap/cgi-bin/analysis.cgi?id=pha001321
RENALENDO	RENAL	URICACIDMV1	Uric acid, ex1, MV adjusted	http://www.ncbi.nlm.nih.gov/projects/gap/cgi-bin/analysis.cgi?id=pha001323
RENALENDO	RENAL	URICACIDMV2	Uric acid, ex2, MV adjusted	http://www.ncbi.nlm.nih.gov/projects/gap/cgi-bin/analysis.cgi?id=pha001325
SubclinCVD	Ankle-brachialindex	RANKLEBI6	Ankle-brachial index, cycle 6, age and sex-djusted	http://www.ncbi.nlm.nih.gov/projects/gap/cgi-bin/analysis.cgi?id=pha000927
SubclinCVD	Ankle-brachialindex	RANKLEBI6MV	Ankle-brachial index, cycle 6, multivariable-adjusted	http://www.ncbi.nlm.nih.gov/projects/gap/cgi-bin/analysis.cgi?id=pha000929
SubclinCVD	Ankle-brachialindex	RANKLEBI7	Ankle-brachial index, cycle 7, age- and sex-adjusted	http://www.ncbi.nlm.nih.gov/projects/gap/cgi-bin/analysis.cgi?id=pha000931
SubclinCVD	Ankle-brachialindex	RANKLEBI7MV	Ankle-brachial index, cycle 7, multivariable-adjusted	http://www.ncbi.nlm.nih.gov/projects/gap/cgi-bin/analysis.cgi?id=pha000933
SubclinCVD	BrainMRI	BMRILWMHVC	Log white matter hyperintensity to intracranial volume ratio, age and sex	http://www.ncbi.nlm.nih.gov/projects/gap/cgi-bin/analysis.cgi?id=pha000935
SubclinCVD	BrainMRI	BMRILWMHVMV	Log white matter hyperintensity to intracranial volume ratio, multivariable	http://www.ncbi.nlm.nih.gov/projects/gap/cgi-bin/analysis.cgi?id=pha000937
SubclinCVD	BrainMRI	BMRIZLWMHVC	Z-score log white matter hyperintensity volume ratio, sex adjusted	http://www.ncbi.nlm.nih.gov/projects/gap/cgi-bin/analysis.cgi?id=pha000939
SubclinCVD	BrainMRI	BMRIZLWMHVMV	Z-score log white matter hyperintensity volume ratio, multivariable	http://www.ncbi.nlm.nih.gov/projects/gap/cgi-bin/analysis.cgi?id=pha000941
SubclinCVD	Carotid	RNKCAROTBULBAS6	Carotid bulb IMT mean max, cycle 6, log transformed age and sex-adjusted	http://www.ncbi.nlm.nih.gov/projects/gap/cgi-bin/analysis.cgi?id=pha000943
SubclinCVD	Carotid	RNKCAROTBULBMV6	Carotid bulb IMT mean max, cycle 6, log transformed multivariable-adjusted	http://www.ncbi.nlm.nih.gov/projects/gap/cgi-bin/analysis.cgi?id=pha000945
SubclinCVD	Carotid	RNKCAROTCCAMAXAS6	Common carotid IMT mean max, cycle 6, log transformed age and sex-adjusted	http://www.ncbi.nlm.nih.gov/projects/gap/cgi-bin/analysis.cgi?id=pha000947
SubclinCVD	Carotid	RNKCAROTCCAMAXMV6	Common carotid IMT mean max, cycle 6, log transformed multivariable-adjusted	http://www.ncbi.nlm.nih.gov/projects/gap/cgi-bin/analysis.cgi?id=pha000949
SubclinCVD	Carotid	RNKCAROTCCAMEANAS6	Common carotid IMT mean mean, cycle 6, log transformed age and sex-adjusted	http://www.ncbi.nlm.nih.gov/projects/gap/cgi-bin/analysis.cgi?id=pha000951
SubclinCVD	Carotid	RNKCAROTCCAMEANMV6	Common carotid IMT mean mean, cycle 6, log transformed multivariable-adjusted	http://www.ncbi.nlm.nih.gov/projects/gap/cgi-bin/analysis.cgi?id=pha000953
SubclinCVD	Carotid	RNKCAROTICAMAXAS6	Internal carotid IMT mean max, cycle 6, log transformed age and sex-adjusted	http://www.ncbi.nlm.nih.gov/projects/gap/cgi-bin/analysis.cgi?id=pha000955
SubclinCVD	Carotid	RNKCAROTICAMAXMV6	Internal carotid IMT mean max, cycle 6, log transformed multivariable-adjusted	http://www.ncbi.nlm.nih.gov/projects/gap/cgi-bin/analysis.cgi?id=pha000957
SubclinCVD	Carotid	RNKCAROTICAMEANAS6	Internal carotid IMT mean mean, cycle 6, log transformed age and sex-adjusted	http://www.ncbi.nlm.nih.gov/projects/gap/cgi-bin/analysis.cgi?id=pha000959
SubclinCVD	Carotid	RNKCAROTICAMEANMV6	Internal carotid IMT mean mean, cycle 6, log transformed multivariable-adjusted	http://www.ncbi.nlm.nih.gov/projects/gap/cgi-bin/analysis.cgi?id=pha000961
SubclinCVD	Carotid	RNKCAROTSTENAS6	Maximum Carotid Stenosis, cycle 6, age and sex-adjusted	http://www.ncbi.nlm.nih.gov/projects/gap/cgi-bin/analysis.cgi?id=pha000963
SubclinCVD	Carotid	RNKCAROTSTENMV6	Maximum Carotid Stenosis, cycle 6 multivariable-adjusted	http://www.ncbi.nlm.nih.gov/projects/gap/cgi-bin/analysis.cgi?id=pha000965
SubclinCVD	CT	RESMDCTAACAS7	Mean Agatston AAC score, MDCT ~cycle 7, log transform, age and sex-adjusted	http://www.ncbi.nlm.nih.gov/projects/gap/cgi-bin/analysis.cgi?id=pha000967
SubclinCVD	CT	RESMDCTAACMV7	Mean Agatston AAC score, MDCT ~cycle 7, log transform multivariable-adjusted	http://www.ncbi.nlm.nih.gov/projects/gap/cgi-bin/analysis.cgi?id=pha000969
SubclinCVD	CT	RESMDCTCACAS7	Mean Agatston CAC score, MDCT ~cycle 7, log transform age and sex-adjusted	http://www.ncbi.nlm.nih.gov/projects/gap/cgi-bin/analysis.cgi?id=pha000971
SubclinCVD	CT	RESMDCTCACMAXAS7	Max Agatston CAC score, MDCT ~cycle 7, log transform age and sex-adjusted	http://www.ncbi.nlm.nih.gov/projects/gap/cgi-bin/analysis.cgi?id=pha000973
SubclinCVD	CT	RESMDCTCACMAXMV7	Max Agatston CAC score, MDCT ~cycle 7, log transform multivariable-adjusted	http://www.ncbi.nlm.nih.gov/projects/gap/cgi-bin/analysis.cgi?id=pha000975
SubclinCVD	CT	RESMDCTCACMV7	Mean Agatston CAC score, MDCT ~cycle 7, log transform multivariable-adjusted	http://www.ncbi.nlm.nih.gov/projects/gap/cgi-bin/analysis.cgi?id=pha000977
