# Supplementary material for: The Framingham Heart Study 100K SNP genome-wide association study resource: overview of 17 phenotype working group reports
Source: BMC Med Genet. 2007 Sep 19;8(Suppl 1):S1. doi: 10.1186/1471-2350-8-S1-S1 (PMC1995613; doi:10.1186/1471-2350-8-S1-S1)
Supplement: Additional file 3 — Phenotypes for linkage analyses. [file 1471-2350-8-S1-S1-S3.pdf]

Group	Category	Trait Label	Name	Linkage Link
Aging	BoneAging	BUA	Bone Ultrasound attenuation measured by QUS, multivariable adjusted	http://www.ncbi.nlm.nih.gov/projects/gap/cgi-bin/analysis.cgi?id=pha001997
Aging	BoneAging	BUAof	Bone Ultrasound attenuation (Offspring), multivariable adjusted	http://www.ncbi.nlm.nih.gov/projects/gap/cgi-bin/analysis.cgi?id=pha002014
Aging	BoneAging	deltaOSSR	Bone age by osseographic scoring system, multivariable adjusted	http://www.ncbi.nlm.nih.gov/projects/gap/cgi-bin/analysis.cgi?id=pha002015
Aging	BoneAging	FNBMD	Femoral Neck BMD measured by DXA, multivariable adjusted	http://www.ncbi.nlm.nih.gov/projects/gap/cgi-bin/analysis.cgi?id=pha001998
Aging	BoneAging	FNBMDof	Femoral Neck BMD measured by DXA (Offspring), multivariable adjusted	http://www.ncbi.nlm.nih.gov/projects/gap/cgi-bin/analysis.cgi?id=pha002016
Aging	BoneAging	LSBMD	Lumbar Spine BMD measured by DXA, multivariable adjusted	http://www.ncbi.nlm.nih.gov/projects/gap/cgi-bin/analysis.cgi?id=pha001999
Aging	BoneAging	LSBMDof	Spine BMD measured by DXA (Offspring), multivariable adjusted	http://www.ncbi.nlm.nih.gov/projects/gap/cgi-bin/analysis.cgi?id=pha002019
Aging	BoneAging	NeckCSMI	Hip geometry by DXA (Neck Moment of Inertia), age- and sex-adjusted	http://www.ncbi.nlm.nih.gov/projects/gap/cgi-bin/analysis.cgi?id=pha002000
Aging	BoneAging	NeckCSMI1	Hip geometry by DXA (Neck Moment of Inertia), multivariable adjusted	http://www.ncbi.nlm.nih.gov/projects/gap/cgi-bin/analysis.cgi?id=pha002001
Aging	BoneAging	NeckLeng	Hip geometry by DXA (Neck Length), age- and sex-adjusted	http://www.ncbi.nlm.nih.gov/projects/gap/cgi-bin/analysis.cgi?id=pha002002
Aging	BoneAging	NeckLeng1	Hip geometry by DXA (Neck Length), multivariable adjusted	http://www.ncbi.nlm.nih.gov/projects/gap/cgi-bin/analysis.cgi?id=pha002003
Aging	BoneAging	NeckW	Hip geometry by DXA (Neck Width), age- and sex-adjusted	http://www.ncbi.nlm.nih.gov/projects/gap/cgi-bin/analysis.cgi?id=pha002004
Aging	BoneAging	NeckW1	Hip geometry by DXA (Neck Width), multivariable adjusted	http://www.ncbi.nlm.nih.gov/projects/gap/cgi-bin/analysis.cgi?id=pha002005
Aging	BoneAging	NeckZ	Hip geometry by DXA (Neck Section Modulus), age- and sex-adjusted	http://www.ncbi.nlm.nih.gov/projects/gap/cgi-bin/analysis.cgi?id=pha002006
Aging	BoneAging	NeckZ1	Hip geometry by DXA (Neck Section Modulus), multivariable adjusted	http://www.ncbi.nlm.nih.gov/projects/gap/cgi-bin/analysis.cgi?id=pha002007
Aging	BoneAging	NSA	Hip geometry by DXA (Neck-Shaft Angle), age- and sex-adjusted	http://www.ncbi.nlm.nih.gov/projects/gap/cgi-bin/analysis.cgi?id=pha002008
Aging	BoneAging	NSA1	Hip geometry by DXA (Neck-Shaft Angle), multivariable adjusted	http://www.ncbi.nlm.nih.gov/projects/gap/cgi-bin/analysis.cgi?id=pha002009
Aging	BoneAging	ShaftCSMI1R	Hip geometry (Shaft CSMI), multivariable adjusted	http://www.ncbi.nlm.nih.gov/projects/gap/cgi-bin/analysis.cgi?id=pha002020
Aging	BoneAging	ShaftW	Hip geometry by DXA (Shaft Width), age- and sex-adjusted	http://www.ncbi.nlm.nih.gov/projects/gap/cgi-bin/analysis.cgi?id=pha002010
Aging	BoneAging	ShaftW1	Hip geometry by DXA (Shaft Width), multivariable adjusted	http://www.ncbi.nlm.nih.gov/projects/gap/cgi-bin/analysis.cgi?id=pha002011
Aging	BoneAging	ShaftZ1R	Hip geometry (Shaft Section Modulus), multivariable adjusted	http://www.ncbi.nlm.nih.gov/projects/gap/cgi-bin/analysis.cgi?id=pha002021
Aging	BoneAging	SOS	Bone Ultrasound speed measured by QUS, multivariable adjusted	http://www.ncbi.nlm.nih.gov/projects/gap/cgi-bin/analysis.cgi?id=pha002012
Aging	BoneAging	SOSof	Bone Ultrasound speed (Offspring), multivariable adjusted	http://www.ncbi.nlm.nih.gov/projects/gap/cgi-bin/analysis.cgi?id=pha002022
Aging	BoneAging	TRBMD	Trochanter BMD measured by DXA, multivariable adjusted	http://www.ncbi.nlm.nih.gov/projects/gap/cgi-bin/analysis.cgi?id=pha002013
Aging	BoneAging	TRBMDof	Troch BMD measured by DXA (Offspring), multivariable adjusted	http://www.ncbi.nlm.nih.gov/projects/gap/cgi-bin/analysis.cgi?id=pha002023
Aging	BrainMRI	AFBV	Frontal lobe to intracranial volume ratio, multivariable adjusted	http://www.ncbi.nlm.nih.gov/projects/gap/cgi-bin/analysis.cgi?id=pha002024
Aging	BrainMRI	AHPV	Hippocampal to intracranial volume ratio, multivariable adjusted	http://www.ncbi.nlm.nih.gov/projects/gap/cgi-bin/analysis.cgi?id=pha002025
Aging	BrainMRI	AHPVapoe1	Hippocampal to intracranial volume ratio, multivariable with APOE	http://www.ncbi.nlm.nih.gov/projects/gap/cgi-bin/analysis.cgi?id=pha002042
Aging	BrainMRI	AHPVr	Hippocampal to total cerebral volume ratio, multivariable adjusted	http://www.ncbi.nlm.nih.gov/projects/gap/cgi-bin/analysis.cgi?id=pha002026
Aging	BrainMRI	AHPVrapoe1	Hippocampal to total cerebral volume ratio, multivariable with APOE	http://www.ncbi.nlm.nih.gov/projects/gap/cgi-bin/analysis.cgi?id=pha002043
Aging	BrainMRI	ALLV	Log lateral ventricular to intracranial volume ratio, multivariable adjusted	http://www.ncbi.nlm.nih.gov/projects/gap/cgi-bin/analysis.cgi?id=pha002027
Aging	BrainMRI	ALWMHIVapoe1	Log white matter hyperintensity to cranial ratio, multivariable with APOE	http://www.ncbi.nlm.nih.gov/projects/gap/cgi-bin/analysis.cgi?id=pha002044
Aging	BrainMRI	AOBV	Occipital lobe to intracranial volume ratio, multivariable adjusted	http://www.ncbi.nlm.nih.gov/projects/gap/cgi-bin/analysis.cgi?id=pha002028
Aging	BrainMRI	APBV	Parietal lobe to intracranial volume ratio, multivariable adjusted	http://www.ncbi.nlm.nih.gov/projects/gap/cgi-bin/analysis.cgi?id=pha002029
Aging	BrainMRI	ATCBV	Total cerebral brain to intracranial volume ratio, multivariable adjusted	http://www.ncbi.nlm.nih.gov/projects/gap/cgi-bin/analysis.cgi?id=pha002030
Aging	BrainMRI	ATCBVapoe1	Total cerebral brain to intracranial volume ratio, multivariable with APOE	http://www.ncbi.nlm.nih.gov/projects/gap/cgi-bin/analysis.cgi?id=pha002045
Aging	BrainMRI	CFBV	Frontal lobe to intracranial volume ratio, age and sex adjusted	http://www.ncbi.nlm.nih.gov/projects/gap/cgi-bin/analysis.cgi?id=pha002031
Aging	BrainMRI	CHPV	Hippocampal to intracranial volume ratio, age and sex adjusted	http://www.ncbi.nlm.nih.gov/projects/gap/cgi-bin/analysis.cgi?id=pha002032
Aging	BrainMRI	CHPVr	Hippocampal to total cerebral volume ratio, age and sex adjusted	http://www.ncbi.nlm.nih.gov/projects/gap/cgi-bin/analysis.cgi?id=pha002033
Aging	BrainMRI	CLLV	Log lateral ventricular to intracranial volume ratio, age and sex adjusted	http://www.ncbi.nlm.nih.gov/projects/gap/cgi-bin/analysis.cgi?id=pha002034
Aging	BrainMRI	COBV	Occipital lobe to intracranial volume ratio, age and sex adjusted	http://www.ncbi.nlm.nih.gov/projects/gap/cgi-bin/analysis.cgi?id=pha002035
Aging	BrainMRI	CPBV	Parietal lobe to intracranial volume ratio, age and sex adjusted	http://www.ncbi.nlm.nih.gov/projects/gap/cgi-bin/analysis.cgi?id=pha002036
Aging	BrainMRI	CTCBV	Total cerebral brain to intracranial volume ratio, age and sex adjusted	http://www.ncbi.nlm.nih.gov/projects/gap/cgi-bin/analysis.cgi?id=pha002037
Aging	BrainMRI	WALTHBV	Winsorized temporal lobe to intracranial volume ratio, multivariable adj	http://www.ncbi.nlm.nih.gov/projects/gap/cgi-bin/analysis.cgi?id=pha002038
Aging	BrainMRI	WATBV	Winsorized log temporal horn to intracranial volume ratio, multivariable	http://www.ncbi.nlm.nih.gov/projects/gap/cgi-bin/analysis.cgi?id=pha002039
Aging	BrainMRI	WCLTHBV	Winsorized temporal lobe to intracranial volume ratio, age and sex	http://www.ncbi.nlm.nih.gov/projects/gap/cgi-bin/analysis.cgi?id=pha002040
Aging	BrainMRI	WCTBV	Winsorized log temporal horn to intracranial volume ratio, age and sex	http://www.ncbi.nlm.nih.gov/projects/gap/cgi-bin/analysis.cgi?id=pha002041
Aging	CognitiveFunction	WF1	Winsorized visual memory composite score, multivariable adjusted	http://www.ncbi.nlm.nih.gov/projects/gap/cgi-bin/analysis.cgi?id=pha002046
Aging	CognitiveFunction	WF2	Winsorized visuospatial memory & organization, multivariable adjusted	http://www.ncbi.nlm.nih.gov/projects/gap/cgi-bin/analysis.cgi?id=pha002047
Aging	CognitiveFunction	WF3	Winsorized visual scanning & motor speed, multivariable adjusted	http://www.ncbi.nlm.nih.gov/projects/gap/cgi-bin/analysis.cgi?id=pha002048
Aging	CognitiveFunction	WNam	Winsorized Boston Naming Test score without cues, multivariable adjusted	http://www.ncbi.nlm.nih.gov/projects/gap/cgi-bin/analysis.cgi?id=pha002049
Aging	CognitiveFunction	WSim	Winsorized similarities raw score, multivariable adjusted	http://www.ncbi.nlm.nih.gov/projects/gap/cgi-bin/analysis.cgi?id=pha002050
Aging	CognitiveFunction	WWRAT	Winsorized Wide Range Achievement Test	http://www.ncbi.nlm.nih.gov/projects/gap/cgi-bin/analysis.cgi?id=pha002051
Aging	Hearing	SPTA	Pure tone audiometry average over medium frequencies, age and sex adjusted	http://www.ncbi.nlm.nih.gov/projects/gap/cgi-bin/analysis.cgi?id=pha002052
Aging	Hearing	SPTAHI	Pure tone audiometry average over high frequencies, age and sex adjusted	http://www.ncbi.nlm.nih.gov/projects/gap/cgi-bin/analysis.cgi?id=pha002053
Aging	Hearing	SPTALO	Pure tone audiometry average over low frequencies, age and sex adjusted	http://www.ncbi.nlm.nih.gov/projects/gap/cgi-bin/analysis.cgi?id=pha002054

Group	Category	Trait Label	Name	Linkage Link
Aging	Hearing	WSPTA	Winsorized pure tone audiometry average over medium freq, age and sex adjusted	http://www.ncbi.nlm.nih.gov/projects/gap/cgi-bin/analysis.cgi?id=pha002055
Aging	Hearing	WSPTAHI	Winsorzed Pure tone audiometry average over high frequencies, age and sex	http://www.ncbi.nlm.nih.gov/projects/gap/cgi-bin/analysis.cgi?id=pha002056
Aging	Hearing	WSPTALO	Winsorized pure tone audiometry average over low frequencies, sex and age	http://www.ncbi.nlm.nih.gov/projects/gap/cgi-bin/analysis.cgi?id=pha002057
Aging	PhysicalDisability	Handgrip727X	hand grip, offspring exam 7 & cohort exam 27	http://www.ncbi.nlm.nih.gov/projects/gap/cgi-bin/analysis.cgi?id=pha002060
Aging	PhysicalDisability	Handgrip7X	hand grip, offspring exam 7	http://www.ncbi.nlm.nih.gov/projects/gap/cgi-bin/analysis.cgi?id=pha002061
Aging	PhysicalDisability	Walkingspeed727X	walking speed, offspring exam 7 & cohort exam 27	http://www.ncbi.nlm.nih.gov/projects/gap/cgi-bin/analysis.cgi?id=pha002062
Aging	PhysicalDisability	Walkingspeed7X	walking speed, offspring exam 7	http://www.ncbi.nlm.nih.gov/projects/gap/cgi-bin/analysis.cgi?id=pha002063
Aging	ReproductiveTraits	MenoageMVX	age at natural menopause, adjusted	http://www.ncbi.nlm.nih.gov/projects/gap/cgi-bin/analysis.cgi?id=pha002064
Aging	ReproductiveTraits	MenoageX	age at natural menopause, crude	http://www.ncbi.nlm.nih.gov/projects/gap/cgi-bin/analysis.cgi?id=pha002065
BloodPressure	Bloodpressure	DBP17AVGIMPAS	diastolic BP average age-sex-adjusted residual exams 1-7	http://www.ncbi.nlm.nih.gov/projects/gap/cgi-bin/analysis.cgi?id=pha002066
BloodPressure	Bloodpressure	DBP17AVGIMPMV	diastolic BP average multivariable-adjusted residual exams 1-7	http://www.ncbi.nlm.nih.gov/projects/gap/cgi-bin/analysis.cgi?id=pha002067
BloodPressure	Bloodpressure	DBP1IMPMEANAS	diastolic BP exam 1, age- sex- adjusted	http://www.ncbi.nlm.nih.gov/projects/gap/cgi-bin/analysis.cgi?id=pha002068
BloodPressure	Bloodpressure	DBP1IMPMEANMV	diastolic BP exam 1, multivariable-adjusted	http://www.ncbi.nlm.nih.gov/projects/gap/cgi-bin/analysis.cgi?id=pha002069
BloodPressure	Bloodpressure	DBP2IMPMEANAS	diastolic BP exam 2, age- sex- adjusted	http://www.ncbi.nlm.nih.gov/projects/gap/cgi-bin/analysis.cgi?id=pha002070
BloodPressure	Bloodpressure	DBP2IMPMEANMV	diastolic BP exam 2, multivariable-adjusted	http://www.ncbi.nlm.nih.gov/projects/gap/cgi-bin/analysis.cgi?id=pha002071
BloodPressure	Bloodpressure	DBP3IMPMEANAS	diastolic BP exam 3, age- sex- adjusted	http://www.ncbi.nlm.nih.gov/projects/gap/cgi-bin/analysis.cgi?id=pha002072
BloodPressure	Bloodpressure	DBP3IMPMEANMV	diastolic BP exam 3, multivariable-adjusted	http://www.ncbi.nlm.nih.gov/projects/gap/cgi-bin/analysis.cgi?id=pha002073
BloodPressure	Bloodpressure	DBP4IMPMEANAS	diastolic BP exam 4, age- sex- adjusted	http://www.ncbi.nlm.nih.gov/projects/gap/cgi-bin/analysis.cgi?id=pha002074
BloodPressure	Bloodpressure	DBP4IMPMEANMV	diastolic BP exam 4, multivariable-adjusted	http://www.ncbi.nlm.nih.gov/projects/gap/cgi-bin/analysis.cgi?id=pha002075
BloodPressure	Bloodpressure	DBP5IMPMEANAS	diastolic BP exam 5, age- sex- adjusted	http://www.ncbi.nlm.nih.gov/projects/gap/cgi-bin/analysis.cgi?id=pha002076
BloodPressure	Bloodpressure	DBP5IMPMEANMV	diastolic BP exam 5, multivariable-adjusted	http://www.ncbi.nlm.nih.gov/projects/gap/cgi-bin/analysis.cgi?id=pha002077
BloodPressure	Bloodpressure	DBP6IMPMEANAS	diastolic BP exam 6, age- sex- adjusted	http://www.ncbi.nlm.nih.gov/projects/gap/cgi-bin/analysis.cgi?id=pha002078
BloodPressure	Bloodpressure	DBP6IMPMEANMV	diastolic BP exam 6, multivariable-adjusted	http://www.ncbi.nlm.nih.gov/projects/gap/cgi-bin/analysis.cgi?id=pha002079
BloodPressure	Bloodpressure	DBP7IMPMEANAS	diastolic BP exam 7, age- sex- adjusted	http://www.ncbi.nlm.nih.gov/projects/gap/cgi-bin/analysis.cgi?id=pha002080
BloodPressure	Bloodpressure	DBP7IMPMEANMV	diastolic BP exam 7, multivariable-adjusted	http://www.ncbi.nlm.nih.gov/projects/gap/cgi-bin/analysis.cgi?id=pha002081
BloodPressure	Bloodpressure	PP17AVGIMPAS	pulse pressure average age-sex-adjusted residual exams 1-7	http://www.ncbi.nlm.nih.gov/projects/gap/cgi-bin/analysis.cgi?id=pha002082
BloodPressure	Bloodpressure	PP17AVGIMPMV	pulse pressure average multivariable-adjusted residual exams 1-7	http://www.ncbi.nlm.nih.gov/projects/gap/cgi-bin/analysis.cgi?id=pha002083
BloodPressure	Bloodpressure	PP1IMPMEANAS	pulse pressure exam 1, age- sex- adjusted	http://www.ncbi.nlm.nih.gov/projects/gap/cgi-bin/analysis.cgi?id=pha002084
BloodPressure	Bloodpressure	PP1IMPMEANMV	pulse pressure exam 1, multivariable- adjusted	http://www.ncbi.nlm.nih.gov/projects/gap/cgi-bin/analysis.cgi?id=pha002085
BloodPressure	Bloodpressure	PP2IMPMEANAS	pulse pressure exam 2, age- sex- adjusted	http://www.ncbi.nlm.nih.gov/projects/gap/cgi-bin/analysis.cgi?id=pha002086
BloodPressure	Bloodpressure	PP2IMPMEANMV	pulse pressure exam 2, multivariable- adjusted	http://www.ncbi.nlm.nih.gov/projects/gap/cgi-bin/analysis.cgi?id=pha002087
BloodPressure	Bloodpressure	PP3IMPMEANAS	pulse pressure exam 3, age- sex- adjusted	http://www.ncbi.nlm.nih.gov/projects/gap/cgi-bin/analysis.cgi?id=pha002088
BloodPressure	Bloodpressure	PP3IMPMEANMV	pulse pressure exam 3, multivariable- adjusted	http://www.ncbi.nlm.nih.gov/projects/gap/cgi-bin/analysis.cgi?id=pha002089
BloodPressure	Bloodpressure	PP4IMPMEANAS	pulse pressure exam 4, age- sex- adjusted	http://www.ncbi.nlm.nih.gov/projects/gap/cgi-bin/analysis.cgi?id=pha002090
BloodPressure	Bloodpressure	PP4IMPMEANMV	pulse pressure exam 4, multivariable- adjusted	http://www.ncbi.nlm.nih.gov/projects/gap/cgi-bin/analysis.cgi?id=pha002091
BloodPressure	Bloodpressure	PP5IMPMEANAS	pulse pressure exam 5, age- sex- adjusted	http://www.ncbi.nlm.nih.gov/projects/gap/cgi-bin/analysis.cgi?id=pha002092
BloodPressure	Bloodpressure	PP5IMPMEANMV	pulse pressure exam 5, multivariable- adjusted	http://www.ncbi.nlm.nih.gov/projects/gap/cgi-bin/analysis.cgi?id=pha002093
BloodPressure	Bloodpressure	PP6IMPMEANAS	pulse pressure exam 6, age- sex- adjusted	http://www.ncbi.nlm.nih.gov/projects/gap/cgi-bin/analysis.cgi?id=pha002094
BloodPressure	Bloodpressure	PP6IMPMEANMV	pulse pressure exam 6, multivariable- adjusted	http://www.ncbi.nlm.nih.gov/projects/gap/cgi-bin/analysis.cgi?id=pha002095
BloodPressure	Bloodpressure	PP7IMPMEANAS	pulse pressure exam 7, age- sex- adjusted	http://www.ncbi.nlm.nih.gov/projects/gap/cgi-bin/analysis.cgi?id=pha002096
BloodPressure	Bloodpressure	PP7IMPMEANMV	pulse pressure exam 7, multivariable- adjusted	http://www.ncbi.nlm.nih.gov/projects/gap/cgi-bin/analysis.cgi?id=pha002097
BloodPressure	Bloodpressure	SBP17AVGIMPAS	systolic BP average age-sex-adjusted residual exams 1-7	http://www.ncbi.nlm.nih.gov/projects/gap/cgi-bin/analysis.cgi?id=pha002098
BloodPressure	Bloodpressure	SBP17AVGIMPMV	systolic BP average multivariable-adjusted residual exams 1-7	http://www.ncbi.nlm.nih.gov/projects/gap/cgi-bin/analysis.cgi?id=pha002099
BloodPressure	Bloodpressure	SBP1IMPMEANAS	systolic BP exam 1, age- sex- adjusted	http://www.ncbi.nlm.nih.gov/projects/gap/cgi-bin/analysis.cgi?id=pha002100
BloodPressure	Bloodpressure	SBP1IMPMEANMV	systolic BP exam 1, multivariable-adjusted	http://www.ncbi.nlm.nih.gov/projects/gap/cgi-bin/analysis.cgi?id=pha002101
BloodPressure	Bloodpressure	SBP2IMPMEANAS	systolic BP exam 2, age- sex- adjusted	http://www.ncbi.nlm.nih.gov/projects/gap/cgi-bin/analysis.cgi?id=pha002102
BloodPressure	Bloodpressure	SBP2IMPMEANMV	systolic BP exam 2, multivariable-adjusted	http://www.ncbi.nlm.nih.gov/projects/gap/cgi-bin/analysis.cgi?id=pha002103
BloodPressure	Bloodpressure	SBP3IMPMEANAS	systolic BP exam 3, age- sex- adjusted	http://www.ncbi.nlm.nih.gov/projects/gap/cgi-bin/analysis.cgi?id=pha002104
BloodPressure	Bloodpressure	SBP3IMPMEANMV	systolic BP exam 3, multivariable-adjusted	http://www.ncbi.nlm.nih.gov/projects/gap/cgi-bin/analysis.cgi?id=pha002105
BloodPressure	Bloodpressure	SBP4IMPMEANAS	systolic BP exam 4, age- sex- adjusted	http://www.ncbi.nlm.nih.gov/projects/gap/cgi-bin/analysis.cgi?id=pha002106
BloodPressure	Bloodpressure	SBP4IMPMEANMV	systolic BP exam 4, multivariable-adjusted	http://www.ncbi.nlm.nih.gov/projects/gap/cgi-bin/analysis.cgi?id=pha002107
BloodPressure	Bloodpressure	SBP5IMPMEANAS	systolic BP exam 5, age- sex- adjusted	http://www.ncbi.nlm.nih.gov/projects/gap/cgi-bin/analysis.cgi?id=pha002108
BloodPressure	Bloodpressure	SBP5IMPMEANMV	systolic BP exam 5, multivariable-adjusted	http://www.ncbi.nlm.nih.gov/projects/gap/cgi-bin/analysis.cgi?id=pha002109
BloodPressure	Bloodpressure	SBP6IMPMEANAS	systolic BP exam 6, age- sex- adjusted	http://www.ncbi.nlm.nih.gov/projects/gap/cgi-bin/analysis.cgi?id=pha002110
BloodPressure	Bloodpressure	SBP6IMPMEANMV	systolic BP exam 6, multivariable-adjusted	http://www.ncbi.nlm.nih.gov/projects/gap/cgi-bin/analysis.cgi?id=pha002111
BloodPressure	Bloodpressure	SBP7IMPMEANAS	systolic BP exam 7, age- sex- adjusted	http://www.ncbi.nlm.nih.gov/projects/gap/cgi-bin/analysis.cgi?id=pha002112

Group	Category	Trait Label	Name	Linkage Link
BloodPressure	Bloodpressure	SBP7IMPMEANMV	systolic BP exam 7, multivariable-adjusted	http://www.ncbi.nlm.nih.gov/projects/gap/cgi-bin/analysis.cgi?id=pha002113
BloodPressure	echocardiography	AOR26AVGAS	M-mode Echo aortic root diastolic diameter, avg age-sex resid, exams 2,4,5,6	http://www.ncbi.nlm.nih.gov/projects/gap/cgi-bin/analysis.cgi?id=pha002114
BloodPressure	echocardiography	AOR26AVGMV	M-mode Echo aortic root diastolic diameter, avg multivar resid, exams 2,4,5,6	http://www.ncbi.nlm.nih.gov/projects/gap/cgi-bin/analysis.cgi?id=pha002115
BloodPressure	echocardiography	AOR2AS	M-mode Echo aortic root diastolic diameter, exam 2, age-sex-adjusted	http://www.ncbi.nlm.nih.gov/projects/gap/cgi-bin/analysis.cgi?id=pha002116
BloodPressure	echocardiography	AOR2MV	M-mode Echo aortic root diastolic diameter, exam 2, multivariable-adjusted	http://www.ncbi.nlm.nih.gov/projects/gap/cgi-bin/analysis.cgi?id=pha002117
BloodPressure	echocardiography	AOR4AS	M-mode Echo aortic root diastolic diameter, exam 4, age-sex-adjusted	http://www.ncbi.nlm.nih.gov/projects/gap/cgi-bin/analysis.cgi?id=pha002118
BloodPressure	echocardiography	AOR4MV	M-mode Echo aortic root diastolic diameter, exam 4, multivariable-adjusted	http://www.ncbi.nlm.nih.gov/projects/gap/cgi-bin/analysis.cgi?id=pha002119
BloodPressure	echocardiography	AOR5AS	M-mode Echo aortic root diastolic diameter, exam 5, age-sex-adjusted	http://www.ncbi.nlm.nih.gov/projects/gap/cgi-bin/analysis.cgi?id=pha002120
BloodPressure	echocardiography	AOR5MV	M-mode Echo aortic root diastolic diameter, exam 5, multivariable-adjusted	http://www.ncbi.nlm.nih.gov/projects/gap/cgi-bin/analysis.cgi?id=pha002121
BloodPressure	echocardiography	AOR6AS	M-mode Echo aortic root diastolic diameter, exam 6, age-sex-adjusted	http://www.ncbi.nlm.nih.gov/projects/gap/cgi-bin/analysis.cgi?id=pha002122
BloodPressure	echocardiography	AOR6MV	M-mode Echo aortic root diastolic diameter, exam 6, multivariable-adjusted	http://www.ncbi.nlm.nih.gov/projects/gap/cgi-bin/analysis.cgi?id=pha002123
BloodPressure	echocardiography	LAD26AVGAS	M-mode Echo Left atrial systolic diameter, avg age-sex resid, exams 2,4,5,6	http://www.ncbi.nlm.nih.gov/projects/gap/cgi-bin/analysis.cgi?id=pha002124
BloodPressure	echocardiography	LAD26AVGMV	M-mode Echo Left atrial systolic diameter, avg multivar resid, exams 2,4,5,6	http://www.ncbi.nlm.nih.gov/projects/gap/cgi-bin/analysis.cgi?id=pha002125
BloodPressure	echocardiography	LAD2AS	M-mode Echo Left atrial systolic diameter, exam 2, age-sex-adjusted	http://www.ncbi.nlm.nih.gov/projects/gap/cgi-bin/analysis.cgi?id=pha002126
BloodPressure	echocardiography	LAD2MV	M-mode Echo Left atrial systolic diameter, exam 2, multivariable-adjusted	http://www.ncbi.nlm.nih.gov/projects/gap/cgi-bin/analysis.cgi?id=pha002127
BloodPressure	echocardiography	LAD4AS	M-mode Echo Left atrial systolic diameter, exam 4, age-sex-adjusted	http://www.ncbi.nlm.nih.gov/projects/gap/cgi-bin/analysis.cgi?id=pha002128
BloodPressure	echocardiography	LAD4MV	M-mode Echo Left atrial systolic diameter, exam 4, multivariable-adjusted	http://www.ncbi.nlm.nih.gov/projects/gap/cgi-bin/analysis.cgi?id=pha002129
BloodPressure	echocardiography	LAD5AS	M-mode Echo Left atrial systolic diameter, exam 5, age-sex-adjusted	http://www.ncbi.nlm.nih.gov/projects/gap/cgi-bin/analysis.cgi?id=pha002130
BloodPressure	echocardiography	LAD5MV	M-mode Echo Left atrial systolic diameter, exam 5, multivariable-adjusted	http://www.ncbi.nlm.nih.gov/projects/gap/cgi-bin/analysis.cgi?id=pha002131
BloodPressure	echocardiography	LAD6AS	M-mode Echo Left atrial systolic diameter, exam 6, age-sex-adjusted	http://www.ncbi.nlm.nih.gov/projects/gap/cgi-bin/analysis.cgi?id=pha002132
BloodPressure	echocardiography	LAD6MV	M-mode Echo Left atrial systolic diameter, exam 6, multivariable-adjusted	http://www.ncbi.nlm.nih.gov/projects/gap/cgi-bin/analysis.cgi?id=pha002133
BloodPressure	echocardiography	LVDD26AVGAS	M-mode Echo LV diastolic diameter, avg age-sex resid, exams 2,4,5,6	http://www.ncbi.nlm.nih.gov/projects/gap/cgi-bin/analysis.cgi?id=pha002134
BloodPressure	echocardiography	LVDD26AVGMV	M-mode Echo LV diastolic diameter, avg multivar resid, exams 2,4,5,6	http://www.ncbi.nlm.nih.gov/projects/gap/cgi-bin/analysis.cgi?id=pha002135
BloodPressure	echocardiography	LVDD2AS	M-mode Echo LV diastolic diameter, exam 2, age-sex-adjusted	http://www.ncbi.nlm.nih.gov/projects/gap/cgi-bin/analysis.cgi?id=pha002136
BloodPressure	echocardiography	LVDD2MV	M-mode Echo LV diastolic diameter, exam 2, multivariable-adjusted	http://www.ncbi.nlm.nih.gov/projects/gap/cgi-bin/analysis.cgi?id=pha002137
BloodPressure	echocardiography	LVDD4AS	M-mode Echo LV diastolic diameter, exam 4, age-sex-adjusted	http://www.ncbi.nlm.nih.gov/projects/gap/cgi-bin/analysis.cgi?id=pha002138
BloodPressure	echocardiography	LVDD4MV	M-mode Echo LV diastolic diameter, exam 4, multivariable-adjusted	http://www.ncbi.nlm.nih.gov/projects/gap/cgi-bin/analysis.cgi?id=pha002139
BloodPressure	echocardiography	LVDD5AS	M-mode Echo LV diastolic diameter, exam 5, age-sex-adjusted	http://www.ncbi.nlm.nih.gov/projects/gap/cgi-bin/analysis.cgi?id=pha002140
BloodPressure	echocardiography	LVDD5MV	M-mode Echo LV diastolic diameter, exam 5, multivariable-adjusted	http://www.ncbi.nlm.nih.gov/projects/gap/cgi-bin/analysis.cgi?id=pha002141
BloodPressure	echocardiography	LVDD6AS	M-mode Echo LV diastolic diameter, exam 6, age-sex-adjusted	http://www.ncbi.nlm.nih.gov/projects/gap/cgi-bin/analysis.cgi?id=pha002142
BloodPressure	echocardiography	LVDD6MV	M-mode Echo LV diastolic diameter, exam 6, multivariable-adjusted	http://www.ncbi.nlm.nih.gov/projects/gap/cgi-bin/analysis.cgi?id=pha002143
BloodPressure	echocardiography	LVDS26AVGAS	M-mode Echo LV systolic diameter, avg age-sex resid, exams 2,4,5,6	http://www.ncbi.nlm.nih.gov/projects/gap/cgi-bin/analysis.cgi?id=pha002144
BloodPressure	echocardiography	LVDS26AVGMV	M-mode Echo LV systolic diameter, avg multivar resid, exams 2,4,5,6	http://www.ncbi.nlm.nih.gov/projects/gap/cgi-bin/analysis.cgi?id=pha002145
BloodPressure	echocardiography	LVDS2AS	M-mode Echo LV systolic diameter, exam 2, age-sex-adjusted	http://www.ncbi.nlm.nih.gov/projects/gap/cgi-bin/analysis.cgi?id=pha002146
BloodPressure	echocardiography	LVDS2MV	M-mode Echo LV systolic diameter, exam 2, multivariable-adjusted	http://www.ncbi.nlm.nih.gov/projects/gap/cgi-bin/analysis.cgi?id=pha002147
BloodPressure	echocardiography	LVDS4AS	M-mode Echo LV systolic diameter, exam 4, age-sex-adjusted	http://www.ncbi.nlm.nih.gov/projects/gap/cgi-bin/analysis.cgi?id=pha002148
BloodPressure	echocardiography	LVDS4MV	M-mode Echo LV systolic diameter, exam 4, multivariable-adjusted	http://www.ncbi.nlm.nih.gov/projects/gap/cgi-bin/analysis.cgi?id=pha002149
BloodPressure	echocardiography	LVDS5AS	M-mode Echo LV systolic diameter, exam 5, age-sex-adjusted	http://www.ncbi.nlm.nih.gov/projects/gap/cgi-bin/analysis.cgi?id=pha002150
BloodPressure	echocardiography	LVDS5MV	M-mode Echo LV systolic diameter, exam 5, multivariable-adjusted	http://www.ncbi.nlm.nih.gov/projects/gap/cgi-bin/analysis.cgi?id=pha002151
BloodPressure	echocardiography	LVDS6AS	M-mode Echo LV systolic diameter, exam 6, age-sex-adjusted	http://www.ncbi.nlm.nih.gov/projects/gap/cgi-bin/analysis.cgi?id=pha002152
BloodPressure	echocardiography	LVDS6MV	M-mode Echo LV systolic diameter, exam 6, multivariable-adjusted	http://www.ncbi.nlm.nih.gov/projects/gap/cgi-bin/analysis.cgi?id=pha002153
BloodPressure	echocardiography	LVFS26AVGAS	M-mode Echo LV fractional shortening, avg age-sex resid, exams 2,4,5,6	http://www.ncbi.nlm.nih.gov/projects/gap/cgi-bin/analysis.cgi?id=pha002154
BloodPressure	echocardiography	LVFS26AVGMV	M-mode Echo LV fractional shortening, avg multivar resid, exams 2,4,5,6	http://www.ncbi.nlm.nih.gov/projects/gap/cgi-bin/analysis.cgi?id=pha002155
BloodPressure	echocardiography	LVFS2AS	M-mode Echo LV fractional shortening, exam 2, age-sex-adjusted	http://www.ncbi.nlm.nih.gov/projects/gap/cgi-bin/analysis.cgi?id=pha002156
BloodPressure	echocardiography	LVFS2MV	M-mode Echo LV fractional shortening, exam 2, multivariable-adjusted	http://www.ncbi.nlm.nih.gov/projects/gap/cgi-bin/analysis.cgi?id=pha002157
BloodPressure	echocardiography	LVFS4AS	M-mode Echo LV fractional shortening, exam 4, age-sex-adjusted	http://www.ncbi.nlm.nih.gov/projects/gap/cgi-bin/analysis.cgi?id=pha002158
BloodPressure	echocardiography	LVFS4MV	M-mode Echo LV fractional shortening, exam 4, multivariable-adjusted	http://www.ncbi.nlm.nih.gov/projects/gap/cgi-bin/analysis.cgi?id=pha002159
BloodPressure	echocardiography	LVFS5AS	M-mode Echo LV fractional shortening, exam 5, age-sex-adjusted	http://www.ncbi.nlm.nih.gov/projects/gap/cgi-bin/analysis.cgi?id=pha002160
BloodPressure	echocardiography	LVFS5MV	M-mode Echo LV fractional shortening, exam 5, multivariable-adjusted	http://www.ncbi.nlm.nih.gov/projects/gap/cgi-bin/analysis.cgi?id=pha002161
BloodPressure	echocardiography	LVFS6AS	M-mode Echo LV fractional shortening, exam 6, age-sex-adjusted	http://www.ncbi.nlm.nih.gov/projects/gap/cgi-bin/analysis.cgi?id=pha002162
BloodPressure	echocardiography	LVFS6MV	M-mode Echo LV fractional shortening, exam 6, multivariable-adjusted	http://www.ncbi.nlm.nih.gov/projects/gap/cgi-bin/analysis.cgi?id=pha002163
BloodPressure	echocardiography	LVM26AVGAS	M-mode Echo LV mass, avg age-sex resid, exams 2,4,5,6	http://www.ncbi.nlm.nih.gov/projects/gap/cgi-bin/analysis.cgi?id=pha002164
BloodPressure	echocardiography	LVM26AVGMV	M-mode Echo LV mass, avg multivar resid, exams 2,4,5,6	http://www.ncbi.nlm.nih.gov/projects/gap/cgi-bin/analysis.cgi?id=pha002165
BloodPressure	echocardiography	LVM2AS	M-mode Echo LV mass, exam 2, age-sex-adjusted	http://www.ncbi.nlm.nih.gov/projects/gap/cgi-bin/analysis.cgi?id=pha002166
BloodPressure	echocardiography	LVM2MV	M-mode Echo LV mass, exam 2, multivariable-adjusted	http://www.ncbi.nlm.nih.gov/projects/gap/cgi-bin/analysis.cgi?id=pha002167
BloodPressure	echocardiography	LVM4AS	M-mode Echo LV mass, exam 4, age-sex-adjusted	http://www.ncbi.nlm.nih.gov/projects/gap/cgi-bin/analysis.cgi?id=pha002168

Group	Category	Trait Label	Name	Linkage Link
BloodPressure	echocardiography	LVM4MV	M-mode Echo LV mass, exam 4, multivariable-adjusted	http://www.ncbi.nlm.nih.gov/projects/gap/cgi-bin/analysis.cgi?id=pha002169
BloodPressure	echocardiography	LVM5AS	M-mode Echo LV mass, exam 5, age-sex-adjusted	http://www.ncbi.nlm.nih.gov/projects/gap/cgi-bin/analysis.cgi?id=pha002170
BloodPressure	echocardiography	LVM5MV	M-mode Echo LV mass, exam 5, multivariable-adjusted	http://www.ncbi.nlm.nih.gov/projects/gap/cgi-bin/analysis.cgi?id=pha002171
BloodPressure	echocardiography	LVM6AS	M-mode Echo LV mass, exam 6, age-sex-adjusted	http://www.ncbi.nlm.nih.gov/projects/gap/cgi-bin/analysis.cgi?id=pha002172
BloodPressure	echocardiography	LVM6MV	M-mode Echo LV mass, exam 6, multivariable-adjusted	http://www.ncbi.nlm.nih.gov/projects/gap/cgi-bin/analysis.cgi?id=pha002173
BloodPressure	echocardiography	LVWT26AVGAS	M-mode Echo LV diastolic wall thickness, avg age-sex resid, exams 2,4,5,6	http://www.ncbi.nlm.nih.gov/projects/gap/cgi-bin/analysis.cgi?id=pha002174
BloodPressure	echocardiography	LVWT26AVGMV	M-mode Echo LV diastolic wall thickness, avg multivar resid, exams 2,4,5,6	http://www.ncbi.nlm.nih.gov/projects/gap/cgi-bin/analysis.cgi?id=pha002175
BloodPressure	echocardiography	LVWT2AS	M-mode Echo LV diastolic wall thickness, exam 2, age-sex-adjusted	http://www.ncbi.nlm.nih.gov/projects/gap/cgi-bin/analysis.cgi?id=pha002176
BloodPressure	echocardiography	LVWT2MV	M-mode Echo LV diastolic wall thickness, exam 2, multivariable-adjusted	http://www.ncbi.nlm.nih.gov/projects/gap/cgi-bin/analysis.cgi?id=pha002177
BloodPressure	echocardiography	LVWT4AS	M-mode Echo LV diastolic wall thickness, exam 4, age-sex-adjusted	http://www.ncbi.nlm.nih.gov/projects/gap/cgi-bin/analysis.cgi?id=pha002178
BloodPressure	echocardiography	LVWT4MV	M-mode Echo LV diastolic wall thickness, exam 4, multivariable-adjusted	http://www.ncbi.nlm.nih.gov/projects/gap/cgi-bin/analysis.cgi?id=pha002179
BloodPressure	echocardiography	LVWT5AS	M-mode Echo LV diastolic wall thickness, exam 5, age-sex-adjusted	http://www.ncbi.nlm.nih.gov/projects/gap/cgi-bin/analysis.cgi?id=pha002180
BloodPressure	echocardiography	LVWT5MV	M-mode Echo LV diastolic wall thickness, exam 5, multivariable-adjusted	http://www.ncbi.nlm.nih.gov/projects/gap/cgi-bin/analysis.cgi?id=pha002181
BloodPressure	echocardiography	LVWT6AS	M-mode Echo LV diastolic wall thickness, exam 6, age-sex-adjusted	http://www.ncbi.nlm.nih.gov/projects/gap/cgi-bin/analysis.cgi?id=pha002182
BloodPressure	echocardiography	LVWT6MV	M-mode Echo LV diastolic wall thickness, exam 6, multivariable-adjusted	http://www.ncbi.nlm.nih.gov/projects/gap/cgi-bin/analysis.cgi?id=pha002183
BloodPressure	Endothelialfunction	BASEFLOW7AS	Brachial artery Baseline flow velocity, exam 7, age-sex-adjusted	http://www.ncbi.nlm.nih.gov/projects/gap/cgi-bin/analysis.cgi?id=pha002184
BloodPressure	Endothelialfunction	BASEFLOW7MV	Brachial artery Baseline flow velocity, exam 7, multivariable-adjusted	http://www.ncbi.nlm.nih.gov/projects/gap/cgi-bin/analysis.cgi?id=pha002185
BloodPressure	Endothelialfunction	BASELINEDIAMETERAS7	baseline brachial artery diameter, exam 7, age and sex-adjusted	http://www.ncbi.nlm.nih.gov/projects/gap/cgi-bin/analysis.cgi?id=pha002186
BloodPressure	Endothelialfunction	BASELINEDIAMETERMV7	baseline brachial artery diameter, exam 7, multivariable-adjusted	http://www.ncbi.nlm.nih.gov/projects/gap/cgi-bin/analysis.cgi?id=pha002187
BloodPressure	Endothelialfunction	FMD7PCTAS	Brachial artery Flow mediated dilation, exam 7, age-adjusted	http://www.ncbi.nlm.nih.gov/projects/gap/cgi-bin/analysis.cgi?id=pha002188
BloodPressure	Endothelialfunction	FMD7PCTMV	Brachial artery Flow mediated dilation, exam 7, multivariable-adjusted	http://www.ncbi.nlm.nih.gov/projects/gap/cgi-bin/analysis.cgi?id=pha002189
BloodPressure	Endothelialfunction	HYPERFLOW7AS	Brachial artery hyperemic flow velocity, exam 7, age-sex-adjusted	http://www.ncbi.nlm.nih.gov/projects/gap/cgi-bin/analysis.cgi?id=pha002190
BloodPressure	Endothelialfunction	HYPERFLOW7MV	Brachial artery hyperemic flow velocity, exam 7, multivariable-adjusted	http://www.ncbi.nlm.nih.gov/projects/gap/cgi-bin/analysis.cgi?id=pha002191
BloodPressure	ExerciseTest	ETT2DBPREC3AS	Exercise recovery 3-min diastolic BP, exam 2, age-sex-adjusted	http://www.ncbi.nlm.nih.gov/projects/gap/cgi-bin/analysis.cgi?id=pha002192
BloodPressure	ExerciseTest	ETT2DBPREC3MV	Exercise recovery 3-min diastolic BP, exam 2, multivariable-adjusted	http://www.ncbi.nlm.nih.gov/projects/gap/cgi-bin/analysis.cgi?id=pha002193
BloodPressure	ExerciseTest	ETT2DBPSTG2AS	Exercise Stage 2 diastolic BP, exam 2, age-sex-adjusted	http://www.ncbi.nlm.nih.gov/projects/gap/cgi-bin/analysis.cgi?id=pha002194
BloodPressure	ExerciseTest	ETT2DBPSTG2MV	Exercise Stage 2 diastolic BP, exam 2, multivariable-adjusted	http://www.ncbi.nlm.nih.gov/projects/gap/cgi-bin/analysis.cgi?id=pha002195
BloodPressure	ExerciseTest	ETT2HRREC3AS	Exercise recovery 3-min heart rate, exam 2, age-sex-adjusted	http://www.ncbi.nlm.nih.gov/projects/gap/cgi-bin/analysis.cgi?id=pha002196
BloodPressure	ExerciseTest	ETT2HRREC3MV	Exercise recovery 3-min heart rate, exam 2, multivariable-adjusted	http://www.ncbi.nlm.nih.gov/projects/gap/cgi-bin/analysis.cgi?id=pha002197
BloodPressure	ExerciseTest	ETT2HRSTG2AS	Exercise Stage 2 heart rate, exam 2, age-sex-adjusted	http://www.ncbi.nlm.nih.gov/projects/gap/cgi-bin/analysis.cgi?id=pha002198
BloodPressure	ExerciseTest	ETT2HRSTG2MV	Exercise Stage 2 heart rate, exam 2, multivariable-adjusted	http://www.ncbi.nlm.nih.gov/projects/gap/cgi-bin/analysis.cgi?id=pha002199
BloodPressure	ExerciseTest	ETT2SBPREC3AS	Exercise recovery 3-min systolic BP, exam 2, age-sex-adjusted	http://www.ncbi.nlm.nih.gov/projects/gap/cgi-bin/analysis.cgi?id=pha002200
BloodPressure	ExerciseTest	ETT2SBPREC3MV	Exercise recovery 3-min systolic BP, exam 2, multivariable-adjusted	http://www.ncbi.nlm.nih.gov/projects/gap/cgi-bin/analysis.cgi?id=pha002201
BloodPressure	ExerciseTest	ETT2SBPSTG2AS	Exercise Stage 2 systolic BP, exam 2, age-sex-adjusted	http://www.ncbi.nlm.nih.gov/projects/gap/cgi-bin/analysis.cgi?id=pha002202
BloodPressure	ExerciseTest	ETT2SBPSTG2MV	Exercise Stage 2 systolic BP, exam 2, multivariable-adjusted	http://www.ncbi.nlm.nih.gov/projects/gap/cgi-bin/analysis.cgi?id=pha002203
BloodPressure	tonometry	AI7AS	augmentation index, exam 7, age-sex-adjusted	http://www.ncbi.nlm.nih.gov/projects/gap/cgi-bin/analysis.cgi?id=pha002204
BloodPressure	tonometry	AI7MV	augmentation index, exam 7, multivariable-adjusted	http://www.ncbi.nlm.nih.gov/projects/gap/cgi-bin/analysis.cgi?id=pha002205
BloodPressure	tonometry	CBPWV7AS	carotid-brachial pulse wave velocity, exam 7, age-sex-adjusted	http://www.ncbi.nlm.nih.gov/projects/gap/cgi-bin/analysis.cgi?id=pha002206
BloodPressure	tonometry	CBPWV7MV	carotid-brachial pulse wave velocity, exam 7, multivariable-adjusted	http://www.ncbi.nlm.nih.gov/projects/gap/cgi-bin/analysis.cgi?id=pha002207
BloodPressure	tonometry	CFPWV7AS	carotid-femoral pulse wave velocity, exam 7, age-sex-adjusted	http://www.ncbi.nlm.nih.gov/projects/gap/cgi-bin/analysis.cgi?id=pha002208
BloodPressure	tonometry	CFPWV7MV	carotid-femoral pulse wave velocity, exam 7, multivariable-adjusted	http://www.ncbi.nlm.nih.gov/projects/gap/cgi-bin/analysis.cgi?id=pha002209
BloodPressure	tonometry	CPP7AS	central pulse pressure, exam 7, age-sex-adjusted	http://www.ncbi.nlm.nih.gov/projects/gap/cgi-bin/analysis.cgi?id=pha002210
BloodPressure	tonometry	CPP7MV	central pulse pressure, exam 7, multivariable-adjusted	http://www.ncbi.nlm.nih.gov/projects/gap/cgi-bin/analysis.cgi?id=pha002211
BloodPressure	tonometry	CRPWV7MV	carotid-radial pulse wave velocity, exam 7, multivariable-adjusted	http://www.ncbi.nlm.nih.gov/projects/gap/cgi-bin/analysis.cgi?id=pha002212
BloodPressure	tonometry	DBP7BRAOSCAS	Diastolic BP Brachial oscillometric, exam 7, age-sex-adjusted	http://www.ncbi.nlm.nih.gov/projects/gap/cgi-bin/analysis.cgi?id=pha002213
BloodPressure	tonometry	DBP7BRAOSCMV	Diastolic BP Brachial oscillometric, exam 7, multivariable-adjusted	http://www.ncbi.nlm.nih.gov/projects/gap/cgi-bin/analysis.cgi?id=pha002214
BloodPressure	tonometry	FWDWAVE7AS	forward wave amplitude, exam 7, age-sex-adjusted	http://www.ncbi.nlm.nih.gov/projects/gap/cgi-bin/analysis.cgi?id=pha002215
BloodPressure	tonometry	FWDWAVE7MV	forward wave amplitude, exam 7, multivariable-adjusted	http://www.ncbi.nlm.nih.gov/projects/gap/cgi-bin/analysis.cgi?id=pha002216
BloodPressure	tonometry	INVCFPWV7AS	LV ejection time, exam 7, age-sex-adjusted	http://www.ncbi.nlm.nih.gov/projects/gap/cgi-bin/analysis.cgi?id=pha002217
BloodPressure	tonometry	INVCFPWV7MV	LV ejection time, exam 7, multivariable-adjusted	http://www.ncbi.nlm.nih.gov/projects/gap/cgi-bin/analysis.cgi?id=pha002218
BloodPressure	tonometry	MAP7AS	mean arterial pressure, exam 7, age-sex-adjusted	http://www.ncbi.nlm.nih.gov/projects/gap/cgi-bin/analysis.cgi?id=pha002219
BloodPressure	tonometry	MAP7MV	mean arterial pressure, exam 7, multivariable-adjusted	http://www.ncbi.nlm.nih.gov/projects/gap/cgi-bin/analysis.cgi?id=pha002220
BloodPressure	tonometry	PERAMPAP7AS	Peripheral amplification apparent, exam 7, age-sex-adjusted	http://www.ncbi.nlm.nih.gov/projects/gap/cgi-bin/analysis.cgi?id=pha002221
BloodPressure	tonometry	PERAMPAP7MV	Peripheral amplification apparent, exam 7, multivariable-adjusted	http://www.ncbi.nlm.nih.gov/projects/gap/cgi-bin/analysis.cgi?id=pha002222
BloodPressure	tonometry	PERAMPTRU7AS	Peripheral amplification true, exam 7, age-sex-adjusted	http://www.ncbi.nlm.nih.gov/projects/gap/cgi-bin/analysis.cgi?id=pha002223
BloodPressure	tonometry	PERAMPTRU7MV	Peripheral amplification true, exam 7, multivariable-adjusted	http://www.ncbi.nlm.nih.gov/projects/gap/cgi-bin/analysis.cgi?id=pha002224

Group	Category	Trait Label	Name	Linkage Link
BloodPressure	tonometry	PP7BRAOSCAS	Pulse pressure Brachial oscillometric, exam 7, age-sex-adjusted	http://www.ncbi.nlm.nih.gov/projects/gap/cgi-bin/analysis.cgi?id=pha002225
BloodPressure	tonometry	PP7BRAOSCMV	Pulse pressure Brachial oscillometric, exam 7, multivariable-adjusted	http://www.ncbi.nlm.nih.gov/projects/gap/cgi-bin/analysis.cgi?id=pha002226
BloodPressure	tonometry	REFWAVE7AS	reflected wave amplitude, exam 7, age-sex-adjusted	http://www.ncbi.nlm.nih.gov/projects/gap/cgi-bin/analysis.cgi?id=pha002227
BloodPressure	tonometry	REFWAVE7MV	reflected wave amplitude, exam 7, multivariable-adjusted	http://www.ncbi.nlm.nih.gov/projects/gap/cgi-bin/analysis.cgi?id=pha002228
BloodPressure	tonometry	RWTT7AS	reflected wave transit time, exam 7, age-sex-adjusted	http://www.ncbi.nlm.nih.gov/projects/gap/cgi-bin/analysis.cgi?id=pha002229
BloodPressure	tonometry	RWTT7MV	reflected wave transit time, exam 7, multivariable-adjusted	http://www.ncbi.nlm.nih.gov/projects/gap/cgi-bin/analysis.cgi?id=pha002230
BloodPressure	tonometry	SBP7BRAOSCAS	Systolic BP Brachial oscillometric, exam 7, age-sex-adjusted	http://www.ncbi.nlm.nih.gov/projects/gap/cgi-bin/analysis.cgi?id=pha002231
BloodPressure	tonometry	SBP7BRAOSCMV	Systolic BP Brachial oscillometric, exam 7, multivariable-adjusted	http://www.ncbi.nlm.nih.gov/projects/gap/cgi-bin/analysis.cgi?id=pha002232
CVDMiscTraits	ECGtraits	PRAdjRRPOOL	Age & RR adjusted PR interval cohort ex 11 offspring ex 1	http://www.ncbi.nlm.nih.gov/projects/gap/cgi-bin/analysis.cgi?id=pha002701
CVDMiscTraits	ECGtraits	QTMEN	Age & RR adjusted QT interval men cohort ex 11 offspring ex 1	http://www.ncbi.nlm.nih.gov/projects/gap/cgi-bin/analysis.cgi?id=pha002702
CVDMiscTraits	ECGtraits	QTPOOL	Age & RR adjusted QT interval men & women cohort ex 11 offspring ex 1	http://www.ncbi.nlm.nih.gov/projects/gap/cgi-bin/analysis.cgi?id=pha002703
CVDMiscTraits	ECGtraits	QTWOMEN	Age & RR adjusted QT interval women cohort ex 11 offspring ex 1	http://www.ncbi.nlm.nih.gov/projects/gap/cgi-bin/analysis.cgi?id=pha002704
CVDMiscTraits	ECGtraits	RRMEN	Age adjusted RR interval men cohort ex 11 offspring ex 1	http://www.ncbi.nlm.nih.gov/projects/gap/cgi-bin/analysis.cgi?id=pha002705
CVDMiscTraits	ECGtraits	RRPOOL	Age adjusted RR interval men & women cohort ex 11 offspring ex 1	http://www.ncbi.nlm.nih.gov/projects/gap/cgi-bin/analysis.cgi?id=pha002706
CVDMiscTraits	ECGtraits	RRWOMEN	Age adjusted RR interval women cohort ex 11 offspring ex 1	http://www.ncbi.nlm.nih.gov/projects/gap/cgi-bin/analysis.cgi?id=pha002707
CVDMiscTraits	HRVtraits	LFHFHRV	Age & HR adjusted low:high freq power HRV exams 18 (cohort), 3 (offspring)	http://www.ncbi.nlm.nih.gov/projects/gap/cgi-bin/analysis.cgi?id=pha002708
CVDMiscTraits	HRVtraits	SDNNHRV	Age & HR adjsuted SDNN HRV exams 18 (cohort), 3 (offspring)	http://www.ncbi.nlm.nih.gov/projects/gap/cgi-bin/analysis.cgi?id=pha002709
CVDMiscTraits	HRVtraits	TOTPWRHRV	Age & HR adjsuted total power HRV exams 18 (cohort), 3 (offspring)	http://www.ncbi.nlm.nih.gov/projects/gap/cgi-bin/analysis.cgi?id=pha002710
CVDMiscTraits	Otherclinicaltraits	ALCOHOL1	Age- & sex-adjusted drinks/day ex 1	http://www.ncbi.nlm.nih.gov/projects/gap/cgi-bin/analysis.cgi?id=pha002711
CVDMiscTraits	Otherclinicaltraits	ALCOHOL2	Age- & sex-adjusted drinks/day across all exams	http://www.ncbi.nlm.nih.gov/projects/gap/cgi-bin/analysis.cgi?id=pha002712
CVDMiscTraits	Otherclinicaltraits	ALCOHOL3	Age- & sex-adjusted gallbladder disease	http://www.ncbi.nlm.nih.gov/projects/gap/cgi-bin/analysis.cgi?id=pha002713
ITBiomarkers	Hematological	Hctavg12asWIN	Hematocrit, average exam 1 & 2, age & sex, Winsorized	http://www.ncbi.nlm.nih.gov/projects/gap/cgi-bin/analysis.cgi?id=pha002233
ITBiomarkers	Hematological	Hctavg12mvWIN	Hematocrit, averaged residuals of exam 1 & 2, multivariable, Winsorized	http://www.ncbi.nlm.nih.gov/projects/gap/cgi-bin/analysis.cgi?id=pha002234
ITBiomarkers	Hematological	Hctex1asWIN	Hematocrit exam 1 age & sex, Winsorized	http://www.ncbi.nlm.nih.gov/projects/gap/cgi-bin/analysis.cgi?id=pha002235
ITBiomarkers	Hematological	Hctex1mvWIN	Hematocrit exam 1 multivariable, Winsorized	http://www.ncbi.nlm.nih.gov/projects/gap/cgi-bin/analysis.cgi?id=pha002236
ITBiomarkers	Hematological	Hctex2asWIN	Hematocrit exam 2 age & sex, Winsorized	http://www.ncbi.nlm.nih.gov/projects/gap/cgi-bin/analysis.cgi?id=pha002237
ITBiomarkers	Hematological	Hctex2mvWIN	Hematocrit exam 2 multivariable, Winsorized	http://www.ncbi.nlm.nih.gov/projects/gap/cgi-bin/analysis.cgi?id=pha002238
ITBiomarkers	Hematological	Hgbavg12asWIN	Hemoglobin averaged residuals of exam 1 & 2, age & sex, Winsorized	http://www.ncbi.nlm.nih.gov/projects/gap/cgi-bin/analysis.cgi?id=pha002239
ITBiomarkers	Hematological	Hgbavg12mvWIN	Hemoglobin, averaged residuals of exam 1 & 2, multivariable, Winsorized	http://www.ncbi.nlm.nih.gov/projects/gap/cgi-bin/analysis.cgi?id=pha002240
ITBiomarkers	Hematological	Hgbex1asWIN	Hemoglobin exam 1 age & sex, Winsorized	http://www.ncbi.nlm.nih.gov/projects/gap/cgi-bin/analysis.cgi?id=pha002241
ITBiomarkers	Hematological	Hgbex1mvWIN	Hemoglobin exam 1 multivariable, Winsorized	http://www.ncbi.nlm.nih.gov/projects/gap/cgi-bin/analysis.cgi?id=pha002242
ITBiomarkers	Hematological	Hgbex2asWIN	Hemoglobin exam 2 age & sex, Winsorized	http://www.ncbi.nlm.nih.gov/projects/gap/cgi-bin/analysis.cgi?id=pha002243
ITBiomarkers	Hematological	Hgbex2mvWIN	Hemoglobin exam 2 multivariable, Winsorized	http://www.ncbi.nlm.nih.gov/projects/gap/cgi-bin/analysis.cgi?id=pha002244
ITBiomarkers	Hematological	MCHCavg12as	Mean corpuscular hemoglobin concentration avg. residuals exam 1&2 age & sex	http://www.ncbi.nlm.nih.gov/projects/gap/cgi-bin/analysis.cgi?id=pha002245
ITBiomarkers	Hematological	MCHCavg12mv	Mean corpuscular hemoglobin concentration avg. residuals of exam 1&2 MV	http://www.ncbi.nlm.nih.gov/projects/gap/cgi-bin/analysis.cgi?id=pha002246
ITBiomarkers	Hematological	MCHCex1asWIN	Mean corpuscular hemoglobin concentration exam 1 age & sex, Winsorized	http://www.ncbi.nlm.nih.gov/projects/gap/cgi-bin/analysis.cgi?id=pha002247
ITBiomarkers	Hematological	MCHCex1mvWIN	Mean corpuscular hemoglobin concentration exam 1 multivariable, Winsorized	http://www.ncbi.nlm.nih.gov/projects/gap/cgi-bin/analysis.cgi?id=pha002248
ITBiomarkers	Hematological	MCHCex2as	Mean corpuscular hemoglobin concentration exam 2 age & sex	http://www.ncbi.nlm.nih.gov/projects/gap/cgi-bin/analysis.cgi?id=pha002249
ITBiomarkers	Hematological	MCHCex2mv	Mean corpuscular hemoglobin concentration exam 2 multivariable	http://www.ncbi.nlm.nih.gov/projects/gap/cgi-bin/analysis.cgi?id=pha002250
ITBiomarkers	Hematological	MCVavg12asNL	Mean corpuscular volume avg. residuals exam 1&2, age & sex, normalized dev.	http://www.ncbi.nlm.nih.gov/projects/gap/cgi-bin/analysis.cgi?id=pha002251
ITBiomarkers	Hematological	MCVavg12mvNL	Mean corpuscular volume avg. residuals exam 1&2, multiv., normalized dev.	http://www.ncbi.nlm.nih.gov/projects/gap/cgi-bin/analysis.cgi?id=pha002252
ITBiomarkers	Hematological	MCVex1asNL	Mean corpuscular volume exam 1 age & sex, normalized deviates	http://www.ncbi.nlm.nih.gov/projects/gap/cgi-bin/analysis.cgi?id=pha002253
ITBiomarkers	Hematological	MCVex1mvNL	Mean corpuscular volume multivariable, normalized deviates	http://www.ncbi.nlm.nih.gov/projects/gap/cgi-bin/analysis.cgi?id=pha002254
ITBiomarkers	Hematological	MCVex2asNL	Mean corpuscular volume exam 2 age & sex, normalized deviates	http://www.ncbi.nlm.nih.gov/projects/gap/cgi-bin/analysis.cgi?id=pha002255
ITBiomarkers	Hematological	MCVex2mvNL	Mean corpuscular volume multivariable exam 2, normalized deviates	http://www.ncbi.nlm.nih.gov/projects/gap/cgi-bin/analysis.cgi?id=pha002256
ITBiomarkers	Hematological	RBCCavg12asWIN	Red blood cell count averaged residuals exam 1 & 2, age & sex, Winsorized	http://www.ncbi.nlm.nih.gov/projects/gap/cgi-bin/analysis.cgi?id=pha002257
ITBiomarkers	Hematological	RBCCavg12mvWIN	Red blood cell count avg. residuals exam 1 & 2, multivariable, Winsorized	http://www.ncbi.nlm.nih.gov/projects/gap/cgi-bin/analysis.cgi?id=pha002258
ITBiomarkers	Hematological	RBCCex1asWIN	Red blood cell count exam 1 age & sex, Winsorized	http://www.ncbi.nlm.nih.gov/projects/gap/cgi-bin/analysis.cgi?id=pha002259
ITBiomarkers	Hematological	RBCCex1mvWIN	Red blood cell count exam 1 multivariable, Winsorized	http://www.ncbi.nlm.nih.gov/projects/gap/cgi-bin/analysis.cgi?id=pha002260
ITBiomarkers	Hematological	RBCCex2as	Red blood cell count exam 2 age & sex	http://www.ncbi.nlm.nih.gov/projects/gap/cgi-bin/analysis.cgi?id=pha002261
ITBiomarkers	Hematological	RBCCex2asWIN	Red blood cell count exam 2 age & sex, Winsorized	http://www.ncbi.nlm.nih.gov/projects/gap/cgi-bin/analysis.cgi?id=pha002262
ITBiomarkers	Hematological	RBCCex2mv	Red blood cell count exam 2 multivariable	http://www.ncbi.nlm.nih.gov/projects/gap/cgi-bin/analysis.cgi?id=pha002263
ITBiomarkers	Hematological	RBCCex2mvWIN	Red blood cell count exam 2 multivariable, Winsorized	http://www.ncbi.nlm.nih.gov/projects/gap/cgi-bin/analysis.cgi?id=pha002264
ITBiomarkers	Hematological	WBCavg12as	White blood cell count averaged residuals of exam 1 & 2, age & sex	http://www.ncbi.nlm.nih.gov/projects/gap/cgi-bin/analysis.cgi?id=pha002265
ITBiomarkers	Hematological	WBCavg12mv	White blood cell count, averaged residuals of exam 1 & 2, multivariable	http://www.ncbi.nlm.nih.gov/projects/gap/cgi-bin/analysis.cgi?id=pha002266
ITBiomarkers	Hematological	WBCex1as	White blood cell count exam 1 age & sex, log transformed	http://www.ncbi.nlm.nih.gov/projects/gap/cgi-bin/analysis.cgi?id=pha002267

Group	Category	Trait Label	Name	Linkage Link
ITBiomarkers	Hematological	WBCex1mv	White blood cell count exam 1 multivariable, log transformed	http://www.ncbi.nlm.nih.gov/projects/gap/cgi-bin/analysis.cgi?id=pha002268
ITBiomarkers	Hematological	WBCex2as	White blood cell count exam 2 age & sex	http://www.ncbi.nlm.nih.gov/projects/gap/cgi-bin/analysis.cgi?id=pha002269
ITBiomarkers	Hematological	WBCex2mv	White blood cell count exam 2 multivariable	http://www.ncbi.nlm.nih.gov/projects/gap/cgi-bin/analysis.cgi?id=pha002270
ITBiomarkers	Hemostatic	dDimerex5as	D dimer at exam 6 age & sex, log transformed	http://www.ncbi.nlm.nih.gov/projects/gap/cgi-bin/analysis.cgi?id=pha002271
ITBiomarkers	Hemostatic	dDimerex5mv	D dimer at exam 6 multivariable, log transformed	http://www.ncbi.nlm.nih.gov/projects/gap/cgi-bin/analysis.cgi?id=pha002272
ITBiomarkers	Hemostatic	FibrinogenAvg567as	Fibrinogen averaged residuals of exam 5, 6 & 7 age & sex, log transformed	http://www.ncbi.nlm.nih.gov/projects/gap/cgi-bin/analysis.cgi?id=pha002273
ITBiomarkers	Hemostatic	FibrinogenAvg567mv	Fibrinogen averaged residuals exam 5, 6 & 7 multivariable, log transformed	http://www.ncbi.nlm.nih.gov/projects/gap/cgi-bin/analysis.cgi?id=pha002274
ITBiomarkers	Hemostatic	Fibrinogenex5as	Fibrinogen at exam 5 age & sex, log transformed	http://www.ncbi.nlm.nih.gov/projects/gap/cgi-bin/analysis.cgi?id=pha002275
ITBiomarkers	Hemostatic	Fibrinogenex5mv	Fibrinogen at exam 5 multivariable, log transformed	http://www.ncbi.nlm.nih.gov/projects/gap/cgi-bin/analysis.cgi?id=pha002276
ITBiomarkers	Hemostatic	Fibrinogenex6as	Fibrinogen at exam 6 age & sex, log transformed	http://www.ncbi.nlm.nih.gov/projects/gap/cgi-bin/analysis.cgi?id=pha002277
ITBiomarkers	Hemostatic	Fibrinogenex6mv	Fibrinogen at exam 6 multivariable, log transformed	http://www.ncbi.nlm.nih.gov/projects/gap/cgi-bin/analysis.cgi?id=pha002278
ITBiomarkers	Hemostatic	Fibrinogenex7as	Fibrinogen at exam 7 age & sex, log transformed	http://www.ncbi.nlm.nih.gov/projects/gap/cgi-bin/analysis.cgi?id=pha002279
ITBiomarkers	Hemostatic	Fibrinogenex7mv	Fibrinogen at exam 7 multivariable, log transformed	http://www.ncbi.nlm.nih.gov/projects/gap/cgi-bin/analysis.cgi?id=pha002280
ITBiomarkers	Hemostatic	FVIIex5as	Factor VII at exam 5 age & sex	http://www.ncbi.nlm.nih.gov/projects/gap/cgi-bin/analysis.cgi?id=pha002281
ITBiomarkers	Hemostatic	FVIIex5mv	Factor VII at exam 5 multivariable	http://www.ncbi.nlm.nih.gov/projects/gap/cgi-bin/analysis.cgi?id=pha002282
ITBiomarkers	Hemostatic	PAI1ex56Avgas	Log plasminogen Activator 1 avg. residuals exam 5& 6 age & sex	http://www.ncbi.nlm.nih.gov/projects/gap/cgi-bin/analysis.cgi?id=pha002283
ITBiomarkers	Hemostatic	PAI1ex56Avgmv	Log plasminogen Activator 1 avg. residuals exam 5 & 6 multivariable	http://www.ncbi.nlm.nih.gov/projects/gap/cgi-bin/analysis.cgi?id=pha002284
ITBiomarkers	Hemostatic	PAI1ex5as	Plasminogen Activator 1 at exam 5 age & sex, log transformed	http://www.ncbi.nlm.nih.gov/projects/gap/cgi-bin/analysis.cgi?id=pha002285
ITBiomarkers	Hemostatic	PAI1ex5mv	Plasminogen Activator 1 at exam 5 multivariable, log transformed	http://www.ncbi.nlm.nih.gov/projects/gap/cgi-bin/analysis.cgi?id=pha002286
ITBiomarkers	Hemostatic	PAI1ex6as	Plasminogen Activator 1 at exam 6 age & sex, log transformed	http://www.ncbi.nlm.nih.gov/projects/gap/cgi-bin/analysis.cgi?id=pha002287
ITBiomarkers	Hemostatic	PAI1ex6mv	Plasminogen Activator 1 at exam 6 multivariable, log transformed	http://www.ncbi.nlm.nih.gov/projects/gap/cgi-bin/analysis.cgi?id=pha002288
ITBiomarkers	Hemostatic	PlateletAgADPex5asNL	Log ADP platelet aggregation exam 5, age & sex, residuals normalized dev.	http://www.ncbi.nlm.nih.gov/projects/gap/cgi-bin/analysis.cgi?id=pha002289
ITBiomarkers	Hemostatic	PlateletAgADPex5mvNL	Log ADP platelet aggregation exam 5, multivariable residuals normalized dev	http://www.ncbi.nlm.nih.gov/projects/gap/cgi-bin/analysis.cgi?id=pha002290
ITBiomarkers	Hemostatic	PlateletAgCollex5as	Platelet Aggregation to collagen, exam 5, age & sex, log transformed	http://www.ncbi.nlm.nih.gov/projects/gap/cgi-bin/analysis.cgi?id=pha002291
ITBiomarkers	Hemostatic	PlateletAgCollex5mv	Platelet Aggregation to collagen, exam 5, multivariable, log transformed	http://www.ncbi.nlm.nih.gov/projects/gap/cgi-bin/analysis.cgi?id=pha002292
ITBiomarkers	Hemostatic	PlateletAgEpiex5as	Platelet Aggregation to Epinephrine at exam 5 age & sex, log transformed	http://www.ncbi.nlm.nih.gov/projects/gap/cgi-bin/analysis.cgi?id=pha002293
ITBiomarkers	Hemostatic	PlateletAgEpiex5mv	Platelet Aggregation to Epinephrine exam 5 multivariable, log transformed	http://www.ncbi.nlm.nih.gov/projects/gap/cgi-bin/analysis.cgi?id=pha002294
ITBiomarkers	Hemostatic	tPAex5as	tPA Antigen at exam 5 age & sex, log transformed	http://www.ncbi.nlm.nih.gov/projects/gap/cgi-bin/analysis.cgi?id=pha002295
ITBiomarkers	Hemostatic	tPAex5mvWIN	tPA Antigen exam 5 multivariable, log transformed and residuals winsorized	http://www.ncbi.nlm.nih.gov/projects/gap/cgi-bin/analysis.cgi?id=pha002296
ITBiomarkers	Hemostatic	Viscoscityex5as	Viscosity I at exam 5 age & sex	http://www.ncbi.nlm.nih.gov/projects/gap/cgi-bin/analysis.cgi?id=pha002297
ITBiomarkers	Hemostatic	Viscoscityex5mv	Viscosity I at exam 5 multivariable	http://www.ncbi.nlm.nih.gov/projects/gap/cgi-bin/analysis.cgi?id=pha002298
ITBiomarkers	Hemostatic	wVWex5as	von Willebrand Factor at exam 5 age & sex	http://www.ncbi.nlm.nih.gov/projects/gap/cgi-bin/analysis.cgi?id=pha002299
ITBiomarkers	Hemostatic	wVWex5mv	von Willebrand Factor at exam 5 multivariable	http://www.ncbi.nlm.nih.gov/projects/gap/cgi-bin/analysis.cgi?id=pha002300
ITBiomarkers	Inflammation	CD40Lplasmaex7as	CD40Ligand plasma exam 7, age- & sex-adjusted, log transformed	http://www.ncbi.nlm.nih.gov/projects/gap/cgi-bin/analysis.cgi?id=pha002301
ITBiomarkers	Inflammation	CD40Lplasmaex7mv	CD40Ligand plasma exam 7 multivariable-adjusted, log transformed	http://www.ncbi.nlm.nih.gov/projects/gap/cgi-bin/analysis.cgi?id=pha002302
ITBiomarkers	Inflammation	CD40Lserumex7as	CD40Ligand serum exam 7, age- & sex-adjusted, log transformed	http://www.ncbi.nlm.nih.gov/projects/gap/cgi-bin/analysis.cgi?id=pha002303
ITBiomarkers	Inflammation	CD40Lserumex7mv	CD40Ligand serum, exam 7 multivariable-adjusted, log transformed	http://www.ncbi.nlm.nih.gov/projects/gap/cgi-bin/analysis.cgi?id=pha002304
ITBiomarkers	Inflammation	CRPavg267asB	Log C-reactive protein average exam 2, 6 & 7 age- & sex-adjusted	http://www.ncbi.nlm.nih.gov/projects/gap/cgi-bin/analysis.cgi?id=pha002329
ITBiomarkers	Inflammation	CRPavg267mvB	Log C-reactive protein average exam 2, 6 & 7, multivariable-adjusted	http://www.ncbi.nlm.nih.gov/projects/gap/cgi-bin/analysis.cgi?id=pha002330
ITBiomarkers	Inflammation	CRPex2asB	Log C-reactive protein exam 2, age- & sex-adjusted	http://www.ncbi.nlm.nih.gov/projects/gap/cgi-bin/analysis.cgi?id=pha002331
ITBiomarkers	Inflammation	CRPex2mvB	Log C-reactive protein exam 2, multivariable-adjusted	http://www.ncbi.nlm.nih.gov/projects/gap/cgi-bin/analysis.cgi?id=pha002332
ITBiomarkers	Inflammation	CRPex5as	Log C-reactive protein Hemagen Assay exam 5, age- & sex-adjusted	http://www.ncbi.nlm.nih.gov/projects/gap/cgi-bin/analysis.cgi?id=pha002305
ITBiomarkers	Inflammation	CRPex5mv	Log C-reactive protein Hemagen Assay exam 5, multivariable-adjusted	http://www.ncbi.nlm.nih.gov/projects/gap/cgi-bin/analysis.cgi?id=pha002306
ITBiomarkers	Inflammation	CRPex6as	Log C-reactive protein exam 6, age- & sex-adjusted	http://www.ncbi.nlm.nih.gov/projects/gap/cgi-bin/analysis.cgi?id=pha002307
ITBiomarkers	Inflammation	CRPex6mv	Log C-reactive protein exam 6, multivariable-adjusted	http://www.ncbi.nlm.nih.gov/projects/gap/cgi-bin/analysis.cgi?id=pha002308
ITBiomarkers	Inflammation	CRPex7as	Log C-reactive protein exam 7, age- & sex-adjusted	http://www.ncbi.nlm.nih.gov/projects/gap/cgi-bin/analysis.cgi?id=pha002309
ITBiomarkers	Inflammation	CRPex7mv	Log C-reactive protein exam 7, multivariable-adjusted	http://www.ncbi.nlm.nih.gov/projects/gap/cgi-bin/analysis.cgi?id=pha002310
ITBiomarkers	Inflammation	ICAM1ex7as	Log intercellular adhesion molecule-1 exam 7, age- & sex-adjusted	http://www.ncbi.nlm.nih.gov/projects/gap/cgi-bin/analysis.cgi?id=pha002311
ITBiomarkers	Inflammation	ICAM1ex7mv	Log intercellular adhesion molecule-1 exam 7, multivariable-adjusted	http://www.ncbi.nlm.nih.gov/projects/gap/cgi-bin/analysis.cgi?id=pha002312
ITBiomarkers	Inflammation	IL6ex7as	Log interleukin 6 Exam 7,age- & sex-adjusted	http://www.ncbi.nlm.nih.gov/projects/gap/cgi-bin/analysis.cgi?id=pha002313
ITBiomarkers	Inflammation	IL6ex7mv	Log interleukin 6 Exam 7,multivariable-adjusted	http://www.ncbi.nlm.nih.gov/projects/gap/cgi-bin/analysis.cgi?id=pha002314
ITBiomarkers	Inflammation	IsoCrUrinePex7as	Log urinary isoprostanes/creatinine exam 7, age- & sex-adjusted	http://www.ncbi.nlm.nih.gov/projects/gap/cgi-bin/analysis.cgi?id=pha002315
ITBiomarkers	Inflammation	IsoCrUrinePex7mv	Log urinary isoprostanes/creatinine exam 7, multivariable-adjusted	http://www.ncbi.nlm.nih.gov/projects/gap/cgi-bin/analysis.cgi?id=pha002316
ITBiomarkers	Inflammation	MCP1ex7as	Log monocyte chemoattractant protein 1 exam 7, age- & sex-adjusted	http://www.ncbi.nlm.nih.gov/projects/gap/cgi-bin/analysis.cgi?id=pha002317
ITBiomarkers	Inflammation	MCP1ex7mv	Log monocyte chemoattractant protein 1 exam 7, multivariable-adjusted	http://www.ncbi.nlm.nih.gov/projects/gap/cgi-bin/analysis.cgi?id=pha002318
ITBiomarkers	Inflammation	MPOex7as	Log myeloperoxidase exam 7, age- & sex-adjusted	http://www.ncbi.nlm.nih.gov/projects/gap/cgi-bin/analysis.cgi?id=pha002319

Group	Category	Trait Label	Name	Linkage Link
ITBiomarkers	Inflammation	MPOex7mv	Log myeloperoxidase exam 7, multivariable-adjusted	http://www.ncbi.nlm.nih.gov/projects/gap/cgi-bin/analysis.cgi?id=pha002320
ITBiomarkers	Inflammation	OPGex7as	Log osteoprotegerin exam 7, age- & sex-adjusted	http://www.ncbi.nlm.nih.gov/projects/gap/cgi-bin/analysis.cgi?id=pha002321
ITBiomarkers	Inflammation	OPGex7mv	Log osteoprotegerin exam 7, multivariable-adjusted	http://www.ncbi.nlm.nih.gov/projects/gap/cgi-bin/analysis.cgi?id=pha002322
ITBiomarkers	Inflammation	Pselectinex7as	Log P-selectin exam 7, age- & sex-adjusted	http://www.ncbi.nlm.nih.gov/projects/gap/cgi-bin/analysis.cgi?id=pha002323
ITBiomarkers	Inflammation	Pselectinex7mv	Log P-selectin exam 7, multivariable-adjusted	http://www.ncbi.nlm.nih.gov/projects/gap/cgi-bin/analysis.cgi?id=pha002324
ITBiomarkers	Inflammation	TNFAex7as	Log tumor necrosis factor alpha exam 7, age- & sex-adjusted	http://www.ncbi.nlm.nih.gov/projects/gap/cgi-bin/analysis.cgi?id=pha002325
ITBiomarkers	Inflammation	TNFAex7mv	Log tumor necrosis factor alpha exam 7, multivariable-adjusted	http://www.ncbi.nlm.nih.gov/projects/gap/cgi-bin/analysis.cgi?id=pha002326
ITBiomarkers	Inflammation	TNFRIIex7as	Log tumor necrosis factor receptor II exam 7, age- & sex-adjusted	http://www.ncbi.nlm.nih.gov/projects/gap/cgi-bin/analysis.cgi?id=pha002327
ITBiomarkers	Inflammation	TNFRIIex7mv	Log tumor necrosis factor receptor II exam 7 multivariable-adjusted	http://www.ncbi.nlm.nih.gov/projects/gap/cgi-bin/analysis.cgi?id=pha002328
ITBiomarkers	LFT	AlkPhosex2asWIN	Log alkaline phosphatase exam 2 age- & sex-adjusted, winsorized	http://www.ncbi.nlm.nih.gov/projects/gap/cgi-bin/analysis.cgi?id=pha002333
ITBiomarkers	LFT	AlkPhosex2mvWIN	Log alkaline phosphatase exam 2, multivariable-adjusted, lwinsorized	http://www.ncbi.nlm.nih.gov/projects/gap/cgi-bin/analysis.cgi?id=pha002334
ITBiomarkers	LFT	ALTex2asNL	Log alanine transaminase exam 2, age- & sex-adjusted, normalized deviates	http://www.ncbi.nlm.nih.gov/projects/gap/cgi-bin/analysis.cgi?id=pha002335
ITBiomarkers	LFT	ALTex2mvNL	Log alanine transaminase exam 2; multivariable-adjusted, normalized deviate	http://www.ncbi.nlm.nih.gov/projects/gap/cgi-bin/analysis.cgi?id=pha002336
ITBiomarkers	LFT	ASTex2asNL	Log aspartate aminotransferase exam 2, age- sex-adjusted normalized deviate	http://www.ncbi.nlm.nih.gov/projects/gap/cgi-bin/analysis.cgi?id=pha002337
ITBiomarkers	LFT	ASTex2mvNL	Log aspartate aminotransferase exam 2 multiv.-adjusted, normalized deviates	http://www.ncbi.nlm.nih.gov/projects/gap/cgi-bin/analysis.cgi?id=pha002338
ITBiomarkers	LFT	Bilirubinex2as	Log bilirubin exam 2, age- & sex-adjusted	http://www.ncbi.nlm.nih.gov/projects/gap/cgi-bin/analysis.cgi?id=pha002339
ITBiomarkers	LFT	Bilirubinex2mv	Log bilirubin exam 2 multivariable	http://www.ncbi.nlm.nih.gov/projects/gap/cgi-bin/analysis.cgi?id=pha002340
ITBiomarkers	LFT	GGTex2as	Log gamma-glutamyl transferase exam 2, age- & sex-adjusted	http://www.ncbi.nlm.nih.gov/projects/gap/cgi-bin/analysis.cgi?id=pha002341
ITBiomarkers	LFT	GGTex2mv	Log Gamma-glutamyl transferase exam 2, Multivariable,adjusted	http://www.ncbi.nlm.nih.gov/projects/gap/cgi-bin/analysis.cgi?id=pha002342
ITBiomarkers	Neurohumoral	ANPex6as	Log atrial natriuretic peptide exam 6, age- & sex-adjusted Tobit-model	http://www.ncbi.nlm.nih.gov/projects/gap/cgi-bin/analysis.cgi?id=pha002343
ITBiomarkers	Neurohumoral	ANPex6mv	Log atrial natriuretic peptide exam 6, multivariable-adjusted Tobit model	http://www.ncbi.nlm.nih.gov/projects/gap/cgi-bin/analysis.cgi?id=pha002344
ITBiomarkers	Neurohumoral	BNPex6as	Log brain natriuretic peptide exam 6, age- & sex-adjusted Tobit model	http://www.ncbi.nlm.nih.gov/projects/gap/cgi-bin/analysis.cgi?id=pha002345
ITBiomarkers	Neurohumoral	BNPex6mv	Log brain natriuretic peptide exam 6, multivariable-adjusted Tobit model	http://www.ncbi.nlm.nih.gov/projects/gap/cgi-bin/analysis.cgi?id=pha002346
ITBiomarkers	Vitamins	VitD25OHex6or7as	25(OH)-D exam 6 or 7, age & sex adjusted	http://www.ncbi.nlm.nih.gov/projects/gap/cgi-bin/analysis.cgi?id=pha002347
ITBiomarkers	Vitamins	VitD25OHex6or7mv	25(OH)-D exam 6 or 7, multivariable-adjusted; covariates from vit D exam	http://www.ncbi.nlm.nih.gov/projects/gap/cgi-bin/analysis.cgi?id=pha002348
ITBiomarkers	Vitamins	VitKPhylloqex6or7as	Vit K exam 6 or 7, age & sex adjusted, log transformed, no warfarin	http://www.ncbi.nlm.nih.gov/projects/gap/cgi-bin/analysis.cgi?id=pha002349
ITBiomarkers	Vitamins	VitKPhylloqex6or7mv	Vit K exam 6 or 7, multivariable-adjusted, log transformed, no warfarin	http://www.ncbi.nlm.nih.gov/projects/gap/cgi-bin/analysis.cgi?id=pha002350
ITBiomarkers	Vitamins	VitKPucOCex6or7asNL	%ucOC, exam 6 or 7, age,sex-adjust, log trans., norm. deviates, no warfarin	http://www.ncbi.nlm.nih.gov/projects/gap/cgi-bin/analysis.cgi?id=pha002351
ITBiomarkers	Vitamins	VitKPucOCex6or7mvNL	%ucOC, exam 6 or 7, multivariable, log-trans., norm. deviates, no warfarin	http://www.ncbi.nlm.nih.gov/projects/gap/cgi-bin/analysis.cgi?id=pha002352
Metabolic	Adiposity	allhgt1602	adj height, offsp 2 & cohort 16 exams	http://www.ncbi.nlm.nih.gov/projects/gap/cgi-bin/analysis.cgi?id=pha002353
Metabolic	Adiposity	allhgt1803	adj height, offsp 3 & cohort 18 exams	http://www.ncbi.nlm.nih.gov/projects/gap/cgi-bin/analysis.cgi?id=pha002354
Metabolic	Adiposity	allhgt1x01	adj height, offsp 1 & cohort 10 exams	http://www.ncbi.nlm.nih.gov/projects/gap/cgi-bin/analysis.cgi?id=pha002355
Metabolic	Adiposity	allhgt2004	adj height, offsp 4 & cohort 20 exams	http://www.ncbi.nlm.nih.gov/projects/gap/cgi-bin/analysis.cgi?id=pha002356
Metabolic	Adiposity	allhgt2205	adj height, offsp 5 & cohort 22 exams	http://www.ncbi.nlm.nih.gov/projects/gap/cgi-bin/analysis.cgi?id=pha002357
Metabolic	Adiposity	allhgt2406	adj height, offsp 6 & cohort 24 exams	http://www.ncbi.nlm.nih.gov/projects/gap/cgi-bin/analysis.cgi?id=pha002358
Metabolic	Adiposity	allhgt2607	adj height, offsp 7 & cohort 26 exams	http://www.ncbi.nlm.nih.gov/projects/gap/cgi-bin/analysis.cgi?id=pha002359
Metabolic	Adiposity	allmeanhgt	adj mean height, offsp 1-7 & cohort 10,16,18,20,22,24,26 exams	http://www.ncbi.nlm.nih.gov/projects/gap/cgi-bin/analysis.cgi?id=pha002360
Metabolic	Adiposity	allowst0007	adj waist, offsp 7	http://www.ncbi.nlm.nih.gov/projects/gap/cgi-bin/analysis.cgi?id=pha002361
Metabolic	Adiposity	allrankbmi1602	rank adj bmi, offsp 2 & cohort 16 exams	http://www.ncbi.nlm.nih.gov/projects/gap/cgi-bin/analysis.cgi?id=pha002362
Metabolic	Adiposity	allrankbmi1803	rank adj bmi, offsp 3 & cohort 18 exams	http://www.ncbi.nlm.nih.gov/projects/gap/cgi-bin/analysis.cgi?id=pha002363
Metabolic	Adiposity	allrankbmi1x01	rank adj bmi, offsp 1 & cohort 10 exams	http://www.ncbi.nlm.nih.gov/projects/gap/cgi-bin/analysis.cgi?id=pha002364
Metabolic	Adiposity	allrankbmi2004	rank adj bmi, offsp 4 & cohort 20 exams	http://www.ncbi.nlm.nih.gov/projects/gap/cgi-bin/analysis.cgi?id=pha002365
Metabolic	Adiposity	allrankbmi2205	rank adj bmi, offsp 5 & cohort 22 exams	http://www.ncbi.nlm.nih.gov/projects/gap/cgi-bin/analysis.cgi?id=pha002366
Metabolic	Adiposity	allrankbmi2406	rank adj bmi, offsp 6 & cohort 24 exams	http://www.ncbi.nlm.nih.gov/projects/gap/cgi-bin/analysis.cgi?id=pha002367
Metabolic	Adiposity	allrankbmi2607	rank adj bmi, offsp 7 & cohort 26 exams	http://www.ncbi.nlm.nih.gov/projects/gap/cgi-bin/analysis.cgi?id=pha002368
Metabolic	Adiposity	allrankchgbmi	rank adj bmi change, offsp 1-7 & cohort exams 10-26	http://www.ncbi.nlm.nih.gov/projects/gap/cgi-bin/analysis.cgi?id=pha002369
Metabolic	Adiposity	allrankchgwgt	rank adj weight change, offsp 1-7 & cohort exams 10-26	http://www.ncbi.nlm.nih.gov/projects/gap/cgi-bin/analysis.cgi?id=pha002370
Metabolic	Adiposity	allrankmeanbmi	rank adj mean bmi, offsp 1-7 & cohort 10,16,18,20,22,24,26 exams	http://www.ncbi.nlm.nih.gov/projects/gap/cgi-bin/analysis.cgi?id=pha002371
Metabolic	Adiposity	allrankmeanwgt	rank adj mean weight, offsp 1-7 & cohort 10,16,18,20,22,24,26 exams	http://www.ncbi.nlm.nih.gov/projects/gap/cgi-bin/analysis.cgi?id=pha002372
Metabolic	Adiposity	allrankochgwst	rank adj waist change, offsp 4 to 7	http://www.ncbi.nlm.nih.gov/projects/gap/cgi-bin/analysis.cgi?id=pha002373
Metabolic	Adiposity	allrankomeanwst	rank adj mean waist, offsp 4 to 7	http://www.ncbi.nlm.nih.gov/projects/gap/cgi-bin/analysis.cgi?id=pha002374
Metabolic	Adiposity	allrankwgt1602	rank adj weight, offsp 2 & cohort 16 exams	http://www.ncbi.nlm.nih.gov/projects/gap/cgi-bin/analysis.cgi?id=pha002375
Metabolic	Adiposity	allrankwgt1803	rank adj weight, offsp 3 & cohort 18 exams	http://www.ncbi.nlm.nih.gov/projects/gap/cgi-bin/analysis.cgi?id=pha002376
Metabolic	Adiposity	allrankwgt1x01	rank adj weight, offsp 1 & cohort 10 exams	http://www.ncbi.nlm.nih.gov/projects/gap/cgi-bin/analysis.cgi?id=pha002377
Metabolic	Adiposity	allrankwgt2004	rank adj weight, offsp 4 & cohort 20 exams	http://www.ncbi.nlm.nih.gov/projects/gap/cgi-bin/analysis.cgi?id=pha002378
Metabolic	Adiposity	allrankwgt2205	rank adj weight, offsp 5 & cohort 22 exams	http://www.ncbi.nlm.nih.gov/projects/gap/cgi-bin/analysis.cgi?id=pha002379

Group	Category	Trait Label	Name	Linkage Link
Metabolic	Adiposity	allrankwgt2406	rank adj weight, offsp 6 & cohort 24 exams	http://www.ncbi.nlm.nih.gov/projects/gap/cgi-bin/analysis.cgi?id=pha002380
Metabolic	Adiposity	allrankwgt2607	rank adj weight, offsp 7 & cohort 26 exams	http://www.ncbi.nlm.nih.gov/projects/gap/cgi-bin/analysis.cgi?id=pha002381
Metabolic	Adiposity	allrankwst2306	rank adj waist, offsp 6 & cohort exam 23	http://www.ncbi.nlm.nih.gov/projects/gap/cgi-bin/analysis.cgi?id=pha002382
Metabolic	Adiposity	allwst2004	adj waist, offsp 4 & cohort 20 exams	http://www.ncbi.nlm.nih.gov/projects/gap/cgi-bin/analysis.cgi?id=pha002383
Metabolic	Adiposity	allwst2205	adj waist, offsp 5 & cohort 22 exams	http://www.ncbi.nlm.nih.gov/projects/gap/cgi-bin/analysis.cgi?id=pha002384
Metabolic	Adiposity	femchgbmi	Female adj bmi change, offsp 1-7 & cohort exams 10-26	http://www.ncbi.nlm.nih.gov/projects/gap/cgi-bin/analysis.cgi?id=pha002385
Metabolic	Adiposity	femhgt1602	Female adj height, offsp 2 & cohort 16 exams	http://www.ncbi.nlm.nih.gov/projects/gap/cgi-bin/analysis.cgi?id=pha002386
Metabolic	Adiposity	femhgt1x01	Female adj height, offsp 1 & cohort 10 exams	http://www.ncbi.nlm.nih.gov/projects/gap/cgi-bin/analysis.cgi?id=pha002387
Metabolic	Adiposity	femhgt2004	Female adj height, offsp 4 & cohort 20 exams	http://www.ncbi.nlm.nih.gov/projects/gap/cgi-bin/analysis.cgi?id=pha002388
Metabolic	Adiposity	femhgt2205	Female adj height, offsp 5 & cohort 22 exams	http://www.ncbi.nlm.nih.gov/projects/gap/cgi-bin/analysis.cgi?id=pha002389
Metabolic	Adiposity	femhgt2406	Female adj height, offsp 6 & cohort 24 exams	http://www.ncbi.nlm.nih.gov/projects/gap/cgi-bin/analysis.cgi?id=pha002390
Metabolic	Adiposity	femhgt2607	Female adj height, offsp 7 & cohort 26 exams	http://www.ncbi.nlm.nih.gov/projects/gap/cgi-bin/analysis.cgi?id=pha002391
Metabolic	Adiposity	femmeanhgt	Female adj mean height, offsp 1-7 & cohort 10,16,18,20,22,24,26 exams	http://www.ncbi.nlm.nih.gov/projects/gap/cgi-bin/analysis.cgi?id=pha002392
Metabolic	Adiposity	femochgwst	Female adj waist change, offsp 4 to 7	http://www.ncbi.nlm.nih.gov/projects/gap/cgi-bin/analysis.cgi?id=pha002393
Metabolic	Adiposity	femowst0007	Female adj waist, offsp 7	http://www.ncbi.nlm.nih.gov/projects/gap/cgi-bin/analysis.cgi?id=pha002394
Metabolic	Adiposity	femrankbmi1602	Female rank adj bmi, offsp 2 & cohort 16 exams	http://www.ncbi.nlm.nih.gov/projects/gap/cgi-bin/analysis.cgi?id=pha002395
Metabolic	Adiposity	femrankbmi1803	Female rank adj bmi, offsp 3 & cohort 18 exams	http://www.ncbi.nlm.nih.gov/projects/gap/cgi-bin/analysis.cgi?id=pha002396
Metabolic	Adiposity	femrankbmi1x01	Female rank adj bmi, offsp 1 & cohort 10 exams	http://www.ncbi.nlm.nih.gov/projects/gap/cgi-bin/analysis.cgi?id=pha002397
Metabolic	Adiposity	femrankbmi2004	Female rank adj bmi, offsp 4 & cohort 20 exams	http://www.ncbi.nlm.nih.gov/projects/gap/cgi-bin/analysis.cgi?id=pha002398
Metabolic	Adiposity	femrankbmi2205	Female rank adj bmi, offsp 5 & cohort 22 exams	http://www.ncbi.nlm.nih.gov/projects/gap/cgi-bin/analysis.cgi?id=pha002399
Metabolic	Adiposity	femrankbmi2406	Female rank adj bmi, offsp 6 & cohort 24 exams	http://www.ncbi.nlm.nih.gov/projects/gap/cgi-bin/analysis.cgi?id=pha002400
Metabolic	Adiposity	femrankbmi2607	Female rank adj bmi, offsp 7 & cohort 26 exams	http://www.ncbi.nlm.nih.gov/projects/gap/cgi-bin/analysis.cgi?id=pha002401
Metabolic	Adiposity	femrankchgwgt	Female rank adj weight change, offsp 1-7 & cohort exams 10-26	http://www.ncbi.nlm.nih.gov/projects/gap/cgi-bin/analysis.cgi?id=pha002402
Metabolic	Adiposity	femrankmeanbmi	Female rank adj mean bmi, offsp 1-7 & cohort 10,16,18,20,22,24,26 exams	http://www.ncbi.nlm.nih.gov/projects/gap/cgi-bin/analysis.cgi?id=pha002403
Metabolic	Adiposity	femrankmeanwgt	Female rank adj mean weight, offsp 1-7 & cohort 10,16,18,20,22,24,26 exams	http://www.ncbi.nlm.nih.gov/projects/gap/cgi-bin/analysis.cgi?id=pha002404
Metabolic	Adiposity	femrankomeanwst	Female rank adj mean waist, offsp 4 to 7	http://www.ncbi.nlm.nih.gov/projects/gap/cgi-bin/analysis.cgi?id=pha002405
Metabolic	Adiposity	femrankwgt1602	Female rank adj weight, offsp 2 & cohort 16 exams	http://www.ncbi.nlm.nih.gov/projects/gap/cgi-bin/analysis.cgi?id=pha002406
Metabolic	Adiposity	femrankwgt1803	Female rank adj weight, offsp 3 & cohort 18 exams	http://www.ncbi.nlm.nih.gov/projects/gap/cgi-bin/analysis.cgi?id=pha002407
Metabolic	Adiposity	femrankwgt1x01	Female rank adj weight, offsp 1 & cohort 10 exams	http://www.ncbi.nlm.nih.gov/projects/gap/cgi-bin/analysis.cgi?id=pha002408
Metabolic	Adiposity	femrankwgt2004	Female rank adj weight, offsp 4 & cohort 20 exams	http://www.ncbi.nlm.nih.gov/projects/gap/cgi-bin/analysis.cgi?id=pha002409
Metabolic	Adiposity	femrankwgt2205	Female rank adj weight, offsp 5 & cohort 22 exams	http://www.ncbi.nlm.nih.gov/projects/gap/cgi-bin/analysis.cgi?id=pha002410
Metabolic	Adiposity	femrankwgt2406	Female rank adj weight, offsp 6 & cohort 24 exams	http://www.ncbi.nlm.nih.gov/projects/gap/cgi-bin/analysis.cgi?id=pha002411
Metabolic	Adiposity	femrankwgt2607	Female rank adj weight, offsp 7 & cohort 26 exams	http://www.ncbi.nlm.nih.gov/projects/gap/cgi-bin/analysis.cgi?id=pha002412
Metabolic	Adiposity	femwst2004	Female adj waist, offsp 4 & cohort 20 exams	http://www.ncbi.nlm.nih.gov/projects/gap/cgi-bin/analysis.cgi?id=pha002413
Metabolic	Adiposity	femwst2205	Female adj waist, offsp 5 & cohort 22 exams	http://www.ncbi.nlm.nih.gov/projects/gap/cgi-bin/analysis.cgi?id=pha002414
Metabolic	Adiposity	femwst2306	Female adj waist, offsp 6 & cohort exam 23	http://www.ncbi.nlm.nih.gov/projects/gap/cgi-bin/analysis.cgi?id=pha002415
Metabolic	Adiposity	malebmi1x01	Male adj bmi, offsp 1 & cohort 10 exams	http://www.ncbi.nlm.nih.gov/projects/gap/cgi-bin/analysis.cgi?id=pha002416
Metabolic	Adiposity	malehgt1803	Male adj height, offsp 3 & cohort 18 exams	http://www.ncbi.nlm.nih.gov/projects/gap/cgi-bin/analysis.cgi?id=pha002417
Metabolic	Adiposity	malehgt1x01	Male adj height, offsp 1 & cohort 10 exams	http://www.ncbi.nlm.nih.gov/projects/gap/cgi-bin/analysis.cgi?id=pha002418
Metabolic	Adiposity	malehgt2004	Male adj height, offsp 4 & cohort 20 exams	http://www.ncbi.nlm.nih.gov/projects/gap/cgi-bin/analysis.cgi?id=pha002419
Metabolic	Adiposity	malehgt2607	Male adj height, offsp 7 & cohort 26 exams	http://www.ncbi.nlm.nih.gov/projects/gap/cgi-bin/analysis.cgi?id=pha002420
Metabolic	Adiposity	malemeanbmi	Male adj mean bmi, offsp 1-7 & cohort 10,16,18,20,22,24,26 exams	http://www.ncbi.nlm.nih.gov/projects/gap/cgi-bin/analysis.cgi?id=pha002421
Metabolic	Adiposity	malemeanhgt	Male adj mean hgt, offsp 1-7 & cohort 10,16,18,20,22,24,26 exams	http://www.ncbi.nlm.nih.gov/projects/gap/cgi-bin/analysis.cgi?id=pha002422
Metabolic	Adiposity	malemeanwgt	Male adj mean weight, offsp 1-7 & cohort 10,16,18,20,22,24,26 exams	http://www.ncbi.nlm.nih.gov/projects/gap/cgi-bin/analysis.cgi?id=pha002423
Metabolic	Adiposity	maleomeanwst	Male adj mean waist, offsp 4 to 7	http://www.ncbi.nlm.nih.gov/projects/gap/cgi-bin/analysis.cgi?id=pha002424
Metabolic	Adiposity	maleowst0007	Male adj waist, offsp 7	http://www.ncbi.nlm.nih.gov/projects/gap/cgi-bin/analysis.cgi?id=pha002425
Metabolic	Adiposity	malerankbmi1602	Male rank adj bmi, offsp 2 & cohort 16 exams	http://www.ncbi.nlm.nih.gov/projects/gap/cgi-bin/analysis.cgi?id=pha002426
Metabolic	Adiposity	malerankbmi1803	Male rank adj bmi, offsp 3 & cohort 18 exams	http://www.ncbi.nlm.nih.gov/projects/gap/cgi-bin/analysis.cgi?id=pha002427
Metabolic	Adiposity	malerankbmi2004	Male rank adj bmi, offsp 4 & cohort 20 exams	http://www.ncbi.nlm.nih.gov/projects/gap/cgi-bin/analysis.cgi?id=pha002428
Metabolic	Adiposity	malerankbmi2205	Male rank adj bmi, offsp 5 & cohort 22 exams	http://www.ncbi.nlm.nih.gov/projects/gap/cgi-bin/analysis.cgi?id=pha002429
Metabolic	Adiposity	malerankbmi2406	Male rank adj bmi, offsp 6 & cohort 24 exams	http://www.ncbi.nlm.nih.gov/projects/gap/cgi-bin/analysis.cgi?id=pha002430
Metabolic	Adiposity	malerankbmi2607	Male rank adj bmi, offsp 7 & cohort 26 exams	http://www.ncbi.nlm.nih.gov/projects/gap/cgi-bin/analysis.cgi?id=pha002431
Metabolic	Adiposity	malerankchgbmi	Male rank adj bmi change, offsp 1-7 & cohort exams 10-26	http://www.ncbi.nlm.nih.gov/projects/gap/cgi-bin/analysis.cgi?id=pha002432
Metabolic	Adiposity	malerankchgwgt	Male rank adj weight change, offsp 1-7 & cohort exams 10-26	http://www.ncbi.nlm.nih.gov/projects/gap/cgi-bin/analysis.cgi?id=pha002433
Metabolic	Adiposity	malerankhgt2406	Male rank adj height, offsp 6 & cohort 24 exams	http://www.ncbi.nlm.nih.gov/projects/gap/cgi-bin/analysis.cgi?id=pha002434
Metabolic	Adiposity	malerankochgwst	Male rank adj waist change, offsp 4 to 7	http://www.ncbi.nlm.nih.gov/projects/gap/cgi-bin/analysis.cgi?id=pha002435

Group	Category	Trait Label	Name	Linkage Link
Metabolic	Adiposity	malerankwgt1602	Male rank adj weight, offsp 2 & cohort 16 exams	http://www.ncbi.nlm.nih.gov/projects/gap/cgi-bin/analysis.cgi?id=pha002436
Metabolic	Adiposity	malerankwgt2004	Male rank adj weight, offsp 4 & cohort 20 exams	http://www.ncbi.nlm.nih.gov/projects/gap/cgi-bin/analysis.cgi?id=pha002437
Metabolic	Adiposity	malerankwgt2205	Male rank adj weight, offsp 5 & cohort 22 exams	http://www.ncbi.nlm.nih.gov/projects/gap/cgi-bin/analysis.cgi?id=pha002438
Metabolic	Adiposity	malerankwst2306	Male rank adj waist, offsp 6 & cohort exam 23	http://www.ncbi.nlm.nih.gov/projects/gap/cgi-bin/analysis.cgi?id=pha002439
Metabolic	Adiposity	malewgt1803	Male adj weight, offsp 3 & cohort 18 exams	http://www.ncbi.nlm.nih.gov/projects/gap/cgi-bin/analysis.cgi?id=pha002440
Metabolic	Adiposity	malewgt1x01	Male adj weight, offsp 1 & cohort 10 exams	http://www.ncbi.nlm.nih.gov/projects/gap/cgi-bin/analysis.cgi?id=pha002441
Metabolic	Adiposity	malewgt2406	Male adj weight, offsp 6 & cohort 24 exams	http://www.ncbi.nlm.nih.gov/projects/gap/cgi-bin/analysis.cgi?id=pha002442
Metabolic	Adiposity	malewgt2607	Male adj weight, offsp 7 & cohort 26 exams	http://www.ncbi.nlm.nih.gov/projects/gap/cgi-bin/analysis.cgi?id=pha002443
Metabolic	Adiposity	malewst2004	Male adj waist, offsp 4 & cohort 20 exams	http://www.ncbi.nlm.nih.gov/projects/gap/cgi-bin/analysis.cgi?id=pha002444
Metabolic	Adiposity	malewst2205	Male adj waist, offsp 5 & cohort 22 exams	http://www.ncbi.nlm.nih.gov/projects/gap/cgi-bin/analysis.cgi?id=pha002445
Metabolic	Adiposity	SATAS	SAT adj for age, age sqr, sex	http://www.ncbi.nlm.nih.gov/projects/gap/cgi-bin/analysis.cgi?id=pha002446
Metabolic	Adiposity	SATMV	SAT adj for age, age square, sex, meno status, smoking	http://www.ncbi.nlm.nih.gov/projects/gap/cgi-bin/analysis.cgi?id=pha002447
Metabolic	Adiposity	SDAS	Sagittal diameter adj for age, age square, sex	http://www.ncbi.nlm.nih.gov/projects/gap/cgi-bin/analysis.cgi?id=pha002448
Metabolic	Adiposity	SDMV	Sagittal diameter adj for age, age square, sex, meno status, smoking	http://www.ncbi.nlm.nih.gov/projects/gap/cgi-bin/analysis.cgi?id=pha002449
Metabolic	Adiposity	VATAS	VAT adj for age, age squared, sex	http://www.ncbi.nlm.nih.gov/projects/gap/cgi-bin/analysis.cgi?id=pha002450
Metabolic	Adiposity	VATMV	VAT adj for age, age squared, sex, meno status, smoking	http://www.ncbi.nlm.nih.gov/projects/gap/cgi-bin/analysis.cgi?id=pha002451
Metabolic	Adiposity	WCAS	Waist by CT adj for age, age squared, sex	http://www.ncbi.nlm.nih.gov/projects/gap/cgi-bin/analysis.cgi?id=pha002452
Metabolic	Adiposity	WCMV	Waist by CT adj for age, age squared, sex, meno status, smoking	http://www.ncbi.nlm.nih.gov/projects/gap/cgi-bin/analysis.cgi?id=pha002453
Metabolic	Glycemic	res1ladipoq7o	Age-sex adjusted Adiponectin ex7 offspring	http://www.ncbi.nlm.nih.gov/projects/gap/cgi-bin/analysis.cgi?id=pha002454
Metabolic	Glycemic	res2ladipoq7o	Multivariable adjusted Adiponectin ex7 offspring	http://www.ncbi.nlm.nih.gov/projects/gap/cgi-bin/analysis.cgi?id=pha002455
Metabolic	Glycemic	rnk1lfglu5o	Rank Age-sex adjusted Fasting Plasma Glucose ex5 offspring	http://www.ncbi.nlm.nih.gov/projects/gap/cgi-bin/analysis.cgi?id=pha002456
Metabolic	Glycemic	rnk1lfglu7o	Rank Age-sex adjusted Fasting Plasma Glucose ex7 offspring	http://www.ncbi.nlm.nih.gov/projects/gap/cgi-bin/analysis.cgi?id=pha002457
Metabolic	Glycemic	rnk1lfins5o	Rank Age-sex adjusted Fasting Insulin ex5 offspring	http://www.ncbi.nlm.nih.gov/projects/gap/cgi-bin/analysis.cgi?id=pha002458
Metabolic	Glycemic	rnk1lfins7o	Rank Age-sex adjusted Fasting Insulin ex7 offspring	http://www.ncbi.nlm.nih.gov/projects/gap/cgi-bin/analysis.cgi?id=pha002459
Metabolic	Glycemic	rnk1lgutt5o	Rank Age-sex adjusted Insulin Sensitivity ex5 offspring	http://www.ncbi.nlm.nih.gov/projects/gap/cgi-bin/analysis.cgi?id=pha002460
Metabolic	Glycemic	rnk1lhba1c5o	Rank Age-sex adjusted Fasting HbA1c ex5 offspring	http://www.ncbi.nlm.nih.gov/projects/gap/cgi-bin/analysis.cgi?id=pha002461
Metabolic	Glycemic	rnk1lhba1c7o	Rank Age-sex adjusted HbA1c ex7 offspring	http://www.ncbi.nlm.nih.gov/projects/gap/cgi-bin/analysis.cgi?id=pha002462
Metabolic	Glycemic	rnk1lhir5o	Rank Age-sex adjusted HOMA-IR ex5 offspring	http://www.ncbi.nlm.nih.gov/projects/gap/cgi-bin/analysis.cgi?id=pha002463
Metabolic	Glycemic	rnk1lhir7o	Rank Age-sex adjusted HOMA-IR ex7 offspring	http://www.ncbi.nlm.nih.gov/projects/gap/cgi-bin/analysis.cgi?id=pha002464
Metabolic	Glycemic	rnk1lmfglu17o	Rank Age-sex adjusted Mean Plasma Glucose exam1-7 offspring	http://www.ncbi.nlm.nih.gov/projects/gap/cgi-bin/analysis.cgi?id=pha002465
Metabolic	Glycemic	rnk1lretn7o	Rank Age-sex adjusted Resistin ex7 offspring	http://www.ncbi.nlm.nih.gov/projects/gap/cgi-bin/analysis.cgi?id=pha002466
Metabolic	Glycemic	rnk2lfglu5o	Rank multivariable adjusted Fasting Plasma Glucose ex5 offspring	http://www.ncbi.nlm.nih.gov/projects/gap/cgi-bin/analysis.cgi?id=pha002467
Metabolic	Glycemic	rnk2lfglu7o	Rank multivariable adjusted Fasting Plasma Glucose ex7 offspring	http://www.ncbi.nlm.nih.gov/projects/gap/cgi-bin/analysis.cgi?id=pha002468
Metabolic	Glycemic	rnk2lfins5o	Rank multivariable adjusted Fasting Insulin ex5 offspring	http://www.ncbi.nlm.nih.gov/projects/gap/cgi-bin/analysis.cgi?id=pha002469
Metabolic	Glycemic	rnk2lfins7o	Rank multivariable adjusted Fasting Insulin ex7 offspring	http://www.ncbi.nlm.nih.gov/projects/gap/cgi-bin/analysis.cgi?id=pha002470
Metabolic	Glycemic	rnk2lgutt5o	Rank multivariable adjusted Insulin Sensitivity ex5 offspring	http://www.ncbi.nlm.nih.gov/projects/gap/cgi-bin/analysis.cgi?id=pha002471
Metabolic	Glycemic	rnk2lhba1c5o	Rank multivariable adjusted Fasting HbA1c ex5 offspring	http://www.ncbi.nlm.nih.gov/projects/gap/cgi-bin/analysis.cgi?id=pha002472
Metabolic	Glycemic	rnk2lhba1c7o	Rank multivariable adjusted HbA1c ex7 offspring	http://www.ncbi.nlm.nih.gov/projects/gap/cgi-bin/analysis.cgi?id=pha002473
Metabolic	Glycemic	rnk2lhir5o	Rank multivariable adjusted HOMA-IR ex5 offspring	http://www.ncbi.nlm.nih.gov/projects/gap/cgi-bin/analysis.cgi?id=pha002474
Metabolic	Glycemic	rnk2lhir7o	Rank multivariable adjusted HOMA-IR ex7 offspring	http://www.ncbi.nlm.nih.gov/projects/gap/cgi-bin/analysis.cgi?id=pha002475
Metabolic	Glycemic	rnk2lmfglu17o	Rank multivariable adjusted Mean Plasma Glucose exam1-7 offspring	http://www.ncbi.nlm.nih.gov/projects/gap/cgi-bin/analysis.cgi?id=pha002476
Metabolic	Glycemic	rnk2lretn7o	Rank multivariable adjusted Resistin ex7 offspring	http://www.ncbi.nlm.nih.gov/projects/gap/cgi-bin/analysis.cgi?id=pha002477
Metabolic	Lipids	ApoC3a	Multivariable adj Plasma ApoCIII Level Offsp ex5	http://www.ncbi.nlm.nih.gov/projects/gap/cgi-bin/analysis.cgi?id=pha002478
Metabolic	Lipids	ApoC3b	Age-sex adj Plasma ApoCIII Level Offsp ex5	http://www.ncbi.nlm.nih.gov/projects/gap/cgi-bin/analysis.cgi?id=pha002479
Metabolic	Lipids	cholhdl1a	Multvariable adj Chol-HDL Ratio Offsp ex1	http://www.ncbi.nlm.nih.gov/projects/gap/cgi-bin/analysis.cgi?id=pha002480
Metabolic	Lipids	cholhdl1b	Age-sex adj Chol-HDL Ratio Offsp ex1	http://www.ncbi.nlm.nih.gov/projects/gap/cgi-bin/analysis.cgi?id=pha002481
Metabolic	Lipids	cholhdl2b	Age-sex adj Chol-HDL Ratio Offsp ex2	http://www.ncbi.nlm.nih.gov/projects/gap/cgi-bin/analysis.cgi?id=pha002482
Metabolic	Lipids	cholhdl3b	Age-sex adj Chol-HDL Ratio Offsp ex3	http://www.ncbi.nlm.nih.gov/projects/gap/cgi-bin/analysis.cgi?id=pha002483
Metabolic	Lipids	cholhdl4b	Age-sex adj Chol-HDL Ratio Offsp ex4	http://www.ncbi.nlm.nih.gov/projects/gap/cgi-bin/analysis.cgi?id=pha002484
Metabolic	Lipids	cholhdl5b	Age-sex adj Chol-HDL Ratio Offsp ex5	http://www.ncbi.nlm.nih.gov/projects/gap/cgi-bin/analysis.cgi?id=pha002485
Metabolic	Lipids	cholhdl7b	Age-sex adj Chol-HDL Ratio Offsp ex7	http://www.ncbi.nlm.nih.gov/projects/gap/cgi-bin/analysis.cgi?id=pha002486
Metabolic	Lipids	hdl35b	Age-sex adj HDL3 Offsp ex5	http://www.ncbi.nlm.nih.gov/projects/gap/cgi-bin/analysis.cgi?id=pha002487
Metabolic	Lipids	HDLNMRint4a	Multivariable adj NMR HDL Int Offsp ex4 or TG > 400	http://www.ncbi.nlm.nih.gov/projects/gap/cgi-bin/analysis.cgi?id=pha002488
Metabolic	Lipids	HDLNMRint4b	Age-sex adj NMR HDL Int Offsp ex4 or TG > 400	http://www.ncbi.nlm.nih.gov/projects/gap/cgi-bin/analysis.cgi?id=pha002489
Metabolic	Lipids	HDLNMRsm4a	Multivariable adj NMR HDL Sm Offsp ex4 or TG > 400	http://www.ncbi.nlm.nih.gov/projects/gap/cgi-bin/analysis.cgi?id=pha002490
Metabolic	Lipids	HDLNMRsm4b	Age-sex adj NMR HDL Sm Offsp ex4 or TG > 400	http://www.ncbi.nlm.nih.gov/projects/gap/cgi-bin/analysis.cgi?id=pha002491

Group	Category	Trait Label	Name	Linkage Link
Metabolic	Lipids	HDLNMRsz4b	Age-sex adj NMR HDL Size Offsp ex4 or TG > 400	http://www.ncbi.nlm.nih.gov/projects/gap/cgi-bin/analysis.cgi?id=pha002492
Metabolic	Lipids	ldl3b	Age-sex adj LDL Offsp ex3	http://www.ncbi.nlm.nih.gov/projects/gap/cgi-bin/analysis.cgi?id=pha002493
Metabolic	Lipids	ldl5b	Age-sex adj LDL Offsp ex5	http://www.ncbi.nlm.nih.gov/projects/gap/cgi-bin/analysis.cgi?id=pha002494
Metabolic	Lipids	LDLNMRlg4a	Multivariable adj NMR LDL Lg Offsp ex4 or TG > 400	http://www.ncbi.nlm.nih.gov/projects/gap/cgi-bin/analysis.cgi?id=pha002495
Metabolic	Lipids	LDLNMRlg4b	Age-sex adj NMR LDL Lg Offsp ex4 or TG > 400	http://www.ncbi.nlm.nih.gov/projects/gap/cgi-bin/analysis.cgi?id=pha002496
Metabolic	Lipids	LDLNMRsz4a	Multivariable adj NMR LDL Size Offsp ex4 or TG > 400	http://www.ncbi.nlm.nih.gov/projects/gap/cgi-bin/analysis.cgi?id=pha002497
Metabolic	Lipids	LDLNMRsz4b	Age-sex adj NMR LDL Size Offsp ex4 or TG > 400	http://www.ncbi.nlm.nih.gov/projects/gap/cgi-bin/analysis.cgi?id=pha002498
Metabolic	Lipids	rApoA14a	Multivariable adj Plasma ApoA1 Level Offsp ex4, ranked res	http://www.ncbi.nlm.nih.gov/projects/gap/cgi-bin/analysis.cgi?id=pha002499
Metabolic	Lipids	rApoA14b	Age-sex adj Plasma ApoA1 Level Offsp ex4, ranked res	http://www.ncbi.nlm.nih.gov/projects/gap/cgi-bin/analysis.cgi?id=pha002500
Metabolic	Lipids	rApoB4a	Multivariable adj Plasma ApoB Level Offsp ex4, ranked res	http://www.ncbi.nlm.nih.gov/projects/gap/cgi-bin/analysis.cgi?id=pha002501
Metabolic	Lipids	rApoB4b	Age-sex adj Plasma ApoB Level Offsp ex4, ranked res	http://www.ncbi.nlm.nih.gov/projects/gap/cgi-bin/analysis.cgi?id=pha002502
Metabolic	Lipids	rchol1a	Multivariable adj Chol Offsp ex1, ranked res	http://www.ncbi.nlm.nih.gov/projects/gap/cgi-bin/analysis.cgi?id=pha002503
Metabolic	Lipids	rchol1b	Age-sex adj Chol Offsp ex1, ranked res	http://www.ncbi.nlm.nih.gov/projects/gap/cgi-bin/analysis.cgi?id=pha002504
Metabolic	Lipids	rchol2b	Age-sex adj Chol Offsp ex2, ranked res	http://www.ncbi.nlm.nih.gov/projects/gap/cgi-bin/analysis.cgi?id=pha002505
Metabolic	Lipids	rchol3b	Age-sex adj Chol Offsp ex3, ranked res	http://www.ncbi.nlm.nih.gov/projects/gap/cgi-bin/analysis.cgi?id=pha002506
Metabolic	Lipids	rchol4b	Age-sex adj Chol Offsp ex4, ranked res	http://www.ncbi.nlm.nih.gov/projects/gap/cgi-bin/analysis.cgi?id=pha002507
Metabolic	Lipids	rchol5b	Age-sex adj Chol Offsp ex5, ranked res	http://www.ncbi.nlm.nih.gov/projects/gap/cgi-bin/analysis.cgi?id=pha002508
Metabolic	Lipids	rchol6b	Age-sex adj Chol Offsp ex6, ranked res	http://www.ncbi.nlm.nih.gov/projects/gap/cgi-bin/analysis.cgi?id=pha002509
Metabolic	Lipids	rchol7b	Age-sex adj Chol Offsp ex7, ranked res	http://www.ncbi.nlm.nih.gov/projects/gap/cgi-bin/analysis.cgi?id=pha002510
Metabolic	Lipids	rcholhdl6b	Age-sex adj Chol-HDL Ratio Offsp ex6, ranked res	http://www.ncbi.nlm.nih.gov/projects/gap/cgi-bin/analysis.cgi?id=pha002511
Metabolic	Lipids	rhdl1a	Multivariable adj HDL Offsp ex1, ranked res	http://www.ncbi.nlm.nih.gov/projects/gap/cgi-bin/analysis.cgi?id=pha002512
Metabolic	Lipids	rhdl1b	HDL Offsp ex1, ranked res	http://www.ncbi.nlm.nih.gov/projects/gap/cgi-bin/analysis.cgi?id=pha002513
Metabolic	Lipids	rhdl24a	Multivariable adj HDL2 Offsp ex4, ranked res	http://www.ncbi.nlm.nih.gov/projects/gap/cgi-bin/analysis.cgi?id=pha002514
Metabolic	Lipids	rhdl24b	Age-sex adj HDL2 Offsp ex4, ranked res	http://www.ncbi.nlm.nih.gov/projects/gap/cgi-bin/analysis.cgi?id=pha002515
Metabolic	Lipids	rhdl25a	Multivariable adj HDL2 Offsp ex5, ranked res	http://www.ncbi.nlm.nih.gov/projects/gap/cgi-bin/analysis.cgi?id=pha002516
Metabolic	Lipids	rhdl25b	Age-sex adj HDL2 Offsp ex5, ranked res	http://www.ncbi.nlm.nih.gov/projects/gap/cgi-bin/analysis.cgi?id=pha002517
Metabolic	Lipids	rhdl2b	Age-sex adj HDL Offsp ex2, ranked res	http://www.ncbi.nlm.nih.gov/projects/gap/cgi-bin/analysis.cgi?id=pha002518
Metabolic	Lipids	rhdl34a	Multivariable adj HDL3 Offsp ex4, ranked res	http://www.ncbi.nlm.nih.gov/projects/gap/cgi-bin/analysis.cgi?id=pha002519
Metabolic	Lipids	rhdl34b	Age-sex adj HDL3 Offsp ex4, ranked res	http://www.ncbi.nlm.nih.gov/projects/gap/cgi-bin/analysis.cgi?id=pha002520
Metabolic	Lipids	rhdl35a	Multivariable adj HDL3 Offsp ex5, ranked res	http://www.ncbi.nlm.nih.gov/projects/gap/cgi-bin/analysis.cgi?id=pha002521
Metabolic	Lipids	rhdl3b	Age-sex adj HDL Offsp ex3, ranked res	http://www.ncbi.nlm.nih.gov/projects/gap/cgi-bin/analysis.cgi?id=pha002522
Metabolic	Lipids	rhdl4b	Age-sex adj HDL Offsp ex4, ranked res	http://www.ncbi.nlm.nih.gov/projects/gap/cgi-bin/analysis.cgi?id=pha002523
Metabolic	Lipids	rhdl5b	Age-sex adj HDL Offsp ex5, ranked res	http://www.ncbi.nlm.nih.gov/projects/gap/cgi-bin/analysis.cgi?id=pha002524
Metabolic	Lipids	rhdl6b	Age-sex adj HDL Offsp ex6, ranked res	http://www.ncbi.nlm.nih.gov/projects/gap/cgi-bin/analysis.cgi?id=pha002525
Metabolic	Lipids	rhdl7b	Age-sex adj HDL Offsp ex7, ranked res	http://www.ncbi.nlm.nih.gov/projects/gap/cgi-bin/analysis.cgi?id=pha002526
Metabolic	Lipids	rHDLNMRlg4a	Multivariable adj NMR HDL Lg Offsp ex4 or TG > 400, ranked res	http://www.ncbi.nlm.nih.gov/projects/gap/cgi-bin/analysis.cgi?id=pha002527
Metabolic	Lipids	rHDLNMRlg4b	Age-sex adj NMR HDL Lg Offsp ex4 or TG > 400, ranked res	http://www.ncbi.nlm.nih.gov/projects/gap/cgi-bin/analysis.cgi?id=pha002528
Metabolic	Lipids	rHDLNMRsz4a	Multivariable adj NMR HDL Size Offsp ex4 or TG > 400, ranked res	http://www.ncbi.nlm.nih.gov/projects/gap/cgi-bin/analysis.cgi?id=pha002529
Metabolic	Lipids	rIDLNMR4a	Multivariable adj NMR IDL Offsp ex4 or TG > 400, ranked res	http://www.ncbi.nlm.nih.gov/projects/gap/cgi-bin/analysis.cgi?id=pha002530
Metabolic	Lipids	rIDLNMR4b	Age-sex adj NMR IDL Offsp ex4 or TG > 400, ranked res	http://www.ncbi.nlm.nih.gov/projects/gap/cgi-bin/analysis.cgi?id=pha002531
Metabolic	Lipids	rldl1a	Multivariable adj LDL Offsp ex1, ranked res	http://www.ncbi.nlm.nih.gov/projects/gap/cgi-bin/analysis.cgi?id=pha002532
Metabolic	Lipids	rldl1b	Age-sex adj LDL Offsp ex1, ranked res	http://www.ncbi.nlm.nih.gov/projects/gap/cgi-bin/analysis.cgi?id=pha002533
Metabolic	Lipids	rldl2b	Age-sex adj LDL Offsp ex2, ranked res	http://www.ncbi.nlm.nih.gov/projects/gap/cgi-bin/analysis.cgi?id=pha002534
Metabolic	Lipids	rldl4b	Age-sex adj LDL Offsp ex4, ranked res	http://www.ncbi.nlm.nih.gov/projects/gap/cgi-bin/analysis.cgi?id=pha002535
Metabolic	Lipids	rldl6b	Age-sex adj LDL Offsp ex6, ranked res	http://www.ncbi.nlm.nih.gov/projects/gap/cgi-bin/analysis.cgi?id=pha002536
Metabolic	Lipids	rldl7b	Age-sex adj LDL Offsp ex7, ranked res	http://www.ncbi.nlm.nih.gov/projects/gap/cgi-bin/analysis.cgi?id=pha002537
Metabolic	Lipids	rLDLNMRsm4a	Multivariable adj NMR LDL Sm Offsp ex4 , ranked res	http://www.ncbi.nlm.nih.gov/projects/gap/cgi-bin/analysis.cgi?id=pha002664
Metabolic	Lipids	rLDLNMRsm4b	Age-sex adj NMR LDL Sm Offsp ex4 , ranked res	http://www.ncbi.nlm.nih.gov/projects/gap/cgi-bin/analysis.cgi?id=pha002665
Metabolic	Lipids	rlpa3a	Multivariable adj Lpa Offsp ex3, ranked res	http://www.ncbi.nlm.nih.gov/projects/gap/cgi-bin/analysis.cgi?id=pha002617
Metabolic	Lipids	rlpa3b	Age-sex adj Lpa Offsp ex3, ranked res	http://www.ncbi.nlm.nih.gov/projects/gap/cgi-bin/analysis.cgi?id=pha002618
Metabolic	Lipids	RLPTG4a	Remnant LP TG Offsp ex4	http://www.ncbi.nlm.nih.gov/projects/gap/cgi-bin/analysis.cgi?id=pha002539
Metabolic	Lipids	RLPTG4b	Remnant LP Age-sex adj TG Offsp ex4	http://www.ncbi.nlm.nih.gov/projects/gap/cgi-bin/analysis.cgi?id=pha002538
Metabolic	Lipids	rmeancholb	Age-sex adj Mean Chol Offsp ex1-7, ranked res	http://www.ncbi.nlm.nih.gov/projects/gap/cgi-bin/analysis.cgi?id=pha002632
Metabolic	Lipids	rmeanhdla	Multivariable adj Mean HDL Offsp ex1-7, ranked res	http://www.ncbi.nlm.nih.gov/projects/gap/cgi-bin/analysis.cgi?id=pha002637
Metabolic	Lipids	rmeanhdlb	Age-sex adj Mean HDL Offsp ex1-7, ranked res	http://www.ncbi.nlm.nih.gov/projects/gap/cgi-bin/analysis.cgi?id=pha002638
Metabolic	Lipids	rmeanldla	Multivariable adj Mean LDL Offsp ex1-7, ranked res	http://www.ncbi.nlm.nih.gov/projects/gap/cgi-bin/analysis.cgi?id=pha002625

Group	Category	Trait Label	Name	Linkage Link
Metabolic	Lipids	rmeanldlb	Age-sex adj Mean LDL Offsp ex1-7, ranked res	http://www.ncbi.nlm.nih.gov/projects/gap/cgi-bin/analysis.cgi?id=pha002626
Metabolic	Lipids	rmeantga	Multivariable adj Mean TG Offsp ex1-7, ranked res	http://www.ncbi.nlm.nih.gov/projects/gap/cgi-bin/analysis.cgi?id=pha002619
Metabolic	Lipids	rmeantgb	Age-sex adj Mean TG Offsp ex1-7, ranked res	http://www.ncbi.nlm.nih.gov/projects/gap/cgi-bin/analysis.cgi?id=pha002620
Metabolic	Lipids	rPlasmaApoEa	Multivariable adj Plasma ApoE Level Offsp ex5, ranked res	http://www.ncbi.nlm.nih.gov/projects/gap/cgi-bin/analysis.cgi?id=pha002697
Metabolic	Lipids	rPlasmaApoEb	Age-sex adj Plasma ApoE Level Offsp ex5, ranked res	http://www.ncbi.nlm.nih.gov/projects/gap/cgi-bin/analysis.cgi?id=pha002698
Metabolic	Lipids	rRLPChol4a	Multivariable adj Remnant LP Chol Offsp ex4, ranked res	http://www.ncbi.nlm.nih.gov/projects/gap/cgi-bin/analysis.cgi?id=pha002699
Metabolic	Lipids	rRLPChol4b	Age-sex adj Remnant LP Chol Offsp ex4, ranked res	http://www.ncbi.nlm.nih.gov/projects/gap/cgi-bin/analysis.cgi?id=pha002700
Metabolic	Lipids	rtg1a	Multivariable adj TG Offsp ex1, ranked res	http://www.ncbi.nlm.nih.gov/projects/gap/cgi-bin/analysis.cgi?id=pha002540
Metabolic	Lipids	rtg1b	Age-sex adj TG Offsp ex1, ranked res	http://www.ncbi.nlm.nih.gov/projects/gap/cgi-bin/analysis.cgi?id=pha002541
Metabolic	Lipids	rtg2b	Age-sex adj TG Offsp ex2, ranked res	http://www.ncbi.nlm.nih.gov/projects/gap/cgi-bin/analysis.cgi?id=pha002542
Metabolic	Lipids	rtg3b	Age-sex adj TG Offsp ex3, ranked res	http://www.ncbi.nlm.nih.gov/projects/gap/cgi-bin/analysis.cgi?id=pha002543
Metabolic	Lipids	rtg5b	Age-sex adj TG Offsp ex5, ranked res	http://www.ncbi.nlm.nih.gov/projects/gap/cgi-bin/analysis.cgi?id=pha002545
Metabolic	Lipids	rtg6b	Age-sex adj TG Offsp ex6, ranked res	http://www.ncbi.nlm.nih.gov/projects/gap/cgi-bin/analysis.cgi?id=pha002546
Metabolic	Lipids	rtg7b	Age-sex adj TG Offsp ex7, ranked res	http://www.ncbi.nlm.nih.gov/projects/gap/cgi-bin/analysis.cgi?id=pha002547
Metabolic	Lipids	rtghdl1a	Multivariable adj TG-HDL Ratio Offsp ex1, ranked res	http://www.ncbi.nlm.nih.gov/projects/gap/cgi-bin/analysis.cgi?id=pha002564
Metabolic	Lipids	rVLDLNMRsz4a	Multivariable adj NMR VLDL Size Offsp ex4, ranked res	http://www.ncbi.nlm.nih.gov/projects/gap/cgi-bin/analysis.cgi?id=pha002680
Metabolic	Lipids	rVLDLNMRsz4b	Age-sex adj NMR VLDL Size Offsp ex4, ranked res	http://www.ncbi.nlm.nih.gov/projects/gap/cgi-bin/analysis.cgi?id=pha002681
Metabolic	Lipids	tg4b	Age-sex adj TG Offsp ex4	http://www.ncbi.nlm.nih.gov/projects/gap/cgi-bin/analysis.cgi?id=pha002544
Metabolic	Lipids	tghdl1b	Age-sex adj TG-HDL Ratio Offsp ex1	http://www.ncbi.nlm.nih.gov/projects/gap/cgi-bin/analysis.cgi?id=pha002692
Metabolic	Lipids	tghdl2b	Age-sex adj TG-HDL Ratio Offsp ex2	http://www.ncbi.nlm.nih.gov/projects/gap/cgi-bin/analysis.cgi?id=pha002565
Metabolic	Lipids	tghdl3b	Age-sex adj TG-HDL Ratio Offsp ex3	http://www.ncbi.nlm.nih.gov/projects/gap/cgi-bin/analysis.cgi?id=pha002566
Metabolic	Lipids	tghdl4b	Age-sex adj TG-HDL Ratio Offsp ex4	http://www.ncbi.nlm.nih.gov/projects/gap/cgi-bin/analysis.cgi?id=pha002567
Metabolic	Lipids	tghdl5b	Age-sex adj TG-HDL Ratio Offsp ex5	http://www.ncbi.nlm.nih.gov/projects/gap/cgi-bin/analysis.cgi?id=pha002568
Metabolic	Lipids	tghdl6b	Age-sex adj TG-HDL Ratio Offsp ex6	http://www.ncbi.nlm.nih.gov/projects/gap/cgi-bin/analysis.cgi?id=pha002569
Metabolic	Lipids	tghdl7b	Age-sex adj TG-HDL Ratio Offsp ex7	http://www.ncbi.nlm.nih.gov/projects/gap/cgi-bin/analysis.cgi?id=pha002570
Metabolic	Lipids	VLDLNMRint4a	Multivariable adj NMR VLDL Int Offsp ex4	http://www.ncbi.nlm.nih.gov/projects/gap/cgi-bin/analysis.cgi?id=pha002670
Metabolic	Lipids	VLDLNMRint4b	Age-sex adj NMR VLDL Int Offsp ex4	http://www.ncbi.nlm.nih.gov/projects/gap/cgi-bin/analysis.cgi?id=pha002671
Metabolic	Lipids	VLDLNMRlg4a	Multivariable adj NMR VLDL Lg Offsp ex4	http://www.ncbi.nlm.nih.gov/projects/gap/cgi-bin/analysis.cgi?id=pha002674
Metabolic	Lipids	VLDLNMRlg4b	Age-sex adj NMR VLDL Lg Offsp ex4	http://www.ncbi.nlm.nih.gov/projects/gap/cgi-bin/analysis.cgi?id=pha002675
Metabolic	Lipids	VLDLNMRsm4a	Multivariable adj NMR VLDL Sm Offsp ex4	http://www.ncbi.nlm.nih.gov/projects/gap/cgi-bin/analysis.cgi?id=pha002686
Metabolic	Lipids	VLDLNMRsm4b	Age-sex adj NMR VLDL Sm Offsp ex4	http://www.ncbi.nlm.nih.gov/projects/gap/cgi-bin/analysis.cgi?id=pha002687
Metabolic	Lipidsmen	menHDLNMRint4b	Age-sex adj NMR HDL Int Offsp men ex4	http://www.ncbi.nlm.nih.gov/projects/gap/cgi-bin/analysis.cgi?id=pha002642
Metabolic	Lipidsmen	menHDLNMRsm4a	Multivariable adj NMR HDL Sm Offsp men ex4	http://www.ncbi.nlm.nih.gov/projects/gap/cgi-bin/analysis.cgi?id=pha002652
Metabolic	Lipidsmen	menHDLNMRsm4b	Age-sex adj NMR HDL Sm Offsp men ex4	http://www.ncbi.nlm.nih.gov/projects/gap/cgi-bin/analysis.cgi?id=pha002653
Metabolic	Lipidsmen	menHDLNMRsz4b	Age-sex adj NMR HDL Size Offsp men ex4	http://www.ncbi.nlm.nih.gov/projects/gap/cgi-bin/analysis.cgi?id=pha002648
Metabolic	Lipidsmen	menldl3b	Age-sex adj LDL Offsp men ex3	http://www.ncbi.nlm.nih.gov/projects/gap/cgi-bin/analysis.cgi?id=pha002573
Metabolic	Lipidsmen	menldl5b	Age-sex adj LDL Offsp men ex5	http://www.ncbi.nlm.nih.gov/projects/gap/cgi-bin/analysis.cgi?id=pha002575
Metabolic	Lipidsmen	menLDLNMRlg4a	Multivariable adj NMR LDL Lg Offsp men ex4	http://www.ncbi.nlm.nih.gov/projects/gap/cgi-bin/analysis.cgi?id=pha002656
Metabolic	Lipidsmen	menLDLNMRlg4b	Age-sex adj NMR LDL Lg Offsp men ex4	http://www.ncbi.nlm.nih.gov/projects/gap/cgi-bin/analysis.cgi?id=pha002657
Metabolic	Lipidsmen	menLDLNMRsz4a	Multivariable adj NMR LDL Size Offsp men ex4	http://www.ncbi.nlm.nih.gov/projects/gap/cgi-bin/analysis.cgi?id=pha002660
Metabolic	Lipidsmen	menLDLNMRsz4b	Age-sex adj NMR LDL Size Offsp men ex4	http://www.ncbi.nlm.nih.gov/projects/gap/cgi-bin/analysis.cgi?id=pha002661
Metabolic	Lipidsmen	menrApoB4a	Multivariable adj Plasma ApoB Level Offsp men ex4, ranked res	http://www.ncbi.nlm.nih.gov/projects/gap/cgi-bin/analysis.cgi?id=pha002693
Metabolic	Lipidsmen	menrApoB4b	Age-sex adj Plasma ApoB Level Offsp men ex4, ranked res	http://www.ncbi.nlm.nih.gov/projects/gap/cgi-bin/analysis.cgi?id=pha002694
Metabolic	Lipidsmen	menrchol1a	Multivariable adj Chol Offsp men ex1, ranked res	http://www.ncbi.nlm.nih.gov/projects/gap/cgi-bin/analysis.cgi?id=pha002585
Metabolic	Lipidsmen	menrchol1b	Age-sex adj Chol Offsp men ex1, ranked res	http://www.ncbi.nlm.nih.gov/projects/gap/cgi-bin/analysis.cgi?id=pha002586
Metabolic	Lipidsmen	menrchol2b	Age-sex adj Chol Offsp men ex2, ranked res	http://www.ncbi.nlm.nih.gov/projects/gap/cgi-bin/analysis.cgi?id=pha002587
Metabolic	Lipidsmen	menrchol3b	Age-sex adj Chol Offsp men ex3, ranked res	http://www.ncbi.nlm.nih.gov/projects/gap/cgi-bin/analysis.cgi?id=pha002588
Metabolic	Lipidsmen	menrchol5b	Age-sex adj Chol Offsp men ex5, ranked res	http://www.ncbi.nlm.nih.gov/projects/gap/cgi-bin/analysis.cgi?id=pha002589
Metabolic	Lipidsmen	menrchol6b	Age-sex adj Chol Offsp men ex6, ranked res	http://www.ncbi.nlm.nih.gov/projects/gap/cgi-bin/analysis.cgi?id=pha002590
Metabolic	Lipidsmen	menrchol7b	Age-sex adj Chol Offsp men ex7, ranked res	http://www.ncbi.nlm.nih.gov/projects/gap/cgi-bin/analysis.cgi?id=pha002591
Metabolic	Lipidsmen	menrhdl1a	Multivariable adj HDL Offsp men ex1, ranked res	http://www.ncbi.nlm.nih.gov/projects/gap/cgi-bin/analysis.cgi?id=pha002600
Metabolic	Lipidsmen	menrhdl1b	Age-sex adj HDL Offsp men ex1, ranked res	http://www.ncbi.nlm.nih.gov/projects/gap/cgi-bin/analysis.cgi?id=pha002601
Metabolic	Lipidsmen	menrhdl2b	Age-sex adj HDL Offsp men ex2, ranked res	http://www.ncbi.nlm.nih.gov/projects/gap/cgi-bin/analysis.cgi?id=pha002602
Metabolic	Lipidsmen	menrhdl3b	Age-sex adj HDL Offsp men ex3, ranked res	http://www.ncbi.nlm.nih.gov/projects/gap/cgi-bin/analysis.cgi?id=pha002603
Metabolic	Lipidsmen	menrhdl4b	Age-sex adj HDL Offsp men ex4, ranked res	http://www.ncbi.nlm.nih.gov/projects/gap/cgi-bin/analysis.cgi?id=pha002604
Metabolic	Lipidsmen	menrhdl5b	Age-sex adj HDL Offsp men ex5, ranked res	http://www.ncbi.nlm.nih.gov/projects/gap/cgi-bin/analysis.cgi?id=pha002605

Group	Category	Trait Label	Name	Linkage Link
Metabolic	Lipidsmen	menrhdl7b	Age-sex adj HDL Offsp men ex7, ranked res	http://www.ncbi.nlm.nih.gov/projects/gap/cgi-bin/analysis.cgi?id=pha002606
Metabolic	Lipidsmen	menrHDLNMRlg4a	Multivariable adj NMR HDL Lg Offsp men ex4 , ranked res	http://www.ncbi.nlm.nih.gov/projects/gap/cgi-bin/analysis.cgi?id=pha002645
Metabolic	Lipidsmen	menrHDLNMRsz4a	Multivariable adj NMR HDL Size Offsp men ex4 , ranked res	http://www.ncbi.nlm.nih.gov/projects/gap/cgi-bin/analysis.cgi?id=pha002649
Metabolic	Lipidsmen	menrldl1a	Multivariable adj LDL Offsp men ex1, ranked res	http://www.ncbi.nlm.nih.gov/projects/gap/cgi-bin/analysis.cgi?id=pha002571
Metabolic	Lipidsmen	menrldl1b	Age-sex adj LDL Offsp men ex1, ranked res	http://www.ncbi.nlm.nih.gov/projects/gap/cgi-bin/analysis.cgi?id=pha002615
Metabolic	Lipidsmen	menrldl2b	Age-sex adj LDL Offsp men ex2, ranked res	http://www.ncbi.nlm.nih.gov/projects/gap/cgi-bin/analysis.cgi?id=pha002572
Metabolic	Lipidsmen	menrldl4b	Age-sex adj LDL Offsp men ex4, ranked res	http://www.ncbi.nlm.nih.gov/projects/gap/cgi-bin/analysis.cgi?id=pha002574
Metabolic	Lipidsmen	menrldl6b	Age-sex adj LDL Offsp men ex6, ranked res	http://www.ncbi.nlm.nih.gov/projects/gap/cgi-bin/analysis.cgi?id=pha002576
Metabolic	Lipidsmen	menrldl7b	Age-sex adj LDL Offsp men ex7, ranked res	http://www.ncbi.nlm.nih.gov/projects/gap/cgi-bin/analysis.cgi?id=pha002577
Metabolic	Lipidsmen	menrLDLNMRsm4a	Multivariable adj NMR LDL Sm Offsp men ex4 , ranked res	http://www.ncbi.nlm.nih.gov/projects/gap/cgi-bin/analysis.cgi?id=pha002666
Metabolic	Lipidsmen	menrLDLNMRsm4b	Age-sex adj NMR LDL Sm Offsp men ex4 , ranked res	http://www.ncbi.nlm.nih.gov/projects/gap/cgi-bin/analysis.cgi?id=pha002667
Metabolic	Lipidsmen	menrmeanchola	Multivariable adj Mean Chol Offsp men ex1-7, ranked res	http://www.ncbi.nlm.nih.gov/projects/gap/cgi-bin/analysis.cgi?id=pha002633
Metabolic	Lipidsmen	menrmeancholb	Age-sex adj Mean Chol Offsp men ex1-7, ranked res	http://www.ncbi.nlm.nih.gov/projects/gap/cgi-bin/analysis.cgi?id=pha002634
Metabolic	Lipidsmen	menrmeanhdlb	Age-sex adj Mean HDL Offsp men ex1-7, ranked res	http://www.ncbi.nlm.nih.gov/projects/gap/cgi-bin/analysis.cgi?id=pha002639
Metabolic	Lipidsmen	menrmeanldla	Multivariable adj Mean LDL Offsp men ex1-7, ranked res	http://www.ncbi.nlm.nih.gov/projects/gap/cgi-bin/analysis.cgi?id=pha002627
Metabolic	Lipidsmen	menrmeanldlb	Age-sex adj Mean LDL Offsp men ex1-7, ranked res	http://www.ncbi.nlm.nih.gov/projects/gap/cgi-bin/analysis.cgi?id=pha002628
Metabolic	Lipidsmen	menrmeantga	Multivariable adj Mean TG Offsp men ex1-7, ranked res	http://www.ncbi.nlm.nih.gov/projects/gap/cgi-bin/analysis.cgi?id=pha002621
Metabolic	Lipidsmen	menrmeantgb	Age-sex adj Mean TG Offsp men ex1-7, ranked res	http://www.ncbi.nlm.nih.gov/projects/gap/cgi-bin/analysis.cgi?id=pha002622
Metabolic	Lipidsmen	menrtg1a	Multivariable adj TG Offsp men ex1, ranked res	http://www.ncbi.nlm.nih.gov/projects/gap/cgi-bin/analysis.cgi?id=pha002548
Metabolic	Lipidsmen	menrtg1b	Age-sex adj TG Offsp men ex1, ranked res	http://www.ncbi.nlm.nih.gov/projects/gap/cgi-bin/analysis.cgi?id=pha002549
Metabolic	Lipidsmen	menrtg2b	Age-sex adj TG Offsp men ex2, ranked res	http://www.ncbi.nlm.nih.gov/projects/gap/cgi-bin/analysis.cgi?id=pha002550
Metabolic	Lipidsmen	menrtg3b	Age-sex adj TG Offsp men ex3, ranked res	http://www.ncbi.nlm.nih.gov/projects/gap/cgi-bin/analysis.cgi?id=pha002551
Metabolic	Lipidsmen	menrtg5b	Age-sex adj TG Offsp men ex5, ranked res	http://www.ncbi.nlm.nih.gov/projects/gap/cgi-bin/analysis.cgi?id=pha002553
Metabolic	Lipidsmen	menrtg6b	Age-sex adj TG Offsp men ex6, ranked res	http://www.ncbi.nlm.nih.gov/projects/gap/cgi-bin/analysis.cgi?id=pha002554
Metabolic	Lipidsmen	menrtg7b	Age-sex adj TG Offsp men ex7, ranked res	http://www.ncbi.nlm.nih.gov/projects/gap/cgi-bin/analysis.cgi?id=pha002555
Metabolic	Lipidsmen	menrVLDLNMRsz4a	Multivariable adj NMR VLDL Size Offsp men ex4, ranked res	http://www.ncbi.nlm.nih.gov/projects/gap/cgi-bin/analysis.cgi?id=pha002682
Metabolic	Lipidsmen	menrVLDLNMRsz4b	Age-sex adj NMR VLDL Size Offsp men ex4, ranked res	http://www.ncbi.nlm.nih.gov/projects/gap/cgi-bin/analysis.cgi?id=pha002683
Metabolic	Lipidsmen	mentg4b	Age-sex adj TG Offsp men ex4	http://www.ncbi.nlm.nih.gov/projects/gap/cgi-bin/analysis.cgi?id=pha002552
Metabolic	Lipidsmen	menVLDLNMRint4a	Multivariable adj NMR VLDL Int Offsp men ex4	http://www.ncbi.nlm.nih.gov/projects/gap/cgi-bin/analysis.cgi?id=pha002672
Metabolic	Lipidsmen	menVLDLNMRlg4a	Multivariable adj NMR VLDL Lg Offsp men ex4	http://www.ncbi.nlm.nih.gov/projects/gap/cgi-bin/analysis.cgi?id=pha002676
Metabolic	Lipidsmen	menVLDLNMRlg4b	Age-sex adj NMR VLDL Lg Offsp men ex4	http://www.ncbi.nlm.nih.gov/projects/gap/cgi-bin/analysis.cgi?id=pha002677
Metabolic	Lipidsmen	menVLDLNMRsm4a	Multivariable adj NMR VLDL Sm Offsp men ex4	http://www.ncbi.nlm.nih.gov/projects/gap/cgi-bin/analysis.cgi?id=pha002688
Metabolic	Lipidsmen	menVLDLNMRsm4b	Age-sex adj NMR VLDL Sm Offsp men ex4	http://www.ncbi.nlm.nih.gov/projects/gap/cgi-bin/analysis.cgi?id=pha002689
Metabolic	Lipidswomen	rmeanchola	Multivariable adj Mean Chol Offsp ex1-7, ranked res	http://www.ncbi.nlm.nih.gov/projects/gap/cgi-bin/analysis.cgi?id=pha002631
Metabolic	Lipidswomen	womenHDLNMRint4a	Multivariable adj NMR HDL Int Offsp wom ex4	http://www.ncbi.nlm.nih.gov/projects/gap/cgi-bin/analysis.cgi?id=pha002643
Metabolic	Lipidswomen	womenHDLNMRint4b	Age-sex adj NMR HDL Int Offsp wom ex4	http://www.ncbi.nlm.nih.gov/projects/gap/cgi-bin/analysis.cgi?id=pha002644
Metabolic	Lipidswomen	womenHDLNMRsm4a	Multivariable adj NMR HDL Sm Offsp wom ex4	http://www.ncbi.nlm.nih.gov/projects/gap/cgi-bin/analysis.cgi?id=pha002654
Metabolic	Lipidswomen	womenHDLNMRsm4b	Age-sex adj NMR HDL Sm Offsp wom ex4	http://www.ncbi.nlm.nih.gov/projects/gap/cgi-bin/analysis.cgi?id=pha002655
Metabolic	Lipidswomen	womenHDLNMRsz4b	Age-sex adj NMR HDL Size Offsp wom ex4	http://www.ncbi.nlm.nih.gov/projects/gap/cgi-bin/analysis.cgi?id=pha002650
Metabolic	Lipidswomen	womenldl3b	Age-sex adj LDL Offsp wom ex3	http://www.ncbi.nlm.nih.gov/projects/gap/cgi-bin/analysis.cgi?id=pha002580
Metabolic	Lipidswomen	womenldl5b	Age-sex adj LDL Offsp wom ex5	http://www.ncbi.nlm.nih.gov/projects/gap/cgi-bin/analysis.cgi?id=pha002582
Metabolic	Lipidswomen	womenLDLNMRlg4a	Multivariable adj NMR LDL Lg Offsp wom ex4	http://www.ncbi.nlm.nih.gov/projects/gap/cgi-bin/analysis.cgi?id=pha002658
Metabolic	Lipidswomen	womenLDLNMRlg4b	Age-sex adj NMR LDL Lg Offsp wom ex4	http://www.ncbi.nlm.nih.gov/projects/gap/cgi-bin/analysis.cgi?id=pha002659
Metabolic	Lipidswomen	womenLDLNMRsz4a	Multivariable adj NMR LDL Size Offsp wom ex4	http://www.ncbi.nlm.nih.gov/projects/gap/cgi-bin/analysis.cgi?id=pha002662
Metabolic	Lipidswomen	womenLDLNMRsz4b	Age-sex adj NMR LDL Size Offsp wom ex4	http://www.ncbi.nlm.nih.gov/projects/gap/cgi-bin/analysis.cgi?id=pha002663
Metabolic	Lipidswomen	womenrApoB4a	Multivariable adj Plasma ApoB Level Offsp wom ex4, ranked res	http://www.ncbi.nlm.nih.gov/projects/gap/cgi-bin/analysis.cgi?id=pha002695
Metabolic	Lipidswomen	womenrApoB4b	Age-sex adj Plasma ApoB Level Offsp wom ex4, ranked res	http://www.ncbi.nlm.nih.gov/projects/gap/cgi-bin/analysis.cgi?id=pha002696
Metabolic	Lipidswomen	womenrchol1a	Multivariable adj Chol Offsp wom ex1, ranked res	http://www.ncbi.nlm.nih.gov/projects/gap/cgi-bin/analysis.cgi?id=pha002592
Metabolic	Lipidswomen	womenrchol1b	Age-sex adj Chol Offsp wom ex1, ranked res	http://www.ncbi.nlm.nih.gov/projects/gap/cgi-bin/analysis.cgi?id=pha002593
Metabolic	Lipidswomen	womenrchol2b	Age-sex adj Chol Offsp wom ex2, ranked res	http://www.ncbi.nlm.nih.gov/projects/gap/cgi-bin/analysis.cgi?id=pha002594
Metabolic	Lipidswomen	womenrchol3b	Age-sex adj Chol Offsp wom ex3, ranked res	http://www.ncbi.nlm.nih.gov/projects/gap/cgi-bin/analysis.cgi?id=pha002595
Metabolic	Lipidswomen	womenrchol4b	Age-sex adj Chol Offsp wom ex4, ranked res	http://www.ncbi.nlm.nih.gov/projects/gap/cgi-bin/analysis.cgi?id=pha002596
Metabolic	Lipidswomen	womenrchol5b	Age-sex adj Chol Offsp wom ex5, ranked res	http://www.ncbi.nlm.nih.gov/projects/gap/cgi-bin/analysis.cgi?id=pha002597
Metabolic	Lipidswomen	womenrchol6b	Age-sex adj Chol Offsp wom ex6, ranked res	http://www.ncbi.nlm.nih.gov/projects/gap/cgi-bin/analysis.cgi?id=pha002598
Metabolic	Lipidswomen	womenrchol7b	Age-sex adj Chol Offsp wom ex7, ranked res	http://www.ncbi.nlm.nih.gov/projects/gap/cgi-bin/analysis.cgi?id=pha002599
Metabolic	Lipidswomen	womenrhdl1a	Multivariable adj HDL Offsp wom ex1, ranked res	http://www.ncbi.nlm.nih.gov/projects/gap/cgi-bin/analysis.cgi?id=pha002607

Group	Category	Trait Label	Name	Linkage Link
Metabolic	Lipidswomen	womenrhdl1b	Age-sex adj HDL Offsp wom ex1, ranked res	http://www.ncbi.nlm.nih.gov/projects/gap/cgi-bin/analysis.cgi?id=pha002608
Metabolic	Lipidswomen	womenrhdl2b	Age-sex adj HDL Offsp wom ex2, ranked res	http://www.ncbi.nlm.nih.gov/projects/gap/cgi-bin/analysis.cgi?id=pha002609
Metabolic	Lipidswomen	womenrhdl3b	Age-sex adj HDL Offsp wom ex3, ranked res	http://www.ncbi.nlm.nih.gov/projects/gap/cgi-bin/analysis.cgi?id=pha002610
Metabolic	Lipidswomen	womenrhdl4b	Age-sex adj HDL Offsp wom ex4, ranked res	http://www.ncbi.nlm.nih.gov/projects/gap/cgi-bin/analysis.cgi?id=pha002611
Metabolic	Lipidswomen	womenrhdl5b	Age-sex adj HDL Offsp wom ex5, ranked res	http://www.ncbi.nlm.nih.gov/projects/gap/cgi-bin/analysis.cgi?id=pha002612
Metabolic	Lipidswomen	womenrhdl6b	Age-sex adj HDL Offsp wom ex6, ranked res	http://www.ncbi.nlm.nih.gov/projects/gap/cgi-bin/analysis.cgi?id=pha002613
Metabolic	Lipidswomen	womenrhdl7b	Age-sex adj HDL Offsp wom ex7, ranked res	http://www.ncbi.nlm.nih.gov/projects/gap/cgi-bin/analysis.cgi?id=pha002614
Metabolic	Lipidswomen	womenrHDLNMRlg4a	Multivariable adj NMR HDL Lg Offsp wom ex4 , ranked res	http://www.ncbi.nlm.nih.gov/projects/gap/cgi-bin/analysis.cgi?id=pha002646
Metabolic	Lipidswomen	womenrHDLNMRlg4b	Age-sex adj NMR HDL Lg Offsp wom ex4 , ranked res	http://www.ncbi.nlm.nih.gov/projects/gap/cgi-bin/analysis.cgi?id=pha002647
Metabolic	Lipidswomen	womenrHDLNMRsz4a	Multivariable adj NMR HDL Size Offsp wom ex4 , ranked res	http://www.ncbi.nlm.nih.gov/projects/gap/cgi-bin/analysis.cgi?id=pha002651
Metabolic	Lipidswomen	womenrldl1a	Multivariable adj LDL Offsp wom ex1, ranked res	http://www.ncbi.nlm.nih.gov/projects/gap/cgi-bin/analysis.cgi?id=pha002578
Metabolic	Lipidswomen	womenrldl1b	Age-sex adj LDL Offsp wom ex1, ranked res	http://www.ncbi.nlm.nih.gov/projects/gap/cgi-bin/analysis.cgi?id=pha002616
Metabolic	Lipidswomen	womenrldl2b	Age-sex adj LDL Offsp wom ex2, ranked res	http://www.ncbi.nlm.nih.gov/projects/gap/cgi-bin/analysis.cgi?id=pha002579
Metabolic	Lipidswomen	womenrldl4b	Age-sex adj LDL Offsp wom ex4, ranked res	http://www.ncbi.nlm.nih.gov/projects/gap/cgi-bin/analysis.cgi?id=pha002581
Metabolic	Lipidswomen	womenrldl6b	Age-sex adj LDL Offsp wom ex6, ranked res	http://www.ncbi.nlm.nih.gov/projects/gap/cgi-bin/analysis.cgi?id=pha002583
Metabolic	Lipidswomen	womenrldl7b	Age-sex adj LDL Offsp wom ex7, ranked res	http://www.ncbi.nlm.nih.gov/projects/gap/cgi-bin/analysis.cgi?id=pha002584
Metabolic	Lipidswomen	womenrLDLNMRsm4a	Multivariable adj NMR LDL Sm Offsp wom ex4 , ranked res	http://www.ncbi.nlm.nih.gov/projects/gap/cgi-bin/analysis.cgi?id=pha002668
Metabolic	Lipidswomen	womenrLDLNMRsm4b	Age-sex adj NMR LDL Sm Offsp wom ex4 , ranked res	http://www.ncbi.nlm.nih.gov/projects/gap/cgi-bin/analysis.cgi?id=pha002669
Metabolic	Lipidswomen	womenrmeanchola	Multivariable adj Mean Chol Offsp wom ex1-7, ranked res	http://www.ncbi.nlm.nih.gov/projects/gap/cgi-bin/analysis.cgi?id=pha002635
Metabolic	Lipidswomen	womenrmeancholb	Age-sex adj Mean Chol Offsp wom ex1-7, ranked res	http://www.ncbi.nlm.nih.gov/projects/gap/cgi-bin/analysis.cgi?id=pha002636
Metabolic	Lipidswomen	womenrmeanhdla	Multivariable adj Mean HDL Offsp wom ex1-7, ranked res	http://www.ncbi.nlm.nih.gov/projects/gap/cgi-bin/analysis.cgi?id=pha002640
Metabolic	Lipidswomen	womenrmeanhdlb	Age-sex Mean HDL Offsp wom ex1-7, ranked res	http://www.ncbi.nlm.nih.gov/projects/gap/cgi-bin/analysis.cgi?id=pha002641
Metabolic	Lipidswomen	womenrmeanldla	Multivariable adj Mean LDL Offsp wom ex1-7, ranked res	http://www.ncbi.nlm.nih.gov/projects/gap/cgi-bin/analysis.cgi?id=pha002629
Metabolic	Lipidswomen	womenrmeanldlb	Age-sex adj Mean LDL Offsp wom ex1-7, ranked res	http://www.ncbi.nlm.nih.gov/projects/gap/cgi-bin/analysis.cgi?id=pha002630
Metabolic	Lipidswomen	womenrmeantga	Multivariable adj Mean TG Offsp wom ex1-7, ranked res	http://www.ncbi.nlm.nih.gov/projects/gap/cgi-bin/analysis.cgi?id=pha002623
Metabolic	Lipidswomen	womenrmeantgb	Age-sex adj Mean TG Offsp wom ex1-7, ranked res	http://www.ncbi.nlm.nih.gov/projects/gap/cgi-bin/analysis.cgi?id=pha002624
Metabolic	Lipidswomen	womenrtg1a	Multivariable adj TG Offsp wom ex1, ranked res	http://www.ncbi.nlm.nih.gov/projects/gap/cgi-bin/analysis.cgi?id=pha002556
Metabolic	Lipidswomen	womenrtg1b	Age-sex adj TG Offsp wom ex1, ranked res	http://www.ncbi.nlm.nih.gov/projects/gap/cgi-bin/analysis.cgi?id=pha002557
Metabolic	Lipidswomen	womenrtg2b	Age-sex adj TG Offsp wom ex2, ranked res	http://www.ncbi.nlm.nih.gov/projects/gap/cgi-bin/analysis.cgi?id=pha002558
Metabolic	Lipidswomen	womenrtg3b	Age-sex adj TG Offsp wom ex3, ranked res	http://www.ncbi.nlm.nih.gov/projects/gap/cgi-bin/analysis.cgi?id=pha002559
Metabolic	Lipidswomen	womenrtg5b	Age-sex adj TG Offsp wom ex5, ranked res	http://www.ncbi.nlm.nih.gov/projects/gap/cgi-bin/analysis.cgi?id=pha002561
Metabolic	Lipidswomen	womenrtg6b	Age-sex adj TG Offsp wom ex6, ranked res	http://www.ncbi.nlm.nih.gov/projects/gap/cgi-bin/analysis.cgi?id=pha002562
Metabolic	Lipidswomen	womenrtg7b	Age-sex adj TG Offsp wom ex7, ranked res	http://www.ncbi.nlm.nih.gov/projects/gap/cgi-bin/analysis.cgi?id=pha002563
Metabolic	Lipidswomen	womenrVLDLNMRsz4a	Multivariable adj NMR VLDL Size Offsp wom ex4, ranked res	http://www.ncbi.nlm.nih.gov/projects/gap/cgi-bin/analysis.cgi?id=pha002684
Metabolic	Lipidswomen	womenrVLDLNMRsz4b	Age-sex adj NMR VLDL Size Offsp wom ex4, ranked res	http://www.ncbi.nlm.nih.gov/projects/gap/cgi-bin/analysis.cgi?id=pha002685
Metabolic	Lipidswomen	womentg4b	Age-sex adj TG Offsp wom ex4	http://www.ncbi.nlm.nih.gov/projects/gap/cgi-bin/analysis.cgi?id=pha002560
Metabolic	Lipidswomen	womenVLDLNMRint4a	Multivariable adj NMR VLDL Int Offsp wom ex4	http://www.ncbi.nlm.nih.gov/projects/gap/cgi-bin/analysis.cgi?id=pha002673
Metabolic	Lipidswomen	womenVLDLNMRlg4a	Multivariable adj NMR VLDL Lg Offsp wom ex4	http://www.ncbi.nlm.nih.gov/projects/gap/cgi-bin/analysis.cgi?id=pha002678
Metabolic	Lipidswomen	womenVLDLNMRlg4b	Age-sex adj NMR VLDL Lg Offsp wom ex4	http://www.ncbi.nlm.nih.gov/projects/gap/cgi-bin/analysis.cgi?id=pha002679
Metabolic	Lipidswomen	womenVLDLNMRsm4a	Multivariable adj NMR VLDL Sm Offsp wom ex4	http://www.ncbi.nlm.nih.gov/projects/gap/cgi-bin/analysis.cgi?id=pha002690
Metabolic	Lipidswomen	womenVLDLNMRsm4b	Age-sex NMR VLDL Sm Offsp wom ex4	http://www.ncbi.nlm.nih.gov/projects/gap/cgi-bin/analysis.cgi?id=pha002691
Pulmonary	Circadian	bedtime	usual weekday bedtime unadjusted	http://www.ncbi.nlm.nih.gov/projects/gap/cgi-bin/analysis.cgi?id=pha002714
Pulmonary	Circadian	bedtimeresid	usual weekday bedtime adjusted	http://www.ncbi.nlm.nih.gov/projects/gap/cgi-bin/analysis.cgi?id=pha002715
Pulmonary	Circadian	sleepdur	usual weekday sleep duration unadjusted	http://www.ncbi.nlm.nih.gov/projects/gap/cgi-bin/analysis.cgi?id=pha002716
Pulmonary	Circadian	sleepdurresid	usual weekday sleep duration adjusted	http://www.ncbi.nlm.nih.gov/projects/gap/cgi-bin/analysis.cgi?id=pha002717
Pulmonary	longitudinal	fef2575longsmoke10w	rate of decline of FEF(25-75) in 10+ packyear smokers, linkage	http://www.ncbi.nlm.nih.gov/projects/gap/cgi-bin/analysis.cgi?id=pha002718
Pulmonary	longitudinal	fef2575longw	rate of decline of FEF(25-75), linkage	http://www.ncbi.nlm.nih.gov/projects/gap/cgi-bin/analysis.cgi?id=pha002719
Pulmonary	longitudinal	feffvclongw	rate of decline of FEF(25-75)/FVC, linkage	http://www.ncbi.nlm.nih.gov/projects/gap/cgi-bin/analysis.cgi?id=pha002720
Pulmonary	longitudinal	fev1fvclongsmoke10w	rate of decline of FEV1/FVC in 10+ packyear smokers, linkage	http://www.ncbi.nlm.nih.gov/projects/gap/cgi-bin/analysis.cgi?id=pha002721
Pulmonary	longitudinal	fev1fvclongw	rate of decline of FEV1/FVC, linkage	http://www.ncbi.nlm.nih.gov/projects/gap/cgi-bin/analysis.cgi?id=pha002722
Pulmonary	longitudinal	fev1longw	rate of decline of FEV1, linkage	http://www.ncbi.nlm.nih.gov/projects/gap/cgi-bin/analysis.cgi?id=pha002723
Pulmonary	longitudinal	fvclongw	rate of decline of FVC, linkage	http://www.ncbi.nlm.nih.gov/projects/gap/cgi-bin/analysis.cgi?id=pha002724
Pulmonary	mean	genomefev1	mean FEV1 from 2 examinations	http://www.ncbi.nlm.nih.gov/projects/gap/cgi-bin/analysis.cgi?id=pha002725
Pulmonary	mean	genomefvc	mean FVC from 2 examinations	http://www.ncbi.nlm.nih.gov/projects/gap/cgi-bin/analysis.cgi?id=pha002726
Pulmonary	mean	genomeratio	mean FEV1/FVC from 2 examinations	http://www.ncbi.nlm.nih.gov/projects/gap/cgi-bin/analysis.cgi?id=pha002727
Pulmonary	ppcrosssectional	ppfefadj	percent predicted FEF(25-75) at latest exam	http://www.ncbi.nlm.nih.gov/projects/gap/cgi-bin/analysis.cgi?id=pha002728

Group	Category	Trait Label	Name	Linkage Link
Pulmonary	ppcrosssectional	ppfefadjsm10	percent predicted FEF(25-75) at latest exam in 10+ packyear smokers	http://www.ncbi.nlm.nih.gov/projects/gap/cgi-bin/analysis.cgi?id=pha002729
Pulmonary	ppcrosssectional	ppfefratadjsm10w	percent predicted FEF(25-75)/FVC latest exam, 10+ packyear smokers, linkage	http://www.ncbi.nlm.nih.gov/projects/gap/cgi-bin/analysis.cgi?id=pha002730
Pulmonary	ppcrosssectional	ppfefratadjw	percent predicted FEF(25-75)/FVC at latest exam, linkage	http://www.ncbi.nlm.nih.gov/projects/gap/cgi-bin/analysis.cgi?id=pha002731
Pulmonary	ppcrosssectional	ppfev1adj	percent predicted FEV1 at latest exam	http://www.ncbi.nlm.nih.gov/projects/gap/cgi-bin/analysis.cgi?id=pha002732
Pulmonary	ppcrosssectional	ppfev1adjsm10	percent predicted FEV1 at latest exam in 10+ packyear smokers	http://www.ncbi.nlm.nih.gov/projects/gap/cgi-bin/analysis.cgi?id=pha002733
Pulmonary	ppcrosssectional	ppfvcadj	percent predicted FVC at latest exam	http://www.ncbi.nlm.nih.gov/projects/gap/cgi-bin/analysis.cgi?id=pha002734
Pulmonary	ppcrosssectional	ppfvcadjsm10	percent predicted FVC at latest exam in 10+ packyear smokers	http://www.ncbi.nlm.nih.gov/projects/gap/cgi-bin/analysis.cgi?id=pha002735
Pulmonary	ppcrosssectional	ppratioadjsm10	percent predicted FEV1/FVC at latest exam in 10+ packyear smokers	http://www.ncbi.nlm.nih.gov/projects/gap/cgi-bin/analysis.cgi?id=pha002736
Pulmonary	ppcrosssectional	ppratioadjw	percent predicted FEV1/FVC at latest exam, linkage	http://www.ncbi.nlm.nih.gov/projects/gap/cgi-bin/analysis.cgi?id=pha002737
Pulmonary	rcrosssectional	rfefadj	residual from predicted FEF(25-75) at latest exam	http://www.ncbi.nlm.nih.gov/projects/gap/cgi-bin/analysis.cgi?id=pha002738
Pulmonary	rcrosssectional	rfefadjsm10	residual from predicted FEF(25-75) at latest exam in 10+ packyear smokers	http://www.ncbi.nlm.nih.gov/projects/gap/cgi-bin/analysis.cgi?id=pha002739
Pulmonary	rcrosssectional	rfefratadj	residual from predicted FEF(25-75)/FVC at latest exam	http://www.ncbi.nlm.nih.gov/projects/gap/cgi-bin/analysis.cgi?id=pha002740
Pulmonary	rcrosssectional	rfefratadjsm10	residual from predicted FEF(25-75)/FVC latest exam in 10+ packyear smokers	http://www.ncbi.nlm.nih.gov/projects/gap/cgi-bin/analysis.cgi?id=pha002741
Pulmonary	rcrosssectional	rfev1adj	residual from predicted FEV1 at latest exam	http://www.ncbi.nlm.nih.gov/projects/gap/cgi-bin/analysis.cgi?id=pha002742
Pulmonary	rcrosssectional	rfev1adjsm10	residual from predicted FEV1 at latest exam in 10+ packyear smokers	http://www.ncbi.nlm.nih.gov/projects/gap/cgi-bin/analysis.cgi?id=pha002743
Pulmonary	rcrosssectional	rfvcadj	residual from predicted FVC at latest exam	http://www.ncbi.nlm.nih.gov/projects/gap/cgi-bin/analysis.cgi?id=pha002744
Pulmonary	rcrosssectional	rfvcadjsm10	residual from predicted FVC at latest exam in 10+ packyear smokers	http://www.ncbi.nlm.nih.gov/projects/gap/cgi-bin/analysis.cgi?id=pha002745
Pulmonary	rcrosssectional	rratioadjsm10	residual from predicted FEV1/FVC at latest exam in 10+ packyear smokers	http://www.ncbi.nlm.nih.gov/projects/gap/cgi-bin/analysis.cgi?id=pha002746
Pulmonary	rcrosssectional	rratioadjw	residual from predicted FEV1/FVC at latest exam, linkage	http://www.ncbi.nlm.nih.gov/projects/gap/cgi-bin/analysis.cgi?id=pha002747
Pulmonary	singleexam	ppfev1single	percent predicted FEV1, exam: offspring 6, cohort 17	http://www.ncbi.nlm.nih.gov/projects/gap/cgi-bin/analysis.cgi?id=pha002748
Pulmonary	singleexam	ppfvcsingle	percent predicted FVC, exam: offspring 6, cohort 17	http://www.ncbi.nlm.nih.gov/projects/gap/cgi-bin/analysis.cgi?id=pha002749
Pulmonary	singleexam	ppratiosinglew	percent predicted FEV1/FVC, exam: offspring 6, cohort 17, linkage	http://www.ncbi.nlm.nih.gov/projects/gap/cgi-bin/analysis.cgi?id=pha002750
Pulmonary	singleexam	rfev1single	residual from predicted FEV1, exam: offspring 6, cohort 17	http://www.ncbi.nlm.nih.gov/projects/gap/cgi-bin/analysis.cgi?id=pha002751
Pulmonary	singleexam	rfvcsingle	residual from predicted FVC, exam: offspring 6, cohort 17	http://www.ncbi.nlm.nih.gov/projects/gap/cgi-bin/analysis.cgi?id=pha002752
Pulmonary	singleexam	rratiosinglew	residual from predicted FEV1/FVC, exam: offspring 6, cohort 17, linkage	http://www.ncbi.nlm.nih.gov/projects/gap/cgi-bin/analysis.cgi?id=pha002753
Pulmonary	Sleepiness	ess	Epworth Sleepiness Scale, unadjusted	http://www.ncbi.nlm.nih.gov/projects/gap/cgi-bin/analysis.cgi?id=pha002754
Pulmonary	Sleepiness	essresid1	Epworth Sleepiness Scale, adjusted A	http://www.ncbi.nlm.nih.gov/projects/gap/cgi-bin/analysis.cgi?id=pha002755
Pulmonary	Sleepiness	essresid2	Epworth Sleepiness Scale, adjusted B	http://www.ncbi.nlm.nih.gov/projects/gap/cgi-bin/analysis.cgi?id=pha002756
Pulmonary	Sleepiness	essresid3	Epworth Sleepiness Scale, adjusted C	http://www.ncbi.nlm.nih.gov/projects/gap/cgi-bin/analysis.cgi?id=pha002757
RENALENDO	ENDO	DHEASAS3	DHEAS adjusted for age and sex	http://www.ncbi.nlm.nih.gov/projects/gap/cgi-bin/analysis.cgi?id=pha002758
RENALENDO	ENDO	DHEASMV3	DHEAS MV adjusted	http://www.ncbi.nlm.nih.gov/projects/gap/cgi-bin/analysis.cgi?id=pha002759
RENALENDO	ENDO	FSHAS3	FSH in men or post meno women no hormones, age-sex adjusted	http://www.ncbi.nlm.nih.gov/projects/gap/cgi-bin/analysis.cgi?id=pha002760
RENALENDO	ENDO	FSHMV3	FSH in men or post meno women no hormones, MV adjusted	http://www.ncbi.nlm.nih.gov/projects/gap/cgi-bin/analysis.cgi?id=pha002761
RENALENDO	ENDO	LHAS3	LH in men or post-meno women no hormones, age-sex adjusted	http://www.ncbi.nlm.nih.gov/projects/gap/cgi-bin/analysis.cgi?id=pha002762
RENALENDO	ENDO	LHMV3	LH in men or post-meno women no hormones, MV adjusted	http://www.ncbi.nlm.nih.gov/projects/gap/cgi-bin/analysis.cgi?id=pha002763
RENALENDO	ENDO	TSHLNASNL3	TSH ex3 log trans plus normalized deviates, age-sex adjusted	http://www.ncbi.nlm.nih.gov/projects/gap/cgi-bin/analysis.cgi?id=pha002764
RENALENDO	ENDO	TSHLNASNL4	TSH ex4 log transformed plus normalized deviates, age-sex adjusted	http://www.ncbi.nlm.nih.gov/projects/gap/cgi-bin/analysis.cgi?id=pha002765
RENALENDO	ENDO	TSHLNMVNL3	TSH ex3 log trans plus normalized deviates, MV adjusted	http://www.ncbi.nlm.nih.gov/projects/gap/cgi-bin/analysis.cgi?id=pha002766
RENALENDO	ENDO	TSHLNMVNL4	TSH ex4 log trans plus normalized deviates, MV adjusted	http://www.ncbi.nlm.nih.gov/projects/gap/cgi-bin/analysis.cgi?id=pha002767
RENALENDO	ENDO	TSHMEAN34LNASNL	Mean TSH log transformed plus normalized deviates, ex 3 and 4, age sex adj	http://www.ncbi.nlm.nih.gov/projects/gap/cgi-bin/analysis.cgi?id=pha002768
RENALENDO	ENDO	TSHMEAN34LNMVNL	Mean TSH ex3 and 4 log transformed plus normalized deviates MV adjusted	http://www.ncbi.nlm.nih.gov/projects/gap/cgi-bin/analysis.cgi?id=pha002769
RENALENDO	RENAL	CALCIUMAS2	Calcium, ex2, age-sex adjusted	http://www.ncbi.nlm.nih.gov/projects/gap/cgi-bin/analysis.cgi?id=pha002770
RENALENDO	RENAL	CALCIUMMV2	Calcium, ex2, age-sex-creatinine adjusted	http://www.ncbi.nlm.nih.gov/projects/gap/cgi-bin/analysis.cgi?id=pha002771
RENALENDO	RENAL	CHNGSCR27ASWIN	Change in creatinine ex2 to 7, age-sex adjusted	http://www.ncbi.nlm.nih.gov/projects/gap/cgi-bin/analysis.cgi?id=pha002772
RENALENDO	RENAL	CHNGSCR27MVWIN	Change in creatinine ex2 to 7, MV adjusted	http://www.ncbi.nlm.nih.gov/projects/gap/cgi-bin/analysis.cgi?id=pha002773
RENALENDO	RENAL	CYSCAS7	Cystatin C, ex 7, age-sex adjusted	http://www.ncbi.nlm.nih.gov/projects/gap/cgi-bin/analysis.cgi?id=pha002808
RENALENDO	RENAL	CYSCMV7	Cystatin C, ex 7, MV adjusted	http://www.ncbi.nlm.nih.gov/projects/gap/cgi-bin/analysis.cgi?id=pha002809
RENALENDO	RENAL	GFRAS7	GFR ex 7, age-sex adjusted	http://www.ncbi.nlm.nih.gov/projects/gap/cgi-bin/analysis.cgi?id=pha002774
RENALENDO	RENAL	GFRASNL5	GFR ex 5 normalized deviates, age-sex adjusted	http://www.ncbi.nlm.nih.gov/projects/gap/cgi-bin/analysis.cgi?id=pha002775
RENALENDO	RENAL	GFRASNL6	GFR ex6 normalized deviates age-sex adjusted	http://www.ncbi.nlm.nih.gov/projects/gap/cgi-bin/analysis.cgi?id=pha002776
RENALENDO	RENAL	GFRASWIN2	GFR ex2, winsorized, age-sex adjusted	http://www.ncbi.nlm.nih.gov/projects/gap/cgi-bin/analysis.cgi?id=pha002777
RENALENDO	RENAL	GFRMV7	GFR ex7, MV adjusted	http://www.ncbi.nlm.nih.gov/projects/gap/cgi-bin/analysis.cgi?id=pha002778
RENALENDO	RENAL	GFRMVNL5	GFR ex5 normalized deviates, MV adjusted	http://www.ncbi.nlm.nih.gov/projects/gap/cgi-bin/analysis.cgi?id=pha002779
RENALENDO	RENAL	GFRMVNL6	GFR ex6 normalized deviates MV adjusted	http://www.ncbi.nlm.nih.gov/projects/gap/cgi-bin/analysis.cgi?id=pha002780
RENALENDO	RENAL	GFRMVWIN2	GFR ex2, winsorized, MV adjusted	http://www.ncbi.nlm.nih.gov/projects/gap/cgi-bin/analysis.cgi?id=pha002781
RENALENDO	RENAL	MeanGFRAS2567WIN	Mean GFR ex 2,5,6,7 age-sex adjusted	http://www.ncbi.nlm.nih.gov/projects/gap/cgi-bin/analysis.cgi?id=pha002782

Group	Category	Trait Label	Name	Linkage Link
RENALENDO	RENAL	MeanGFRMV2567WIN	Mean GFR ex 2,5,6,7,MV adjusted	http://www.ncbi.nlm.nih.gov/projects/gap/cgi-bin/analysis.cgi?id=pha002783
RENALENDO	RENAL	MeanSCR2567LNASWIN	Mean creatinine ex 2,5,6,7 age-sex adjusted	http://www.ncbi.nlm.nih.gov/projects/gap/cgi-bin/analysis.cgi?id=pha002784
RENALENDO	RENAL	MeanSCR2567LNMVWIN	Mean creatinine ex 2,5,6,7, MV adjusted	http://www.ncbi.nlm.nih.gov/projects/gap/cgi-bin/analysis.cgi?id=pha002785
RENALENDO	RENAL	MeanURICACID12AS	Mean uric acid, ex 1 and 2, age-sex adjusted	http://www.ncbi.nlm.nih.gov/projects/gap/cgi-bin/analysis.cgi?id=pha002786
RENALENDO	RENAL	MeanURICACID12MV	Mean uric acid, ex 1 and 2, MV adjusted	http://www.ncbi.nlm.nih.gov/projects/gap/cgi-bin/analysis.cgi?id=pha002787
RENALENDO	RENAL	PHOSAS2	Phos, ex 2, age-sex adjusted	http://www.ncbi.nlm.nih.gov/projects/gap/cgi-bin/analysis.cgi?id=pha002788
RENALENDO	RENAL	PHOSMV2	Phos, ex 2, MV adjusted	http://www.ncbi.nlm.nih.gov/projects/gap/cgi-bin/analysis.cgi?id=pha002789
RENALENDO	RENAL	SCRLNASNL5	Creatinine, ex5 normalized deviates, age-sex adjusted	http://www.ncbi.nlm.nih.gov/projects/gap/cgi-bin/analysis.cgi?id=pha002790
RENALENDO	RENAL	SCRLNASNL6	Ceatinine, ex6 normalized deviates, age-sex adjusted	http://www.ncbi.nlm.nih.gov/projects/gap/cgi-bin/analysis.cgi?id=pha002791
RENALENDO	RENAL	SCRLNASWIN2	Creatinine ex2, log transformed and winsorized, age-sex adjusted	http://www.ncbi.nlm.nih.gov/projects/gap/cgi-bin/analysis.cgi?id=pha002792
RENALENDO	RENAL	SCRLNASWIN7	Creatinine ex7, log transformed and winsorized, age-sex adjusted	http://www.ncbi.nlm.nih.gov/projects/gap/cgi-bin/analysis.cgi?id=pha002793
RENALENDO	RENAL	SCRLNMVNL5	GFR ex 5 uncalibrated creat normalized deviates, MV adjusted	http://www.ncbi.nlm.nih.gov/projects/gap/cgi-bin/analysis.cgi?id=pha002794
RENALENDO	RENAL	SCRLNMVNL6	GFR ex6 uncalibrated creat normalied deviates, MV adjusted	http://www.ncbi.nlm.nih.gov/projects/gap/cgi-bin/analysis.cgi?id=pha002795
RENALENDO	RENAL	SCRLNMVWIN2	Creatinine ex 2 log transformed winsorized MV adjusted	http://www.ncbi.nlm.nih.gov/projects/gap/cgi-bin/analysis.cgi?id=pha002796
RENALENDO	RENAL	SCRLNMVWIN7	Creatinine ex 7 uncalibrated MV adjusted	http://www.ncbi.nlm.nih.gov/projects/gap/cgi-bin/analysis.cgi?id=pha002797
RENALENDO	RENAL	UAELNASNL6	Log-transformed UAE, age-sex adjusted, linkage	http://www.ncbi.nlm.nih.gov/projects/gap/cgi-bin/analysis.cgi?id=pha002798
RENALENDO	RENAL	UAELNHTNASNL6	Log-transformed UAE in HTN enriched sample, age-sex adjusted, linkage	http://www.ncbi.nlm.nih.gov/projects/gap/cgi-bin/analysis.cgi?id=pha002799
RENALENDO	RENAL	UAELNHTNMVNL6	Log-transformed UAE in HTN enriched sample, MV adjusted-linkage	http://www.ncbi.nlm.nih.gov/projects/gap/cgi-bin/analysis.cgi?id=pha002800
RENALENDO	RENAL	UAELNMVNL6	Log-transformed UAE MV adjusted-linkage	http://www.ncbi.nlm.nih.gov/projects/gap/cgi-bin/analysis.cgi?id=pha002801
RENALENDO	RENAL	UNALNASWIN6	Urinary sodium, age-sex adjusted	http://www.ncbi.nlm.nih.gov/projects/gap/cgi-bin/analysis.cgi?id=pha002802
RENALENDO	RENAL	UNALNMVWIN6	Urinary sodium, MV adjusted	http://www.ncbi.nlm.nih.gov/projects/gap/cgi-bin/analysis.cgi?id=pha002803
RENALENDO	RENAL	URICACIDAS1	Uric acid, ex 1, age-sex adjusted	http://www.ncbi.nlm.nih.gov/projects/gap/cgi-bin/analysis.cgi?id=pha002804
RENALENDO	RENAL	URICACIDAS2	Uric acid, ex 2, age-sex adjusted	http://www.ncbi.nlm.nih.gov/projects/gap/cgi-bin/analysis.cgi?id=pha002805
RENALENDO	RENAL	URICACIDMV1	Uric acid, ex1, MV adjusted	http://www.ncbi.nlm.nih.gov/projects/gap/cgi-bin/analysis.cgi?id=pha002806
RENALENDO	RENAL	URICACIDMV2	Uric acid, ex2, MV adjusted	http://www.ncbi.nlm.nih.gov/projects/gap/cgi-bin/analysis.cgi?id=pha002807
SubclinCVD	Ankle-brachialindex	RANKLEBI6	Ankle-brachial index, cycle 6, age and sex-djusted	http://www.ncbi.nlm.nih.gov/projects/gap/cgi-bin/analysis.cgi?id=pha002810
SubclinCVD	Ankle-brachialindex	RANKLEBI6MV	Ankle-brachial index, cycle 6, multivariable-adjusted	http://www.ncbi.nlm.nih.gov/projects/gap/cgi-bin/analysis.cgi?id=pha002811
SubclinCVD	Ankle-brachialindex	RANKLEBI7	Ankle-brachial index, cycle 7, age- and sex-adjusted	http://www.ncbi.nlm.nih.gov/projects/gap/cgi-bin/analysis.cgi?id=pha002812
SubclinCVD	Ankle-brachialindex	RANKLEBI7MV	Ankle-brachial index, cycle 7, multivariable-adjusted	http://www.ncbi.nlm.nih.gov/projects/gap/cgi-bin/analysis.cgi?id=pha002813
SubclinCVD	BrainMRI	BMRILWMHVC	Log white matter hyperintensity to intracranial volume ratio, age and sex	http://www.ncbi.nlm.nih.gov/projects/gap/cgi-bin/analysis.cgi?id=pha002814
SubclinCVD	BrainMRI	BMRILWMHVMV	Log white matter hyperintensity to intracranial volume ratio, multivariable	http://www.ncbi.nlm.nih.gov/projects/gap/cgi-bin/analysis.cgi?id=pha002815
SubclinCVD	BrainMRI	BMRIZLWMHVC	Z-score log white matter hyperintensity volume ratio, sex adjusted	http://www.ncbi.nlm.nih.gov/projects/gap/cgi-bin/analysis.cgi?id=pha002816
SubclinCVD	BrainMRI	BMRIZLWMHVMV	Z-score log white matter hyperintensity volume ratio, multivariable	http://www.ncbi.nlm.nih.gov/projects/gap/cgi-bin/analysis.cgi?id=pha002817
SubclinCVD	Carotid	RNKCAROTBULBAS6	Carotid bulb IMT mean max, cycle 6, log transformed age and sex-adjusted	http://www.ncbi.nlm.nih.gov/projects/gap/cgi-bin/analysis.cgi?id=pha002818
SubclinCVD	Carotid	RNKCAROTBULBMV6	Carotid bulb IMT mean max, cycle 6, log transformed multivariable-adjusted	http://www.ncbi.nlm.nih.gov/projects/gap/cgi-bin/analysis.cgi?id=pha002819
SubclinCVD	Carotid	RNKCAROTCCAMAXAS6	Common carotid IMT mean max, cycle 6, log transformed age and sex-adjusted	http://www.ncbi.nlm.nih.gov/projects/gap/cgi-bin/analysis.cgi?id=pha002820
SubclinCVD	Carotid	RNKCAROTCCAMAXMV6	Common carotid IMT mean max, cycle 6, log transformed multivariable-adjusted	http://www.ncbi.nlm.nih.gov/projects/gap/cgi-bin/analysis.cgi?id=pha002821
SubclinCVD	Carotid	RNKCAROTCCAMEANAS6	Common carotid IMT mean mean, cycle 6, log transformed age and sex-adjusted	http://www.ncbi.nlm.nih.gov/projects/gap/cgi-bin/analysis.cgi?id=pha002822
SubclinCVD	Carotid	RNKCAROTCCAMEANMV6	Common carotid IMT mean mean, cycle 6, log transformed multivariable-adjusted	http://www.ncbi.nlm.nih.gov/projects/gap/cgi-bin/analysis.cgi?id=pha002823
SubclinCVD	Carotid	RNKCAROTICAMAXAS6	Internal carotid IMT mean max, cycle 6, log transformed age and sex-adjusted	http://www.ncbi.nlm.nih.gov/projects/gap/cgi-bin/analysis.cgi?id=pha002824
SubclinCVD	Carotid	RNKCAROTICAMAXMV6	Internal carotid IMT mean max, cycle 6, log transformed multivariable-adjusted	http://www.ncbi.nlm.nih.gov/projects/gap/cgi-bin/analysis.cgi?id=pha002825
SubclinCVD	Carotid	RNKCAROTICAMEANAS6	Internal carotid IMT mean mean, cycle 6, log transformed age and sex-adjusted	http://www.ncbi.nlm.nih.gov/projects/gap/cgi-bin/analysis.cgi?id=pha002826
SubclinCVD	Carotid	RNKCAROTICAMEANMV6	Internal carotid IMT mean mean, cycle 6, log transformed multivariable-adjusted	http://www.ncbi.nlm.nih.gov/projects/gap/cgi-bin/analysis.cgi?id=pha002827
SubclinCVD	Carotid	RNKCAROTSTENAS6	Maximum Carotid Stenosis, cycle 6, age and sex-adjusted	http://www.ncbi.nlm.nih.gov/projects/gap/cgi-bin/analysis.cgi?id=pha002828
SubclinCVD	Carotid	RNKCAROTSTENMV6	Maximum Carotid Stenosis, cycle 6 multivariable-adjusted	http://www.ncbi.nlm.nih.gov/projects/gap/cgi-bin/analysis.cgi?id=pha002829
SubclinCVD	CT	RESMDCTAACAS7	Mean Agatston AAC score, MDCT ~cycle 7, log transform, age and sex-adjusted	http://www.ncbi.nlm.nih.gov/projects/gap/cgi-bin/analysis.cgi?id=pha002830
SubclinCVD	CT	RESMDCTAACMV7	Mean Agatston AAC score, MDCT ~cycle 7, log transform multivariable-adjusted	http://www.ncbi.nlm.nih.gov/projects/gap/cgi-bin/analysis.cgi?id=pha002831
SubclinCVD	CT	RESMDCTCACAS7	Mean Agatston CAC score, MDCT ~cycle 7, log transform age and sex-adjusted	http://www.ncbi.nlm.nih.gov/projects/gap/cgi-bin/analysis.cgi?id=pha002832
SubclinCVD	CT	RESMDCTCACMAXAS7	Max Agatston CAC score, MDCT ~cycle 7, log transform age and sex-adjusted	http://www.ncbi.nlm.nih.gov/projects/gap/cgi-bin/analysis.cgi?id=pha002833
SubclinCVD	CT	RESMDCTCACMAXMV7	Max Agatston CAC score, MDCT ~cycle 7, log transform multivariable-adjusted	http://www.ncbi.nlm.nih.gov/projects/gap/cgi-bin/analysis.cgi?id=pha002834
SubclinCVD	CT	RESMDCTCACMV7	Mean Agatston CAC score, MDCT ~cycle 7, log transform multivariable-adjusted	http://www.ncbi.nlm.nih.gov/projects/gap/cgi-bin/analysis.cgi?id=pha002835
